# Integrated care pathway in individuals with Long COVID: STIMULATE-ICP, a cluster-randomized, phase 3 trial

**DOI:** 10.1038/s41591-026-04552-x

**Published:** 2026-07-28

**Authors:** STIMULATE-ICP Consortium, Amitava Banerjee, Gordon Prescott, Emma Wall, Caroline Leigh Watkins, Melissa Heightman, Alison Gibbon, Al-Tahoor Butt, Amanpreet Sarna, Amir Manzelat, Anand Viswanathan, Andrea Dennis, Andrew Clegg, Andrew Williams, Angela Green, Annalisa Parabita, Antony Loveless, Anu Chandra, Ashkan Dashtban, Charles Latchford, Charlotte Adams-Heath, Chris Robson, Christina M. van der Feltz-Cornelis, Christopher Brown, Claire Hooper, Dan Cuthbertson, Dan Wootton, William David Strain, David Sunkersing, Deepak Ravindran, Denise Forshaw, Donna Clutterbuck, Eamonn Foley, Elena Bax, Elizabeth Murray, Emily Attree, Emily Fraser, Emma Mi, Fauzia Begum, Florence Mutesva, Francesco Gambino, Gail Allsopp, Gregory Y. H. Lip, Hakim-Moulay Dehbi, Han-I Wang, Helen Grice, Henry Goodfellow, Hugh Montgomery, Ileana L. Selejan, Jagdeep Sahota, Jai Prashar, Janet Scott, Jasmine Hayer, Jennifer Sweetman, Joanna Peel, Joanna Wright, Jonathan Waywell, Josie Newton, Kamlesh Khunti, Keri Hildick, Kim Horstmanshof, Kiran Khan, Kumaran Balasundaram, Laura Pasea, Lauren O’Mahoney, Lere Fisher, Lyth Hishmeh, Mag Leahy, Manuel Gomes, Marija Pantelic, Mark Gabbay, Matthew Robert Burns, Maya Leach, Mel Ramasawmy, Michael Crooks, Michael Zandi, Mohamed Mohamed, Nataliia Kryvutenko, Natalie Smith, Nefyn Williams, Nisreen A. Alwan, Ondine Sherwood, Pablo Fernandez Medina, Paula Lorgelly, Phil Cumberlidge, Rachael A. Evans, Rachel Botell, Rachel Hext, Rachel Nyam, Rachel Williams, Rajarshi Banerjee, Rebecca Livingston, Rita Mallinson, Robert Bell, Rob Suriano, Samantha Eccles, Sarah Clegg, Sophie Burchell-Kirk, Sophie Equi, Stephen Cone, Terence Elliott-Cooper, Toby Hillman, Tushy Kailayanathan, Valerio Benedetto, Yi Mu

**Affiliations:** 1 University College London, London, UK; 2 Lancashire Clinical Trials Unit, University of Lancashire, Preston, UK; 3 The Francis Crick Institute, London, UK; 4 University College London Hospitals NHS Trust, London, UK; 5 Tameside and Glossop Integrated Care NHS Foundation Trust, Tameside General Hospital, Lancashire, UK; 6 Royal Free NHS Foundation Trust, London, UK; 7 Sheffield Teaching Hospitals NHS Foundation Trust, Northern General Hospital, Sheffield, UK; 8 Perspectum Ltd, Oxford, UK; 9 Applied Health Research Hub, University of Lancashire, Preston, UK; 10 Patient and Public Representative, C/O Institute of Health Informatics, University College London, London, UK; 11 Hull University Teaching Hospitals NHS Foundation Trust, Hull Royal Infirmary, Hull, UK; 12 Derbyshire Community Health Services NHS Foundation Trust, Ash Green Learning Disability Centre, Chesterfield, UK; 13 Living With Ltd, Brighton, UK; 14 Department of Health Sciences, University of York, York, UK; 15 University of Liverpool, Liverpool, UK; 16 University of Exeter, Exeter, UK; 17 Reading Central Primary Care Network, Pembroke Surgery, Reading, UK; 18 University of Southampton, Southampton, UK; 19 University Hospitals Sussex NHS Foundation Trust, Worthing Hospital, Worthing, UK; 20 Oxford University Hospitals NHS Foundation Trust, John Radcliffe Hospital, Oxford, UK; 21 NHS England, London, UK; 22 Livewell Southwest, Plymouth, UK; 23 Department for Work and Pensions, London, UK; 24 Barts Health NHS Trust, The Royal London Hospital, London, UK; 25 NHS Highlands, Inverness, UK; 26 University of Leicester, Leicester, UK; 27 The Princess Alexandra Hospital NHS Trust, Harlow, UK; 28 University Hospitals of Leicester NHS Trust, Leicester Royal Infirmary, Leicester, UK; 29 University of Sussex, Brighton, UK; 30 University of Hull, Kingston upon Hull, UK; 31 Long Covid SOS, Aldridge, UK; 32 Department of Economics, The University of Auckland, Auckland, New Zealand; 33 Vauxhall Primary Care, Liverpool, UK; 34 Royal Devon and Exeter NHS Foundation Trust, Royal Devon and Exeter Hospital (Wonford), Exeter, UK

**Keywords:** Viral infection, Rehabilitation, Magnetic resonance imaging, Randomized controlled trials, SARS-CoV-2

## Abstract

Combining multidisciplinary care plans into integrated care pathways (ICPs) is scalable, generalizable and effective for several long-term conditions, but not evaluated in Long COVID (LC). Our phase 3, cluster-randomized, multicenter clinical trial investigated the effectiveness of ICP interventions for LC. Recruitment of adults ≥18 years with LC was conducted in 6 National Health Service LC clinics in England with specialist ICPs. The intervention arms were (i) multi-organ magnetic resonance imaging (MRI; Coverscan), (ii) digital rehabilitation (Living with COVID Recovery), (iii) both multi-organ MRI and digital rehabilitation, and (iv) neither multi-organ MRI nor digital rehabilitation (usual care); cluster randomizing at primary care network (PCN) level to deliver interventions as ‘standard of care’ in that area. The primary endpoint was mean Fatigue Assessment Scale (FAS) at 12 weeks. Secondary endpoints included FAS at 24 weeks and EQ-5D-5L visual assessment scale at 12 and 24 weeks. A total of 1,152 participants from allocated PCNs consented to data collection, and 122 PCN clusters were allocated to the multi-organ MRI (33 PCNs), digital rehabilitation (32 PCNs), ‘both’ (27 PCNs) and ‘neither’ (30 PCNs) arms. Baseline FAS was equivalent across groups (mean 35.8, s.d. 8.21, 66.4% female). Overall, the primary outcome was achieved in all arms, with scores improving by 4.5 points to 31.3 (s.d. 9.31) at 12 weeks. Compared with usual care, effects on 12-week FAS were −0.18 (95% confidence interval −0.72, 1.09; *P* = 0.69) with multi-organ MRI; −0.53 (−1.42, 0.36; *P* = 0.25) with digital rehabilitation; and −0.17 (−1.06, 0.72; *P* = 0.71) with their interaction. Absolute differences for other interventions from usual care were modest. There were no serious adverse events related to ICP interventions. Multidisciplinary holistic clinical care is associated with fatigue reduction in LC, irrespective of multi-organ MRI. Digital rehabilitation could be useful in longer-term LC management. Large, multi-site trials of optimal ICP interventions are feasible, but further trials are required. ISRCTN identifier: ISRCTN10665760.

## Abstract (web summary)

This cluster-randomized phase 3 trial examining the effectiveness of integrated care pathways comparing usual care with the addition of MRI imaging (Coverscan), or a digitally enabled rehabilitation platform app, or both (Coverscan + app), or neither, found specialist Long COVID care improved fatigue response in all arms.

## Main

Many countries have implemented ICPs^1–3^ due to population aging and increasing burden of multiple long-term conditions (LTCs)^4^. ICPs use “structured, multidisciplinary care plans coordinated across specialties, investigations, treatments and rehabilitation”^5^ with improved clinical and patient-reported outcomes^6,7^ in LTCs, such as diabetes and chronic obstructive pulmonary disease. Since the early coronavirus disease 2019 (COVID-19) pandemic, longer-term complications, termed LC, posed dual health system challenges globally. First, underlying mechanisms and definitions remain unclear^8^. Second, the burden on individuals and systems necessitated pragmatic care development and evaluation^9,10^. From 2021, a network of 100 LC clinics was started in England for ICP delivery.

Patients and clinicians have advocated for more ICP research^11^. ICPs can ‘bundle’ evidence-based interventions, achieving consistency of care and reducing demands on staff (and patients). However, ICPs are only as good as their component investigations and interventions and coherence between components. Therefore, it is important to keep iterating the pathway.

Early in the pandemic, longer-term, multi-organ impairment was documented^12–14^ after severe acute respiratory syndrome coronavirus 2 infection. Early diagnosis and management of multi-organ impairment could reduce the overall burden of healthcare associated with LC^15^. Moreover, there was early recognition that rehabilitation, both self-administered and digital, had a role in LC care^16^. Although LC clinics in England offered a multidisciplinary, patient-centered ICP, many patients and clinicians have sought alternative management with little evidence^17^. There was a need to evaluate whether diagnostic and rehabilitation tools, already in use, could enhance or transform existing LC ICPs. Based on consensus statements by patients, health professionals and current evidence base, this need remains an important gap in evidence and implementation^7,18–21^.

Coverscan, a multi-organ MRI of heart, liver, kidney, pancreas and spleen, received emergency use authorization in 2021 as an LC diagnostic test^13^. It quantifies multi-organ characteristics using a single noninvasive scan (without intravenous contrast), providing clinicians with a report from multiparametric magnetic resonance data for easy assessment of multi-organ function relative to reference ranges. The potential of an early multi-organ scan to improve LC outcomes was highlighted but had not been evaluated^15,22^. Living With LC is a community-based, digital rehabilitation platform, which was already being evaluated in certain LC clinics in the United Kingdom from 2020 onward^16,23–26^. It involves a mobile app for individuals with LC, which collects their symptoms and uses that information to deliver tailored, personalized advice; a dashboard that allows clinicians to review patient’s progress and communicate with them; and a clinical pathway that specifies how patients can safely receive this remote-supported care^24^.

In a cluster-randomized trial in nonhospitalized adults with LC, we evaluated efficacy of either/or diagnostic (multi-organ MRI scan) and treatment (digital rehabilitation) interventions already being used in clinical practice, compared with an existing ICP via specialist LC clinics in England for fatigue management^15^ (Supplementary Information, pp. 1–121).

## Results

### Patient disposition

**Fig. 1:**
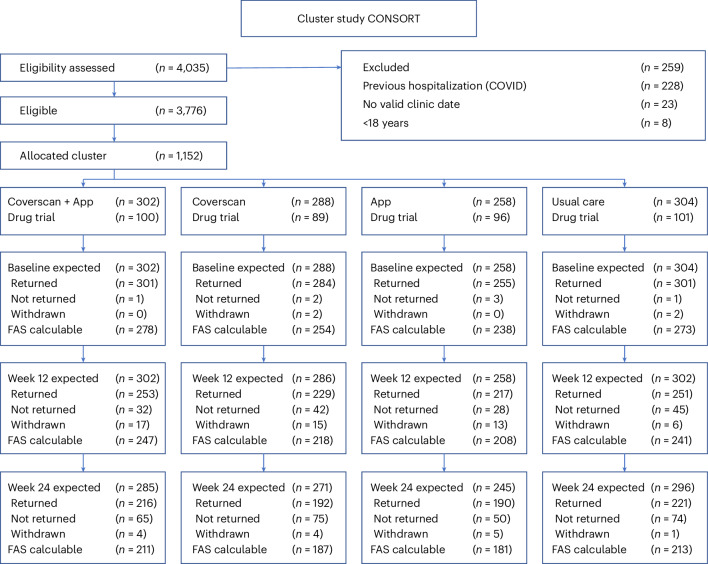
Study population for the STIMULATE-ICP cluster-randomized trial. CONSORT diagram for the Symptoms, Trajectory, Inequalities and Management: Understanding Long-COVID to Address and Transform Existing Integrated Care Pathways (STIMULATE-ICP) trial.

A total of 6,934 potential participants were screened. The first and last participants were enrolled on 22 August 2022 and 7 August 2024, respectively. All those eligible were offered the allocation that was uplift of care in their PCN. Of those eligible, 1,152 adults aged over 18 years with LC, defined as persistent, otherwise unexplained post-COVID symptoms for ≥4 weeks (positive severe acute respiratory syndrome coronavirus 2 test not required for diagnosis), and eligible at first referral to one of six National Health Service (NHS) LC clinics, gave consent for their data to be used for the ICP trial and were allocated to usual care with multi-organ MRI (*n* = 288), digital rehabilitation (*n* = 258), both (*n* = 302) or neither (*n* = 304; Fig. 1) as standard of care in their PCN. A total of 122 PCN clusters were allocated to the four arms (33, 32, 27 and 30 PCNs, in the order above). Participants were referred from only 114 of the 122 PCNs (in the ratio of 31, 30, 27 and 28, respectively). Where there was at least one participant, the median number of participants per PCN was 8 (interquartile range (IQR) 4–14, range 1–40). There were no substantial differences in the IQR of participants per PCN between treatment groups. Mean age was 48.9 years (s.d. 13.8), 765 (66.4%) were female, and 154 (14.4%) were from minority ethnic groups (Table 1). Asthma (19.2%), allergy (19.7%) and mental health conditions (anxiety, 14.1%; depression, 23.4%; concurrent anxiety and depression, 8.2%) and diabetes (5.9%) were common. Overall, 1,036 (94.4%) had ≥1 COVID-19 vaccine dose at baseline. The median time between initial infection and trial recruitment/LC symptom duration was 394 days (IQR 238–717 days). The median number of LC symptoms and mean baseline FAS were 9 (IQR 7–9) and 35.8 (s.d. 8.2), respectively. Common baseline medications were for depression (*n* = 178, 15.5%), asthma (*n* = 203, 17.6%), acid reflux (*n* = 160, 13.9%), pain (*n* = 201, 17.4%), supplements (*n* = 272, 23.6%) and LC symptom management (*n* = 65, 5.6%; Table 1). Of those in the ICP trial, 395 were also randomized into the drug trial, receiving usual care (no drug), famotidine with loratadine, colchicine or rivaroxaban in a 1:1:1:1 ratio (Fig. 1).

**Table 1:** Demographic details of included participants at baseline by intervention

	Coverscan + App *n* = 302	Coverscan *n* = 288	App *n* = 258	Usual care *n* = 304	All *n* = 1,152
Age at baseline, mean (s.d.)	48.9 (13.60)	48.9 (14.27)	47.1 (13.50)	50.3 (13.53)	48.9 (13.75)
Female at birth, *n* (%)	196 (64.9)	204 (70.8)	162 (62.8)	203 (66.8)	765 (66.4)
GP IMD quintile: 1—most deprived, *n* (%)	31 (10.3)	54 (18.8)	71 (27.5)	89 (29.3)	245 (21.3)
2, *n* (%)	95 (31.5)	95 (33.0)	50 (19.4)	58 (19.1)	298 (25.9)
3, *n* (%)	58 (19.2)	51 (17.7)	51 (19.8)	81 (26.6)	241 (20.9)
4, *n* (%)	88 (29.1)	62 (21.5)	41 (15.9)	24 (7.9)	215 (18.7)
5—least deprived, *n* (%)	30 (9.9)	26 (9.0)	45 (17.4)	52 (17.1)	153 (13.3)
Ethnicity *n* (%): white	247 (87.0)	222 (83.8)	202 (84.5)	247 (87.0)	918 (85.6)
Mixed	11 (3.9)	14 (5.3)	14 (5.9)	9 (3.2)	48 (4.5)
Asian	16 (5.6)	17 (6.4)	14 (5.9)	17 (6.0)	64 (6.0)
Black	7 (2.5)	7 (2.6)	6 (2.5)	5 (1.8)	25 (2.3)
Other	3 (1.1)	5 (1.9)	3 (1.3)	6 (2.1)	17 (1.6)
FAS total score at baseline, mean (s.d.)	34.6 (8.57)	35.5 (8.61)	36.9 (7.25)	36.5 (8.08)	35.8 (8.21)
Vaccinated against COVID, *n* (%)	274 (93.5)	260 (94.2)	233 (95.1)	269 (94.7)	1036 (94.4)
LC—duration/days, median (IQR)	415 (256, 779)	437 (255, 798)	378 (248, 678)	347 (223, 645)	394 (238, 717)
Number of symptoms, median (IQR)	9.0 (7.0, 9.0)	9.0 (7.0, 9.0)	9.0 (7.0, 10.0)	9.0 (8.0, 10.0)	9.0 (7.0, 9.0)
**Self-reported COVID infections pre-clinic,** ***n*** **(%)**	296 (98.0)	277 (96.2)	248 (96.1)	291 (95.7)	1112 (96.5)
1, *n* (%)	165 (55.7)	154 (55.6)	134 (54.0)	171 (58.8)	624 (56.1)
2, *n* (%)	80 (27.0)	87 (31.4)	72 (29.0)	85 (29.2)	324 (29.1)
3 or more, *n* (%)	51 (17.2)	36 (13.0)	42 (16.9)	35 (12.0)	164 (14.7)
BMI, median (IQR)	26.9 (23.1, 31.0)	27.2 (24.1, 31.3)	25.7 (22.8, 29.3)	27.2 (23.6, 31.9)	26.7 (23.4, 30.9)
**Medical history,** *n* (%)					
Depression	75 (24.8)	68 (23.6)	62 (24.0)	64 (21.1)	269 (23.4)
Anxiety	47 (15.6)	36 (12.5)	41 (15.9)	38 (12.5)	162 (14.1)
Anxiety and depression	32 (10.6)	16 (5.6)	24 (9.3)	23 (7.6)	95 (8.2)
Hypertension	32 (10.6)	25 (8.7)	32 (12.4)	36 (11.8)	125 (10.9)
Allergies	61 (20.2)	64 (22.2)	48 (18.6)	54 (17.8)	227 (19.7)
Migraine	20 (6.6)	29 (10.1)	19 (7.4)	25 (8.2)	93 (8.1)
Asthma	54 (17.9)	55 (19.1)	55 (21.3)	57 (18.8)	221 (19.2)
Diabetes	16 (5.3)	18 (6.2)	15 (5.8)	19 (6.2)	68 (5.9)
Thyroid disorder	30 (9.9)	28 (9.7)	17 (6.6)	20 (6.6)	95 (8.2)
**Drug indication**, *n* (%)					
Depression	42 (13.9)	46 (16.0)	41 (15.9)	49 (16.1)	178 (15.5)
Anxiety	12 (4.0)	18 (6.2)	19 (7.4)	9 (3.0)	58 (5.0)
Anxiety and depression	15 (5.0)	9 (3.1)	13 (5.0)	11 (3.6)	48 (4.2)
Thyroid	31 (10.3)	24 (8.3)	16 (6.2)	21 (6.9)	92 (8.0)
Allergies	11 (3.6)	14 (4.9)	15 (5.8)	15 (4.9)	55 (4.8)
Asthma	47 (15.6)	52 (18.1)	46 (17.8)	58 (19.1)	203 (17.6)
Cholesterol	26 (8.6)	29 (10.1)	24 (9.3)	32 (10.5)	111 (9.6)
Hypertension	43 (14.2)	38 (13.2)	39 (15.1)	48 (15.8)	168 (14.6)
Menopause/perimenopause	17 (5.6)	17 (5.9)	7 (2.7)	14 (4.6)	55 (4.8)
Post-COVID syndrome	18 (6.0)	16 (5.6)	20 (7.8)	11 (3.6)	65 (5.6)
Reflux	46 (15.2)	37 (12.8)	27 (10.5)	50 (16.4)	160 (13.9)
Pain	44 (14.6)	51 (17.7)	46 (17.8)	60 (19.7)	201 (17.4)
Supplement	74 (24.5)	67 (23.3)	56 (21.7)	75 (24.7)	272 (23.6)
**Blood investigations, median (IQR)**					
Hemoglobin (g l^−1^)	142 (133, 151)	139 (131, 146)	140 (131, 150)	140 (132, 148)	140 (132, 149)
White blood cell count (10^9^ l^−1^)	6.7 (5.9, 8.0)	6.7 (5.5, 8.2)	6.8 (5.7, 7.8)	6.8 (5.6, 8.2)	6.7 (5.7, 8.1)
Platelets (10^9^ l^−1^)	267 (227, 317)	266 (232, 305)	264 (229, 304)	268 (228, 312)	267 (229, 310)
Erythrocyte sedimentation rate (mm h^−1^)	8.0 (2.0, 14.0)	6.0 (2.0, 11.0)	5.0 (2.0, 12.0)	5.0 (2.0, 11.0)	5.0 (2.0, 12.0)
Neutrophils (10^9^ l^−1^)	4.0 (3.3, 4.8)	4.0 (3.1, 5.3)	4.0 (3.1, 5.0)	3.8 (3.0, 4.8)	4.0 (3.1, 5.0)
Lymphocytes (10^9^ l^−1^)	2.0 (1.6, 2.4)	1.9 (1.5, 2.3)	1.9 (1.6, 2.4)	2.0 (1.7, 2.5)	2.0 (1.6, 2.4)
Creatinine (µmol l^−1^)	70.0 (62.0, 83.0)	71.0 (63.0, 82.5)	72.0 (63.0, 83.0)	72.0 (62.0, 84.0)	71.0 (63.0, 83.0)
Albumin (g l^−1^)	45.0 (43.0, 48.0)	46.0 (43.0, 48.0)	45.0 (41.0, 48.0)	45.0 (42.0, 47.0)	45.0 (42.0, 48.0)
Alkaline phosphatase (IU ml^−1^)	71.0 (59.0, 86.0)	71.0 (59.0, 88.0)	69.0 (57.0, 84.0)	74.0 (60.0, 86.0)	72.0 (59.0, 86.0)
Alanine amino-transferase (IU ml^−1^)	23.0 (17.0, 33.0)	22.0 (17.0, 32.0)	22.0 (16.0, 30.0)	21.0 (15.0, 29.0)	22.0 (16.0, 31.0)
Total cholesterol (mmol l^−1^)	5.2 (4.5, 5.9)	4.9 (4.2, 5.7)	4.8 (4.3, 5.6)	5.1 (4.3, 5.9)	5.0 (4.4, 5.7)
HDL cholesterol (mmol l^−1^)	1.5 (1.2, 1.9)	1.5 (1.2, 1.9)	1.5 (1.2, 1.7)	1.6 (1.3, 1.9)	1.5 (1.2, 1.8)
LDL cholesterol (mmol l^−1^)	2.8 (2.2, 3.7)	2.8 (2.2, 3.3)	2.6 (2.1, 3.1)	2.8 (2.1, 3.4)	2.8 (2.2, 3.4)
Serum ferritin (µg l^−1^)	65.0 (35.0, 137.0)	63.0 (41.0, 137.0)	70.0 (38.0, 162.0)	79.0 (41.0, 129.0)	69.0 (39.0, 141.0)
C-reactive protein (mg l^−1^)	2.4 (0.8, 5.0)	2.7 (0.9, 5.0)	2.0 (0.8, 5.0)	3.5 (1.0, 5.0)	2.5 (0.9, 5.0)
Lactate dehydrogenase (U l^−1^)	213 (184, 235)	222 (181, 247)	208 (186, 261)	210 (187, 240)	213 (184, 243)
Vitamin D (IU l^−1^)	63.0 (49.0, 82.0)	66.0 (48.0, 79.0)	59.0 (47.5, 77.0)	57.0 (43.0, 75.0)	61.0 (47.0, 79.0)
HbA1c (mmol mol^−1^)	36.0 (33.0, 39.0)	36.0 (34.0, 40.0)	36.0 (34.0, 40.0)	37.0 (34.0, 40.0)	36.0 (34.0, 40.0)
eGFR (ml min^−1^ 1.73 m^−^^2^)	90.0 (85.0, 90.0)	90.0 (80.0, 90.0)	90.0 (83.0, 90.0)	90.0 (78.0, 90.0)	90.0 (81.0, 90.0)
TSH (IU l^−1^)	1.7 (1.2, 2.2)	1.6 (1.1, 2.4)	1.6 (1.1, 2.2)	1.7 (1.2, 2.3)	1.6 (1.2, 2.3)

BMI, body mass index; eGFR, estimated glomerular filtration rate; GP, general practice; HbA1C, glycated hemoglobin; HDL, high-density lipoprotein; IMD, Index of Multiple Deprivation; LDL, low-density lipoprotein; TSH, thyroid-stimulating hormone.

The prespecified primary outcome (mean FAS at 12 weeks) is reported here. Secondary outcomes included FAS at 24 weeks (12 weeks after drug cessation) and other patient-reported outcome measures (PROMs), including the quality-of-life EQ-5D-5L visual assessment scale or EQ-VAS (by Visual Analog Scale) at 12 and 24 weeks^11^, a 100-point scale used to visually assess current health and well-being as a percentage of self-defined ‘best health’, where 0 = worst imaginable health and 100 = best health. Over 85% of patients can satisfactorily complete the EQ-VAS^27^. We only report FAS at 24 weeks, FAS at 12 weeks per protocol, and EQ-VAS at 12 and 24 weeks here. These same outcomes are presented for the drug trial in a separate publication^28^. Other secondary outcomes (Supplementary Information, pp. 37–38, 149–150, 231–244) will be analyzed and reported within 12–24 months of the end of the trial in a mental health-focused paper. Health economic/health utilization outcomes not presented here will be available in a health economic-focused paper.

#### Primary outcome

**Fig. 2:**
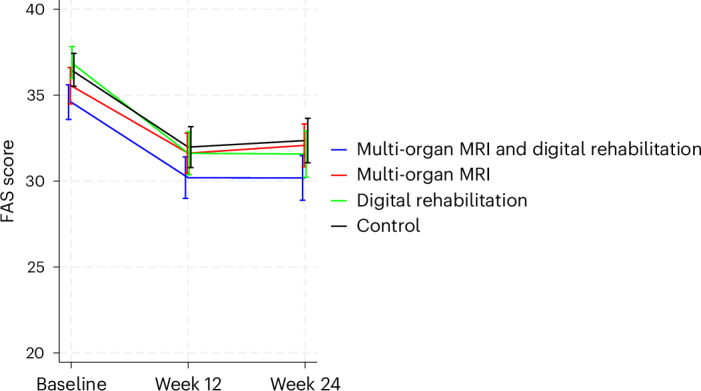
FAS over time by cluster allocation. FAS for *n* = 1,043, 914 and 792 at baseline, 12 weeks and 24 weeks, respectively. No replicates have been performed. FAS data were requested once from each participant at baseline, 12 weeks and 24 weeks. The estimates at each time point represent the means of all participants with a calculable FAS score at that time, and the bars represent the 95% CIs at that time point.

Changes in mean FAS from baseline to 12 weeks for multi-organ MRI, digital rehabilitation, both and neither arms were 3.9 (from 35.5 (s.d. 8.61) to 31.6 (8.78)), 5.3 (from 36.9 (7.25) to 31.6 (9.19), 4.4 (from 34.6 (8.57) to 30.2 (9.66)) and 4.5 (from 36.5 (8.08) to 32.0 (9.45); Table 2 and Fig. 2), respectively. Few models with random terms converged. In the mixed model for FAS at 12 weeks with PCN fitted as a random effect and site fitted as fixed, which did converge, the intra-cluster correlation coefficient for PCN was 0.01. There was sometimes disparity between the form of the model fitted to 12-week and 24-week outcomes that converged, so a linear model with fixed site effects is presented unless otherwise specified. Of 1,152 participants, 870 with baseline and 12-week FAS were included in linear regression analyses. They were 2 years older and more likely to be female (67%) and white (87%), but within 1 point of baseline FAS and EQ-VAS, compared with those excluded. After adjusting for baseline FAS, gender, PCN size and deprivation, drug trial participation and trial site, compared with usual care, estimated FAS at 12 weeks for multi-organ MRI, digital rehabilitation and their interaction differed by 0.18 (95% CI −0.72, 1.09; *P* = 0.691), −0.53 (−1.42, 0.36; *P* = 0.245) and −0.17 (−1.06, 0.72; *P* = 0.710), respectively (Table 3). These numbers represent three orthogonal effects: MRI versus no MRI, digital rehabilitation versus no digital rehabilitation and the interaction of the two other effects. The last of these shows whether the effect of the offer of digital rehabilitation on FAS differed according to whether MRI had been offered. Baseline FAS was statistically significant, that is, for every unit higher FAS was at baseline, FAS at 12 weeks was 0.80 units higher. For context, 59% of 870 participants with both baseline and 12-week FAS available experienced a clinically important drop of at least 3 points in FAS by 12 weeks (61%, 55%, 60% and 59% in both interventions, multi-organ MRI, digital rehabilitation and usual care, respectively).

**Table 2:** FAS scores across allocation groups from pre-LC, baseline, 12 weeks and 24 weeks

Mean (s.d.)	Multi-organ MRI + digital rehabilitation *n* = 302	Multi-organ MRI *n* = 288	Digital rehabilitation *n* = 258	Usual care *n* = 304	All *n* = 1,152
**FAS score—pre-COVID**	17.3 (5.85)	16.5 (5.81)	16.9 (5.70)	16.7 (6.05)	16.8 (5.86)
**FAS score—baseline**	34.6 (8.57)	35.5 (8.61)	36.9 (7.25)	36.5 (8.08)	35.8 (8.21)
**FAS score—12 weeks**	30.2 (9.67)	31.6 (8.78)	31.6 (9.19)	32.0 (9.45)	31.3 (9.31)
**Percentage change in FAS score at 12 weeks (%)**	−12.7	−11.0	−14.4	−12.3	−12.6
**FAS score—24 weeks**	30.2 (9.61)	32.1 (8.66)	31.6 (9.29)	32.4 (9.64)	31.5 (9.35)
**Percentage change in FAS score at 24 weeks (%)**	−12.7	−9.6	−14.4	−11.2	−12.0

**Table 3:** Primary outcome modeling of effect size of cluster on FAS at 12 weeks

Variable (in analysis *N* = 870)	Coefficient	95% CI	*P* value
FAS total score at baseline	0.804	(0.749, 0.859)	<0.001
Female gender at birth	0.580	(−0.370, 1.530)	0.231
PCN size 2 categories: <45,000	−0.475	(−1.439, 0.490)	0.335
IMD rank 2 categories: <14,500	0.886	(−0.031, 1.803)	0.058
Both studies	−0.714	(−1.649, 0.221)	0.134
Site (5 first): site 1	2.532	(−0.318, 5.381)	0.082
Site (5 first): site 2	−0.842	(−2.252, 0.569)	0.242
Site (5 first): site 3	−0.026	(−1.167, 1.114)	0.964
Site (5 first): site 4	−0.274	(−3.613, 3.066)	0.872
Site (5 first): site 6	−0.492	(−1.927, 0.944)	0.501
Multi-organ MRI^a^	0.092	(−0.361, 0.545)	0.691
Digital rehabilitation^a^	−0.264	(−0.709, 0.181)	0.245
Multi-organ MRI and digital rehabilitation^a^	−0.085	(−0.531, 0.362)	0.710
Constant	2.299	(0.139, 4.458)	0.037

Table 3 shows the model coefficients for a linear regression of 870 participants with FAS available at baseline and 12 weeks. Estimates for measured variables are for a one-unit change and for categorical variables for each category versus the reference category. Estimates are shown with 95% CIs and *P* values to 3 decimal places. All *P* values are two sided. No adjustments have been made for multiple comparisons. ^a^The effect sizes for the ICP components Coverscan (multi-organ MRI) and/or Living With COVID Recovery (digital rehabilitation) and their interaction are twice the model coefficients.

#### Secondary outcomes

**Extended Data Fig. 1:**
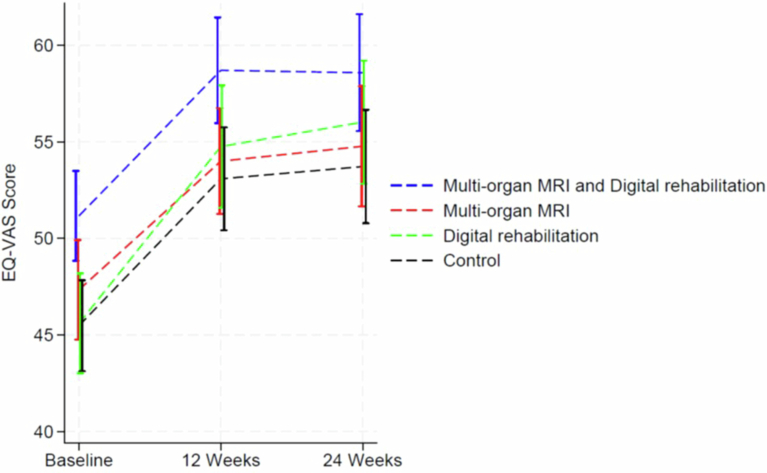
Change in EQ-VAS between recruitment and completion of follow up in participants allocated to integrated care pathway interventions. Abbreviations: EQ-5D-5L: EuroQoL-5 dimensions-5 levels; IMD: Index of Multiple Deprivation; MRI: Magnetic Resonance Imaging; VAS: Visual Assessment Scale.

FAS scores at 24 weeks compared with usual care for multi-organ MRI, digital rehabilitation and their interaction were 0.43 (−0.57, 1.43; *P* = 0.398), −1.14 (−2.12, −0.16; *P* = 0.023) and −0.23 (−1.21, 0.76; *P* = 0.651; Table 4). Quality of life showed no significant differences across ICP arms from usual care (Extended Data Table 1). However, EQ-VAS at 24 weeks improved with digital rehabilitation. EQ-VAS at 24 weeks was higher by −0.96 (−3.70, 1.78: *P* = 0.492), 2.74 (0.07, 5.41; *P* = 0.045) and −0.15 (−2.84, 2.55; *P* = 0.915) units for participants in multi-organ MRI, digital rehabilitation and for their interaction, respectively (Extended Data Table 2 and Extended Data Fig. 1).

**Table 4:** Secondary outcome modeling of effect sizes of ICP intervention on change in FAS scores from baseline to week 24

Variable	Coefficient	95% CI	*P* value
FAS total score at baseline	0.787	(0.726, 0.848)	<0.001
Participant gender at birth: female	0.286	(−0.767, 1.338)	0.594
PCN size 2 categories: <45,000	−0.803	(−1.857, 0.250)	0.135
IMD rank 2 categories: <14,500	0.685	(−0.323, 1.694)	0.182
Both studies	0.506	(−0.524, 1.536)	0.335
Site (5 first): site 1	0.941	(−2.133, 4.015)	0.548
Site (5 first): site 2	0.199	(−1.347, 1.746)	0.800
Site (5 first): site 3	−0.620	(−1.851, 0.612)	0.323
Site (5 first): site 4	1.291	(−3.500, 6.083)	0.597
Site (5 first): site 6	−0.805	(−2.397, 0.787)	0.321
Coverscan -1_1^a^	0.215	(−0.285, 0.715)	0.398
App -1_1^a^	−0.569	(−1.059, −0.079)	0.023
ScanApp -1_1^a^	−0.114	(−0.606, 0.379)	0.651
Constant	3.109	(0.688, 5.529)	0.012

^a^Note the treatment effects and CIs for Coverscan, digital rehabilitation and their interaction are double the values in the table.

#### Safety

The combined trials had 11 serious adverse events (SAEs) for 9 participants. In the ICP trial, there were 4 SAEs for 4 participants, 3 of whom were in the drug trial, and were allocated to Coverscan (with colchicine), digital rehabilitation (with famotidine and loratadine), usual care (no drug) and digital rehabilitation (not in drug trial). No SAEs were related to drug or ICP trial interventions (Extended Data Table 3).

### Sensitivity analyses

The no drugs intention-to-treat ITT analysis (*N* = 626 and 547 at 12 and 24 weeks) showed similar results: small nonsignificant fatigue reductions with multi-organ MRI and digital rehabilitation. In per-protocol analyses, neither intervention had significant effects at 12 weeks, with effects similar in size and direction to the full ICP trial at 24 weeks. Differences from usual care in FAS at 24 weeks were 1.22 (95% CI 0.02, 2.43), −1.75 (95% CI −2.95, −0.54) and 0.53 (95% CI −0.65, 1.71) for multi-organ MRI, digital rehabilitation and their interaction, respectively. Treatment effects were similar in per-protocol versus ITT analyses. Of the 437 participants who received multi-organ MRI, 105 (24%) had site-reported abnormal findings.

### Post hoc analyses

Those with an abnormal Coverscan had lower FAS scores by 0.5, 0.4 and 1.0 at baseline, 12 weeks and 24 weeks, than those with normal Coverscan, respectively. Regression analyses restricted to those with a Coverscan showed almost no difference in FAS at 12 and 24 weeks between those with site-reported abnormal and normal scans, although power was very low for this comparison.

Those who were registered with the digital rehabilitation and had used it at least once were analyzed considering their FAS scores at 12 weeks and at 24 weeks against counts of engagements with the digital application by that time; no significant associations were seen per additional engagement. Similarly, having more than 50 engagements at 12 or 24 weeks appeared to offer no significant benefit in FAS at either time point.

## Discussion

In the largest interventional LC trial to date, we report an overall decrease in reported fatigue with specialist LC care. Additional diagnostic information from multi-organ MRI did not impact fatigue or quality of life at 12 or 24 weeks. There was no additional improvement in fatigue with digital rehabilitation versus usual care at 12 weeks, but a small subsequent improvement in the secondary outcome of fatigue and quality of life at 24 weeks was seen, which is of uncertain clinical significance. We show the importance of holistic clinical LC care, and the feasibility of conducting pragmatic trials to evaluate ICP components in complex post-viral conditions during pandemics.

Our study has multiple strengths. Diagnosis of LC was validated by a specialist clinician in a specialist LC clinic with an accepted LC definition, reducing heterogeneity and increasing the validity of findings. Our interventions were already being used in clinical practice, and the trial was pragmatically designed to reflect routine care and a representative, patient population with clinician-adjudicated diagnosis of LC. The primary outcome was the commonest reported LC symptom and is a common endpoint of many LC symptoms. Robust primary and secondary outcome measures were selected. Cluster randomization allowed routine care deployment of ICP interventions, increasing validity and real-world application of findings. We tested interventions (diagnostic and rehabilitation) within services commissioned according to a shared NHS ICP model. Therefore, despite heterogeneity in LC definitions and care, we standardized delivery of ICP components as much as possible.

However, there were several limitations. Although we had adequate power to report the primary outcome in all arms of the trial, we could not control for interactions between drug allocation and ICP components for the minority of participants co-enrolled in the nested drug trial due to lower-than-expected recruitment at ICP trial sites. We designed the trial in 2021, without fully anticipating changes in viral variants, repeat infections, vaccine updates and deployment, national policy, clinical service landscape and resource constraints during the recruitment period. Resulting variability in intervention delivery across sites may have impacted findings. Full evaluation of all ICP components tested requires analyses incorporating clinical and health economic data, which we will complete over the next year. During the trial, we collected and collated data, including imaging findings, rehabilitation engagement and healthcare utilization and cost, enabling ongoing and future detailed analysis that will benefit the clinical and academic LC community and ultimately further inform implementation and acceptability of ICP interventions for LC.

Overall, we observed a 12.6% FAS reduction from a high baseline. Although a minimal clinically important difference for FAS is not confirmed in LC, this change is commensurate with clinically meaningful reductions in respiratory conditions^29^. Participants reported long symptom duration and severe fatigue at baseline with consistent 12-week reduction across sites, but not 12–24 weeks with usual care, arguing against spontaneous recovery alone. Although not designed to test the NHS ICP’s efficacy, we suggest its direct impact. This care is commissioned for the first 12 weeks after referral in England, making a non-ICP control arm impractical and unethical. Further research will determine the efficacy of individual ICP components within this standard model.

Multi-organ imaging could identify aging and chronic diseases early in trajectories of several LTCs^30^. We hypothesized that multi-organ impairment in LC, well-described on MRI^13,14^, could guide timely treatment if identified earlier in the disease process. Access to pre-clinic, multi-organ MRI did not incrementally improve fatigue, suggesting no significant benefit in the context of the time window between the scan and clinic review, treatments currently available or within the trial timescale. Current clinical guidance recommends use of investigations to exclude other causes of symptoms, but clinical LC diagnosis is based on symptoms with symptom-based, largely nonpharmacological management. Therefore, Coverscan abnormalities do not currently open different therapeutic options, which may change with increased understanding of LC^31^. Specific imaging abnormalities, compared with prior findings in LC^13^ and how multi-organ MRI affected later tests and healthcare use and costs are important to contextualize findings and will be reported subsequently. Our results do not support routine use of comprehensive multi-organ imaging in LC at present.

Digital rehabilitation has improved PROMS in LC and other conditions in smaller trials^32–35^. Evidence for rehabilitation, including digital rehabilitation, in improving symptoms in LC is at least equivalent to the weak evidence base for drugs^36^, despite heterogeneity in definition, design, implementation and evaluation of digital rehabilitation^16,37^. We report that use of the Living With COVID Recovery App was associated with some improvement in fatigue and quality of life at 24 weeks, rather than 12 weeks. This observed difference between treatment groups of 1.14 points on FAS was statistically significant but may not be clinically meaningful. This difference between a treatment group and usual care of <1.5 is small on a 40-point FAS scale and, moreover, is a secondary outcome, which should be viewed as hypothesis-generating rather than definitive. The LC clinic model typically involves focused multidisciplinary input in the first 12 weeks after first assessment, when digital rehabilitation may have provided overlapping benefit. Digital rehabilitation may play a stronger role in the absence of usual care or during longer follow-up where reliance on self-management increases.

Even with a national LC clinic program, there has been substantial variability in care^38^. Both drugs and ICP components need to be tested at scale to improve care and reduce variability, and also to optimize use of limited staffing and resources. There is an increasing evidence base for the benefit of ICP approaches and more detailed analysis of which components work in design and implementation^1,3,39^. The COVID-19 pandemic raised interest in platform trials and electronic health record trials, but mostly for drugs^40^. There is perhaps an even more pressing need to use similar methods to test components of ICPs^41^.

Our findings have implications for research, practice and public health. We combined quantitative and qualitative service evaluation, demonstrating this approach is feasible, with cross-disciplinary relevance to ICPs for other post-viral conditions and LTCs. In practice, rapid evaluation of ICP components is important to improve care at scale in quickly evolving diseases and services. A coordinated approach to ICPs across LC and other LTCs is needed.

Overall, our findings support investment in accessible, specialist care for LC, and further assessment of the efficacy of digital rehabilitation. Future trials of ICP interventions of different designs in different settings are urgently required to determine optimal ICPs for LC.

## Methods

### Trial design and oversight

The STIMULATE-ICP trial aimed to investigate the efficacy of an enhanced ICP for LC, compared with usual care^15^ (Supplementary Information, pp. 1–121). The interventions were pre-hospital, community-based, multi-organ MRI (Coverscan and clinical decision support^13,42,43^) and community-based, digital rehabilitation (Living with COVID Recovery)^16,25^. These interventions were already fully developed, licensed for use and being used in some centers in routine clinical practice to enable pragmatic evaluation, which would directly inform care of individuals with LC^13,16,25,42,43^. Usual care was NHS guideline-based specialist LC care, including clinical assessment and investigations, self-management, multidisciplinary rehabilitation for support with symptoms and pacing and ongoing community support^44–46^. After assessment in one of six NHS LC clinics and formal diagnosis of LC, drug trial participation was also offered. Here, we report ICP trial results. Trial Steering and Independent Data Monitoring Committees provided oversight of the trial, and safety and data monitoring, respectively. We vouch for data completeness and adherence to the protocol and statistical analysis plan (SAP).

### Patient and public involvement

A patient and public involvement panel (*n* = 11) contributed to all aspects of the study, from funding application and design to publication and dissemination. For example, patient and public involvement representatives were part of the trial steering committee, study management and co-authorship for publications.

### Participants

Participants were eligible with the following inclusion criteria:

1. Capable of giving informed consent.
2. Age 18 years and above.
3. Persistent signs and symptoms for a period of 4 weeks or longer in duration after COVID-19 infection (either by test result or symptomology).
4. Able to read or understand English or have a relative/family member able to read/understand English to facilitate participation (essential for PROMs at follow-up time points and virtual contact).
5. Not enrolled in any other interventional study where study intervention/activities may affect outcome measures (patients enrolled in purely observational studies could be included).
6. Presenting at their first referral to a participating LC clinic pathway.

Prior COVID-19 hospitalization was an exclusion, to focus on nonhospitalized individuals (that is, those individuals who were not hospitalized with their initial COVID-19 illness) with higher burden and healthcare utilization^44,45^.

### Interventions and randomization

The rationale for cluster randomization is published elsewhere^15,47^. Randomization used data available for each PCN, the sum of list sizes of linked GPs (larger ≥ 45,000, smaller < 45,000) and median of the rank of deprivation indices of linked GP postcodes (less deprived ≥ 14,500, more deprived < 14,500), ensuring equity of access. PCN-level clustering allowed Coverscan and/or Living With COVID Recovery as an uplift to usual care in allocated PCNs, becoming an ICP within that PCN without the need for individual consent. Research access to and collection of Coverscan and Living With COVID Recovery data were by individual consent on study entry (by research staff). Cluster randomization (2 × 2 factorial design) provided four groups:

- Coverscan+Usual Care Self-Management Rehabilitation (‘multi-organ MRI’).
- No Coverscan+Living With COVID Recovery App (‘digital rehabilitation’).
- Coverscan+Living With COVID Recovery App (‘both’).
- No Coverscan+Usual Care Self-Management Rehabilitation (‘neither’).

Like other large, pragmatic COVID-19 era trials^48^, investigators and participants were unblinded. Blinding of analysts was impractical given original plans for interim analyses. Participants could refuse uplift of care, by rejecting the offer of Coverscan or refusing to register with, or engage with, the Living With COVID Recovery App.

### Outcomes

The primary outcome was fatigue at 12 weeks by mean FAS (10–50 scale, 50 is most severe fatigue), a well-validated PROM across diseases^47,48^. Secondary outcomes included FAS at 24 weeks (12 weeks after drug cessation for those in the drug trial), FAS at 12 weeks per protocol, and PROMs, including quality of life (by EQ-VAS: 0–100 scale, 100 is the best imaginable health state) at 12 and 24 weeks^11,29,49,50^. If one of ten FAS items was missing, it was replaced by the average of the other nine items (after reversing two specific items), to allow FAS score to be calculated. Adverse events were not routinely recorded for those not also in the drug trial. SAEs were reported. Note that in the protocol on the ISRCTN, the FAS scores at baseline, 12 weeks and 24 weeks were incorrectly listed as the trial primary outcomes, rather than FAS at 12 weeks, which is the correct primary outcome. This oversight has now been addressed and the ISRCTN website updated accordingly. In the published protocol, the primary outcome was correctly recorded as 12-week FAS.

### Procedures

Participants were referred to each site’s trial team, screened by eligibility criteria and given trial information. After informed consent and random treatment allocation, standard case report form data were collected, for example, new diagnoses and prescriptions (baseline case report form). Information on sex, gender and race/ethnicity was collected (for example, self-reported by a questionnaire). The trial team conducted 12-week and 24-week visits (in-person or telephone).

There were five amendments to the original protocol. The first (issued 14 January 2022) incorporated only famotidine/loratidine combination in response to a Medicines and Healthcare products Regulatory Agency and Health Research Authority combined review. The second (issued 07 March 2022) was for inclusion of colchicine and rivaroxaban and associated changes in analyses. The third (issued 12 December 2022) was uncoupling of the ICP trial from the drug trial by adding drug arm-only sites for clinics unable to access Coverscan or the Living with COVID Recovery App due to challenges in drug trial recruitment. The fourth (issued 09 June 2023) included: (i) inclusion of individuals with ongoing LC symptoms seen before the study commencement into the drug study; (ii) update as per urgent safety measure for famotidine (Supplementary Information, pp. 205). The fifth amendment (issued 19 Dec 2024) removed: (i) characterization of pathophysiology, trajectory, healthcare utilization and outcomes of LC from secondary objectives and changed to exploratory objectives. Full details of amendments are provided in Supplementary Information (pp. 128–135).

### Statistical analysis

Sample size was calculated using PASS v21.0.1 (2021) for a 2 × 2 cluster factorial trial, powered to detect an interaction on the FAS scale between Coverscan and Living With COVID Recovery rather than main effects. Based on published data, a FAS standard deviation for individuals with LC was estimated at six units. The difference in mean FAS between people who had or had not recovered from LC was 9 points in a 2021 study^49^. An interaction effect of 3 points in FAS could be detected with >90% power and (two-sided) significance level of 0.05 with 960 participants in 48 PCN clusters of 20 participants, assuming a conservative intra-cluster correlation coefficient of 0.02. Assuming a conservative 15% dropout and equal missingness across arms, sample size was inflated by a factor of 100/85 to 1,130 (ref. ^15^).

Multilevel model analysis was used to evaluate the effects of Coverscan, digital rehabilitation and their interaction on FAS at 12 weeks, adjusting for baseline FAS, with gender, binary PCN size and binary PCN deprivation, and a binary indicator of participation also in the drug trial, as fixed effects and clinic site and PCN as random effects. If the variance estimate for a clinic site, fitted as a random effect in a multilevel model, appeared unstable (very close to zero, or confidence interval could not be estimated), it was fitted instead as a fixed effect relative to the largest clinic (site 5). If the variance estimate for a PCN fitted as a random effect also appeared unstable, a linear regression model was used instead. In this model, clinic was fitted as a fixed effect, and PCN was represented only by PCN size and deprivation. Each of the clustered elements (Coverscan, digital rehabilitation and their interaction) was coded as −1 and 1 so that the three terms were orthogonal and each comparison was two groups versus two groups, improving precision of the interaction term. The consequence of this is that the model coefficient for the cluster terms must be doubled to estimate treatment difference relative to usual care. All *P* values are two sided and shown without adjustment for multiple testing. All analyses used all available outcomes. The same model structure was applied to secondary outcomes.

Further details are provided in the SAP (Supplementary Information, pp. 122–204). Analyses were conducted using statistical package Stata version 17. The trial database was held by University College London. University of Lancashire Clinical Trials Unit led trial data collection and analysis. For the primary outcome, three ICP trial analysis populations were considered: all (ITT), those not allocated to active drug in the drug trial (no drugs ITT) and those receiving full allocation of uplifted care (per-protocol). CONSORT 2025 guidelines were followed, as well as the CONSORT cluster-randomized trial extension^51,52^ (Supplementary Information, pp. 249–255).

The treatment policy approach research question aimed to assess the effect of offering Coverscan, digitally enabled community rehabilitation and their interaction, in comparison to usual care, on FAS at 12 weeks. This was assessed in everyone assigned a pathway in the cluster-randomized trial (analysis population 1), who provided a calculable primary outcome at 12 weeks and at baseline, regardless of receipt of the allocated pathway. A multilevel model analysis was used to evaluate effects of Coverscan, digitally enabled community rehabilitation and the interaction of Coverscan, digitally enabled community rehabilitation, on FAS at 12 weeks adjusting for baseline (post-COVID) FAS, with gender as a fixed effect and clinic and PCN as random effects (random intercepts). To account for any systematic effects of participating in both the cluster and drug trial, an indicator term was added to the model. The strata variables binary IMD (from median IMD of the PCN GP practice postcodes) and binary PCN patient population size (from the sum of the PCN GP practice lists) were fitted as fixed effects.

The modeling coefficients for the ICP elements represent three orthogonal effects: MRI versus no MRI, digital rehabilitation versus no digital rehabilitation and the interaction of the two other effects. The interaction term being significant would suggest that any improvement in FAS from the offer of digital rehabilitation is similar, whether or not MRI is offered. As the three modeling intervention terms were orthogonal and parameterized (1, −1) rather than (0, 1), the model coefficients for these terms were doubled to get the expected effects on FAS of being offered one of the interventions given in the text. The assumed covariance structure of the random effects terms was independence.

When there were difficulties with convergence, the first step was to model site as fixed (relative to the largest site). The second step was to drop PCN as a random effect and to fit a linear regression instead with site fitted as fixed. Where the models for the 12-week and 24-week time points had different forms due to convergence problems, the default was to choose the simplest form of the model to present, usually a linear regression model with site fixed and PCN dropped as a random effect. Residuals of all models were assessed for normality graphically using a histogram. The analyses presented used all available outcome data (ITT). Missing data approaches are described in the SAP (Supplementary Information, pp. 178–179).

For the primary outcome, two further analysis populations were considered: (1) all participants in the cluster trial excluding those taking the active drugs (population 2) and (2) those in the cluster trial receiving their allocated ICP intervention (population 3). The analysis comparing the primary outcome between scanned participants with normal and abnormal scans was limited to two treatment arms. The analysis modeling the primary outcome within digital rehabilitation participants considering their engagement was limited to two treatment arms and those where summary data on engagement could be matched.

### Ethics statement

The NHS Health Research Authority (IRAS 1004698) Research and Ethics Committee South Central- Berkshire (21/SC/0416) and Medicines Health Regulatory Agency approved the trial. The trial was registered under ISRCTN identifier ISRCTN10665760. After receiving full study information and sufficient time, informed consent was taken by suitably qualified, trained and experienced trial staff for all participants.

### Reporting summary

Further information on research design is available in the Nature Portfolio Reporting Summary linked to this article.

## Online content

Any methods, additional references, Nature Portfolio reporting summaries, source data, extended data, supplementary information, acknowledgements, peer review information; details of author contributions and competing interests; and statements of data and code availability are available at 10.1038/s41591-026-04552-x.

## Supplementary information

Supplementary Information Trial Protocol 1–121; SAP 122–204; Urgent Safety Measure 205; Patient Baseline Questionnaire 206–248; CONSORT checklist 249–255.

Reporting Summary

Peer Review File

## Figures and Tables

**Extended Data Table 1:**
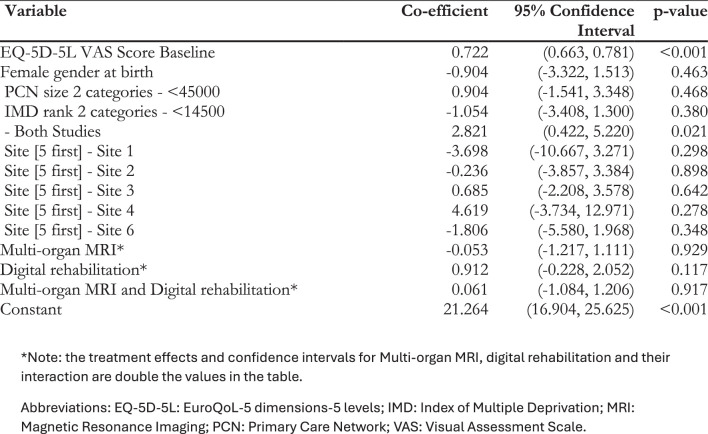
Secondary outcome modelling of effect size of integrated care pathway intervention on change in EQ-VAS from baseline to 12 weeks

*Note: the treatment effects and confidence intervals for Multi-organ MRI, digital rehabilitation and their interaction are double the values in the table.

**Extended Data Table 2:**
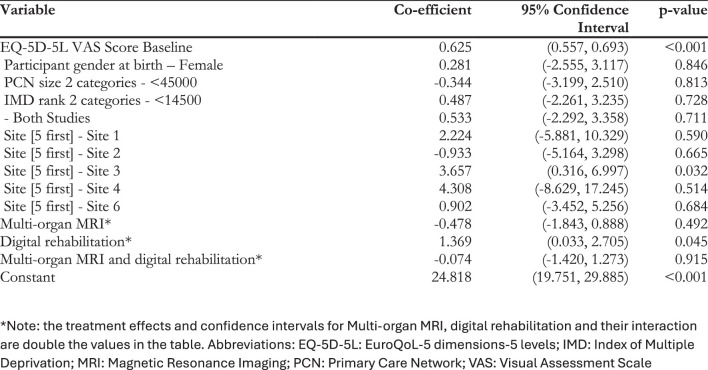
Secondary outcome modelling of effect size of integrated care pathway intervention on change in EQ-VAS from baseline to 24 weeks

*Note: the treatment effects and confidence intervals for Multi-organ MRI, digital rehabilitation and their interaction are double the values in the table.

## Data Availability

Anonymized data (and supporting materials) are held at UCL in line with ethical approval and consent of participants. Access to the minimum dataset will be available by application to the corresponding author A.B., which will be reviewed and approved by the trial steering committee, aiming for a response within 2 months. These data will be available within 12 months of completion of the study (by December 2026) and will include individual-level participant data.

## Extended data

**Extended Data Table 3:** Serious adverse events and adverse events

Participant	ICP trial/Drug trial	Severity	Outcome	Required	Event	Related/Not Related
A	Digital rehabilitation/Not in drug trial	Life-threatening	Recovered	Care of mental health team	Overdose of antidepressant drug	Not Related (No Reasonable Possibility)
B	Usual Care/Usual Care (no drug)	Severe	Recovered	Hospitalisation	Community acquired pneumonia	Not Related (No Reasonable Possibility)
C	Multi-organ MRI/ Colchicine	Severe	Recovered with Sequelae	Hospitalisation	Left posterior cerebral artery infarct. Colchicine not being taken at time of SAE	Not Related (No Reasonable Possibility)
D	Digital rehabilitation/ Famotidine + Loratadine	Moderate	Recovered	Hospitalisation	Pneumonitis after treated pneumonia	Not Related (No Reasonable Possibility)

C: Colchicine; F+L: Famotidine/loratadine; IMP: Investigational Medical Product; R: Rivaroxaban; UC: Usual Care.

**Extended Data Table 4:** Details of the STIMULATE-ICP consortium

Committee	Section	Members
*Steering committee*		
	Independent members	Patrick Mallon (Chair; University College Dublin); Ondine Sherwood (Long COVID SOS; deceased 2025); Matthew Sydes (NHS England); Marion Mafham (University of Oxford).
	Sponsor	Michelle Quaye (UCL/UCLH Joint Research Office)
	Study team	Amitava Banerjee (CI; University College London); Melissa Heightman (co-CI), UCLH NHS Trust; Gordon Prescott (Lead trial statistician; University of Lancashire); Matthew Burns (Trial management; University of Lancashire), Hakim-Moulay Dehbi (Trial statistician; University College London); Caroline Watkins (Trial management; University of Lancashire)
*Data Monitoring Committee*	Independent members	Peter Langhorne (Chair: University of Glasgow); Tim Peters (University of Bristol); Ly-Mee Yu (University of Oxford)
	Study team	Amitava Banerjee (CI; University College London); Gordon Prescott (Lead trial statistician; University of Lancashire); Matthew Burns (Trial management; University of Lancashire
