# Supplementary material for: Integrated care pathway in individuals with Long COVID: STIMULATE-ICP, a cluster-randomized, phase 3 trial
Source: Nat Med. 2026 Jul 28;32(8):3040–7. doi: 10.1038/s41591-026-04552-x (PMC13472957; doi:10.1038/s41591-026-04552-x)
Supplement: Supplementary file 1 [file 41591_2026_4552_MOESM1_ESM.pdf]

nature medicine

Supplementary information
https://doi.org/10.1038/s41591-026-04552-x

Integrated care pathway in individuals with Long COVID: STIMULATE-ICP, a cluster-randomized, phase 3 trial

In the format provided by the authors and unedited

Docusign Envelope ID: 3B3A8C9B-95D4-4BCB-A627-C64CC90D1747

A pragmatic, multi-centre, cluster randomised trial of an integrated care pathway with a nested, Phase III, open label, adaptive platform randomised drug trial in individuals with Long COVID

STIMULATE-ICP

PROTOCOL VERSION: 5.1

This protocol has regard for HRA guidance and order of content.

RESEARCH REFERENCE NUMBERS

**IRAS Number:**	1004698
**EudraCT Number:**	2021-006598-47
**SPONSOR:**	University College London (UCL)
**SPONSOR Number:**	143602
**REC Reference Number:**	21/SC/0416
**FUNDERS:**	National Institute for Health Research<br>Perspectum
**FUNDERS Number:**	29622 NIHR COV-LT2-0043

CONFIDENTIAL

SIGNATURE PAGE

The undersigned confirm that the following protocol has been agreed and accepted and that the Chief Investigator agrees to conduct the trial in compliance with the approved protocol and will adhere to the principles outlined in the Medicines for Human Use (Clinical Trials) Regulations 2004 (SI 2004/1031), amended regulations (SI 2006/1928) and any subsequent amendments of the clinical trial regulations, the UK Policy Framework for Health and Social Care Research 3rd edition 2017 (as amended), GCP guidelines, the UK Data Protection Act (2018), the Sponsor's (and any other relevant) SOPs, and other regulatory requirements as amended.

I agree to ensure that the confidential information contained in this document will not be used for any other purpose other than the evaluation or conduct of the clinical investigation without the prior written consent of the Sponsor.

I also confirm that I will make the findings of the trial publicly available through publication or other dissemination tools without any unnecessary delay and that an honest accurate and transparent account of the trial will be given; and that any discrepancies and serious breaches of GCP from the trial as planned in this protocol will be explained.

CONFIDENTIAL

Chief Investigator:

Signature and Date: Amitava Banerjee 20-08-2025

Name (please print): Amitava Banerjee

Professor Amitava Banerjee

For and on behalf of the Trial Sponsor:

Signature and Date: Michelle Quaye 19-08-2025

Name (please print): Michelle Quaye

Position: Regulatory Manager, Advanced Therapy Trials

PROTOCOL VERSION HISTORY

Version Number	Date	Protocol Update Finalised By (insert name of person)	Reasons for Update
1.0	10.12.2022	Denise Forshaw	Submitted to MHRA and HRA combined review for approval
1.1	04.01.2022	Denise Forshaw	Updated in response to MHRA and HRA combined review letter
2.0	07.03.2022	Denise Forshaw	Updated for inclusion of Colchicine and Rivaroxaban to the randomisation arms of the trial and associated changes in statistical analysis, optional blood sample collections
2.1	29.04.2022	Denise Forshaw	Updated contents page numbering and the creatinine clearance rate had been changed from <30 mL/min to <15 mL/min to reflect the introduction of the Rivaroxaban and Colchicine arms
3.0	12.12.2022	Denise Forshaw	1. Addition of drug arm only sites for clinics unable to access MRI scanning and unable to also access Living with COVID Recovery™ App.<br>Inclusion of randomisation and statistical details for drug only sites.<br><br>2. Addition of option for patients to receive the baseline questionnaire in advance of clinic/enrolment and to bring it to their appointment.<br><br>3. Scan to be provided as an uplift to usual care for all non-hospitalised long COVID patients only<br><br>4. Addition to exclusion. Participants with known Blood Borne Viruses or conditions severely affecting clotting will not be eligible for gifting blood as per the sample manual.<br><br>5. Advice included as to blood test results for liver function and coagulation (previously approved by MHRA as file note subject to inclusion in next amendment submission) now added formally to protocol.<br><br>6. Extension of time frame for gifting blood samples for patients consented to data collection and bloods only.<br><br>7. Data collection time frame corrected from +7 days post due date to +/- 7 days to increase flexibility for patient preference and clinic capacity.<br><br>8. Addition of witnessed consent form for a. participants with visual and/or functional impairment; that prevents valid wet ink signature. Participants with good verbal English skill but limited reading/writing English skills.

CONFIDENTIAL

			9. The description of Coverscan has been amended for clarity detailing cross sectional images taken for function assessment in the lungs not complete lung scan.<br>10. Addition of 7-day washout period post last dose of IMP before resuming contraindicated or same class drugs.<br><br>11. Addition of detail of direct to patient IMP delivery service (currently one site only).<br><br>12. Date of randomisation clarified as date used for timing of follow-up points for all patients in the drug study. Date on enrolment for all other participants.<br><br>13. Patients gifting blood samples will be able to have these destroyed if requested following withdrawal from the trial.<br><br>14. Blood dyscrasias added as an exclusion criterion for drug trial is per MHRA requirement following Substantial Amendment 1.<br><br>15. Addition of wording to clarify SSRIs and NSRIs are to be used with caution in participants receiving rivaroxaban as recommended in the SmPC and requested by MHRA in response to Substantial Amendment 1.<br><br>16. Addition of wording to clarify randomisation may only occur in some participants as a result of eligibility testing post consent (tests for research purposes only requiring consent)
4.0	09.06.2023	Denise Forshaw	1. Inclusion of patients (with ongoing long COVID symptoms) seen prior to the study commencement into the drug study<br><br>2. Revision of statistical sections so that the principal analysis population for the cluster trial is changed to "those who consented to data collection for the cluster RCT regardless of their participation in the drug trial or their drug trial treatment allocation"<br><br>3. Revision of statistical sections so that principal analysis population for drug vs. usual care comparisons within the drugs trial will to "anyone consenting to the drug trial including participants also cluster randomised to any treatment pathway in the cluster trial, participants from cluster trial sites who have previously been seen at a clinic, and participants randomised into the drug trial from clinics that can only offer the control treatment pathway including both new clinic patients and those who have previously been seen at a clinic".

CONFIDENTIAL

			4. The p-value for each of 5 interim analyses was due to be reset from p=0.005 to the more conservative p=0.001 on advice of the IDMC. The interim analysis plan has been rewritten and as the number of interim analyses is reduced from 5 to 1 the p-value for the interim analyses, is instead changed to p=0.005.5. Duration of trial amended to 30 months from 24 months to reflect approval of 6-month non-costed extension<br><br>6. Correction eGFR to (eGFR <30 mL/min) in eligibility criteria
4.1	31.10.2023	Denise Forshaw	SmPC update issued for Famotidine to exclude Posaconazole, Dasatinib, Pazopanib, Erlotinib and Gefitinib for concomitant use. (N.B., Posaconazole is already excluded for use with Colchicine, Dasatinib and Pazopanib were already excluded for concomitant use with Rivaroxaban and listed in section 6.8, but Erlotinib and Gefitinib were not previous exclusions for any of the selected IMP`s.) Requiring update to:<br><br>1. Inclusion & Exclusion criteria (page 19)<br><br>2. Section 6.8: Drug Interactions and Specific Contraindications.<br>Table 3: List of Specific Drug Interactions & Contraindications/Exclusions with trial IMPs (page 45)<br><br>3. Section 7.2 Participant Eligibility Criteria (page 51)
5.0	19.12.2024	Gordon Prescott	1. Updates to section 4.2 of the protocol, to change characterisation of the pathophysiology and trajectory of Long COVID from a secondary trial objective to an exploratory objective.<br>2. Updates to section 4.4 and 4.5 of the protocol to move secondary outcome measures (blood investigations (e.g., genomic, proteomic, metabolomic/ lipidomic, functional T cell and live virus neutralisation assays and endocrine analyses) and linked electronic health record data to monitor healthcare utilisation and outcomes) to exploratory outcome measures. Further detail on this outcome measure has also been added to section 4.5 of the protocol.<br>3. Additional information added to section 9.5.2 and 9.5.2.2 regarding the samples for sub-study analysis at central laboratories. Clarifying work being undertaken as part of this trial and

CONFIDENTIAL

			analysis which will be part of future research.<br>4. Additional information added regarding data transfer between LCTU and Perspectum, Living with COVID Recovery™, and the Crick Institute in section 13.5 of the protocol.<br>5. Addition of a new section describing the exploratory analysis for the study (section 14.11).<br>6. Removal of interim analysis following oversight committee consultation. The protocol stated that an interim analysis would be performed after the recruitment of 600 participants into the drug trial. This occurred much closer to the end of the recruitment period than anticipated. It was therefore agreed that the interim analysis served no purpose, as even if performed immediately after the 600th patient was randomised (occurred in May 2024), there would be no opportunity to change anything about the treatments offered in the trial before the end of recruitment (31st July 2024). This results in updates to sections 5.3.1, 8.4, 14.2 14.10 and 17.3. This is supported by the Trial Steering Committee chair.<br>7. Small changes in the adjustment factors for the drug analysis in section 14.7. Participants who join the drug trial directly will not have been cluster randomised by PCN. Therefore the adjustment for all participants in the drug trial needs to reflect the individual randomisation by gender, site and relevant pathway (cluster treatment pathway and drug only new or previously seen participant).<br>8. Updates to the Lancashire CTU contact information and IDMEC membership<br>9. Widening of week 24 data collection window to -7 days/+ 6 weeks to allow a greater amount of time for completion of questionnaires by participants<br>10. Minor corrections and clarifications
5.1	18.08.2025	Gordon Prescott	Removed details pertaining to use of NHS data regarding medical history from January 2020 and up to 1 year following enrolment in the study via NHS digital. This data will no longer be collected as part of the trial.

CONFIDENTIAL

			1. Estimated trial duration per participant updated to remove data collection will continue 12 months from enrolment (page 17).<br>2. Section 13.3 Linked National Data Resource (healthcare systems data) removed (page 89).<br>3. Section 14.8 Secondary Outcome Analysis, sentence referring to data taken from medical records up to 12 months from enrolment removed (page 94).<br>4. Section 15. End of Trial, 12-months following enrolment details removed (page 96).

CONFIDENTIAL

KEY TRIAL CONTACTS

**Chief Investigator**	Professor Amitava Banerjee<br>Institute of Health Informatics<br>University College London<br>222 Euston Road<br>London<br>NW1 2DA<br>Email: ami.banerjee@ucl.ac.uk
**Sponsor**	Michelle Quaye<br>Joint Research Office, UCL, 4th Floor West,<br>250 Euston Road<br>London NW1 2PG Postal address: Joint Research Office, Gower Street, London WC1E 6BT<br>Email: CTIMPs@UCL.ac.uk
**Clinical Trials Unit**	Prof Dame Caroline Watkins, Director Lancashire Clinical Trials Unit, University of Central Lancashire, Preston PR1 2HE<br>Email: CLWatkins@uclan.ac.uk
**Statisticians**	Dr Gordon Prescott<br>Deputy Director of Lancashire Clinical Trials Unit, The University of Central Lancashire, Preston PR1 2HE<br>Email: GPrescott1@uclan.ac.uk<br><br>Dr Hakim-Moulay Dehbi<br>University College London, Comprehensive Clinical Trials Unit, London.<br>Email: h.dehbi@ucl.ac.uk
**Principal Clinical Trials Manager**<br><br><br>**Trial Management Team**	Matthew Burns, Lancashire Clinical Trials Unit, University of Central Lancashire, Preston PR1 2HE<br>Email: MRBurns@uclan.ac.uk<br><br>Trial Management Team<br>Lancashire Clinical Trials Unit, The University of Central Lancashire, Preston PR1 2HE<br>Email: LCTUStimulate@uclan.ac.uk
**Central Laboratories**	Oxford Community Diagnostic Laboratory (Perspectum)<br>c/o Sarah.larkin@perspectum.com<br>Perspectum,<br>Gemini One, 5520 John Smith Drive,<br>Oxford, OX4 2LL,<br>United Kingdom<br>Office: +44 (0) 1865 655 343<br>perspectum.com<br><br>Science Technology Platform,<br>High-Throughput Screening Laboratory

CONFIDENTIAL

	C/O Mike Howell (Michael.Howell@crick.ac.uk)<br>Francis Crick Institute<br>1 midland Road, London NW1 1AT<br>Mike Howell
**Central Pharmacy / IMP**	Study drugs to be issued by prescription from participating site pharmacy or clinic setting. An individualised plan will be put in place for each setting.
**Collaborators / Co-Investigators**	Professor Christina Van der Feltz-Cornelis<br>Professor Dame Caroline Watkins<br>Professor Dan Cuthbertson<br>Professor Elizabeth Murray<br>Professor Gregory Lip<br>Professor Hugh Montgomery<br>Professor Kamlesh Khunti<br>Professor Mark Gabbay<br>Professor Nefyn Williams<br>Professor Paula Lorgelly<br>Professor Sir Michael Brady<br>Dr Angela Green<br>Dr Dan Wootton<br>Dr Emily Attree<br>Dr Emma Wall<br>Dr Gail Allsopp<br>Dr Melissa Heightman<br>Dr Michael Crooks<br>Dr Michael Zandi<br>Dr Nisreen Alwan<br>Dr Toby Hillman<br>Dr William Strain<br>Mr Lyth Hishmeh
**Trial Management Group**	Professor Amitava Banerjee (CI)<br>Dr Melissa Heightman (UCLH:Co-CI)<br>Mr Matthew Burns (UCLan)<br>Dr Gordon Prescott (UCLan)<br>Senior Clinical Trials Manager (LCTU)<br>Data Manager (LCTU)<br>Sponsor Representatives (UCL)
**Trial Steering Committee**	Independent members;<br>Professor Patrick Mallon (Paddy) – St Vincent University Hospital (Dublin) - Chair<br>Dr Marion Mafham – Nuffield Department of Population Health University of Oxford<br>Ondine Sherwood – Founder of Long COVID SoS and Public and Patient Involvement (PPI) Representative<br>Supported by Trial Managment Group members:<br>Professor Amitava Banerjee (CI) (University College London)

CONFIDENTIAL

	Dr Melissa Heightman (University College London Hospitals NHS FT:Co-CI) Mr Matthew Burns (University of Central Lancashire)<br>Dr Gordon Prescott (University of Central Lancashire)<br>Professor Matthew Sydes – (University College London)<br>University College London – Sponsor Representative
**Independent Data Monitoring Committee**	Professor Tim Peters (University of Bristol)<br>Prof Peter Langhorne (University of Glasgow)<br>Dr Ly-Mee Yu (University of Oxford)

CONFIDENTIAL

i. TABLE OF CONTENTS

RESEARCH REFERENCE NUMBERS 2
SIGNATURE PAGE 3
PROTOCOL VERSION HISTORY 4
KEY TRIAL CONTACTS 9
i. TABLE OF CONTENTS 12
ii. LIST OF ABBREVIATIONS 15
iii. TRIAL SUMMARY 17
iv. KEY ROLES AND RESPONSIBILITIES 23
v. KEY WORDS 23
1. BACKGROUND 24
2. RATIONALE 26
2.1. Further Rationale for the Trial and Interventions 27
2.2. Rationale for the Use of Drugs in STIMULATE-ICP Trial and Underlying Data 27
2.3. Predicted Efficacy of the Drugs in Long COVID 28
3. RISK ASSESSMENT AND MANAGEMENT OF RISKS 31
3.1. Risks Associated with Patient Population in the Trial 31
3.2. Risks Associated with non-IMP (Intervention) Component of the Trial 31
3.3. Safety Profile of the Trial IMPs 33
4. OBJECTIVES AND OUTCOME MEASURES/ENDPOINTS 37
4.1. Primary Objectives 37
4.2. Secondary Objectives 37
4.3. Primary Outcome Measure 37
4.4. Secondary Outcome Measures 37
4.5. Exploratory Outcome Measures / Endpoints 39
5. TRIAL DESIGN 39
5.1. Definitions 40
5.2. Justification for Cluster Randomisation 40
5.3. Design, Implementation and Evaluation of Integrated Care 41
5.4. Trial setting 45
6. INVESTIGATIONAL MEDICINAL PRODUCTS 45
6.1. Name and Description of IMP(s) 45
6.2. Formulation, Packaging and labelling of the IMPs 46
6.3. Source of IMP, Manufacture and Distribution 46
6.4. Storage and Handling of IMP(s) at Site 46
6.5. Accountability of IMP(s) 47
6.6. Regulatory Status of the IMP(s) 47
6.7. Dosage Schedules 47
6.8. Drug Interactions and Specific Contraindications 47
6.9. Consideration of Dose Modification 53
6.10. Concomitant Medications 53
7. TRIAL PROCEDURES 53
7.1. Selection of Participants 53
7.2. Participant Eligibility Criteria 53
7.3. Participant Identification 56
7.4. Recruitment of Trial Participants 57
8. REGISTRATION / RANDOMISATION PROCEDURES 61
8.1. Patient Registration 61

CONFIDENTIAL

8.2. Cluster Randomisation for Usual Care service uplift 62
8.3. Drug Trial Randomisation 62
8.4. Blinding 63
9. TRIAL ASSESSMENTS 63
9.1. Eligibility Assessments 63
9.2. Baseline Visit 64
9.3. Assessment Visit (12 Week Assessment) 67
9.4. Follow-up Assessment (24 Weeks) Visit 68
9.5. Laboratory Procedures 69
9.6. Schedule of Assessments 72
9.7. Post-Trial Treatment 74
10. DISCONTINUATION / EARLY CESSATION OF TREATMENT AND "STOPPING RULES" 75
10.1. Replacements 75
10.2. Cessation of Treatment and Withdrawal 75
10.3. Lost to Follow-up 76
11. SAFETY REPORTING / PHARMACOVIGILANCE 76
11.1. Definitions 76
11.2. Recording and reporting of Adverse Events 78
11.3. Assessing Adverse Events 78
11.4. Assessing Serious Adverse Events and Serious Adverse Reactions (nested drug trial participants only) 79
11.5. Serious Adverse Events Which Do Not Require Reporting to Sponsor 80
11.6. SUSAR Reporting 80
11.7. Adverse Event Recording and Processing Flow Chart 82
11.8. Notification of deaths (nested drug trial participants only) 83
11.9. Pregnancy reporting (nested drug trial participants only) 83
11.10. Overdose Reporting 83
11.11. Loratadine Overdose 84
11.12. Famotidine Overdose 84
11.13. Colchicine Overdose 84
11.14. Rivaroxaban Overdose 85
11.15. New Safety Findings 86
11.16. Urgent Safety Measures 86
11.17. Development of Safety Update Reports 86
11.18. Responsibilities 86
12. DATA MANAGEMENT AND QUALITY ASSURANCE 87
13. DATA COLLECTION AND SOURCE DOCUMENT IDENTIFICATION 88
13.1. Data Collection from Coverscan™ 89
13.2. Data Collection from Long COVID Clinics 89
13.3. Linked National Data Resource (Healthcare Systems Data) **Error! Bookmark not defined.**
13.4. Completing Case Report Forms 89
13.5. Data Handling and Analysis 90
13.6. Access to the final trial dataset 90
14. STATISTICAL CONSIDERATIONS 90
14.1. Sample Size Calculation – Main study 90
14.2. Sample Size Calculation – Nested Drug Trial (and Non-Nested Drug Trial) 91
14.3. Planned Recruitment Rate 92
14.4. Randomisation Methods 92
14.5. Statistical Analysis Plan 93
14.6. Summary of Baseline Data and Flow of Participants 93
14.7. Primary Outcome Analysis 93
14.8. Secondary Outcome Analysis 94

CONFIDENTIAL

14.9. Sensitivity and other planned analysis 94
14.10. Interim Analysis 95
14.11. Proteome Statistical Analysis 95
15. END OF TRIAL 96
16. RECORD KEEPING AND ARCHIVING 97
17. OVERSIGHT COMMITTEES 97
17.1. Trial Management Group (TMG) 97
17.2. Trial Steering Committee (TSC) 97
17.3. Independent Data Monitoring Committee (IDMC) 98
17.4. Stopping Rules 98
18. MONITORING, AUDIT, AND INSPECTION 98
18.1. Direct Access to Source Data 99
19. ETHICS AND REGULATORY REQUIREMENT REPORTING 99
19.1. Peer Review 99
19.2. Public and Patient Involvement 100
19.3. Regulatory Compliance 100
19.4. Data Protection and Participant Confidentiality 101
19.5. Financial and other competing interests for the Chief Investigator & PIs at each site 103
19.6. Insurance and Indemnity 103
20. PUBLICATION & DISSEMINATION 104
20.1. Publication Policy 104
20.2. Open Access and Data Sharing 104
20.3. Authorship Eligibility 105
21. REFERENCES 106
APPENDICES 109
Appendix I 109
Appendix II 120

CONFIDENTIAL

ii. LIST OF ABBREVIATIONS

AE	Adverse Event
AR	Adverse Reaction
BD	Twice Daily
CA	Competent Authority
CI	Chief Investigator
CRF	Case Report Form
CTA	Clinical Trial Authorisation
CTIMP	Clinical Trial of Investigational Medicinal Product
DHSC	Department of Health & Social Care
DI	Designated Individual
DSUR	Development Safety Update Report
EC	European Commission
EMA	European Medicines Agency
EQ-5D-5L	Euroqol 5 level instrument (5 questions on mobility, self-care, usual activities, pain or discomfort and anxiety or depression)
EU	European Union
EUCTD	European Clinical Trials Directive
EudraCT	European Clinical Trials Database
EudraVigilance	European database for Pharmacovigilance
GAD-7	Generalised Anxiety Disorder-7 questionnaire
GAfREC	Governance Arrangements for NHS (National Health Service) Research Ethics
GCP	Good Clinical Practice
GP	General Practitioner
HRA	Health Research Authority
IC	Integrated Care
ICP	Integrated Care Pathway
ICF	Informed Consent Form
ICH	International Council on Harmonisation of technical requirements for registration of pharmaceuticals for human use
IDMC	Independent Data Monitoring Committee
IMP	Investigational Medicinal Product
IMPD	Investigational Medicinal Product Dossier
IPR	Individual Patient Randomisation
ISF	Investigator Site File
ISRCTN	International Standard Randomised Controlled Trials Number
LCTU	Lancashire Clinical Trials Unit
MHRA	Medicines and Healthcare products Regulatory Agency
MWSAS	Modified Work and Social Adjustment Scale
NHS R&D	National Health Service Research & Development
OD	Once Daily
PCN	Primary Care Network
PCS	Post COVID Syndrome
PDQ	Perceived Deficit Questionnaire

CONFIDENTIAL

PHQ-9	The Primary Care Evaluation of Mental Disorders Patient Health Questionnaire
PI	Principal Investigator
PIS	Participant Information Sheet
QA	Quality Assurance
QC	Quality Control
QP	Qualified Person
RCT	Randomised Control Trial
REC	Research Ethics Committee
RSI	Reference Safety Information
SAE	Serious Adverse Event
SAR	Serious Adverse Reaction
SDV	Source Data Verification
SF12	Short Form Questionnaire 12 questions
SOP	Standard Operating Procedure
SmPC	Summary of Product Characteristics
SUSAR	Suspected Unexpected Serious Adverse Reaction
TMF	Trial Master File
TMG	Trial Management Group
TSC	Trial Steering Committee
UC	Usual Care or Standard Care
UCL	University College London
UCLAN	University of Central Lancashire
UCLH	University College London NHS Foundation Trust
USM	Urgent Safety Measure
WOCBP	Women of Childbearing Potential
WP	Work Package
WSAS	Work and Social Adjustment Scale

CONFIDENTIAL

iii. TRIAL SUMMARY

**NIHR Project Title**	**Symptoms, Trajectory, Inequalities and Management: Understanding Long-COVID to Address and Transform Existing Integrated Care Pathways**
**Trial Title**	A pragmatic, multi-centre, cluster-randomised trial of an integrated care pathway with a nested Phase III, open label, adaptive platform, randomised drug trial in individuals with Long COVID.
**Short Title**	STIMULATE-ICP
**Sponsor Protocol Number**	143602
**Active IMPs**	Famotidine, Loratadine, Colchicine and Rivaroxaban
**Formulation, Dose, Route of Administration**	Famotidine (40mg) once daily by mouth + Loratadine (10mg) tablets once daily by mouth, or Colchicine (500mcg) tablet twice daily by mouth, or Rivaroxaban (10mg) tablet once daily by mouth
**Trial Participants**	i. Individuals with Long COVID referred to participating Long COVID clinic, presenting at first referral (in person or virtual)<br>ii. Individuals previously seen at clinic prior to study commencement (with ongoing current long-COVID symptoms) into drug study or drug study + blood sampling only.
**Evaluable Sample Size**	Maximum 4,520 in the nested drug trial
**Estimated Total Trial Duration**	30 months
**Estimated Trial Duration per Participant**	24 Weeks
**Trial Objectives**	Primary:<br><br>To evaluate: “integrated care” with combinations of multi-organ MRI (Coverscan™) and clinical decision support; and digitally enabled, community rehabilitation: Living with COVID Recovery™ versus “usual care” [Usual Investigations; and self-management website].<br><br>Secondary:<br><br>To evaluate:

CONFIDENTIAL

	1. Clinical efficacy of the individual components of the integrated care pathway including potential therapies.<br>2. Effect of the ICP on mean measured health related quality of life, mental health (including depression), work and social adjustment, physical function, organ impairment and healthcare utilisation through patient reported outcomes.<br><br>Exploratory:<br><br>To characterise the pathophysiology and trajectory of Long COVID.
**Trial Design**	Pragmatic, cluster-randomised trial with nested, Phase III, open-label, adaptive platform drug trial. (Using a platform approach, new drugs may be added to the protocol, pending approval from NIHR and Department of Health and Social Care (DHSC)).
**Trial Methods**	I. In a pragmatic, cluster (primary care network-level)-randomised trial, we will evaluate integrated care pathway combinations with early, supported investigation (multi-organ MRI with Coverscan™ and clinical decision support) and enhanced rehabilitation (Living with COVID Recovery™), compared with usual care (Usual Investigations and Your COVID Recovery™ self-management), recruiting adults with suspected Long COVID at referral.<br><br>II. In a nested, patient-level, adaptive platform drug trial, we will investigate Famotidine plus Loratadine in combination, or Colchicine, or Rivaroxaban, versus standard of care, with the option to test further drugs using an adaptive platform. The nested data will be supplemented by equivalent non-nested patient-level, adaptive platform drug trial data, of the same drugs versus standard of care, with the option to test further drugs using an adaptive platform.<br><br>The primary outcome measure is Fatigue Assessment Scale at 12 weeks.<br><br>III. Long COVID is a new disease or syndrome, and mechanisms of disease and potential mechanisms of new treatments need to be analysed. Baseline blood samples will be taken from all participants who consent to gift bloods to allow our team to

CONFIDENTIAL

CONFIDENTIAL

	look at the underlying disease processes. This will involve standard clinical tests (e.g., haematology and biochemistry) as well as looking at research areas such as genomics. Samples will be stored to develop a biobank for use by the broader global research community in the largest study of Long COVID patients to date.
**Clinical Phase**	Phase III
**Inclusion & Exclusion Criteria**	**Participant Inclusion Criteria for ALL Participants**<br><br>1. Participants capable of giving informed consent.<br>2. Age 18 years and above<br>3. Clinical Parameters; persistent signs and symptoms for a period of 4 weeks or longer in duration post-COVID-19 infection (either by test result or symptomology).<br>4. Able to read or understand English or have a relative/family member able to read/understand English to facilitate participation (essential for patient reported outcome measures at follow-up time points and virtual contact).<br>5. Not enrolled in any other interventional study where study intervention/activities may affect outcome measures (patients enrolled in purely observational studies can be included)<br><br>**Additional Participant Inclusion Criteria for the cluster randomised integrated care pathway study**<br><br>6. Presenting at their first referral to a participating Long COVID clinic pathway.<br><br>**Additional Participant Inclusion Criteria for the nested, platform randomised drug trial** <u>(to be eligible participants must meet **above criteria** and all those below)</u><br>*Note: Potential participants with drug-specific contraindications for any arm, including interactions of pre-prescribed essential medication will be consented for data collection but will be excluded from the drug study.*<br><br>7. Presenting at their first referral to a participating Long COVID clinic pathway or previously referred to a participating Long COVID clinic and still suffering from Long COVID symptoms<br>8. Females of childbearing potential must be willing to use an acceptable, effective method of contraception during treatment with the IMP and for 30 days after the last dose. Such methods include:

	i. combined (oestrogen and progestogen containing) hormonal contraception:<br>a) oral<br>b) intravaginal<br>c) transdermal<br>ii. progestogen-only hormonal contraception<br>a) oral<br>b) injectable<br>c) implantable<br>iii. intrauterine device (IUD)<br>iv. intrauterine hormone-releasing system (IUS)<br>v. bilateral tubal occlusion<br>vi. vasectomised partner<br>vii. male or female condom with spermicide<br>viii. cap, diaphragm or sponge with spermicide sexual abstinence (true abstinence)<br>9. Male participants must be willing to use condom during IMP treatment and for a further 90 days after the last dose of trial IMP to protect their female partner becoming pregnant.<br><br>10. Patients on pre-existing treatments for the same drug classes or contraindicated drugs MUST undergo a 7-day washout period before being randomised.<br>*(Patients will be assessed, and if safe to exclude that medication for 7 days, they will be asked if they would be willing to undergo a washout period of at least 7 days after consent but before being randomised and exclude that medication until 7 days after last dose of IMP)*<br><br>**Participant Exclusion Criteria for ALL Participants**<br>1. Previously hospitalised for COVID-19 infection.<br><br>**Additional Participant Exclusion Criteria for Research Blood Sampling**.<br>2. Participants with any known blood borne viruses (BBV's) including Hepatitis C, Hepatitis B and/or HIV<br>3. Participants with any condition that may severely prolong clotting times

CONFIDENTIAL

	**Additional Participant Exclusion criteria for nested, adaptive randomised drug trial**<br>4. Females who are pregnant, planning pregnancy or breastfeeding<br>5. Known hypersensitivity to any of the study drugs or their excipients<br>6. Currently taking any of the following drugs:<br>Probenecid, Sucralfate, Isocarboxazid, Phenelzine, Tranylcypromine of any other CNS depressant (such as diphenhydramine, dextromethorphan, or pseudoephedrine) (Contraindications to Famotidine/Loratadine)<br>Amiodarone, Aprepitant, Atazanavir, Atorvastatin, Azithromycin, Bezafibrate, Ciclosporin, Ciprofibrate, Clarithromycin, Cobicistat, Croztibib, Darunavir, Diltiazem, Dronedarone, Eliglustat, Erythromycin, Fenobibrate, Fluconazole, Fluvastatin, Fosamprenavir, Gemfibrozil, Idelalisib, Imatinib, Isavuconazole, Itraconazole, Ketoconazole, Letermovir, Lopinavir, Netupitant, Nilotinib, Posaconazole, Pravastatin, Ranolazine, Ritonavir, Rosuvastatin, Simvastatin, Tipranavir, Velpatasvir, Vemurafenib, Venetoclax, Verapamil, Voriconazole (Contraindications to Colchicine)<br>Acalabrutinib, Aceclofenac, Acenocoumarol, Alprostadil, Alteplase, Argatroban, Aspirin, Axitinib, Beniparin, Benzydamine, Bevacizumab, Bismuth, Bivalirudin, Bosutinib, Bromfenac, Cabozantinib, Cangrelor, Caplacizumab, Celecoxib, Cilostazol, Clopidogrel, Cobimetinib, Dabigatran, Dalteparin, Danaparoid, Dasatinib, Dexkeptorofen, Diclofenac, Dipyridamole, Enoxaparin, Epoprostenol, Eptifibatide, Erlotinib, Etodolac, Etoricoxib, Flurbiprofen, Gefitinib, Heparin, Ibrutinib, Ibuprofen, Iloprost, Imatinib, Indomethacin, Inotersen, Ketoprofen, Ketorolac, Levatinib, Mefenamic acid, Meloxicam, Nabumetone, Naproxen, Nicotinic acid, Nintenanib, Parecoxib, Pazopanib, Phenazone, Phenindione, Piroxicam, Ponatinib, Prasugrel, Regorafenib, Ruxolitinib, Sorafenib, Streptokinase, Sulindac, Sunitinib, Tenecteplase, Tenoxicam, Tiaprofenic acid, Ticagrelor, Tinzaparin, Tirofiban, Tolfenamic acid, Trametinib, Traztuzumab emtansine, Treprostinil, Urokinase, Volanesorsen, Warfarin (Contraindications to Rivaroxaban)<br>7. No history or presenting symptomology suggestive of Renal failure/insufficiency (eGFR <30 mL/min) on the basis of blood investigations (eGFR) within the last 6 months and clinical assessment

CONFIDENTIAL

	8. Severe liver dysfunction on the basis of blood investigations within the last 6 months (liver function and in some cases coagulation) and clinical assessment.<br>9. Patients with blood dyscrasias
**Statistical Methodology and Analysis**	Primary Outcome – Effectiveness: Fatigue at 12 weeks (measured with the Fatigue Assessment Scale) analysed on an intention-to-treat basis. An analysis of covariance will be employed to estimate the effects of Coverscan™, digitally enabled community rehabilitation, and their interaction.

CONFIDENTIAL

iv. KEY ROLES AND RESPONSIBILITIES

SPONSOR: University College London (UCL) will act as Sponsor for this trial. The sponsor is responsible for ensuring that before the trial begins arrangements are in place for the research team to access resources and support to deliver the research as proposed, and allocate responsibilities for the management, monitoring and reporting of the research. The Sponsor also must be satisfied that there is agreement on appropriate arrangements to record, report and review significant developments as the research proceeds, and approve any modifications to the study design. The Sponsor will maintain oversight of the trial, be involved in all research contract negotiations, and sit on the Trial Management Group. UCL will provide insurance for the clinical trial and undertake to ensure that the above trial is conducted in accordance with the Medicines for Human Use (Clinical Trials) Regulations 2004 (as amended), the UK Policy Framework for Health and Social Care Research 3rd edition 2017 (as amended) and all applicable regulatory requirements. The Sponsor has delegated some of its responsibilities to the Lancashire Clinical Trials Unit.

Lancashire Clinical Trials Unit (LCTU) is managing the trial setup, submission and will oversee regulatory approvals for all aspects of the trial and manage the conduct of the STIMULATE-ICP trial in conjunction and or on behalf of the Sponsor. LCTU is responsible for the maintenance of the trial related documentation and archiving. Research Agreements and standard service level agreements will be established with all relevant organisations and stored at LCTU and Joint Research Office (Sponsor) at UCL.

FUNDER: The funder is the entity that will provide the funds (financial support) for the conduction of the study. Funders are expected to provide assistance to any enquiry, audit or investigation related to the funded work.

CHIEF INVESTIGATOR (CI): The person who takes overall responsibility for the design, conduct and reporting of the trial. If the trial involves researchers, clinicians, and academic personnel at more than once site, the CI takes on the primary responsibility for the suitability of an investigator at any particular site.

PRINCIPAL INVESTIGATOR (PI): Individually or as leader of the researchers at a site; ensuring that the study is conducted as per the approved STIMULATE-ICP protocol, and report/notify the relevant parties – this includes the CI of any breaches or incidents related to the trial.

v. KEY WORDS

Long COVID, diagnostic, epidemiology, informatics, Health Service Research, Clinical Trials, Coronavirus, Integrated Care Pathway, Long Term Condition, Work Packages

CONFIDENTIAL

1. BACKGROUND

Long COVID (1) spans multiple healthcare challenges, particularly how to deliver sustainable, high-quality care for multimorbidity and long-term conditions (LTC). There are inequalities in health and access to healthcare, incomplete pathways across community and hospital care, inadequate research translation to practice, and insufficient resources. Integrated care pathways (ICP) are structured multidisciplinary care plans of essential steps in care of specific conditions (2). An effective and deliverable ICP for Long COVID could reduce direct and indirect pandemic effects and improve LTC care beyond COVID-19 and potentially be cost effective. Long COVID services were initiated whilst research is still defining the disease. Guidelines are developing in the absence of evidence. An ICP approach offers coordination across specialties, across investigation, treatment, and rehabilitation, as well as opportunities for real-time iterative improvements in service design and delivery. ICPs are of increasing relevance in many LTCs. ICPs can improve quality and access to care, patient experience and satisfaction, while reducing complications and non-elective admission rates (3-5). Research and trials of ICPs should incorporate design elements “likely to maximise effectiveness” (5) and engagement of patients, clinicians and health systems to improve healthcare performance (6).

Long COVID is a syndrome, not a single condition. The “syndemic” of COVID-19 (convergence of an infectious disease, under-treated non-communicable diseases, and geographical, and economic determinants of health (7)) necessitates ICP strategies, which could inform other LTCs. Predictors of poor acute COVID-19 outcomes are established (8), but effective treatments for Long COVID are not yet defined (9, 10).

The NIHR-funded STIMULATE-ICP project consists of three inter-connected, cross-linked **Work Packages,** which are led and managed by a multidisciplinary team across different organisations from academia, health services and industry.

The work packages are designed to be time-sensitive, iterative, adaptive and effective, utilising learning health systems (health science, clinical evidence, and care) (11), implementing science (11-13) and complex interventions (14).

Figure 1 is illustrative of the inter-connection between the different work packages for context purposes only.

This protocol details work package 2 which is a pragmatic, multi-centre, cluster-randomised, Phase III trial of an integrated care pathway with a nested, adaptive, open label, randomised drug trial in individuals with Long COVID.

CONFIDENTIAL

Figure 1: The Schema for STIMULATE-ICP Grant award showing the interconnections between this STIMULATE_ICP Trial and other work packages

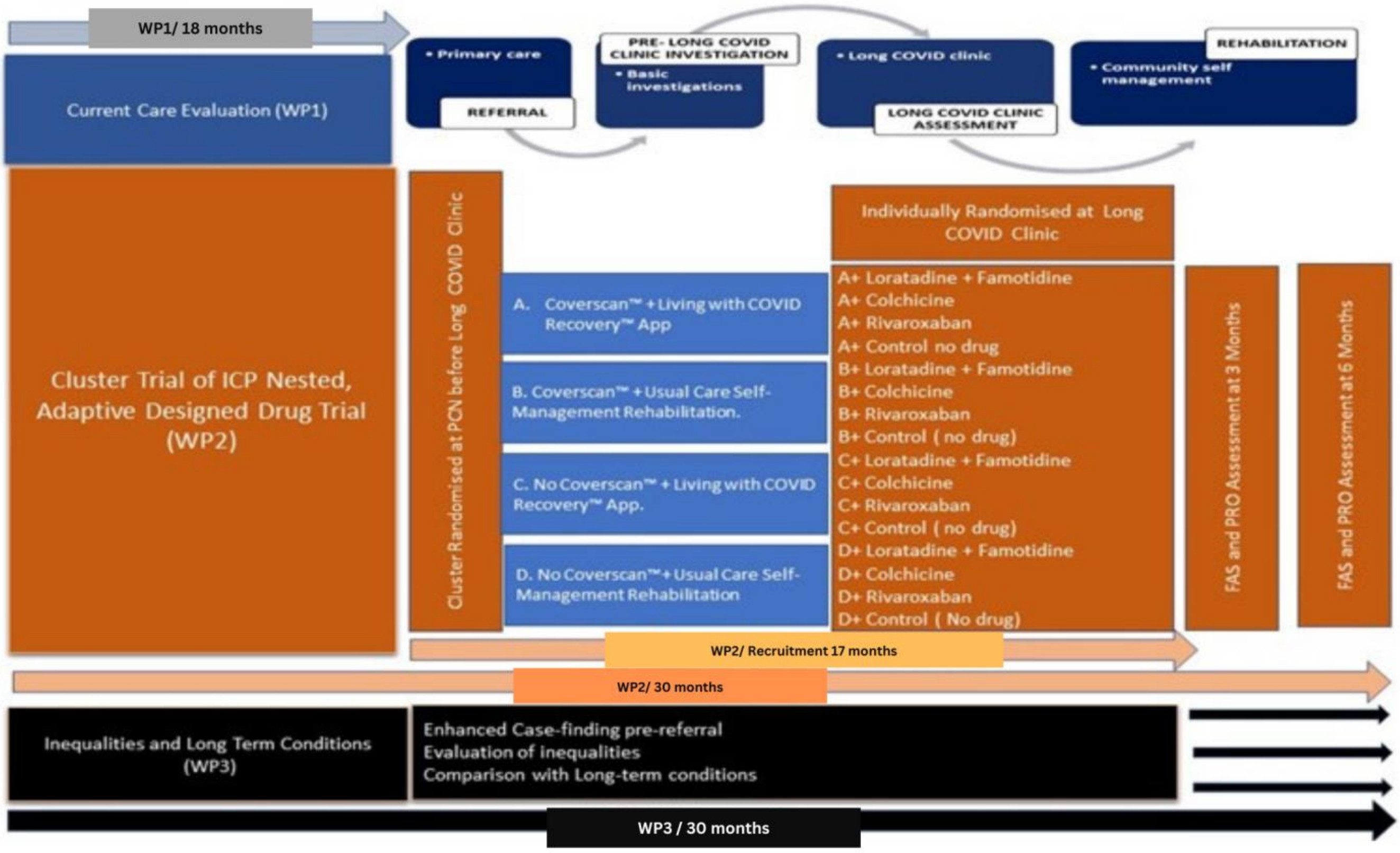

2. RATIONALE

There is variation in access to referral and care, and poor patient experience for Long COVID (1). With stretched resources during the pandemic, the need for rationalisation of investigation and rehabilitation across diseases is paramount. In Long COVID, where up to 70% of patients show evidence of mild impairment in one or more organs, (15) an early supported investigation strategy may direct management, as well as better defining the syndrome, with relevance to other LTCs. Despite the primary care setting, there is no evidence-based treatment or rehabilitation. There have been few Long COVID clinical trials to date, particularly in non-hospitalised individuals, or in the context of overall care pathways. As Long COVID clinics are implemented, trials of ICPs can inform diagnosis, care, public health, policy planning, resource allocation and budgeting.

In the UCLH Long COVID clinic (the first and largest in the UK), community-referred patients were more symptomatic, more likely to have psychological impacts, and less likely to be fit for work than patient's post-hospitalisation. Any ethnic and socioeconomic disparities in referrals, assessment, and rehabilitation (16) need to be understood at a system level and compared with other LTCs, to provide lessons beyond COVID-19, which has worsened pre-existing inequities (7). Pragmatic implementation of effective and equitable interventions is urgently needed to reduce inequalities (17).

Long COVID is estimated to have a substantial population burden, with 2 million people affected in the UK. Even in non-hospitalised individuals, up to 14% of people with COVID-19 may have persistent symptoms (3), suggesting future major healthcare and economic burden (4, 18). We are challenged "to design research that integrates reported lived experience needs with clinical models of care" (6). Guidelines recommend referral to Long COVID clinics if symptoms persist for 4-12 weeks (10). However, neither the syndrome nor effective care is defined (19). Despite roll-out of 83 centrally funded Long COVID clinics, ICPs are not coordinated and the most effective ICP remains unknown because elements of the pathway and their delivery are lacking in evidence. Patients and public want "recognition, research and rehabilitation" simultaneously (14), as well as timely tests and treatment, which may be more deliverable via an ICP.

Real world data needs to be analysed to understand the trajectory of Long COVID, the nature of "usual care" and the effectiveness of current care pathways. These data have already informed our choice of interventions for diagnosis and rehabilitation for a cluster-randomised trial.

Coverscan™ is a multi-organ MRI assessment to assess the impact of COVID and Long COVID on affected organ function. This is either by scanning the whole organ or (in the case of the lungs) by cross sectional scanning for representational function assessment.
Coverscan™ is a UKCA marked medical device which also has exceptional use authorisation by the MHRA and needs to be evaluated at scale, where there is currently significant variation in investigations prior to Long COVID clinic. Self-management has been the mainstay of rehabilitation in usual care, but more comprehensive, digitally enabled programmes need to be quickly evaluated, such as Living with COVID Recovery™. Rather than studying specific components in isolation, given the scale of Long COVID and the urgency of the public health burden, integrated pathways need to be evaluated.

In addition to the components of the ICP, we need a robust evidence-base to inform drug therapies for effective treatment of Long COVID. Some medications are being widely used among patients and health professionals without evidence base.

As a pragmatic trial design based in routine care, it is important to analyse data pre-trial (WP1), data from the trial and demographic and socio-economic data to monitor inequalities (WP3) in the intervention, as well as considering applicability to other LTCs.

2.1. Further Rationale for the Trial and Interventions

Patient surveys, interviews with patients, health professionals and policymakers, and data from UCLH clinic suggest a lack of coordination of care and missed opportunities to improve investigation and rehabilitation. An example of this is repetitive investigations in different modalities for the same organ, and different scans at different times for different organs. One of the Integrated Care components that we will assess is Coverscan™ (15) which is a non-CE marked medical device given MHRA-authorisation for exceptional use in research and clinical care and has been used in 500 patients. It aligns with national policy for community diagnostics (NHS Diagnostic Hubs (34)). Interviews with clinicians, patients and researchers show support for Coverscan™ to be used as an early investigation strategy, to be evaluated in routine care, and to help stratify treatment.

The second component of Integrated Care to be assessed is "Living with COVID Recovery™" which is an NIHR-funded digital health intervention (NIHR132243), initiated in August 2020 (36), providing videos, information and rehabilitation treatments under guidance of a rehabilitation clinician. The "Living with COVID Recovery™ App is a class I, CE Marked Medical Device. Over 400 patients in 15 of the 83 Long COVID clinics (mean age: 54 years, range 23–78; 50% from ethnic minorities), have used the app to-date. Patients perform seven actions per week such as weight recording, patient reported outcome measures (PROMs), completion of GAD-2, MRC Dyspnoea score or exercise tracking, with half creating and tracking chosen goals. A physiotherapist needs an hour to review each patient, managing 70 individuals per month. Clinicians find it efficient and patient centred.

2.2. Rationale for the Use of Drugs in STIMULATE-ICP Trial and Underlying Data

To date, little to no treatment data exists for Long COVID; understanding of the pathophysiological mechanisms of disease is poor, and few well-designed clinical trials are currently registered. UCLH hosts the first and largest of the NHS Long COVID clinics. In a cohort of >1000 patients living with Long COVID at UCLH, the most common reported symptoms are fatigue, shortness of breath and reduced exercise tolerance, similar to reports from other centres (16, 20). Median Fatigue Assessment Scores (FAS) at presentation are >30 in >60% of Long COVID patients (normal range FAS <20) irrespective of time since symptom onset. FAS scores are significantly higher than post-hospitalised patients, similar to other reports (21).

Proposed mechanisms underlying Long COVID symptom clusters include mast cell activation, microcirculatory deficiency caused by microthrombi, and mitochondrial insufficiency. Preliminary observations by our UCLH team suggest Long COVID patients have evidence of persistent inflammation and/or microcirculatory insufficiency, resulting in poor aerobic muscle capacity and profound fatigue. The

CONFIDENTIAL

nested STIMULATE-ICP trial interventions support existing hypotheses and are based on data from UCLH Long COVID clinic patients.

We hypothesise that drug treatments in Long COVID may significantly improve symptoms and physical function, through different mechanisms of action. We have designed an adaptive, platform clinical trial (22-25) within STIMULATE-ICP, to robustly test efficacy of drugs in Long COVID. We will start with Loratadine plus Famotidine, Colchicine and Rivaroxaban and continue to add/remove drugs according to emerging preliminary data in Long COVID and as approved by the Independent Data Monitoring Committee (IDMC), Trial Steering Committee (TSC) and the Funder (NIHR). It is likely that Long COVID represents a heterogenous group of different pathological responses to primary mild to moderate COVID-19 infection, with unknown predisposition. However, since the underlying cause(s) of Long COVID are unknown, we have chosen to test these drugs across all participants with Long COVID, irrespective of their individual symptoms or investigation findings, as we cannot currently determine which approach is optimal to treat the hypothesised underlying inflammatory process. This approach will minimise selection bias and allow multiple secondary analyses to test for efficacy in particular sub-groups of patients, based on symptom clusters or imaging findings.

The large sample size will enable us to undertake multiple pre-specified "symptom phenotypes" analyses based on both Coverscan™ findings and symptom clusters to determine if these sub-groups do benefit from specific drugs or not.

Estimating the individual effect size of these drugs on fatigue is complex, as extrapolating preliminary data to the larger patient cohort is difficult. We hypothesise that Long COVID patients who experience symptoms may gain most benefit from the trial drugs, but in the absence of detailed pathophysiological data for Long COVID, we are open to possibility of differential effects of these drugs in different sub-groups.

Alternative treatment arms may be included/changed during the trial at pre-specified points on the recommendation of the IDMC and TSC, based on advice and involvement from the COVID-19 UK Government Therapeutics Task Force at the Department of Health & Social Care.

2.3. Predicted Efficacy of the Drugs in Long COVID

Long COVID is a new condition, without consensus on the underlying pathophysiology. While a myriad of symptoms is reported by Long COVID patients, fatigue is the most frequent symptom in our experience of over 1000 Long COVID patients and most commonly reported, as the symptom that most limits quality of life (21, 26). Among other symptoms reported are chest pain, cognitive slowing, reported commonly as 'brain fog', dyspnoea, headache, dizziness, palpitations, and sleep disturbances.

The drugs selected for use in the STIMULATE-ICP trial are based on emerging data from our service and other Long COVID clinics, patient groups and emerging, early clinical trials.

Using an adaptive platform approach, new drugs may be added to the protocol, following discussions and approval from the NIHR specialist advisory group for Long COVID, through amendment to this protocol.

2.3.1. Loratadine and Famotidine ($H_1 + H_2$ Receptor Blockade)

Loratadine and Famotidine are both histamine receptor antagonists. In combination they inhibit both the H_1 and H_2 receptors. Famotidine is an effective competitive H_2 receptor antagonist. It reduces the concentration and amount of acid and pepsin of the gastric juices. The effect of oral administration is rapid, long lasting when used at the recommended dosage and it is effective with relatively low concentration in the blood. The duration of its effect, plasma concentration and secretion in the urine are dose-dependent. Famotidine is licensed as an over the counter (OTC) treatment for dyspepsia/gastric ulceration. Loratadine is also an OTC treatment licensed for treatment of mild allergic symptoms including seasonal rhinitis.

Patients with Long COVID are hypothesised to have a persistent inflammatory process that may drive the symptoms of fatigue and myalgia. In a small minority of patients, more specific symptoms suggestive of mast-cell activation, including rashes, diarrhoea and flushing, may be present. Histamine receptor antagonists are commonly used to treat mast-cell activation in other conditions.

Mixed reports of efficacy of histamine receptor antagonists in acute COVID-19, particularly reduction in oxygen requirements and possibly mortality, have led to ongoing clinical trials of these treatments in acute COVID-19 (27). The suggested mechanisms of action in acute COVID-19 are reduction in inflammation due to suppression of T-cell mediated cytokine release, however, available data are very limited. In an observational cohort study, 49 patients with Long COVID were compared to matched asymptomatic fully recovered COVID-19 patients without Long COVID. Long COVID patients were offered treatment with combined histamine H1/H2 blockade, using H1 (Loratadine 10 mg once daily or Fexofenadine 180 mg twice daily (not being used in this study) and H_2 (Famotidine 40 mg once daily or Nizatidine 300 mg once daily (not being used in this study)) for a minimum of 4 weeks (28), based on data from acute COVID-19 suggesting histamine receptor antagonist therapy improved symptoms (29, 30). In this preliminary observational study, patients with physician diagnosed Long COVID reported a 48% reduction in symptom burden after 4 weeks of combined histamine receptor antagonist therapy compared to baseline. When compared to recovered non-hospitalised patients, patients with Long COVID had significantly lower circulating CD4 but not CD8 effector memory cells, suggesting that this T cell subset may be involved in Long COVID pathology (28). These data, however, have significant limitations and the findings require testing in a formal clinical trial.

In this study we will be using Famotidine 40mg once daily in combination with Loratadine 10mg OD, ensuring combined histamine receptor antagonist activity across the H_1 and H_2 receptors. Both drugs are being used within their licensed dose and are safe to be co-administered.

2.3.2. Colchicine

Colchicine inhibits cellular transport and mitosis by binding to tubulin and preventing its polymerisation as part of the cytoskeleton transport system. Colchicine has a short half-life of 9-12 hours and is prescribed as a twice daily (BD) dosing regimen. Standard doses for acute gout range from 500mcg BD to 2mg BD, depending on the dose response. Colchicine has a wide range of anti-inflammatory effects, including inhibition of certain inflammasomes (cytosolic pattern recognition receptor systems that are activated in response to detection of pathogens in the cytosol) (31), (32). Evidence shows that inflammasomes are activated in COVID-19, and the degree of activation is correlated with disease severity (33).

CONFIDENTIAL

Colchicine has been shown to have cardiovascular benefit in patients with coronary artery disease and pericarditis at a dose of 500mcg BD. Its primary mechanism of action is reduction of serositis, inflammation of membranes around joints and viscera. Patients with Long COVID frequently complain of symptoms suggestive of serositis, either atypical chest pain that may indicate pericarditis, costochondritis or pleural inflammation, or joint pain in the absence of clinically evident inflammation. Disease severity in Long COVID correlates with myocardial damage on Coverscan™ (15). Of the non-hospitalised patients with Long COVID at UCLH, 20% report chest pain and or palpitations, 60% report shortness of breath. Standard investigations are frequently normal: prolonged rhythm monitoring typically shows resting sinus tachycardia, chest pain is generally atypical with predominantly normal echocardiogram, Troponin T and ECG. In a prospective cohort of over 50 patients (median age 43, 69% female) reporting persistent chest pain underwent cardiac MRI (CMR). 26% had evidence of myocarditis-pattern late gadolinium enhancement and/or evidence of abnormalities, (meeting diagnostic criteria for myocarditis (34). T1/T2 abnormalities were seen in 29%. Long COVID patients with myocarditis contrasted to patients admitted to hospital with moderate/severe respiratory COVID (mean age 64, 66% male). However, the prevalence of myocarditis is strikingly similar (35). To date, we have treated in excess of 200 Long COVID patients with abnormal CMR empirically with Colchicine 500mcg BD, 9/10 reported significant symptomatic improvement within 3 months.

2.3.3. Rivaroxaban (Low Dose Anticoagulation)

Rivaroxaban is an oral factor Xa inhibitor, inhibiting the clotting cascade. Patients with Long COVID who complain of marked exertional fatigue with abnormalities on the 6-minute walk test, have evidence of microvascular anaerobic respiration despite normal oxygenation in peripheral blood. This suggests that while adequate haemoglobin-bound oxygen is present in blood, the dissociated oxygen is unavailable to large muscles on increased aerobic demand during exercise. Abnormalities of the clotting cascade are a marked feature of acute Covid-19, including microvascular thrombi (36). Thrombi in acute Covid-19 are strongly associated with abnormally elevated von-Willebrand Factor (VWF): ag/ADAMTS 13 ratio (37). Recent data are showing longer term risk of VTE with Long COVID. We extended the measurement of VWF Ag/ADAMTS 13 levels to the UCLH community Long COVID patient cohort (Scully and Heightman, 2021. Unpublished data.) Of 272 patients in Long COVID clinic describing extreme lethargy, headaches and poor exercise tolerance, 81/272 (30%) had an abnormal VWF Ag/ADAMTS 13 ratio of >1.5. Elevated VWF Ag/ADAMTS 13 ratio strongly associated with impaired exercise capacity on a 6-minute walk test: 1.5 compared to 1.1 in patients with normal exercise capacity ($p<0.001$).

A further 28 patients had blood analysed by flow chamber assay, which measures VWF, platelet binding and in-vitro thrombus formation in real time; 8/28 (29%) had clot formation by 5 minutes. In a small feasibility project, 5 patients were initiated on low dose aspirin (LDA) 75 mg daily, selected based on symptom severity and elevated VWF(Ag): ADAMTS13 ratio. In 3/5 patients, improvement in symptoms and VWF(Ag): ADAMTS13 ratio was observed. The remaining two stopped aspirin due to upcoming procedure and bruising. Patients B and C were analysed on the flow chamber pre- and post-aspirin usage with marked improvement in surface coverage from 100% to 29% and 9% respectively on LDA, reporting corresponding symptom improvement.

CONFIDENTIAL

Evidence of microvascular thrombi in some patients has led to the hypothesis that endothelial dysfunction due to multiple microvascular thrombi in large muscles may significantly contribute to reduced aerobic capacity and symptoms of fatigue. Low-dose anticoagulation is a safe approach to test this hypothesis, by measuring fatigue as the primary outcome, we will determine if this approach indicates the presence of microvascular thrombi in Long COVID patients. Aspirin was poorly tolerated when tested in our pilot study due to gastro-intestinal side effects. We have included Rivaroxaban 10mg once daily in the STIMULATE-ICP trial instead of aspirin due to improved safety profile, easy comparability with other clinical trials using factor Xa inhibitors such as apixaban (HEAL-COVID) and proven efficacy data on prophylaxis of intravascular thrombi. The Rivaroxaban regime of 10mg OD proposed in STIMULATE-ICP is a prophylaxis dose, which aims to effectively prevent generation of further microvascular thrombi, while minimising bleeding side effects. This prophylaxis dose is therapeutically equivalent to low-dose aspirin tested in our preliminary cohort.

3. RISK ASSESSMENT AND MANAGEMENT OF RISKS

3.1. Risks Associated with Patient Population in the Trial

Patients with Long COVID Syndrome generally have poor prognosis for underlying health status, with few medical co-morbidities. Multi-morbid patients, or those with severe complications from their initial COVID illness (including pulmonary fibrosis) are at risk of increased adverse reactions to the study drugs.

Severely ill patients will be closely monitored by their treating physicians and the protocol accepts the requirement for unscheduled visits according to the clinical needs of the patients. Adverse events and biochemical abnormalities will be documented on a continuous basis the reporting procedure will be followed according to the Protocol and the Sponsor (UCL) SOP.

The research team will aim to recruit largely from the local population in the 6-10 designated geographical areas, to enable participants to travel to the clinics easily accessible to them. The recruitment period has allowed for these factors to be considered. Severely ill patients travelling from long distances may not be able to adhere to the scheduled visit but remain in the trial for the purpose of follow-up and remote assessments. A small percentage may withdraw. The trial team anticipate recruiting approximately 100 patients per month per area. The recruitment, the eligibility criteria and concomitant medications will be monitored and documented regularly (at least every 4 weeks in the first 24 Weeks of the trial).

In some areas, due to the design of the clinics, recruitment may need to be centralised to support eligibility screening, consenting, collection and processing of research bloods, and delivery of Living with COVID Recovery ™ digital App. A full risk assessment will be carried out for this trial; mitigations will be provided before the centres are activated and patients are recruited as part of the Sponsorship Green Light approval.

3.2. Risks Associated with non-IMP (Intervention) Component of the Trial

Coverscan™ is a multi-organ MRI scan for Long COVID. The scan has received Emergency Use Authorisation from the MHRA for the indication of investigation of Long COVID (23). The benefit of Coverscan™ is that it

CONFIDENTIAL

will provide a multi-organ assessment in individuals with Long COVID, which may help to map the trajectory of disease and to predict progression, recovery and/or response to potential treatments or rehabilitation.

The table below summarises the risks and mitigations of all non-IMP tests and/or procedures above Pre-ICP standard of care that are being performed:

CONFIDENTIAL

Table 1: Summary of the risks and mitigations of all non-IMP tests and /or procedures

Intervention	Potential risk	Risk Management
**Coverscan™: MRI is a safe and non-invasive technique with minimal risk to the patient. It does not involve ionising radiation or contrast media injection.**	In body ferromagnetic objects or implanted devices can be damaged by the MRI magnet or can cause patient injury.	On receipt of clinic referral, staff will screen the patient record for any obvious exclusion factors. Participants will receive an appointment letter outlining any exclusions and be asked to contact staff if they have any queries. Before entering the scanner room, all participants will be screened for any exclusion factors by the scanning radiographer and patients will confirm the assessment in writing.
	MRI can be noisy.	Patients will be provided with headphones and earplugs to reduce noise.
	Patients are required to remain still in a confined space and may feel anxious or the scan may have to be stopped due to patients becoming claustrophobic.	Participants will always be in communication with the scanning radiographer and may choose to end the scan at any time.
	While the risk is minimal, given the target population's post-infection circulating antibodies and the general immunisation status, the scanner room is a confined space and there is a risk of COVID-19 infection.	Adequate cleaning protocols between patient scans will minimise any risk of transmission and symptom screening will be done prior to scanning.
	Participants will be asked to not eat or drink for 3 hours prior to their MRI scans, but will be able to drink and eat after the scan.	To minimise any risk of infection, participants will be reminded to come equipped with a snack to consume after their MRI scan.
**Living with COVID Recovery™**	No known potential risks.	Any reports as to adverse effects of using the app will be monitored and reported to the site and TMG and Living with COVID Recovery™ manufacturer

CONFIDENTIAL

3.3. Safety Profile of the Trial IMPs

All the drugs used in this trial are licenced by the UK regulator (MHRA) for their therapeutic indications but not for Long COVID Syndrome. In accordance with the MRC/DH/MHRA Joint Project Risk-adapted

Approaches to the Management of Clinical Trials of Investigational Medicinal Products, this trial is categorised as **Type B = Somewhat higher than the risk of standard medical care.**

Patient medical records and past medical history will be examined for comorbidities that may interfere with the pharmacodynamic and bioavailability of the trial drugs. The exclusion of certain medical conditions will be implemented as part of the eligibility criteria to protect the safety of the participants. The enrolment of participants will be based on the inclusion and exclusion criteria, taking into account comorbidities, outcome of physical examination, laboratory investigations, functional tests and current drug prescriptions as part of standard care.

Screening of medical history, comorbidities and recorded clinic and GP ordered test results will mitigate against the risks posed by these drugs and reduce the burden of unwanted side effects of the trial IMPs.

Prior to randomisation all participants will have their clinic referral and clinic notes checked for comorbidities and concomitant medications to exclude conditions that might cause overdose or side effects relating to higher bioavailability or reduced clearance of the drugs. Their existing medications will be checked for contraindications to each of the drugs on the trial. Their pre-existing laboratory results will be examined for abnormalities that might potentiate the effects of the IMPs or their side effects. Important safety measures are Full Blood Count (FBC) within last 6 months, and no history or presenting symptoms to suggest renal or hepatic impairment. Any abnormalities of tests or suggestive symptomology will need to be considered carefully by the site PI and treating clinician (if different) and definitive tests ordered as appropriate before considering the participant for inclusion in the drug trial. Patients will be given a wallet card containing an emergency contact number and the details of the trial drugs in case urgent medical attention is needed. Common side effects and contact details of the trial team will be included in the Participant Information Sheet (PIS). Patients experiencing adverse reactions will be sign posted and advised to seek medical help from their General Practitioner (GP)s, or treating physicians if they are available, and will be treated as per Standard of Care treatment available on NHS.

3.3.1. Safety Profile of Loratadine

Loratadine is a long-acting, non-sedating tricyclic antihistamine (piperidine derivative) with selective antagonistic properties to peripheral histamine H_1-receptors. Loratadine selectively inhibits H_1-receptors primarily located on respiratory smooth muscle cells, vascular endothelial cells, the gastrointestinal tract, and immune cells. Loratadine has a long-established safety record and is widely available over the counter as well as by prescription.

At the recommended dose of 10mg daily, adverse reactions with Loratadine were reported in 2% of patients in excess of those treated with placebo. The most frequent adverse reactions reported were somnolence (1.2%), headache (0.6%), increased appetite (0.5%) and insomnia (0.1%), according to the SmPC: Clarityn Allergy (Loratadine) 10mg Tablets (Bayer plc).

Loratadine should be administered with caution and at reduced doses in patients with severe liver impairment because they may have reduced clearance of Loratadine. Therefore, patients with severe liver impairment will be excluded from the trial.

Participants, before entering the trial and before issued with a new prescription for the trial drugs, will be screened for contraindicated medications, in particular for concomitant medications with other CNS depressants such as diphenhydramine, dextromethorphan, or pseudoephedrine as these may cause dose-related sedation. Patients will be advised to use caution when driving or operating heavy machinery as it may cause drowsiness. Patients with Chronic Obstructive Pulmonary Diseases may experience flare-up of the condition due to its anticholinergic effects.

3.3.2. Safety Profile of Famotidine

Famotidine is a competitive histamine H_2-receptor antagonist that binds to the histamine receptors located on the basolateral membrane of the parietal cell in the stomach, effectively blocking histamine actions. It decreases the production of stomach acid and is used in the treatment of acid-related gastrointestinal conditions. Famotidine is available both by prescription and over the counter.

Most common adverse effects include agitation in less than 1%, headache (5%), dizziness (1%), diarrhoea (2%), and constipation (1%), according to the SmPC: SmPC Famotidine 40mg Tablets (Tillomed Laboratories Ltd).

Famotidine is secreted mainly via the kidneys; caution should be exercised when treating patients with renal insufficiency. Patients with creatinine clearance (eGFR) below 30 mL/min will be excluded from the study.

Patients will be informed to notify the research team and their treating physician if they experience frequent chest pain and frequent wheezing (atrioventricular block is very rare but serious side effect), unexplained weight loss, stomach pain, heartburn for a duration exceeding 12 weeks (Famotidine could mask the symptoms of underlying malignancy, therefore underlying malignancy must be excluded), heartburn with light-headedness, sweating, or dizziness. These symptoms will stop with discontinuation of the trial drug.

3.3.3. Safety Profile of Colchicine

Low-dose Colchicine has been used safely for many years for the prevention of inflammatory complications of Familial Mediterranean Fever and Bechet's disease. In contrast to the higher doses of Colchicine used for treatment of acute gout, low dose (0.5mg to 1mg/day) Colchicine used in recent cardiovascular trials (CORE) (64) shows excellent safety profile and the frequency of side effects were less than 2%.

According to the SmPC: SmPC Colchicine Tablets BP 500 mcg (Wockhardt UK Ltd), the side effects associated with using Colchicine are highly dose dependent, infrequent, and usually stops following reductions in the dosage or complete cessation of the treatment. Of note, Colchicine is genotoxic (in vitro and in vivo) and teratogenic in animal studies and contraindicated in pregnant females. However, a 2018 metanalysis of 4 trials concluded that in pregnant and breastfeeding women prescribed Colchicine, no increased incidence of foetal malformations or miscarriage was noted (38). All participants of child-bearing potential enrolled in STIMULATE-ICP will be required to use an acceptable, effective method of contraception (see section 7.2 and Appendix II for definition of acceptable effective methods of contraception for this trial) for up to 30 days after treatment discontinuation and females of childbearing potential will undertake a pregnancy test prior to enrolment. Male participants will be required to use a condom to protect their female partner from becoming pregnant for up to 90 days after last dose of IMP.

CONFIDENTIAL

Colchicine is significantly excreted in urine in healthy subjects. Clearance of Colchicine is decreased in patients with impaired renal function, therefore patients with a creatinine clearance (eGFR) below 30 mL/min will be excluded from the study.
Grapefruit juice may increase plasma levels of Colchicine. Grapefruit juice should therefore not be taken together with Colchicine.

3.3.4. Safety Profile of Rivaroxaban

Patients receiving Rivaroxaban do not require regular monitoring at the recommended dose of 10mg per day. Rivaroxaban is highly protein-bound. Participants randomised to the drug trial will have a blood test for platelet count, activated partial prothrombin time, prothrombin time and bleeding time.

Rivaroxaban is an anticoagulant, and as such the most common complication associated with Rivaroxaban treatment is bleeding. Risk factors for bleeding include bacterial endocarditis, underlying congenital or acquired bleeding disorders, vascular retinopathy, thrombocytopenia, recent procedure/surgery, stroke, neuraxial procedures, uncontrolled hypertension, renal impairment (patients with an eGFR below 30mL/min will be excluded from the study), recent major bleeding, concomitant use of other drugs that affect haemostasis, and advanced age. Clinical evaluation is necessary for any unexplained decrease in haemoglobin or blood pressure.
Other complications associated with central nervous system, skin, gastrointestinal and neuromuscular systems are rare with frequencies ranging from 1 to 3%:

A small increased risk of thrombosis is present with premature cessation of Rivaroxaban so, when discontinuing the drug for reasons besides pathological bleeding or completing the course of therapy, the addition of alternative anticoagulant therapy should merit consideration by the treating physician.

Patients on this arm of the trial will be informed to seek help if they experience unexplained weight loss, feeling faint or high blood pressure. The participants would be able to contact the trial team for advice who will sign post them to the nearest emergency department if urgent medical attention is required.

In addition, reproductive toxicity and embryo-foetal toxicity has been shown in animal studies of Rivaroxaban which is contraindicated in pregnant females. All participants of child-bearing potential enrolled in STIMULATE-ICP will be required to use acceptable effective method of contraception (see section 7.2 and Appendix II for definitions of acceptable effective contraceptive methods) and females of childbearing potential will undertake a pregnancy test prior to enrolment.

Section 6.8 provides full detail on drug interactions, specific contraindications and mitigations related to the trial IMPs.

Appendix I provides a summary of risks associated with the drugs used in the trial and management of the risks. The full list of adverse reactions associated with the above-mentioned IMPs are provided in the appropriate SmPCs.

CONFIDENTIAL

CONFIDENTIAL

4. OBJECTIVES AND OUTCOME MEASURES/ENDPOINTS

4.1. Primary Objectives

The overall primary objective is to evaluate the effect of "integrated care" with combinations of multi-organ MRI (Coverscan™) and clinical decision support, and digitally enabled community rehabilitation (Living with COVID Recovery™) versus "usual care" (Usual Investigations; and Your COVID Recovery™ self-management website) on Fatigue Assessment Score (FAS) at 12 weeks from the enrolment date.

4.2. Secondary Objectives

The secondary objectives include:

1. Clinical efficacy of the individual components of the integrated care pathway, including potential therapies.
2. Effect of the ICP on mean measured health related quality of life, mental health (including depression), work and social adjustment, physical function, organ impairment and healthcare utilisation through patient reported outcomes.

4.3. Primary Outcome Measure

Fatigue is the dominant symptom in 60% of patients and Long COVID clinics are being commissioned to provide rehabilitation for 12 weeks or less, therefore a 12-week outcome is a pragmatic primary outcome measure.

The FAS (Fatigue Assessment scale) is a 10-item, validated questionnaire in patients with chronic diseases. Therefore, it is a pragmatic primary end point for the STIMULATE-ICP trial. Five questions reflect physical fatigue and five questions (questions 3 and 6-9) reflect mental fatigue. The total score ranges from 10 to 50.

4.4. Secondary Outcome Measures

Secondary outcomes will include:

1. Health related quality of life (EQ-5D-5L)
2. Mental health (GAD-7)
3. Depression (PHQ-9)
4. Medical Research Council Dyspnoea Score
5. Perceived Deficit Questionnaire (PDQ-5)
6. Work and Social Adjustment Scale (WSAS) (Question 4 from Productivity Cost Questionnaire (iPCQ) for absenteeism and Question 8 from iPCQ for presenteeism added)
7. Short Form Questionnaire (SF-12)
8. Cognitive Failure Questionnaire (CFQ) if a patient scores 3 or more on PDQ5 (patients receive an email to complete this questionnaire online via a secure password and patient ID number)

9. Organ impairment (as identified by Coverscan™) and healthcare utilisation
10. Cost-effectiveness of ICP
11. Process outcomes for different ICP components

A summary of the objectives and outcome measures are presented in Table 2 below:

Table 2: Summary of the primary and secondary objectives and outcome measures

Objectives	Outcome Measures	Timepoint(s) of evaluation of this outcome measure
**Primary Objective**<br>Evaluation of the effect of "integrated care" with combinations of multi-organ MRI (Coverscan™) and clinical decision support, and digitally enabled community rehabilitation (Living with COVID Recovery™) versus "usual care" (Usual Investigations; and Your COVID Recovery ™ self-management website) on Fatigue Assessment Score at 12 weeks from the date of enrolment in the trial.	FAS	Baseline, 12 and 24 weeks
**Secondary Objectives**<br>The overall clinical efficacy of the individual components of the integrated care pathway including potential therapies.<br><br>1. Effect of the ICP on mean measured health related quality of life, mental health (including depression), work and social adjustment, physical function, organ impairment and healthcare utilisation, through patient reported outcomes.<br>2. To determine if pre-specified sub-groups of patients, either grouped by clinical symptom cluster or imaging findings benefit from either early investigation with Coverscan™, IMPs or rehabilitation.	1. Health related quality of life (EQ-5D-5L)<br>2. Mental health (GAD-7)<br>3. Depression (PHQ-9)<br>4. MRC Dyspnoea Score<br>5. Perceived Deficit Questionnaire (PDQ-5)<br>6. Work and Social Adjustment Scale (WSAS) (Question 4 from Productivity Cost Questionnaire (iPCQ) for absenteeism and Question 8 from iPCQ for presenteeism added)<br>7. Short Form Questionnaire (SF-12)<br>8. Cognitive Failure Questionnaire (CFQ) if a patient scores 3 or more on PDQ5 (patients receive an email to complete this questionnaire online via a secure password and patient ID number)<br>9. Organ impairment (as identified by Coverscan™) and healthcare utilisation<br>10. Cost-effectiveness of ICP<br>11. Process outcomes for different ICP components	Baseline, 12 and 24 weeks

CONFIDENTIAL

~~3.~~	~~1.~~	

4.5. Exploratory Outcome Measures / Endpoints

4.5.1 Core outcome set to characterise the trajectory of Long COVID

There is ongoing research iteratively developing a core outcome data set for clinical and research use in individuals with Long COVID. For example, based on a NIHR-funded research programme (Long COVID Core Outcome Set, LC-COS) which conducted an extensive, international Delphi process in over 1500 patients and health professionals internationally, we have included consensus physiological/clinical, life impact and recovery domains in our trial. We will continue to work with LC-COS in their work to iteratively and pragmatically develop core outcomes for Long COVID as our trial progresses (unpublished communication https://www.comet-initiative.org/Studies/Details/1847).

4.5.2 Discovery science to characterise the pathophysiology of Long COVID

Investigations into the pathophysiological processes that cause long COVID have progressed significantly 2021-2024, but many questions remain unanswered. Gifted blood samples and clinical metadata from STIMULATE-ICP participants, particularly those randomised to drug therapy, present a unique opportunity to interrogate the underlying pathophysiology of Long COVID.

This work will be undertaken as an exploratory sub-study by STIMULATE-ICP investigators analysing participant samples using central laboratories as described in section 9.5.2.

Preliminary proteomics data on a subset of participants, will be used to compare with de-identified imaging metrics to investigate the link between organ impairment found by Coverscan and the proteome.

In the UCLH participant cohort samples where feasible, proteomics assays will be tested for associations with clinical symptoms and laboratory abnormalities. Assay output data will be stratified by symptom duration and severity to characterise the trajectory of Long COVID.
All data will be used to design further experimental work to delineate the mechanistic basis of Long COVID and design interventions for future clinical trials.

We intend to establish a large bioresource of gifted samples and data, to support future work both for the trial investigators and the wider medical community.

5. TRIAL DESIGN

The STIMULATE-ICP trial is a pragmatic, multi-centre, cluster-randomised trial of an integrated care pathway with a nested, Phase III, open label, adaptive platform randomised drug trial in individuals with Long COVID.

CONFIDENTIAL

The STIMULATE-ICP trial is testing the hypothesis that

i. an Integrated Care Pathway for Long COVID is more effective than Usual Care at improving the most common symptom of fatigue.
ii. Any one of the trial drugs is more effective than Usual Care at improving the most common symptom of fatigue.

Currently, patients with signs and symptoms of Long COVID undergo usual investigations by their primary care physicians before they are referred to Long COVID clinics where they undergo further investigations and clinical decision support.

5.1. Definitions

STIMULATE-ICP trial is a pragmatic, multi-centre, cluster-randomised trial of an integrated care pathway with a nested, open label, adaptive platform randomised drug trial in individuals with Long COVID.

"Integrated Care" is defined as:

a. Community based multi-organ MRI (Coverscan™) and clinical decision support; and
b. Enhanced community based, digitally enabled, rehabilitation "Living with COVID Recovery™" App

"Usual Care" is defined as:

a. Usual investigations (includes blood tests, ECG, Chest X ray and Exercise Tolerance Test); and
b. Self-management rehabilitation with associated online resources; (Your COVID Recovery™) for rehabilitation; https:\\www.yourcovidrecovery.nhs.uk

In this trial, two integrated care pathway interventions will be cluster randomised at the Primary Care Network (PCN) level, the two innovations are Coverscan™ and Living with COVID Recovery™.
This is a pragmatic randomised clinical trial (RCT) to compare new ICPs to usual care and we need to ensure that any research activities do not impact/change workload for already over-stretched NHS resources, whilst ensuring equity of access to patient groups.

In clinic areas that are not able to provide both Coverscan™ and the Living with COVID Recovery™ app, it will not be possible to randomise PCNs into integrated care pathway clusters. We will identify these sites to participate in the drug study only, widening access for patients and increasing participation in the platform drug study. All participants in these sites will receive all Usual Care in addition to the opportunity to participate in the individually randomised drug trial. In these sites we will only collect data from those enrolled in the drug study.

5.2. Justification for Cluster Randomisation

If using Individual Patient Randomisation (IPR), provision of information for the scan, time to consider, and consent would need to take place at the General Practitioner (GP) level. Given the current strain on services this is not feasible and could potentially increase waiting times for other practice patients, affect number of

CONFIDENTIAL

patients approached and recruitment would be unlikely to achieve sample size required within the trial timeline. Similarly, if IPR is used for Living with COVID Recovery™ at the GP level the same barriers to recruitment occur.

IPR would mean patients have a 50/50 chance of receiving a scan and a 50/50 chance of being allocated to Living with COVID Recovery™. Cluster randomising at the PCN level will have the same allocation of access across the total number of patients referred to the participating clinic so that equipoise exists at the clinic level. Clustering will use demographic and socio-economic data available for each PCN to ensure equity of access across deprivation indexes within GP codes. Clustering at the PCN level allows Coverscan™ and/or Living with COVID Recovery™ to be provided as an uplift to previous care in allocated PCNs. It will become integrated care within that PCN and, as such, individual patient consent will not be sought (access to and collection of Coverscan™ and Living with COVID Recovery™ app data for research purposes will be by individual patient consent on entering into the study).
Cluster randomisation using a two-by-two factorial design will give us 4 groups:

a. Coverscan™ + Living with COVID Recovery™ App.
b. Coverscan™ + Usual Care Self-Management Rehabilitation.
c. No Coverscan™ + Living with COVID Recovery™ App.
d. No Coverscan™+ Usual Care Self-Management Rehabilitation.

Patients within c and d cluster of (No Coverscan™) are receiving basic investigation as standard of care (see section 5.1) with either Integrated care of the digital community-based Living with COVID Recovery™ App (c) or with community based self-management with the online resources (Your COVID Recovery™) which is an NHS supported online resources for patients recovering from COVID-19.

5.3. Design, Implementation and Evaluation of Integrated Care

This trial aims to investigate two components of care:

5.3.1. To Evaluate "Integrated Care" versus "Usual Care"

"Integrated Care" involves:
a) Multi-organ MRI (Coverscan™) and clinical decision support; and/or
b) Enabling a community-based rehabilitation using a digital Application Platform (Living with COVID Recovery™).

"Usual Care" involves:
a) Usual Investigations; and
b) Self- management website with associated online resources (*Your COVID Recovery™)* for rehabilitation (39).

A four-arm, two-by-two factorial, cluster-randomised, controlled pragmatic trial of ICPs for Long COVID which is *adaptive* to local resources and needs, *reactive* to changing pandemic and policy environments and *iterative*, developed upon ongoing results; plus an adaptive platform open label, individual patient

CONFIDENTIAL

randomised clinical trial to evaluate 12 weeks treatment of four potential therapies: H_1/H_2 receptor blockade with Loratadine 10 mg once daily plus Famotidine 40 mg once daily or Colchicine 500 mcg twice daily or Rivaroxaban 10 mg once daily or usual care (4 arms).

Newly referred patients who are eligible will be approached to consent to a **nested, adaptive platform randomised drug trial.** All patients will be sent an invitation letter to the trial with the trial Participant Information Sheet and Consent Form prior to their Long COVID Clinic appointment. The patients may contact the trial team to participate prior to their Long COVID clinic appointment, at the clinic appointment, up to 7-10 days* after their clinic appointment.

Consent will be taken at any of these time points but randomisation to the nested, adaptive platform randomised drug trial will only occur at the clinic appointment or in the 7-10 days after the clinic appointment. No randomisation will occur until eligibility for the nested drug trial has been confirmed by the clinical assessment and patient history. Randomisation will be Individual Patient Randomisation within clusters. Patients who are ineligible or opt not to participate in the drug trial will be asked to consider consent for data collection or data collection plus blood samples and donation to biobank.
For participants wishing to participate in the drug study, enrolment must take place no later than 10 days post clinic appointment. If they also wish to gift blood, samples should be taken at time of enrolment.

For participants wishing to participate for data collection only consent/enrolment may be up to two weeks post clinic appointment. If they also wish to gift blood samples, they may do so at any time up to 12 weeks post enrolment.

*We will not enrol participants for the drug arm after this time frame so as to align the taking of IMP as far as possible with the 12-week clinic pathway and the 12-week follow-up. The 12-week follow-up date is based on date of randomisation for drug trial participants, and date of consent for participants in data only and data and bloods only.

Patients who have previously been referred to a participating Long COVID Clinic and who are eligible will be offered the opportunity to participate in the individually randomised drug trial. They will also be asked if they are willing to gift blood. Data from these participants will not be used in the analysis of the integrated care pathway trial, only the analysis of the drug trial. These patients will be sent an invitation letter to the trial with the trial Participant Information Sheet. They will be asked to contact the Research Team to arrange a study visit if they wish to participate. Randomisation will be individual patient randomisation.

In clinic areas that are not able to provide either Coverscan™ or the Living with COVID Recovery™ app, it will not be possible to randomise PCNs into the integrated care pathway clusters. We will utilise these clinics as centres for the drug trial only. All participants in these clinic areas will receive or will have received the Usual Care treatment pathway only. Newly referred and previous patients will be invited to participate in the drug study. Data from these participants will not be used in the analysis of the integrated care pathway trial, only the analysis of the drug trial. Randomisation will be individual patient randomisation.

Eligible patients will be randomised to one of the following 4 arms:

CONFIDENTIAL

1. Famotidine + Loratadine + usual care
2. Colchicine + usual care
3. Rivaroxaban + usual care
4. Usual care (no drug)

All patients will be assessed at 12 weeks and 24 weeks for FAS.

The initial plan for the study allowed drugs to be added as additional or replacement arms for new patients at predetermined time points during the trial (6 and 12 months). However the plan for interim analysis was removed through oversight consultation due to changes in the total recruitment target.

Through a cluster randomised trial and a nested adaptive drug trial, evaluation of the Integrated Care versus Usual Care will be conducted by applying validated methods for investigating complex interventions and health services.

Figure 2: STIMULATE-ICP Flow Chart *

CONFIDENTIAL

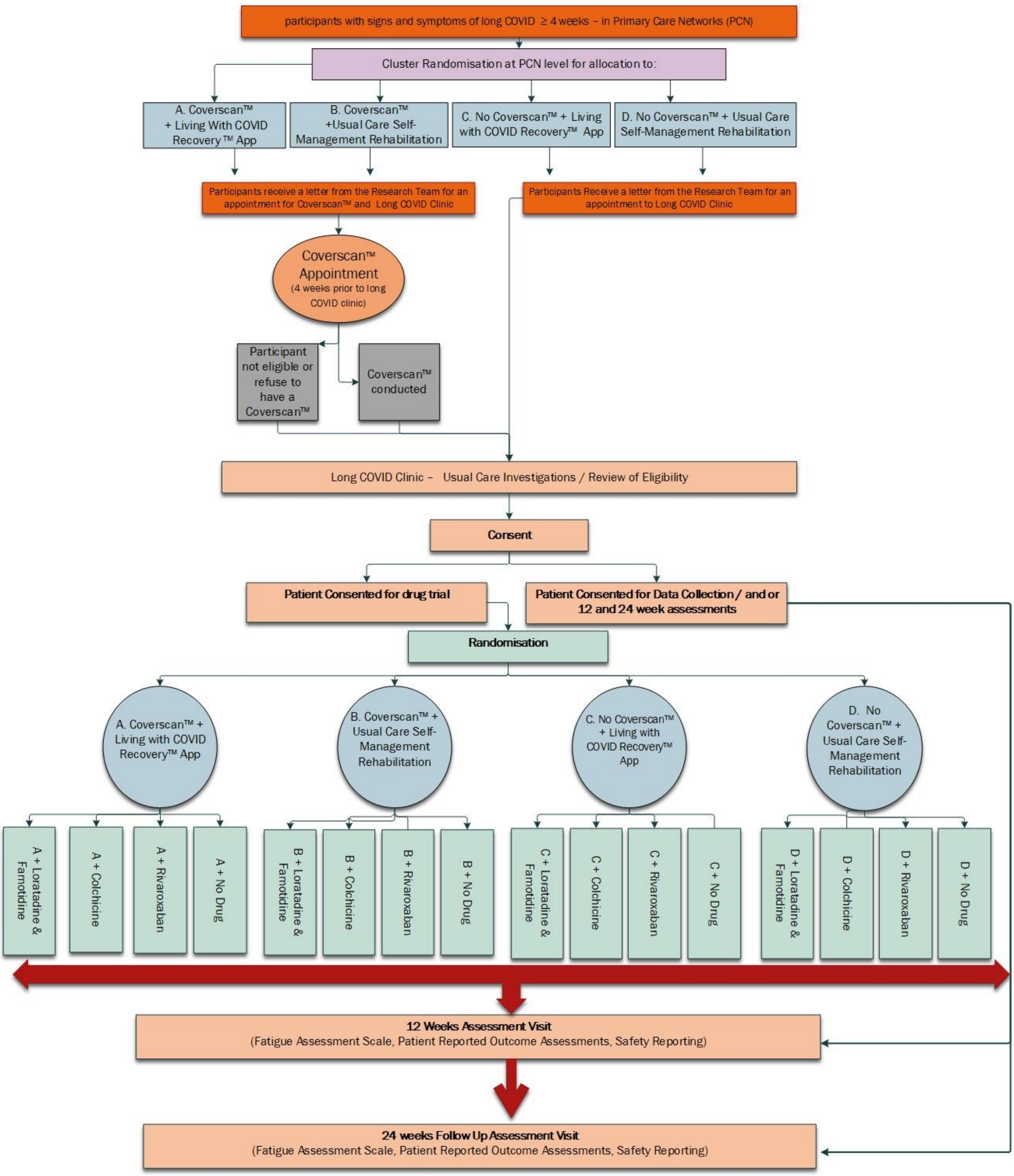
participants with signs and symptoms of long COVID ≥ 4 weeks – in Primary Care Networks (PCN)
Cluster Randomisation at PCN level for allocation to:
A. Coverscan™ + Living With COVID Recovery™ App
B. Coverscan™ +Usual Care Self-Management Rehabilitation
C. No Coverscan™ + Living with COVID Recovery™ App
D. No Coverscan™ + Usual Care Self-Management Rehabilitation
Participants receive a letter from the Research Team for an appointment for Coverscan™ and Long COVID Clinic
Participants Receive a letter from the Research Team for an appointment to Long COVID Clinic
Coverscan™ Appointment (4 weeks prior to long COVID clinic)
Participant not eligible or refuse to have a Coverscan™
Coverscan™ conducted
Long COVID Clinic – Usual Care Investigations / Review of Eligibility
Consent
Patient Consented for drug trial
Patient Consented for Data Collection / and or 12 and 24 week assessments
Randomisation
A. Coverscan™ + Living with COVID Recovery™ App
B. Coverscan™ + Usual Care Self-Management Rehabilitation
C. No Coverscan™ + Living with COVID Recovery™ App
D. No Coverscan™ + Usual Care Self-Management Rehabilitation
A + Loratadine & Famotidine
A + Colchicine
A + Rivaroxaban
A + No Drug
B + Loratadine & Famotidine
B + Colchicine
B + Rivaroxaban
B + No Drug
C + Loratadine & Famotidine
C + Colchicine
C + Rivaroxaban
C + No Drug
D + Loratadine & Famotidine
D + Colchicine
D + Rivaroxaban
D + No Drug
12 Weeks Assessment Visit
(Fatigue Assessment Scale, Patient Reported Outcome Assessments, Safety Reporting)
24 weeks Follow Up Assessment Visit
(Fatigue Assessment Scale, Patient Reported Outcome Assessments, Safety Reporting)

* Patients recruited through a site taking part in the drug study only, or previously referred patients, will not undergo the cluster randomisation element or activities. Their pathway will start at either their first Long COVID Clinic appointment or at their first study appointment (in case of previously referred patients), and they will be randomised to one of the four drug arms.

5.4. Trial setting

STIMULATE-ICP is a multi-centre trial involving a combination of Primary Care Networks and Long COVID Clinics based in 6-10 different geographical areas of England. The trial is managed by LCTU on behalf of the Sponsor. The spread of the sites will be purposeful to encompass geographic and demographically diverse areas. We anticipate 6 -10 sites will be needed dependant on throughput and size of area served.

The LCTU (on behalf of the Sponsor) will ensure all relevant approvals and support are in place prior to activation of study sites.

6. INVESTIGATIONAL MEDICINAL PRODUCTS

The four IMPs to be used for the STIMULATE-ICP trial are licensed for therapeutic use in other conditions and will be used off licence in this study. All aspects of supply, storage and management will be in accordance with standard local policy and practice for prescription medications.

6.1. Name and Description of IMP(s)

6.1.1. Loratadine 10mg tablets taken once daily by mouth

Therapeutic Properties and Licensed indications of Loratadine
Loratadine (10 mg tablets) is indicated for the symptomatic treatment of allergic rhinitis and chronic idiopathic urticaria. In STIMULATE-ICP, Loratadine will be used outside of its licensed indication but within the licensed dose.

6.1.2. Famotidine 40 mg tablet taken once daily by mouth

Therapeutic Properties and Licensed indications of Famotidine
Famotidine is licensed for treatment of duodenal and benign gastric ulcers which have been confirmed by radiological or endoscopic examination, Zollinger-Ellison syndrome, reflux oesophagitis confirmed by endoscopy, including curative treatment of erosion or ulcer associated with reflux oesophagitis. In STIMULATE-ICP, Famotidine will be used outside of its licensed indication but within the licensed dose.

6.1.3. Colchicine 500mcg tablet taken twice daily by mouth

Therapeutic Properties and licensed indications of Colchicine

CONFIDENTIAL

Colchicine is licensed and has been widely used for treatment of acute gout and prophylaxis of gout attack during therapy initiation with allopurinol and uricosuric drugs. In STIMULATE-ICP, Colchicine will be used outside of its licensed indication but within the licensed dose.

6.1.4. Rivaroxaban film coated 10mg tablet taken once daily by mouth

Therapeutic Properties and licensed indications of Rivaroxaban:

Rivaroxaban is licensed for prevention of venous thromboembolism (VTE) in adult patients undergoing elective hip or knee replacement surgery, treatment of deep vein thrombosis, pulmonary embolism (PE) and prevention of recurrent deep vein thrombosis and PE in adults, and stroke prevention in atrial fibrillation. In STIMULATE-ICP, Rivaroxaban will be used outside of its licensed indication but within the licensed prophylaxis dose.

6.2. Formulation, Packaging and labelling of the IMPs

Loratadine, Famotidine, Colchicine and Rivaroxaban will be supplied centrally (details provided in the summary of drug arrangements). IMPs will be supplied to participating sites in their original packaging and repackaging will not be required.
The IMPs are currently exempt from Annex 13 compliant labels following advice from the MHRA.

6.3. Source of IMP, Manufacture and Distribution

All trial IMPs- Loratadine, Famotidine, Colchicine and Rivaroxaban - are licensed by the MHRA and the Sponsor of the trial will procure the IMPs via DOH (Therapeutic Task Force) procurement. All aspects of drug supply and management will be in accordance with regulatory guidelines and standard local policy and practice for prescription medications.

6.4. Storage and Handling of IMP(s) at Site

For most sites sufficient supply of study IMPs will be stored at the site pharmacy or within the clinics at the Investigator site until they are dispensed for participant's use. Details of reordering and resupply of IMPs are detailed in the Summary of Drug Arrangements.
They will be stored as per SmPC. The clinic site/pharmacy will be responsible for the storage and dispensing of the study drug, recording of batch numbers, expiry log and temperature log following local standard procedures and instructions provided in the IMP SmPC.

For clinics without direct access to pharmacy services, a bespoke direct to patient dispensing and delivery service will be provided. Specific study prescriptions will be transferred electronically from clinic-based clinicians to a centralised pharmacy service, (Target Healthcare Ltd under contract with the Sponsor) to provide direct to patient dispensing. Potential participants at such sites will be informed of this process and asked for consent to share relevant data with Target Healthcare Ltd for dispensing/delivery purposes.
Destruction will be performed at study sites by study staff, after verification of returned tablets. For patients attending virtual clinics or if the patient does not return to the clinic in person for the 12-week assessment,

CONFIDENTIAL

the patient is advised to return the remaining tablets to the nearest pharmacy for destruction and accountability is performed over the phone.

There are no special requirements for disposal.

The Trial IMPs supplied to Investigator sites are for trial participants only and are to be used only within the context of the trial.

6.5. Accountability of IMP(s)

Participants randomised to the drug arm will have their allocation entered into their clinical record and their GP will be informed of allocation. Details of the date of prescription, batch number (all prescriptions will consist of 12-week supply of each IMP), and who issued by, will be recorded in the prescription/clinic log spreadsheet held in the Pharmacy file of the ISF.

At the follow-up time point the participants will be asked to bring back any unused medication for counting. If medications are unused, the reasons for non-usage will be documented and the accountability will be assessed. For follow-up over the phone or online, participants will be asked to count any remaining medications and asked for clarification in relation to the return of any remaining medications to their local pharmacy for destruction. Compliance will be noted on the study CRFs and prescription/clinic log spreadsheet.

6.6. Regulatory Status of the IMP(s)

All drugs used in this trial are licenced for their therapeutic indications. Famotidine and Loratadine are available over the counter. None are currently licensed for the treatment of Long COVID by the UK regulator (MHRA).

6.7. Dosage Schedules

- Loratadine: 10 mg tablet orally once a day swallowed whole with water every morning ***PLUS***
- Famotidine: 40 mg tablet orally once a day swallowed with water every morning.
  Or
- Colchicine: 500 mcg tablet orally twice a day swallowed with water every morning and evening.
  Or
- Rivaroxaban: 10 mg tablet orally once a day swallowed with water every morning.

IMPs are taken for 12 weeks/84 days.

6.8. Drug Interactions and Specific Contraindications

The appropriate SmPCs provide the full list of the drug interactions and contraindications for all of the trial IMPs and their degrees of severity. A summary of the list of contraindications and a detailed list of all potential drug-drug interactions are listed in Table 3 below. None of the trial drugs have any documented interaction with any oral or injectable contraceptive agent. Of note, co-prescription of drugs in the classes of serotonin re-uptake inhibitors (SSRIs) and serotonin-norepinephrine re-uptake inhibitors (SNRIs) is

CONFIDENTIAL

permitted in the trial. SSRIs and NSRIs must be used with caution in participants receiving Rivaroxaban as recommended in the SmPC. The risk of increased bleeding, as stated in the SmPC, does not apply to the prophylactic dose of Rivaroxaban 10 mg used. Co-prescription of SSRIs/SNRIs with Rivaroxaban 10 mg does not affect platelet function and thus does not increase the bleeding risk.

Table 3 shows the summary of drug-specific interactions for drugs used in the trial and the associated risk management. For the latest information about drug interactions, British National Formulary (BNF) and SmPCs should be referred to for full details of all contraindications to each drug. The current drug contraindications in the BNF (accessed September 2021) for each of the drugs in this trial are shown below.

CONFIDENTIAL

Table 3: List of Specific Drug Interactions and contraindications/exclusions with the trial IMPs

IMPS	Specific Interactions / exclusions	Risk Level / Class	Risk Management
**Famotidine**	Probenecid	Probenecid inhibits renal tubular secretion of Famotidine and can cause a 50% increase in plasma levels of Famotidine.	Patients to be excluded from drug trial if taking probenecid
	Sucralfate	Sucralfate inhibits absorption of Famotidine.	Patients to be excluded from drug trial if taking Sucralfate
	Posaconazole oral-suspension	Famotidine may reduce the absorption of posaconazole during concomitant use and coadministration should be avoided if possible	Patients to be excluded from drug trial if taking Posaconazole oral -suspension
	Tyrosine Kinase Inhibitors (TKIs) Dasatinib, Erlotinib, Gefitinib, Pazopanib	Famotidine may decrease plasma concentration of TKIs resulting in lower efficacy therefore concomitant use is not recommended	Patients to be excluded from the drug trial if taking these TKIs. For further specific recommendations please refer to the product information of individual TKI medicinal products
**Loratadine**	CNS depressants (such as diphenhydramine, dextromethorphan, pseudoephedrine)	May cause dose-related sedation when administered together	Patients on these drugs will be excluded from the drug trial
	Isocarboxazid<br>Phenelzine<br>Tranylcypromine	These drugs may increase the risk of antimuscarinic adverse effects with Loratadine.	Patients taking these drugs at time of screening will be excluded from the drug trial.

**Colchicine**	CYP3A4 and systemic treatment with azole-antimycotics or HIV protease inhibitors (Aprepitant, Atazanavir, Clarithromycin, Cobicistat, Crizotinib, Darunavir, Diltiazem, Dronedarone, Erythromycin, Fluconazole, Fosamprenavir, Fosamprenavir, Idelalisib, Imatinib, Isavuconazole, Itraconazole, Ketoconazole, Letermovir, Lopinavir, Netupitant, Nilotinib, Posaconazole, ritonavir, Tipranavir, Verapamil, Voriconazole	May cause induction of increased Colchicine levels beyond therapeutic window, increasing the risk of toxicity and agranulocytosis	Patients on these drugs will be excluded from the drug trial
	Amiodarone, Azithromycin, Ciclosporin, lapatinib, Ranolazine, Vemurafenib	P-glycoprotein inhibitors, increase exposure to Colchicine	Patients on these drugs will be excluded from the drug trial
	Atorvastatin, Bezafibrate, Ciprofibrate, Fenofibrate, Fluvastatin, Gemfibrozil, Pravastatin, Rosuvastatin, Simvastatin, Eliglustat	Colchicine increases the risk of rhabdomyolysis	Patients on these drugs will be excluded from the drug trial
	Creatinine clearance <30 mL/minute	Increase exposure to Colchicine	Patients with these conditions will be excluded from the drug trial

Rivaroxaban	Active bleeding; antiphospholipid syndrome (increased risk of recurrent thrombotic events); malignant neoplasms at high risk of bleeding; oesophageal varices; recent brain surgery; recent gastro-intestinal ulcer; recent intracranial haemorrhage; recent ophthalmic surgery; recent spine surgery; vascular aneurysm, uncontrolled severe hypertension. Existing anti-platelet therapy following stroke/myocardial infarction	Significant risk of major bleeding	Patients on these drugs will be excluded from the drug trial
	Acalabrutinib, Aceclofenac, Acenocoumarol, Alprostadil, Alteplase, Argatroban, Aspirin, Axitinib, Beniparin, Benzydamine, Bevacizumab, Bismuth, Bivalirudin, Bosutinib, Bromfenac, Cabozantinib, Cangrelor, Caplacizumab, Celecoxib, Cilostazol, Clopidogrel, Cobimetinib, Dabigatran, Dalteparin, Danaparoid, Dasatinib, Dexkeptorofen, Diclofenac, Dipyridamole, Enoxaparin, Epoprostenol, Eptifibatide, Etodolac, Etoricoxib, Flurbiprofen, Heparin, Ibrutanib, Ibuprofen, Iloprost, Imatinib, Indometacin, Inotersen, ketoprofen, Ketorolac, Levatinib, Mefenamic	Concurrent use with these drugs increases the risk of bleeding	Patients on these drugs will be excluded from the drug trial

	Acid, Meloxicam, Nabumetone, Naproxen, Nicotinic Acid, Nintenanib, Parecoxib, Pazopanib, Phenazone, Phenindione, Piroxicam, Ponatinib, Prasugrel, Regorafenib, Ruxolitinib, Sorafenib, Streptokinase, Sulindac, Sunitinib, Tenecteplase, Tenoxicam, Tiaprofenic Acid, Tecagrelor, Tinzaparin, Tirofiban, Tolfenamic Acid, Trametinib, Traztuzumab Emtansine, Treprostinil, Urokinase, Volanesorsen, Warfarin		
	Amiodarone, Anagrelide, Cobicistat, Darunavir, Dronedarone, Erythromycin, Fosamprenavir, Itraconazole, Ivacaftor, Ketoconazole, Lopinavir, Neratinib, Nevirapine, Olaparib, Osimertinib, Posaconazole, Ritonavir, Tipranavir, Vandetanib, Vemurafenib, Venetoclax, Voriconazole		
	Atazanavir	CyP3A4 inhibitors increase exposure to Rivaroxaban	Patients on these drugs will be excluded from the drug trial
	Severe hepatic impairment	Increase exposure to Rivaroxaban	Patients with these conditions will be excluded from the drug trial
	Creatinine clearance <30mL/minute	Increase exposure to Rivaroxaban	Patients with these conditions will be excluded from the drug trial

6.9. Consideration of Dose Modification

Given the design and short intervention period within this study, dose modification will not be used except in cases where a short period of contraindicated antibiotic use is required. For these cases, a short cessation of the study drug will be accepted and recorded. The participant may restart study drug 48 hours after cessation of antibiotic treatment.

6.10. Concomitant Medications

The participant's medical records will be accessed by the trial team at the Long COVID clinic to confirm eligibility (once the participant has consented), all concomitant medications will be recorded and assessed for contraindications to study drugs. The patient will also be asked to confirm if the list is complete and if they are taking other prescribed medications from either primary or secondary care clinicians. They will also be asked for details of any over the counter medications, herbal supplements, vitamins, minerals homeopathic or "leisure drugs" e.g., cannabis.

7. TRIAL PROCEDURES

7.1. Selection of Participants

Participants will be 18 years or older, with symptoms of Long COVID for a period longer than 4 weeks based on clinician's diagnosis and referred to participating Long COVID clinics. The site-specific trial teams are embedded within the Long COVID clinics or at local recruitment centres, and will identify potential participants from the clinic list of new referrals. All new referrals will be sent the trial information sheet and contact details for the research team in the appointment notification letter prior to attendance at their first appointment. This will give participants time to consider taking part prior to their appointment. In instances where the potential participant has not received information then information will be provided at time of approach or clinic visit. The patient will be given as much time as they feel they need to make a decision regarding participation and may consent on that day if they wish or return at a later time point within the 7-10 days post clinic appointment as per patient choice. Patients at sites taking part in the cluster randomised trial, who do not wish to consent the drug arm but do wish to consent to data may enrol up to 2 weeks post clinic and gift blood samples up to 12 weeks post enrolment.

Previously seen patients with ongoing long COVID symptoms will be invited to take part in the drug study only. Their data will contribute to the drug study findings only and will not be used in the integrated care pathway analysis.

Sites participating in the drug study only may recruit from both newly referred patients and previously seen patients with ongoing long COVID symptoms. Their data will contribute to the drug study findings only and will not be used in the integrated care pathway analysis

7.2. Participant Eligibility Criteria

Participant Inclusion Criteria for ALL Participants

1. Participants capable of giving informed consent

2. Aged 18 years and above

3. Clinical Parameters: persistent signs and symptoms for a period of 4 weeks or longer in duration post-COVID-19 infection (either by test result or symptomology).

4. Able to read or understand English or have a relative/family member able to read/understand English to facilitate participation (essential for patient reported outcome measures at follow-up time points and virtual contact)

5. Not enrolled in any other interventional study where study intervention/activities may affect outcome measures (patients enrolled in purely observational studies can be included)

Additional Participant Inclusion Criteria for the cluster randomised integrated care pathway study

6. Presenting at their first referral to a participating Long COVID clinic pathway.

Additional Participant Inclusion Criteria for the nested, platform randomised drug trial (to be eligible participants must meet **criteria 1 – 5 above** and all those below)

Note: Potential participants with drug-specific contraindications for any arm, including interactions of pre-prescribed essential medication will be consented for data collection but will be excluded from the drug study.

1. Presenting at their first referral to a participating Long COVID clinic pathway or previously referred to a participating Long COVID clinic and still suffering from Long COVID symptoms

1. Females of childbearing potential (see definition below) must be willing to use at least an acceptable effective method of contraception during the treatment with IMP and for a further 30 days after the last dose (34).

   Such methods include:

   a. combined (oestrogen and progestogen containing) hormonal contraception:

      I. oral
      II. intravaginal
      III. transdermal

   b. progestogen-only hormonal contraception

      I. oral
      II. injectable
      III. implantable

   c. intrauterine device (IUD)

   d. intrauterine hormone-releasing system (IUS)

CONFIDENTIAL

e. bilateral tubal occlusion

f. vasectomised partner

g. male or female condom with spermicide

h. cap, diaphragm or sponge with spermicide i. sexual abstinence; only true abstinence is acceptable i.e. when this is in line with the preferred and usual lifestyle of the participant (periodic abstinence, declaration of abstinence during exposure to IMP and withdrawal are not accepted methods of contraception).

Definition of females of childbearing potential:

For the purpose of this trial, a female is considered of childbearing potential (i.e., fertile) following menarche and until becoming post-menopausal unless permanently sterile. Permanent sterilisation methods include hysterectomy, bilateral salpingectomy, and bilateral oophorectomy.

A post-menopausal state is defined as no menses for 12 months without alternative medical cause.

3. Male participants must be willing to use condom during IMP treatment to protect their female partner becoming pregnant and for a further 90 days after the last dose.

4. Patients on pre-existing treatments for the same drug classes or contraindicated drugs MUST undergo a 7-day washout period before being randomised.

(Patients will be assessed and, if safe to do so, asked if they would be willing to undergo a washout period of at least 7 days after consent but before being randomised and exclude that medication until 7 days after last dose of IMP.)

Participant Exclusion Criteria for ALL Participants

1. Previously hospitalised for COVID-19 infection.

Additional Participant Exclusion Criteria for Research Blood Sampling

3. Participants with any known blood borne viruses (BBV's) including Hepatitis C, Hepatitis B and/or HIV

4. Participants with any condition that may severely prolong clotting times

Additional Participant Exclusion criteria for nested, adaptive randomised drug trial

5. Females who are pregnant, planning pregnancy or breastfeeding

6. Known hypersensitivity to any of the study drugs or their excipients

7. Currently taking any of the following drugs:

Probenecid, Sucralfate, Posaconazole oral-suspension, Dasatinib, erlotinib, gefitinib, pazopanib (for further specific recommendations please refer to product information of individual TKIs product information,

CONFIDENTIAL

Isocarboxazid, Phenylzine, Tranylcypromine of any other CNS depressant (such as diphenhydramine, dextromethorphan, or pseudoephedrine) *(Contraindications to Famotidine/Loratadine)*

Amiodarone, Aprepitant, Atazanavir, Atorvastatin, Azithromycin, Bezafibrate, Ciclosporin, Ciprofibrate, Clarithromycin, Cobicistat, Croztibib, Darunavir, Diltiazem, Dronedarone, Eliglustat, Erythromycin, Fenobibrate, Fluconazole, Fluvastatin, Fosamprenavir, Gemfibrozil, Idelalisib, Imatinib, Isavuconazole, Itraconazole, Ketoconazole, Letermovir, Lopinavir, Netupitant, Nilotinib, Posaconazole, Pravastatin, Ranolazine, Ritonavir, Rosuvastatin, Simvastatin, Tipranavir, Velpatasvir, Vemurafenib, Venetoclax, Verapamil, Voriconazole *(Contraindications to Colchicine)*

Acalabrutinib, Aceclofenac, Acenocoumarol, Alprostadil, Alteplase, Argatroban, Aspirin, Axitinib, Beniparin, Benzydamine, Bevacizumab, Bismuth, Bivalirudin, Bosutinib, Bromfenac, Cabozantinib, Cangrelor, Caplacizumab, Celecoxib, Cilostazol, Clopidogrel, Cobimetinib, Dabigatran, Dalteparin, Danaparoid, Dasatinib, Dexkeptorofen, Diclofenac, Dipyridamole, Enoxaparin, Epoprostenol, Eptifibatide, Etodolac, Etoricoxib, Flurbiprofen, Heparin, Ibrutanib, Ibuprofen, Iloprost, Imatinib, Indomethacin, Inotersen, Ketoprofen, Ketorolac, Levatinib, Mefenamic acid, Meloxicam, Nabumetone, Naproxen, Nicotinic acid, Nintenanib, Parecoxib, Pazopanib, Phenazone, Phenindione, Piroxicam, Ponatinib, Prasugrel, Regorafenib, Ruxolitinib, Sorafenib, Streptokinase, Sulindac, Sunitinib, Tenecteplase, Tenoxicam, Tiaprofenic acid, Ticagrelor, Tinzaparin, Tirofiban, Tolfenamic acid, Trametinib, Traztuzumab emtansine, Treprostinil, Urokinase, Volanesorsen, Warfarin *(Contraindications to Rivaroxaban)*

8. Renal failure/insufficiency (eGFR <30 mL/minute) on the basis of blood investigations within the last 6 months and clinical assessment

9. Severe liver dysfunction on the basis of blood investigations within the last 6 months (liver function and in some cases coagulation) and clinical assessment

10. Patients with blood dyscrasias

7.3. Participant Identification

The participants are identified by referral to a participating Long COVID clinic. All patients scheduled for their first clinic appointment are eligible for participation within the study.

All newly referred patients will be sent a clinic appointment letter which will also contain a study invitation letter and a study information leaflet. The invitation will ask patients to consider taking part and provide contact details of the site study team. The patient can contact the team prior to their clinic appointment and ask any questions they may have and, if willing, make an appointment for eligibility assessment and consent prior to clinic. They may consent at the time of clinic or in the 7 -10 days after their clinic appointment for enrolment in the drug arm, or up to 2 weeks after their clinic appointment for data only. Only consent for data collection and blood collection can be taken prior to clinic. Randomisation into the drug study can only occur after confirmation of eligibility by the clinic team and the trial team.

Patients who have previously been seen at a participating Long COVID Clinic and who are eligible will be offered the opportunity to participate in the individually randomised drug trial for Data Collection and Drug. Clinic staff

CONFIDENTIAL

will identify previously seen patients from the clinic lists and will screen patients for potential eligibility and disseminate invites. These patients will be sent an invitation letter (or site-specific communication) to the trial with the trial Participant Information Sheet.

In the information sheet, all potential participants will be invited to call the research team if they are interested in participating and offered the option of receiving a paper copy of the Baseline CRF to begin completing at home (pages suitable for self-completion will be listed) the week prior to clinic and bring with them to their appointment. Consent will be confirmed formally at the clinic appointment. The CRF does not contain identifiable information. Participants that complete the CRF but then decided not to consent to the study will have their CRF destroyed at site, or they can destroy it at home. At clinic, the research team will review the CRF in full and complete those measures requiring assessment and/or assistance.

Potential participants will also be given information about the study by their treating physician at the Long COVID clinic appointment.

Eligibility for the drug arm must be confirmed by the PI or suitably delegated clinician as per the delegation log, following results of clinical assessments, to confirm the participant meets all the inclusion criteria and none of the exclusion criteria. Eligibility for data collection only (and bloods if applicable) can be confirmed by a suitably delegated member of the research team as per the delegation log.

7.4. Recruitment of Trial Participants

It is estimated that there are potentially 30,000 adults with Long COVID across our initial 6 areas. However, referrals may be limited by clinic capacity and GP referral trends. We estimated that it was feasible to consent 4520 patients into the drug study through the referral of these patients to the Long COVID clinics. Total participant recruitment was expected to be more than this to accommodate those that are ineligible for the drug arm and those that choose not to participate in the drug arm to consent to data collection and blood sample collection.

The recruitment figures to end of May 2023, and the decision to partially decouple the cluster and drug trials through the inclusion of drug only sites, means that a revision of the planned recruitment rate is required. We estimate that it is feasible to consent 1130 recruited participants, not previously seen by a Long Covid Clinic, into the cluster trial from the cluster trial sites. Recruitment into the drug trial from cluster sites and from drug only sites is expected to be least 300 per arm.

PCNs within the areas served by the individual clinic will be cluster randomised to receive as usual care:

a. Coverscan™ + Living with COVID Recovery™ App.
b. Coverscan™ + Usual Care Self-Management Rehabilitation.
c. No Coverscan™ + Living with COVID Recovery™ App.
d. No Coverscan™+ Usual Care Self-Management Rehabilitation.

Site-specific research practitioners embedded in the Long COVID Clinics will identify new referrals who have not been previously hospitalised with COVID-19 and, if from within a PCN with Coverscan™ as usual care will arrange a scan appointment circa 4 weeks prior to the first Long COVID clinic appointment (to ensure scan report available at time of clinic assessment). This appointment is usual care within this PCN and will follow the same pathway as other clinical appointments.

Similarly, patients referred from PCNs allocated to "Living with COVID recovery™" app will be identified and referred to the physiotherapist/s assigned to delivery of this app, who will contact participants directly (as usual care). An appointment with the participant may be required prior to the Long COVID clinic to discuss the app wherever possible or within 7 -10 days of the first Long COVID clinic appointment.

In clinic areas that are not able to provide both Coverscan™ and the Living with COVID Recovery™ app, it will not be possible to randomise PCNs into the integrated care pathway clusters. All participants in these clinic areas will receive or will have received the usual care treatment pathway only, participants from these areas will be given the opportunity to participate in the individually randomised drug trial and data collection. They will also be asked to consider gifting blood samples.

Some patients interested in participating in the trial may have "brain fog" and, therefore, may require more time to consider taking part. We anticipate a gap of around 4-6 weeks between referral by the GP and patient's attendance to the appointment at the Long COVID clinic. Wherever possible, patients will be sent details of the study (the options to participate in all or part of the study) with the first appointment letter. This will include the study details, directions to the study website and contact details of both the research team based at their clinic and LCTU. If they wish to know more about the study prior to their first clinic appointment, then they can contact the team. We will also include details of any participation timeline, i.e., eligibility cannot be determined for the drug study prior to the first appointment, consent for the drug study should be no later than 7-10 days after the first appointment, consent for data only should be no later than 2 weeks after their clinic appointment with the option to gift blood up to 12 weeks post appointment. In instances where the potential participant has not received information, then information will be provided at time of approach or clinic visit. Previously see patients will receive an invitation letter and PIS outlining all sources of information and contact. The patient will be given as much time as they feel they need to make a decision regarding participation and may consent on that day if they wish, or return at a later time point within the required timeframes as per patient choice.

We will explain the value of data collection from all participants for the cluster trial and contribution to the blood sample collection in particular, to engage with those participants who may not be eligible for the drug trial but can make a valuable contribution to the study and its outcomes.

For patients where English may not be their first language, the information will contain details of how they can take part assisted by an English-speaking relative/friend.

7.4.1. Informed Consent

The Principal Investigator (PI) retains overall responsibility for the conduct of research at their site. They must ensure that any person delegated responsibility for any study activities is duly authorised, trained, and competent in the principles of Good Clinical Practice (GCP) and the Declaration of Helsinki. If delegation of consent is acceptable, then details should be provided.

The participant remains free to withdraw at any time from the trial without giving reasons and without prejudicing his/her further treatment and must be provided with a contact point where he/she may obtain further information about the trial. Data collected up to the point of withdrawal will be kept and used in the trial for

CONFIDENTIAL

analysis as detailed in the patient information sheet. However no further data will be collected if the participant requests this. Any intention to utilise such data shall be included and outlined in the consent form. It will be made clear that, if data has been anonymised and aggregated, it will not be possible to identify individual data for withdrawal. It will also not be possible to identify any one person from such data. Samples in storage at the point of withdrawal will be destroyed if the participants requests this.

Participants will be notified that their identifiable data (name, date of birth, NHS number, and contact details) will be shared with LCTU, and central pharmacy if applicable, to enable contact for follow-up within the study, and for well-being reporting if needed.

This trial will only recruit participants who are able to consent. If a participant later becomes incapacitated, the participant shall be withdrawn from the trial and no further data will be collected or any trial investigation or assessments conducted. It is not expected that this would be disease progression within this group. Assessments relating to safety issues arising from participation will continue until the trial ends but will be the remit of the patient`s clinician.

Participants who understand English but who have difficulty reading/writing English, who are visually impaired or who are physically unable to sign a document themselves should not be excluded. For these participants, a member of the research team may sign a Witnessed Consent Form on their behalf, countersigned by a witness. The witness may be a member of the participants family or another member of the research team in addition to the member of staff obtaining consent.

Participants allocated to the cluster with the Living with COVID Recovery™ digital app will be informed that data related to their symptoms, physical and mental health, heart rate and physical activities - through the use of the app - will be used to inform their care plan by the Multidisciplinary Team at the Long COVID clinics. The app forms part of their usual care and is part of the clinical record: data from consented patients will be used by the trial team to assess the use of the app (level of engagement) and use of content-specific areas within the app.

7.4.2. Informed Consent Procedure

Patients should be given sufficient time after receiving the trial Participant Information Sheet and Informed Consent Form to consider and discuss participation in the trial with friends and family (adequate time). The person taking consent will be GCP trained, suitably qualified and experienced, and will have been delegated this duty by the CI/ PI on the study delegation log.

Previously referred patients will be asked to contact the study team to make an appointment if they wish to consent.

We anticipate that newly referred patients will receive study information in their Long COVID clinic appointment letter so will have had ample time to read this, go to the study website or contact the research team for information. Patients may consent at the time of clinic or if they wish to have more time to consider in the required timeframe. In instances where the potential participant has not received information prior to clinic then information will be provided at time of clinic visit. The patient will be given as much time as they feel they need to make a decision regarding participation and may consent on that day if they wish. For newly referred patients, consent for the drug arm should be no later than 7-10 days after the first appointment, consent for data only

CONFIDENTIAL

should be no later than 2 weeks after their clinic appointment, with the option to gift blood up to 12 weeks post appointment as per patient choice.

All the data collected has to cover the same treatment pathway and trial assessments timeline as other participants so newly referred patients in integrated care sites may not enter the drug study after this timepoint to ensure that their 12-week assessment is current to their clinic pathway and the administration of IMP (if allocated to that arm). This is also relevant to the "before" and "after" data assessments in the data collection.

At the time of the Long COVID Clinic appointment, a member of the research team will be present at clinic (if face to face) for queries and information prior to the participant signing the consent form. For virtual clinics, patients will be initially screened over the telephone and then asked to attend in person for full eligibility checking if they wish to participate in the drug study or give research and biobank bloods. Participants on contraindicated medications requiring a 7-day washout period on screening would be consented for the drug arm at clinic but not randomised until after the washout period following full eligibility checking. It is not possible to randomise newly referred patients to the nested drug trial until after the first clinic assessment and confirmation of eligibility.

In integrated care pathway clinics patients with known contraindications to the drug study or those that do not wish to participate in the drug study may be consented to data collection or data collection + blood samples at that visit (if at a site taking part in the cluster randomised trial).

Previously seen patients will require an updated eligibility check by a clinician before consent, and eligibility will **not** be taken from medical notes or clinic records alone.

Trial sites will use a proactive approach (in keeping with COVID restrictions, vulnerability/fatigue, and equity of access for this group) to contact those patients who have not already expressed an opinion or been asked to participate, to ascertain interest in taking part.

Newly referred patients in integrated care clinic sites who are too fatigued to attend in person or who are attending a virtual clinic will be given the opportunity to take part in data collection only by verbal consent. For patients who express this preference, a telephone consent form (which only the research team at the participating site study will complete) and a copy will be sent by post or email to the patient for their reference. This will also include another copy or e-copy of the participant information sheet for those who have not received one, or who have asked for another copy for their records.

After having time to consider, a member of the site study team will contact them to ask if they would like to participate. For telephone consent, the staff member will read out each consent statement and ask the patient if they agree to each one. The staff member will initial and date all the statements. This consent form will be counter signed by the PI or another member of the research team. This will be considered full consent given by the participant. They will not be asked to complete and/or return a separate consent form. Copies of this consent will be treated as other consents with a copy sent to the patient (either postal or email as per patient choice), a copy in the site file and a copy in the medical records.

Consent for blood samples and the drug study will not be available in these formats and will continue to be face-to-face consent only for all participants.

CONFIDENTIAL

For those able to attend and sign in person, a copy of the signed, informed consent form will be given to the participant. Participants who understand English but who have difficulty reading/writing English, who are visually impaired or who are physically unable to sign a document themselves may have a member of the research team sign a Witnessed Consent Form on their behalf, countersigned by a witness. The witness may be a member of the participants family or another member of the research team in addition to the member of staff obtaining consent. The staff member taking consent will read out each consent statement and ask the patient if they agree to each one. The original signed form will be retained in the trial file at site, and a copy placed in the medical notes.

The study will use a suite of consent forms to reflect the different options available to participants. The eligibility check form will enable staff to identify which consent form is suitable for each participant.

Consent form options:
1. Data collection (face to face)
2. Data collection and research blood sample donation (face to face)
3. Data collection and drug arm (face to face)
4. Data collection, drug arm and research blood sample donation (face to face)
5. Data collection (telephone)
6. Data collection (face to face - witnessed consent)
7. Data collection and research blood sample donation (face to face - witnessed consent)
8. Data collection and drug arm (face to face - witnessed consent)
9. Data collection, drug arm and research blood sample donation (face to face - witnessed consent)

The PIS and consent form will be reviewed and updated as necessary throughout the trial (e.g., where new safety information becomes available) and participants will be re-consented as appropriate.

As part of the consent procedure, all participants in all sites who have consented for bloods will be asked to confirm if they would be willing to be contacted at the 12- or 24-week time point for further blood sampling if early analysis suggests emerging evidence of utility.

Consent for further future contact is also asked for, to enable sharing of information to participate in other STIMULATE-ICP activities generated by early findings and other studies in relation to COVID-19 and/or Long COVID. Contact will be for up to one year after study closure unless at time of contact participant declined to be contacted again.

8. REGISTRATION / RANDOMISATION PROCEDURES

8.1. Patient Registration

All patients consenting to the trial will be registered on the trial enrolment log and given a trial ID number which is a 6-digit number. The first two digits will refer to the Long COVID centre and the last four digits will be unique to the participant. Each centre will be pre-allocated with Participant ID numbers on the trial database. Records of each enrolment log will be kept in the Investigator Site File and a copy will be kept in the Trial Master File (TMF).

CONFIDENTIAL

Enrolment date is the date of randomisation for participants in the drug arms of the trial, or the date of consent for participants in data collection only and data collection + bloods.

Participants in integrated care sites who consent for the drug study but subsequently fail further eligibility tests (e.g., pregnancy testing, repeat blood testing, incomplete washout period) will be ineligible for the drug study and will not be randomised; date of enrolment will be taken as date of original consent. These patients will be in included in data or data + blood arms as per patient choice.
Previously seen patients who fail eligibility will be thanked for their interest but will not be asked to continue for data collection.
Newly referred patients in drug only and drug and blood only sites who fail eligibility will not be asked to continue for data collection.

8.2. Cluster Randomisation for Usual Care service uplift

Up to 226 Primary Care Network (PCN) sites will be allocated using blocked stratified randomisation using block size of 4 into one of the four 2x2 factorial allocations labelled as a, b, c, d (see below) in ratio 1:1:1:1:

a. Coverscan™ + Living with COVID Recovery™ App.
b. Coverscan™ + Usual Care Self-Management Rehabilitation.
c. No Coverscan™ + Living with COVID Recovery™ App.
d. No Coverscan™+ Usual Care Self- Management Rehabilitation.

Randomisation lists will be created by the study statisticians based at UCL and LCTU.
To form the strata, PCNs linked to each clinic site will be ordered first by descending size of patient population and then defined by a single cut off value as large PCNs or small PCNs. Then large PCNs will be sorted by descending deprivation and small PCNs by ascending deprivation and defined by a single cut off value as more or less deprived. Within the four strata of PCNs: large, less deprived; large, more deprived; small, more deprived; and small, less deprived, treatment pathways will be allocated using randomised blocks of size 4. This will reduce bias and ensure that there is balance across the pathways by deprivation and population size within each clinic site.

8.3. Drug Trial Randomisation

Participant randomisation will be undertaken using an online system provided by the company Sealed Envelope™.

Integrated care sites
Coordinated registration and allocation of participant trial numbers will be required to enrol participants. Blocked randomisation will be used to allocate study participants to one of the treatment groups, stratified by clinic, gender and treatment pathway for site taking part in the cluster randomised study and drug study.

To allow the inclusion of participants who have previously been seen at a clinic into the drug trial, a further stratification factor (previously seen at a clinic or not) will be added to the randomisation portal. To allow a single randomisation process to be used, a treatment pathway is needed, and those previously seen at a clinic will nominally be assigned to the no Coverscan™+ Usual Care Self- Management Rehabilitation treatment pathway, before being randomised to a drug arm. The randomisation will be implemented through the online randomisation portal, supplied by Sealed Envelope™ Ltd.

Drug study only sites
Blocked randomisation will also be used to allocate study participants to one of the treatment groups, stratified by clinic and gender for clinic sites that take part in the drug trial only. To allow the inclusion of participants who have previously been seen at a clinic into the drug trial, a further stratification factor (previously seen at a clinic or not) will be added to the randomisation portal. The randomisation will be implemented through the online randomisation portal, supplied by Sealed Envelope™ Ltd.

Participants will be assigned to treatment groups through consecutive allocation of subject numbers, and the use of a Trial Subject Enrolment Log which will be stored on the Sealed Envelope™ system.

8.4. Blinding

There will be no blinding for the drug arm of this study: this is an open label study. As this is a pragmatic trial with currently unconfirmed drugs being introduced later, for practical reasons it has not been feasible to pursue encapsulation or placebos for multiple drugs. The majority of the key outcomes are patient-reported outcomes and so there is little potential for outcome assessors to introduce bias by knowing cluster or drug allocation. The number of interim analyses originally planned in the trials made blinding of the statisticians impractical.

9. TRIAL ASSESSMENTS

9.1. Eligibility Assessments

Newly referred patients who have received the study information prior to clinic attendance may contact the study team to confirm potential eligibility prior to their appointment. If wishing to take part for data collection only (integrated care sites only), they will have the option to consent in person at clinic or to consent over the telephone (see 7.4.2). All patients will be screened using the eligibility criteria.

For patients who indicate that they want to take part for data collection plus blood sampling/biobank (integrated care sites only), consent will be face-to-face.

For patients who indicate that they want to take part in the drug arm or drug arm plus blood sampling and biobank, they will be screened for any contraindication for the drug arm (and blood sampling if appliable) to establish eligibility. For the drug arm, all eligibility criteria will be assessed using blood tests done at the time of referral, GP referral letter content, presenting symptomology, patient discussion and Long COVID clinic notes. If any concerns are noted at clinic/eligibility appointment, or the relevant blood results are outside the timeline for screening acceptability (potentially in previously seen patients)(see below for timelines), then further blood tests may be ordered, and the research team will assess these results to confirm eligibility. For newly referred patients in integrated care sites, eligibility must be confirmed no later than 10 days post-clinic appointment. If found to be ineligible, then patients will be excluded from the drug arm and invited to continue for data collection and bloods only

The following procedures/test results must be available to confirm eligibility for the nested drug trial:

- Participant full medical history (at Long COVID or Research Clinic visit)

- Concomitant medication review (at Long COVID or Research Clinic visit)
- Physical Examination (at Long COVID or Research Clinic visit)
- Urine pregnancy test within 7 days prior to the first dose of IMP (in women of childbearing potential)
- eGFR (within 6 months)
- Liver function test (within 6 months)
- FBC (within 6 months)
- Coagulation screening (within 6 months) *

All participants, once consented, regardless of participatory arm, will be invited to complete assessments at baseline, 12 and 24 weeks.

* Coagulation screening in the prior 6 months is not needed in all patients but only as follows:

1. If FBC and LFTs (bilirubin and albumin) are normal in last 6 months and clinical assessment does not suggest liver impairment, then coagulation is not required.

2. If FBC is normal, but LFTs shows severe impairment (i.e., bilirubin >50 micromol/L or albumin <28 g/L, as per Child-Pugh criteria), then please check coagulation (including PT INR). If the individual meets criteria for severe liver impairment (i.e., PT INR>2.3), then they should be excluded from the study).

3. If LFTs are normal, but FBC is abnormal (whether in terms of platelets, red blood cells, or white blood cells), then the following conditions denote exclusion from the study:

    - congenital or acquired bleeding disorders
    - uncontrolled severe arterial hypertension
    - other gastrointestinal disease without active ulceration that can potentially lead to bleeding complications (e.g., inflammatory bowel disease, oesophagitis, gastritis and gastroesophageal reflux disease)
    - vascular retinopathy
    - bronchiectasis or history of pulmonary bleeding

If these exclusion criteria are not met, but there is an abnormality in the FBC, then this should be managed as per local clinical practice guidelines. If an individual still meets inclusion criteria, then they can be included in the trial. A guidance document consisting of a flow chart will be provided to sites.

9.2. Baseline Visit

At the baseline visit, the patient will be given a copy of the CRF questionnaire to complete at the clinic or to take home and to be contacted by a member of the trial team the following day to complete over the phone. Some patients may have been provided with the CRF questionnaire to complete prior to the baseline visit, this will be collected by the trial team and reviewed with the patient at the visit.
During the visit the following will be recorded:

A. Patient's demographics

B. Patient's full medical history from January 2020 (when COVID was recognised as circulating in the UK) including comorbidities and all COVID-related illnesses, treatments, and vaccinations.
C. Patient's history of concomitant medication including current, pre- and post-COVID-19 and over the counter medications
D. Physical examination including weight, height, temperature, resting pulse, and blood pressure

Laboratory Tests:

Routine clinical blood tests performed by the General Practice doctors, as a result of clinic assessment at the point of referral, and any tests reordered to confirm eligibility will be collected as part of trial data. Blood tests will differ in different clinics based on differences in usual care and capacity and capability to provide within that service. Patients who consent for research blood sampling and for further research will have blood taken. For participants also consenting to the drug arm, these bloods will be taken at baseline first visit; if they do not wish to take part in the drug arm, they will be able to give bloods at a separate visit any time up to 12 weeks after their first appointment. Blood tests outlined below will be undertaken at central laboratories using a batched analysis approach at a later time point. It is not expected that the results of these bloods will directly impact on individual patient usual care during the 12-week clinic pathway.

Approximately 60mL of blood will be taken at in sites that have capacity and capability for further research (translational sub-studies) and biobanking (see section 9.5 for details); In the London site approximately 500 participants will be asked if they would be willing to give further blood (30mL) at 12 weeks for comparative analysis.

As part of the consent procedure, all participants in all sites who have consented for bloods will be asked to confirm if they would be willing to be contacted at the 12- or 24-week time point for further blood sampling if early analysis suggests emerging evidence of utility.

Functional Tests:

Standard investigations differ from clinic to clinic and the study will not require a standardised testing strategy as that would impact on its aim of assessing usual care; but it will collect any and all data, related to individual consented participants tests and investigations (either ordered by the GP at time of referral or ordered as a result of Long COVID clinic appointment). This will include all data related to Coverscan™, which will be provided as an uplift to usual care in randomised areas.

Examples of functional tests in some clinics are:

a. Chest X Ray
b. High Resolution Computerised Tomography (CT) scan of the Chest
c. CT Pulmonary Angiogram
d. Pulmonary Function Test
e. 6-minute walk test
f. 1-minute sit to stand test
g. Functional Exhaled Nitric Oxide (FeNO) test
h. Echocardiogram (ECHO)
i. Electrocardiogram (ECG) if the patient had cardiac symptoms

CONFIDENTIAL

j. Holter monitor of the heart
k. Cardiovascular Magnetic Resonance Scan (CMR)
l. Stress Electrocardiogram
m. Magnetic Resonance Imaging (MRI) scan of Brain
n. Tilt Table Test
o. Coverscan™

Baseline process

The following process applies to all participants consenting for inclusion in the trial. The Research team will action the following:

a) Consent form: Ensure 4 copies of the consent form have been obtained; one copy retained in the patient's medical record, one copy given to the patient, one copy is stored in the Investigator Site File (ISF) and one copy sent to LCTU.

b) Study number allocation: All participants will be allocated a study number via Sealed Envelope. The date of entry into Sealed Envelope and the generation of a study number will be day 1 for participants.

c) Randomisation: The system will then ask the researcher if the participant is willing to enter into the drug study. Once confirmed, the system will then randomise the participant to either drug or usual care (randomisations for new patients in integrated care sites will use cluster (treatment pathway) allocation as an additional strata to site and gender; randomisations for previously seen patients in integrated care sites will have a nominal cluster allocation to the usual care treatment pathway as an additional strata to site and gender; drug study only will use previously seen at a clinic, site and gender). An automated email will be sent to the trial site, with LCTU confirming participant ID number and allocation arm. If the participant has consented only for the data collection, there will be a "not randomised" allocation and a Participant ID number.

d) Tracker database: The research team, using encrypted individual password remote access, should then enter the participant onto the participant tracker database (held centrally at the LCTU) and enter participants personal contact details, address, email, in order to contact the participant for follow-up data collection. This participant tracker will not contain any clinical or research data but acts as a contact list for follow-up purposes and GP contact safety reporting. Individual sites will only be able to see data relating to their own participants.

e) IMP: If the patient is participating in the drug trial, a prescription or the IMP will be issued by the PI or a designated member of the research team and the first dose taken by the participant at the clinic (if feasible depending on service design and trial provision of the recruiting site at the participating centres). Where IMP is dispensed centrally direct to patients, the first dose should be taken on receipt.

f) Wallet card: The participant will be given a Participant Wallet card with the trial registration number with the title and name of the trial drug (if applicable), the name of the PI, emergency contact number of the Research Nurse or the participating site team in case of emergency or hospital admissions.

CONFIDENTIAL

g) Research bloods and biobank samples 60 mL (as per Laboratory tests) if applicable. For participants in the drug arm this MUST be at the baseline visit. For those not taking part in the drug arm of the trial, these bloods can be taken anytime up to 12 weeks after their first appointment.

Functional and Patient Reported Outcome Assessments

All participants must have given consent before the following Patient Reported Outcome Questionnaires and functional tests are carried out:

a. Fatigue Assessment Score (FAS)
b. EuroQol Research Foundation Health related quality of life (EQ-5D-5L)
c. Mental health (GAD-7)
d. Medical Research Council Dyspnoea Score
e. Public Health Questionnaire - Depression (PHQ-9)
f. Perceived Deficit Questionnaire (PDQ-5)
g. Work and Social Adjustment Scale (WSAS) [Question 4 from Productivity Cost Questionnaire (iPCQ) for absenteeism and Question 8 from iPCQ for presenteeism added]
h. Short Form Questionnaire (SF-12)
i. Cognitive Failure Questionnaire (CFQ) if a patient scores 3 or more on PDQ5 (patients receive an email to complete this questionnaire online via a secure password and patient ID number)
j. Functional abilities and physical function using pedometer monitoring/wearables data
k. Organ impairment and healthcare utilisation
l. Cost-effectiveness of ICP
m. Process outcomes for different ICP components

As part of duty of care, research staff should review participants' responses to PHQ-9 and GAD-7 scales, in case clinical judgement is needed prior to dispensing study drug.

9.3. Assessment Visit (12 Week Assessment)

The following Patient Reported Outcome Measures and activities will be collected at the 12-week timepoint (-/+ 7 days) to record the primary and secondary outcome measures. This may not be possible in all patients due to their level of fatigue. Participants will be supported by the flexibility of data collection processes (below) to contribute as much as they are able.

For patients recruited from the London site 500 participants will be invited to return to gift a 30 mL blood sample following completion of their drug treatment for analysis comparison.

Patient reported outcome measures will be collected either at the Long COVID clinics or research clinics, by post, entered by participants themselves via secure electronic link or over the phone as per participant choice. Any non-

CONFIDENTIAL

patient reported data will be extracted from the clinical record by the site-specific research staff and input on to the eCRF database:

a. Fatigue Assessment Score
b. IMP accountability (over the phone or in person at the clinic)
c. 6-minute walk test (if performed at baseline visit and where possible undertaken at follow-up)
d. 1-minute Sit to Stand test (if performed at baseline visit and where possible undertaken at follow-up)
e. Medical Research Council (MRC) dyspnoea score
f. Modified Work and Social Adjustment Scale (WSAS) [Q4 from iPCQ for absenteeism and Q8 from iPCQ for presenteeism added]
g. General Anxiety Disorder Questionnaire- 7 (GAD-7)
h. The Primary Care Evaluation of Mental Disorders Patient Health Questionnaire (PHQ-9)
i. EQ-5D-5L (EUROQOL-5 domain- 5 level)
j. Perceived Deficit Questionnaire (PDQ-5)
k. 12-item Short Form Survey (SF12)
n. Cognitive Failure Questionnaire (CFQ) if a patient scores 3 or more on PDQ5 (patients receive an email to complete this questionnaire online via a secure password and patient ID number)
l. Adverse Event review (over the phone or in person in clinic) – Patient completed eCRFs or paper questionnaires will be reported back to site PIs by LCTU, for review and follow-up of any potential AEs reported by patients
m. Concomitant medication review (over the phone or in person at the clinic)

Patient's details including mobile phone number will be confirmed and documented in the patient's record for the purpose of sending a reminder digital text approximately 7 days before the next assessment appointment.

9.4. Follow-up Assessment (24 Weeks) Visit

All patients will be followed up for 24 Weeks (-7 days/+ 6 weeks) from the enrolment date.

These assessments will be carried out by staff based at LCTU. Participants will be given the choice of completing questionnaires online using an individualised secure link, by postal return or via telephone for participants requiring support. In the event of receiving an incomplete outcome assessment, the patient will be contacted by phone within 7 days of the team receiving the form (e-form or paper) and complete the missing information over the phone with the patient. The data will be entered into the trial database by a member of staff at Lancashire Clinical Trials Unit.

The following Patient Reported Outcome Measures and functional assessments are recorded at the 24 weeks follow up visit:

a. Fatigue Assessment Score
b. IMP accountability (over the phone or in person at the clinic)
c. 6-minute walk test (if performed at baseline visit and where possible undertaken at follow-up)
d. 1-minute Sit to Stand test (if performed at baseline visit and where possible undertaken at follow-up)
e. MRC dyspnoea score
f. Modified Work and Social Adjustment Scale (WSAS) [Q4 from iPCQ for absenteeism and Q8 from iPCQ for presenteeism added]

CONFIDENTIAL

g. General Anxiety Disorder Questionnaire- 7 (GAD-7)
h. The Primary Care Evaluation of Mental Disorders Patient Health Questionnaire (PHQ-9)
i. EQ-5D-5L (EUROQOL-5 domain- 5 level)
j. Perceived Deficit Questionnaire (PDQ-5)
k. 12-item Short Form Survey (SF12)
l. Cognitive Failure Questionnaire (CFQ), if a patient scores 3 or more on PDQ5 (patients receive an email to complete this questionnaire online via a secure password and patient ID number)
m. Functional ability and Fidelity of delivery of Treatment as Usual and Living with COVID Recovery ™
n. Adverse Event review (over the phone or in person in clinic) for participants on the nested drug trial and participants expressing suicidal ideation on the patient reported outcome questionnaires – Patient completed eCRFs or paper questionnaires will be reported back to site PIs by LCTU, for review and follow-up of any potential AEs reported by patients. AEs will be reported up to 28 days following the last dose of the trial drugs.
o. Concomitant medication review (over the phone or in person at the clinic) for participants on the nested drug trial only. Concomitant medications will be reported up to 28 days following the last dose of the trial drugs.

The outcome from these questionnaires constitutes the secondary end points for the trial.

9.5. Laboratory Procedures

It is the responsibility of the trial site to ensure that samples are appropriately labelled in accordance with the trial procedures to comply with the Data Protection Act 2018. Biological samples collected from participants as part of this trial will be transported, stored, accessed and processed in accordance with national legislation relating to the use and storage of human tissue for research purposes and such activities shall at least meet the requirements as set out in the 2004 Human Tissue Act and the 2006 Human Tissue (Scotland) Act.

9.5.1. Local analysis of Clinical Samples

Samples of blood for clinical routine care and to assess eligibility for the drug are taken by the treating physician at the Long COVID clinics (baseline visit) if clinically indicated.
Bloods may need to be taken for eligibility purposes if not available within the specified timelines (see section 9.1). These may include:

- LFT
- FBC
- eGFR
- Urine Pregnancy test
- Coagulation screening including (platelet count, activated partial prothrombin time, Prothrombin time and bleeding time)

The data from these analyses will inform the eligibility and the outcome of this trial. Clinics will follow their own local policies and procedures relating to the management of samples at NHS sites.

CONFIDENTIAL

9.5.2. Central Analysis and Storage of Clinical Samples for Research

For those participants consenting for research bloods a total of 60 mL (90 mL in total for participants at the London Site) will be taken. Blood will either be taken at clinic; for those clinics able to support the taking and processing of the samples or at a centralised facility within the area served by the clinic. The data from these analyses will inform the outcome of this trial and future research.

Research blood samples will be labelled with trial ID number, date and time taken. If the sample requires preparation locally by the site staff, the procedure will be outlined in a trial specific laboratory manual for necessary preparation and separation of different components at site, before storage and transfer, via courier to the Oxford Community Diagnostic Laboratory (Perspectum), biobank and onwards to corresponding laboratories for further analysis.

In the case of UCLH patient samples for Functional T-cell and live-virus neutralisation antibodies assay and exploratory work in Long COVID including proteomics, will be sent direct from the UCLH site to the Francis Crick Institute for analysis or held for future ethically approved research.

9.5.2.1 Samples for Future Research and Biobanking Storage at Oxford Community Diagnostic Laboratory (Perspectum)

Approximately 5mL of the research blood taken will be for biobanking at Perspectum and use in future ethically approved research studies regarding the pathophysiology and mechanism of Long COVID, where separate ethical approval may be required. This blood will be stored in an HTA registered biobank at Perspectum's central laboratory and UCL will remain the custodian.

9.5.2.2 Samples for Sub-Study Analysis and Future Research at Central Laboratories

Approximately 60mL of the blood sample will be collected for the analyses set out below and relate to the exploratory endpoint of the trial and future research. The frozen components of the blood samples will be stored at Perspectum's central laboratory. Some may be sent on to sub-contracted laboratories for testing, any that are not analysed will be stored for future research. In the case of UCLH patient samples for exploratory work on Long COVID including functional T-cell and live-virus neutralisation antibodies assay, samples will be sent direct from the UCLH site to the Francis Crick Institute for analysis and storage for future research. **.**

Guided by clinical practice, patient lived experience and latest scientific hypotheses, the following analyses will be performed in blood samples of some participants, extending to the whole cohort only if there is a clear scientific rationale. Laboratory analysis will be performed by the most appropriate partner organisation with the relevant expertise, most usually UCL, the Francis Crick Institute or Perspectum. Any samples not used within these sub-studies will be retained within the biobank at Perspectum for future research use. All samples (except those UCLH samples sent directly to the Francis Crick Institute) will be sent to Perspectum and stored until analysis by third party laboratories or kept for analysis as part of future research :

I. **Genomics analysis:** one sample per patient will be taken for initial genome-wide and focused gene (using a long list of immune-regulated genes) analyses. They will be performed using standard protocols

adjusting for any population structure. Models will incorporate clinical and environmental determinants of disease severity. Analyses of these samples will be done as future research.

II. **Proteomics analysis:** one sample per patient. Proteomics will be assessed by proximity extension assay enabling over 1400 proteins to be rapidly analysed. The assay uses oligonucleotide-labelled antibody pairs allowing for pair-wise binding to target proteins. In parallel, high-throughput, unbiased proteomics of the entire plasma proteome will be conducted on samples from UCLH held at the Francis Crick Institute to generate a protein signature for symptom clusters within Long COVID and provide insight into disease pathogenesis as part of the exploratory endpoint of the trial.

III. **Metabolomics and Lipidomics**: one sample per patient. A combination of liquid chromatography with tandem mass spectrometry (LC-MS/MS) based metabolomics and lipidomics will be performed based on a targeted analysis of over 200 metabolites of core metabolism, including acyl-carnitines, acyl-CoAs, amino acids, glycolysis and TCA intermediates and nucleotides using a Thermo Quantiva triple quadrupole mass spectrometer and lipidomics by open-profiling UHPLC-MS/MS using a Thermo Elite Orbitrap interfaced with an Advion Nanomate to allow direct nanoinfusion to detect over 600 annotated lipids. Analysis of these samples will be done as future research.

IV. **Functional T-cell and live-virus neutralisation antibodies:** in participants recruited to the London trial site. Two samples per patient will be collected (approximately 3 0 mL). Serum and peripheral blood mono-nuclear cells (PBMCs) will be isolated on arrival at the FCI. Cells will be stained and analysed using flow and mass-cytometry and neutralising antibodies quantified in the live-virus neutralisation assay. Residual plasma samples retained during extraction of PBMCs will be stored and used for unbiased, high-throughput proteomics and future analyses where indicated under exploratory endpoint 4.5.2. The Functional T-Cells assay will be done as future research.

V. **Endocrine investigation** (thyroid, hypothalamo-pituitary-gonadal and hypothalamo-pituitary-adrenal axes) will be included for those with suggestive symptoms associated with thyroiditis, autoimmune hypothyroidism and adrenal impairment for detailed phenotyping to define potential pathophysiology involved in ongoing organ-specific or physiological abnormalities for example endocrine disturbances explaining diverse nonspecific symptoms, including fatigue, hypothermia and dysmenorrhoea. Analyses of these samples will be done as future research.

CONFIDENTIAL

9.6. Schedule of Assessments

Table 4 Schedule of Assessments

	STIMULATE-ICP Trial Schedule of Assessments					
	**Procedures**	**Referral to LCC and /or Coverscan™****	**First Long COVID Clinic Visit**	**Baseline/ Visit**	**Treatment / Assessments**	**Follow Up / Assessments**
**Visiting window**			**Day 0**	No later than 7-10 days post clinic for newly referred patients	12 weeks **(-/+** 7 days)***	24 weeks **(-7 days/+ 6 weeks**)****
**Visits No**			**0**	**1**	**2**	**3**
	**Informed consent**		**X***			
**Patient's History**	**Demographics**			**X**		
	**Medical history**			**X**		
	**Concomitant Medications**			**X**		
**Physical examination**	**Height, Weight, Temperature**			**X**		
	**Resting Pulse and BP**			**X**		
**Laboratory Tests[1]**	**FBC**			**X**		
	**LFT**			**X**		
	**eGFR**			**X**		
	**Coagulation screening if required**			**X**		
	**Urine pregnancy test[5]**			**X**		
	**COVID-19 Antibody**			**X**		
**Sample Collections**	**Samples of blood for biobank storage at Perspectum, Genomics, proteomics, metabolomic & Lipidemic, functional T cell and live virus Neutralisation antibody, Endocrine analysis**			**X**	**X [6]**	

CONFIDENTIAL

	STIMULATE-ICP Trial Schedule of Assessments					
	**Procedures**	**Referral to LCC and /or Coverscan™****	**First Long COVID Clinic Visit**	**Baseline/ Visit**	**Treatment / Assessments**	**Follow Up / Assessments**
**Visiting window**			**Day 0**	No later than 7-10 days post clinic for newly referred patients	12 weeks (-/+ 7 days)***	24 weeks (-7 days/+ 6 weeks)****
**Cardiovascular**	**(ECG, ECHO, Holter Monitor, Stress Electrocardiogram)[1]**			X		
**Pulmonary**	**CXR or CMR, CT Pulmonary angiogram[1]**			X		
	**6 Minute Walk Test, 1-minute Sit to Stand test,**			X	x	x
	**PFT (FeNo)[1]**			x		
**CNS**	**MRI of brain[1]**			X		
	**Tilt Table Test[1]**			X		
**Whole Body**	**Coverscan™[2] ****	X				
**Inclusion and Exclusion**	**Eligibility assessment**			X		
	**Randomisation iv**			X		
**IMP**	**Dispensing of trial IMP [3]**			X		
	**Accountability**				X	
**Patient Reported Outcome Assessments**	**FAS Assessment**			X	X	X
	**MRC Dyspnoea Score**			X	X	X
	**General Anxiety Disorder Questionnaire- 7 (GAD-7)**			X	X	X
	**The Primary Care Evaluation of Mental Disorders Patient Health Questionnaire (PHQ-9)**			X	X	X
	**EQ-5D-5L (EUROQOL-5 domain- 5 level)**			X	X	X
	**Perceived Deficit Questionnaire) PDQ-5**			X	X	X

CONFIDENTIAL

	**STIMULATE-ICP Trial Schedule of Assessments**					
	**Procedures**	**Referral to LCC and /or Coverscan™****	**First Long COVID Clinic Visit**	**Baseline/ Visit**	**Treatment / Assessments**	**Follow Up / Assessments**
**Visiting window**			**Day 0**	No later than 7-10 days post clinic for newly referred patients	12 weeks (-/+ 7 days)***	24 weeks (-7 days/+ 6 weeks)****
	**SF12**			X	X	X
	**Cognitive Failure Questionnaire (CFQ)** [4]			X	X	X
	**Modified Work and Social Adjustment Scale (WSAS)**			X	X	X
**Safety Assessments**	**Adverse Event Reporting**				X	X[8]
**Concomitant medication**	**Concomitant medication review**				X	X[7]

* Newly referred patients who have consented to the drug study need to have their baseline appointment no later than 7-10 days post clinic. This is to align the administration on IMP with follow-up data collection and clinic pathway. Patients who do not wish to consent to the drug arm but do wish to consent to data and research blood arms only (integrated care sites) may be consented and enrolled up to 2 weeks after their first clinic appointment and gift blood samples up to 12 weeks post clinic appointment.
**Coverscan™ as usual care for allocated clusters
*** The first follow-up assessment is 12 weeks from date of randomisation, or date of consent/enrolment if data collection only
**** Follow up assessment 24 weeks from date of randomisation, or date of consent/enrolment if data collection only
1: If requested by the treating physician at Long COVID Clinics as part of their routine care
2: Only if recruited from a site randomised to Coverscan™ cluster
3: If randomised to receive the trial drug
4: If the participant scores ≥3 on PDQ5 questionnaire
5: For female participants of childbearing potential randomised to drug arm of the trial
6: Blood samples collected for functional T cell and live virus neutralisation assays from London Participants only at baseline and 12 weeks. All other participating centres will collect blood for external laboratory analysis once at baseline visit. Permission will be sort from all participants to recontact to participate in further testing due to any changes to the study on the basis of emerging evidence/results
7. Concomitant medications will be reviewed at 12- and 24-weeks visit is only for participants on the nested drug trial
8. Adverse events will be collected at the 24 week visit for participants on the nested drug trial only.

Randomisation to the drug arm can only be after clinic attendance. The participant will be consented face to face and randomised following confirmation of eligibility, this will be at the same visit if possible.

9.7. Post-Trial Treatment

There are no arrangements for the supply of the study drugs following the end of the 12-week treatment and assessment for research purposes. However, if in the opinion of the treating physician the treatment will benefit

CONFIDENTIAL

patients, it is in their discretion to continue with the treatment for as long as clinically indicated as per local trust policy. The supply of the drugs for long term use is at the discretion of the treating physician.

10. DISCONTINUATION / EARLY CESSATION OF TREATMENT AND "STOPPING RULES"

10.1. Replacements

Withdrawn participants will not be replaced. Participants who only stop treatment early have not stopped participation in the trial for the purpose of intention to treat. However, participants who withdraw consent will have their data analysed to the point of withdrawal. The PIS will make it clear that participants may remain in the trial if they stop treatment early or are not eligible for the drug trial for any reason, for the purpose of data collection and intention to treat. No data will be removed from the analysis once collected.

10.2. Cessation of Treatment and Withdrawal

Participants may be withdrawn from the trial or their treatment discontinued by the PI or after withdrawal of consent by the participant. It is always within the remit of the physician responsible for a patient to stop participation in a trial (or certain aspects of the trial) for appropriate medical reasons, be they individual adverse events or new information gained about a treatment.

The reason for withdrawal should be documented on the CRFs as soon as it occurs, and an alternative treatment plan should be provided and documented on the patient's notes. The possible reasons for withdrawal of participants include:

1. On clinical grounds such as abnormal laboratory tests
2. Severe or intractable side effects deemed to be secondary to the trial IMP administration, that cannot be resolved without discontinuation of the study drug
3. Other significant adverse reactions
4. Patient choice to withdraw consent for treatment
5. Patient choice to withdraw consent for follow up
6. Patient's choice to withdraw from all activities of the trial including data collection and retention.
7. Following safety review by the TSC/IDMC
8. Severe disease where palliative approach is adopted

All side effects should be reported initially to the treating physician and the site trial team responsible for their treatment as adverse events. The physician responsible for the patient may decide the side effects are severe enough to necessitate cessation of the trial drug immediately. For milder side effects, a discussion with the study participant should be held to ascertain if the participant is willing to continue. If the participant is unwilling, then all study medications should cease.

Cessation of drugs within the trial should occur in the following conditions; all reasons for cessation must be recorded in the appropriate CRF page, and reported to LCTU/CI/ Sponsor as appropriate:

CONFIDENTIAL

1. Reported adverse effects, if deemed severe enough to necessitate early cessation of trial drug by the physician responsible for the patient.
2. A study participant is diagnosed with a new condition that necessitates cessation of the trial drug or modification of the dose.
3. A study participant is intolerant of the prescribed dose or reports significant adverse effects.
4. Where the trial drug is newly contraindicated by a new diagnosis.
5. Report of a new pregnancy in female participants.

A decision by a participant that they no longer wish to continue receiving the trial drug should **not** be considered to be a withdrawal of consent for follow-up or data collection unless the patient explicitly expresses that they no longer want their data collected or to be followed up. However, participants are free to withdraw consent for some or all aspects of the study at any time if they wish to do so. Ideally, they should understand the implications of this. In accordance with regulatory guidance, de-identified data that have already been collected will continue to be used (and any identifiable research data held by LCTU or Target Healthcare Ltd will be destroyed). Any stored blood or tissue samples that can still be identified as theirs will be destroyed if the participant explicitly requests this.

Participants who lose capacity during the study will be withdrawn from the trial and no further data will be collected or any trial investigation or assessments conducted.

The trial may be prematurely discontinued by the Sponsor, CI, or regulatory authority based on new safety information or for other reasons given by the Ethics Committee, MHRA or TSC on recommendations of the regulatory authority concerned.

If the trial is prematurely discontinued, active participants will be informed and no further participant data will be collected. The MHRA and Research Ethics Committee will be informed within 15 days of the early termination of the trial.

10.3. Lost to Follow-up

If a participant moves from the area, every effort should be made for the participant to be followed up at another participating trial site and for this new site to take over the responsibility for the participant.
If a participant is lost to follow-up at a site every effort should be made to contact the participant's GP to obtain information on the participant's status. The PIL and ICF will include consent to contact the participant's GP to obtain information about the participant in relation to the treatment or inclusion in the trial.

11. SAFETY REPORTING / PHARMACOVIGILANCE

Collection, recording and reporting of adverse events to the sponsor will be completed according to the sponsor's SOP for the Recording, Management and Reporting of Adverse Events by Investigators (JRO/INV/S05).

11.1. Definitions

Table 5: Definitions

CONFIDENTIAL

Term	Definition
Adverse Event (AE)	Any untoward medical occurrence in a subject to whom a medicinal product is administered and which does not necessarily have a causal relationship with this treatment.<br>*Therefore an AE can be any unfavourable or unintended change in the structure (signs), function (symptoms) or chemistry (laboratory data) in a subject to whom an IMP has been administered, including occurrences which are not necessarily caused by or related to that product.*
Adverse Reaction (AR)	A response to a medicinal product which is noxious and unintended, and which occurs at doses normally used in man for the prophylaxis, diagnosis, or therapy of disease or for the restoration, correction, or modification of physiological function.<br>*This definition implies a reasonable possibility of a causal relationship between the event and the IMP. This means that there are facts (evidence) or arguments to suggest a causal relationship.*<br>*This definition also covers medication errors and uses outside what is foreseen in the protocol, including misuse and abuse of the product.*
Serious Adverse Event (SAE)<br>or<br>Serious Adverse Reaction (SAR)	Any adverse event or adverse reaction in a trial subject that:<br>• Requires inpatient hospitalisation or prolong action of existing hospitalisation<br>*(Note: hospitalisation is defined as an inpatient admission, regardless of length of stay, even if the hospitalisation is a precautionary measure for continued observation. Therefore, participants do not need to be hospitalised overnight to meet the hospitalisation criteria. Hospitalisation (including hospitalisation for an elective procedure) for a pre-existing condition (prior to study entry) which has not worsened does not constitute a serious experience).*<br>• Results in persistent or significant disability or incapacity<br>*(Note: substantial disruption of one's ability to conduct normal life functions).*<br>• Results in a congenital anomaly or birth defect<br>*(Note: in offspring of subjects or their partners taking the IMP regardless of time of diagnosis).*<br>• Is life threatening<br>*(Note: places the subject, in the view of the investigator, at immediate risk of death from the experience as it occurred, this does not include an adverse experience that, had it occurred in a more severe form, might have caused death);* or<br>• Results in death<br><br>Some medical events may jeopardise the subject or may require an intervention to prevent one of the above characteristics/consequences. Such important medical events should also be considered as serious.<br>The term "severe" is often used to describe the intensity of an event or reaction (e.g., mild, moderate, or severe) and should not be confused or interchanged with the term "serious".

CONFIDENTIAL

Term	Definition
Suspected Unexpected Serious Adverse Reaction (SUSAR)	A serious adverse reaction, the nature, severity, or outcome of which is not consistent with the Reference Safety Information (RSI).
Reference Safety Information (RSI)	A list of medical events that defines which reactions are expected for the IMP being administered to clinical trial subjects, and so do not require expedited reporting to the MHRA. It is contained in a specific section in the Summary of product characteristics (SmPC) or the Investigator Brochure (IB).

11.2. Recording and reporting of Adverse Events

All adverse events (AEs) will be recorded in the participant's medical records in the first instance, regardless of the allocation of the participant in the nested drug trial or the cluster trial.
Adverse events will be recorded with clinical symptoms and accompanied with a simple, brief description of the event, including dates as appropriate.

AEs for participants on the data collection arm do not need to be recorded in the eCRF.

For participants enrolled in the nested drug trial all AEs will be recorded in the eCRF following consent. For participants randomised to standard of care drugs only, AEs will be recorded until 12 weeks post enrolment. For participants randomised to receive an IMP, AEs will be recorded until 28 days following last dose of an IMP.

Where possible, a diagnosis rather than a list of symptoms should be recorded. If a diagnosis has not been made, then each symptom should be listed individually. All AEs should be captured on the appropriate AE pages in the eCRF.

11.3. Assessing Adverse Events

Each adverse event (AE) will be assessed for severity, causality and seriousness as described below:

11.3.1. Severity

The intensity will be determined by using the following definitions:

Table 6: Definitions of grades of severity of the Adverse Events

Category	Definition
**Mild**	The adverse event does not interfere with the participant's daily routine and does not require intervention; it causes slight discomfort.
**Moderate**	The adverse event interferes with some aspects of the participant's routine, or requires intervention, but is not damaging to health; it causes moderate discomfort.
**Severe**	The adverse event results in alteration, discomfort or disability which is clearly damaging to health.

CONFIDENTIAL

11.3.2. Assessing Causality

The assessment of relationship of adverse events to the administration of IMP must be made by the investigator (or delegated medically qualified person). It is based on clinical judgement using all available information at the time of the completion of the CRF.

The following categories will be used to define the causality of the adverse event:

Table 7: Definitions of relatedness of the SAEs to the trial drugs

Category	Definition
**Related**	A causal relationship between an IMP/investigational treatment and an adverse event is at least a **reasonable possibility**, i.e. the relationship cannot be ruled out.
**Not Related**	There is **no reasonable possibility** of a causal relationship between an IMP/investigational treatment and an adverse event.

11.3.3. Seriousness

All events are assessed for seriousness as defined for a serious adverse event (SAE) in the definitions table.

11.4. Assessing Serious Adverse Events and Serious Adverse Reactions (nested drug trial participants only)

All Serious Adverse Reactions (SARs) occurring from the time of written informed consent until 28 days after the last dose of the IMP, or the end of their assessments at 12 weeks for participants on standard of care drugs only, must be recorded in the medical records, the eCRF and the trial specific SAE Reporting Form and reported to the Sponsor **within 24 hours** of the research staff becoming aware of the event.
The Investigator or designated individual will complete the Sponsor's trial specific SAE Reporting Form and email it to the Sponsor at SAE@ucl.ac.uk **and** LCTUSTIMULATE@uclan.ac.uk. The Investigator will respond to any SAE queries raised by the Sponsor as soon as possible.

Completed SAE Reporting Forms must be sent to the Sponsor within 24 hours of becoming aware of the event

Email SAE Forms to: SAE@ucl.ac.uk **and** LCTUSTIMULATE@uclan.ac.uk

Any change of condition or other follow-up information should be emailed to the Sponsor, on an SAE Reporting Form (clearly marked as follow-up) as soon as it is available or at least within 24 hours of the information becoming available.

CONFIDENTIAL

Events will be followed up until the event has resolved or a final outcome has been reached. SAE follow-up should continue after completion of protocol treatment and/or trial follow-up if necessary. Any SAR will need to be reported to the Sponsor, irrespective of how long after IMP administration the reaction has occurred until resolved.

The SAE listing will be reported from each site to the CI and Sponsor upon request.

11.5. Serious Adverse Events Which Do Not Require Reporting to Sponsor

SAEs for participants on the data collection only arm of the trial do not need recording in the eCRF or reporting to Sponsor with the exception of the SAEs which are suggestive of suicidal ideation following review of participant completed questionnaire which will be recorded and reported to the Sponsor.

For participants enrolled in the nested drug trial, the following events do not require immediate reporting to the sponsor as SAEs, however they will still be recorded in the participant's medical records.

Hospitalisation for:

- Routine treatment or monitoring of the studied indication not associated with any deterioration in condition.
- Any admission to hospital or other institution for general care where there was no deterioration in condition.
- Treatment on an emergency, outpatient basis for an event not fulfilling any of the definitions of serious as given above and not resulting in hospital admission.

11.6. SUSAR Reporting

All SAEs assigned by the PI or delegate as suspected to be related to IMP-treatment (SARs) will be assessed for expectedness against the current approved RSI for the trial by the Sponsor.

The following categories will be used to define the expectedness of the SAR:

Table 8: Definitions of expectedness of the SARs

Category	Definition
Expected	An adverse event which is consistent with the information about the IMP listed in the current approved Reference Safety Information (RSI) for the trial.
Unexpected	An adverse event which is not consistent with the information about the IMP listed in the current approved Reference Safety Information (RSI) for the trial.

All SARs assessed as unexpected will be classified as SUSARs and will be subject to expedited reporting to the MHRA and REC.
The RSI to be used to assess expectedness against the IMPs are:

- SmPC Famotidine 40mg Tablets (Tillomed Laboratories Ltd), Section 4.8: Undesirable effects
- SmPC Clarityn Allergy (Loratadine) 10mg Tablets (Bayer plc), Section 4.8: Undesirable effects

CONFIDENTIAL

- SmPC Colchicine Tablets BP 500 mcg (Wockhardt UK Ltd), Section 4.8: Undesirable effects
- SmPC Xarelto (Rivaroxaban) 10 mg film-coated tablets (Bayer plc), Section 4.8: Undesirable effects

The sponsor will inform the MHRA within the required expedited reporting timescales. SUSARs that are fatal or life-threatening must be notified to the MHRA within 7 days after sponsor awareness. Other SUSARs must be reported to the MHRA within 15 days after sponsor awareness.

CONFIDENTIAL

11.7. Adverse Event Recording and Processing Flow Chart

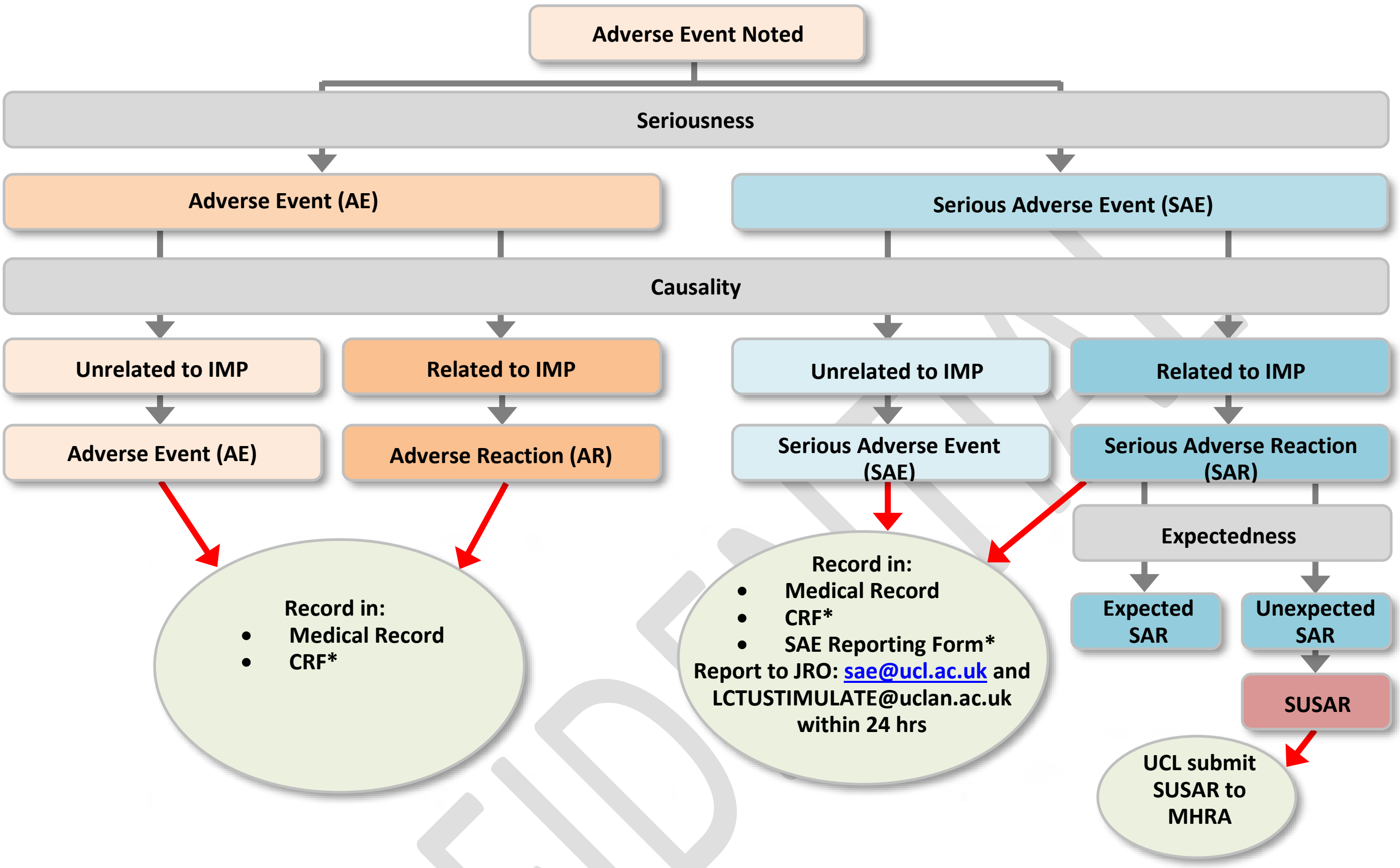

** AEs/SAEs recorded in CRF / SAE reporting Forms for nested drug trial participants only*

CONFIDENTIAL

11.8. Notification of deaths (nested drug trial participants only)

All deaths will be reported to the Sponsor within 24 hours of the Research Team becoming aware of it as an SAE.

- All deaths will be reported to the sponsor irrespective of whether the death is related to disease progression, the IMP, or an unrelated event.
- All deaths, including deaths deemed unrelated to the IMP, if they occur earlier than expected, will be reported to the sponsor.

11.9. Pregnancy reporting (nested drug trial participants only)

If a female participant becomes pregnant (or partner of a male participant who has received the IMP colchicine) at any point during their treatment in the trial and up to 30 days from last dose (female participants) or 90 days from last dose (partner of male participant), a completed trial specific Pregnancy Reporting Form will be completed and emailed to the Sponsor at SAE@ucl.ac.uk and **LCTUSTIMULATE@uclan.ac.uk**, within 24 hours of the Investigator becoming aware of the event. The Investigator will respond to any queries raised by the Sponsor as soon as possible.

Completed Pregnancy Reporting Forms must be sent to the Sponsor within 24 hours of becoming aware of the event<br>**Email Pregnancy Forms to: SAE@ucl.ac.uk and LCTUSTIMULATE@uclan.ac.uk**

The Sponsor must be kept informed of any new developments involving the pregnancy through the completion of a follow-up Pregnancy Reporting Form. Any pregnancy that occurs in a female trial subject during a clinical trial should be followed to termination or to term.

Consent to report information regarding the pregnancy include follow-up of a child born if applicable must be obtained from the pregnant participant including the partner if applicable. A trial-specific pregnancy monitoring information sheet and informed consent form for trial participants including the Pregnant Partner of trial participants if applicable must be used for this purpose.

With consent, additional information regarding the pregnancy will be collected and reported to the Sponsor, the Sponsor will advise on the length of follow up of the pregnancy/child on a case-by-case basis.

11.10. Overdose Reporting

All incidences of overdose will be reported on eCRFs and reported as an SAE within 24 hours of the trial team becoming aware of it. The priority for the trial team is to assist with directing patients affected to be treated urgently through standard NHS emergency admission to the nearest Accident and Emergency department for immediate medical attention and treatment. The patient's GPs will be notified of the

CONFIDENTIAL

incident and the patient will be monitored under standard of care. The patient will be discontinued on the trial drug but remain in the trial for follow up and assessment at the usual 12 and 24-week assessment.

11.11. Loratadine Overdose

Overdosage with Loratadine increased the occurrence of anticholinergic symptoms. Somnolence, tachycardia, and headache have been reported with overdoses.

In the event of overdose, general symptomatic and supportive measures are to be instituted and maintained for as long as necessary. Administration of activated charcoal as a slurry with water may be attempted. Gastric lavage may be considered.

Loratadine is not removed by haemodialysis, and it is not known if Loratadine is removed by peritoneal dialysis.

Patients will be sign posted for urgent medical attention at the emergency department of the nearest hospital to the patient's residence. Medical monitoring of the patient is to be continued after hospital treatment.

11.12. Famotidine Overdose

The adverse reactions in overdose cases are similar to the adverse reactions encountered in normal clinical experience (see the adverse reactions table for Famotidine in Appendix I).

No incidence of overdose with Famotidine had been reported so far from clinics. Patients who had received daily doses of up to 800 mg over a period of one year without exhibiting any significant undesirable effects.

In the event of overdose, the aim should be to remove any unabsorbed drug from the alimentary tract with the usual measures from the gastrointestinal tract; clinical monitoring and supportive therapy should be employed.

11.13. Colchicine Overdose

Procedures for Colchicine overdose will follow those outlined in the SmPC for Colchicine. The lethal dose varies widely (7 - 65 mg single dose) for adults but is generally about 20 mg. Therefore, overdose of study Colchicine treatment is unlikely during the trial, given the low dose of Colchicine (1 mg daily)

In the unlikely event of Colchicine overdose, all patients, even in the absence of early symptoms, should be referred for immediate medical assessment at an emergency department closest to the patient's residence for urgent medical treatment.

Symptoms of acute overdosage may be delayed (3 hours on average): nausea, vomiting, abdominal pain, haemorrhagic gastroenteritis, volume depletion, electrolyte abnormalities, leucocytosis, hypotension in severe cases. The second phase with life threatening complications develops 24 to 72 hours after drug administration: multisystem organ dysfunction, acute renal failure, confusion, coma, ascending peripheral motor and sensory neuropathy, myocardial depression, pancytopenia, dysrhythmias, respiratory failure,

consumption coagulopathy. Death may result from respiratory depression and cardiovascular collapse. If the patient survives, recovery may be accompanied by rebound leucocytosis and reversible alopecia starting about one week after the initial ingestion.

Treatment:

No antidote is available.

Treatment will focus on elimination of toxins by gastric lavage if treatment is begun early (within one hour of acute poisoning). Oral activated charcoal may be of benefit in adults who have ingested more than 0.1 mg/kg bodyweight within 1 hour of presentation. Haemodialysis has no efficacy.

Close clinical and biological monitoring in hospital environment will be performed, plus symptomatic and supportive treatment (control of respiration, maintenance of blood pressure and circulation, correction of fluid and electrolytes imbalance.

11.14. Rivaroxaban Overdose

Rare cases of overdose up to 600 mg have been reported without bleeding complications or other adverse reactions. Due to limited absorption a ceiling effect with no further increase in average plasma exposure is expected at supratherapeutic doses of 50 mg Rivaroxaban or above.

A specific reversal agent (andexanet alfa) antagonising the pharmacodynamic effect of Rivaroxaban is available (refer to the SmPC of andexanet alfa).

The use of activated charcoal to reduce absorption in case of Rivaroxaban overdose may be considered.

Management of bleeding

In the event of overdose with Rivaroxaban treatment should be discontinued. Rivaroxaban has a half-life of approximately 5 to 13 hours.

Management should be individualised according to the severity and location of the haemorrhage. Appropriate symptomatic treatment could be used as needed, such as mechanical compression (e.g. for severe epistaxis), surgical haemostasis with bleeding control procedures, fluid replacement and haemodynamic support, blood products (packed red cells or fresh frozen plasma, depending on associated anaemia or coagulopathy) or platelets.

If bleeding cannot be controlled by the above measures, either the administration of a specific factor Xa inhibitor reversal agent (andexanet alfa), which antagonises the pharmacodynamic effect of Rivaroxaban, or a specific procoagulant reversal agent, such as prothrombin complex concentrate (PCC), activated prothrombin complex concentrate (APCC) or recombinant factor VIIa (r-FVIIa), should be considered. However, there is currently very limited clinical experience with the use of these medicinal products in

individuals receiving Rivaroxaban. The recommendation is also based on limited non-clinical data. Re-dosing of recombinant factor VIIa shall be considered and titrated depending on improvement of bleeding. Depending on local availability, a consultation with a coagulation expert should be considered in case of major bleedings. Protamine sulphate and vitamin K are not expected to affect the anticoagulant activity of Rivaroxaban. There is limited experience with tranexamic acid and no experience with aminocaproic acid and aprotinin in individuals receiving Rivaroxaban. There is neither scientific rationale for benefit nor experience with the use of the systemic haemostatic desmopressin in individuals receiving Rivaroxaban. Due to the high plasma protein binding Rivaroxaban is not expected to be dialysable.

11.15. New Safety Findings

If a new safety finding emerges (from sources such as IMP manufacturers, data analysis, IDMC findings), the CI reviews the finding for its impact on the subjects participating in the relevant trial(s). If there is a potential impact on trial participant's safety, the Sponsor takes appropriate action in conjunction with the CTM, CI and research team. Appropriate reporting mechanisms are followed in the event of actions being taken.

11.16. Urgent Safety Measures

Where an urgent safety measure (to prevent immediate hazard to trial subject's health and safety) is necessary, prior authorisations from the MHRA and ethics are not required.

Where the PI takes urgent action that is not consistent with the protocol to prevent harm to a subject on a trial, the PI must immediately **report** via phone to the Clinical Trial Unit at the MHRA (020 3080 6456) and discussed with a safety scientist/medical assessor. This should be done ideally **within 24 hours** of measures being taken, but **no later than 3 days.** In addition, the PI must immediately inform the CI and the CTM and give full details of the measures taken, the decision-making process surrounding the action(s) taken and the details of the conversation with the MHRA. The CTM will inform the Sponsor, of these measures immediately, and report to the MHRA via IRAS **no later than 3 days** of the actions being taken.

Further guidance can be found in the study specific guidance document 'Identifying, Recording & Reporting of Protocol Deviations, Violations, Potential Serious Breaches, Serious Breaches and Urgent Safety Measures'.

An amendment is formally submitted as soon as possible by the CI and CTM to the relevant bodies in conjunction with the sponsor.

11.17. Development of Safety Update Reports

The Sponsor will provide the MHRA with Development Safety Update Reports (DSUR) which will be written by the Sponsor's office in conjunction with the trial team. The report will be submitted within 60 days of the Developmental International Birth Date (DIBD) of the trial each year until the trial is declared ended.

11.18. Responsibilities

11.18.1. Responsibilities of Principal Investigator / Delegates

Checking for AEs and ARs when participants attend for treatment/follow-up.

1. Using medical judgement in assigning seriousness and causality.
2. Ensuring that all SAEs are recorded and reported to the sponsor within 24 hours of becoming aware of the event and provide further follow-up information as soon as available. Ensuring that SAEs are chased with Sponsor if a record of receipt is not received within 3 working days of initial reporting.
3. Ensuring that AEs and ARs are recorded and reported to the sponsor in line with the requirements of the protocol.

11.18.2. Responsibilities of Chief Investigator / Delegates

All of the above responsibilities of a PI, and in addition:

1. Clinical oversight of the safety of participants participating in the trial, including an ongoing review of the risk / benefit.
2. Using medical judgement in assigning the SAEs seriousness and causality where it has not been possible to obtain local medical assessment.
3. Review of all SUSARs.
4. Review of specific SAEs and SARs in accordance with the trial risk assessment and protocol.
5. Reporting safety information to the independent oversight committees identified for the trial including Data Monitoring Committee (DMC) and / or Trial Steering Committee (TSC).
6. Reviewing and contributing to the annual Development Safety Update Report (DSUR).

11.18.3. Responsibilities of the Sponsor / Delegates

1. Data collection and verification of SAEs, SARs and SUSARs according to the trial protocol onto a database.
2. Reporting safety information to the CI or delegate for the ongoing assessment of the risk / benefit.
3. Expedited reporting of SUSARs to the Competent Authority (MHRA in UK) and REC within required timelines.
4. Notifying Investigators of SUSARs that occur within the trial.
5. Checking for and notifying PIs of updates to the Reference Safety Information for the trial.
6. Preparing the DSUR in collaboration with the CI and ensuring timely submission to the MHRA and REC (within 60 calendar days).

12. DATA MANAGEMENT AND QUALITY ASSURANCE

In accordance with the principles of Good Clinical Practice and the recommendations and guidelines issued by UK HRA and MHRA, the design, conduct and analysis of this trial is focussed on issues that might have a material impact on the wellbeing and safety of study participants with Long COVID and the reliability of the results that would inform the care for future patients.

Quality Control (QC) includes the operational techniques and activities done within the QA system to verify that the requirements for quality of the trial-related activities are fulfilled. A risk-adapted approach will be

used for monitoring. All sites will be centrally monitored for recruitment, data completeness, quality and timeliness of data entry, number of data change requests. A minimum routine remote monitoring schedule of 3 monthly will be set. Any concerns not resolved or raised as a result of the remote monitoring will trigger a full site monitoring visit. To this end:

- Coordinated by LCTU Trial Manager and monitor, site initiation visits will be performed to enable training of local research personnel
- The Trial Manager at LCTU will document the completion of the initiation checklist for each site to verify appropriate approvals are in place prior to initiation of the site
- The Trial Manager will document, as part of the initiation checklist, that all relevant personnel have undergone trial specific training
- Data will be centrally monitored by the LCTU Monitor and Trial Manager to check:
    - Adverse Event reporting rates between centres
    - Screening, recruitment, and dropout rates between centres
    - Data entry consistency. The Data Manager and Trial Manager, with the Monitor, will follow-up on the data queries; central monitoring reports will thus be generated for the TMG, who will oversee the activity and in accordance with the monitoring plan, will identify when additional intervention, e.g., site visits, should be undertaken.
    - Independent oversight of the trial will be provided by IDMC and independent members of the TSC.

Among the most important factors influencing the delivery of these quality objectives are:

- Minimising the burden on the clinicians working in overstretched Long COVID clinics.
- Ensuring suitability of the participants and having access to the trial treatment without impacting on their other medical needs.
- Ensuring information given to the participants and the PIs in a timely and readily digestible fashion without adversely impacting on the patient care.
- To allow the treating physician to use their clinical judgement to decide whether any of the treatment arms are not suitable for the patient under their care.
- To collect comprehensive information on the mortality as well as morbidity of the Long COVID status.
- In all aspects of the trial, any risks to the patients and well-being will be a key principle in that of proportionality. Risks associated with participation in the trial must be considered in the context of usual care.

13. DATA COLLECTION AND SOURCE DOCUMENT IDENTIFICATION

Data will be collected from sites on trial-specific case report forms or data collection tools such as electronic CRFs and electronic documents.

Source data are contained in source documents and must be accurately transcribed on to the paper CRF (pCRF). Examples of source documents are medical records which include laboratory and other clinical reports etc.

A source document list will be implemented and recorded in the Data Management Plan and Monitoring Plan, prior to the start of the trial, to identify:

a) which data is to be recorded directly onto the pCRF
b) which data is recorded firstly into source documents, such as medical notes, laboratory reports or scan reports and then transcribed into the pCRF for entry into the trial database
c) which data is not to be recorded in the pCRF but only recorded in source documents
d) Data entry into the eCRF, completing and closing a record
e) Data change requests

The Case Report Forms (CRFs) will not bear the participant's name or other personal identifiable data.

13.1. Data Collection from Coverscan™

This trial aims to measure the prevalence of organ volume changes and damages in lungs, heart, kidney, liver, pancreas, spleen as assessed by the MRI scanner among those having recovered, or recovering, from the SARS-CoV-2 infection in the cluster randomised trial. Patients who are allocated by PCN deprivation score to receive the Coverscan™, will be referred for the scan 4 weeks prior to their Long COVID clinic appointment. The scan results will be assessed and reported on by a specialist radiologist group supported by Perspectum. Reports will be returned to clinic site and become part of the clinical record. Reports should be available to clinicians at the time of the clinic appointment to inform care decision making.

13.2. Data Collection from Long COVID Clinics

Routine standard of care clinical data from patients undergoing investigations at the 6 participating Long COVID clinics will be captured through the patient facing questionnaire pCRF and research staff completed pCRF (clinical data and investigations collected from the clinical record and physical assessment of participant) by a member of the trial team at the site.

13.3. Completing Case Report Forms

All eCRFs and pCRFs must be completed and signed by the research team staff that are listed on the site staff delegation log and authorised by the PI to perform this duty. The PI is responsible for the accuracy of all data reported in the CRFs. The information from pCRF will be entered into an eCRF within the electronic trial database by the site team directly for the baseline and 12 week assessment (where the participant attends in person). All site team members will have individualised encrypted access to the database allowing for all entries and changes to be seen within the database audit and change log.

For virtual clinics and where the patients are not returning to the clinic in person, participants will be given the option of completion via postal, elink (directly into database for self-completion) or over the telephone for participants requiring support.

All the CRFs will be entered into the trial database within 15 days of data received by the site staff or LCTU staff as applicable.

13.4. Data Handling and Analysis

LCTU data management SOP (DM-03 and DM-05) will be followed and a trial specific Data Management Plan will be in place for the trial. This will contain details of the software to be used for the database, the process of database design, data validation plan, data entry, data quality checks, data queries, data security, database lock.

Where data are transferred electronically this will be in accordance with the UK Data Protection Act 2018 as well as UCL, LCTU and UCLan Information Security Policy and Trust Information Governance Policy. Data Transfer and the associated processes between organisations in the trial have been / will be reviewed by the Sponsor and will be covered by appropriate data sharing agreements.

There will be a documented record of data transfer and measures in place for the recovery of original information after transfer.

Within the trial, data will be transferred to and from Perspectum, Living with COVID Recovery™, and the Crick Institute, by LCTU. The data transfer arrangements will be documented in the data management plan and in appropriate agreements.

13.5. Access to the final trial dataset

The CI, Sponsor and trial statisticians will have access to the full dataset prior to database lock and data analysis at the end of the trial. Collaborators and the PIs have access to pseudonymised data.

A copy of the data dictionary will be deposited with the Sponsor and a copy archived at LCTU to comply with the regulatory requirement for archiving clinical trials data.

14. STATISTICAL CONSIDERATIONS

14.1. Sample Size Calculation – Main study

Motivation: The main study takes the form of a 2x2 factorial trial where the unit of randomisation is the PCN. The sample size calculation was based on the power to detect an interaction effect of clinically important size of 3 points (40) on the FAS scale (41) rather than on the size of the main effects themselves. Therefore, the sizes of the main effects are not specified below. For context, the difference in means of FAS between people who say they have or have not recovered from Long COVID was 9 points in an observational study (41).

The sample size was calculated using PASS v21.0.1(2021) for a 2x2 cluster factorial trial. The study was powered to detect an interaction on the FAS scale between Coverscan™ and Living with COVID Recovery™. The sample size does not depend on the size of main effects of both individual interventions. Based on published data the standard deviation on the FAS scale for patients with Long COVID is estimated at 6 units (see Figure 12 of reference (41). An interaction effect of 3 points (40) on the FAS can be detected with just over 90% power and (two-sided) significance level of 0.05 with 960 participants in 48 PCN clusters of 20 participants, assuming a conservative intra-cluster correlation coefficient (ICC) of 0.02. If there is dropout

of 15% (to be conservative) and assuming missingness is roughly equal across the arms, then the number of participants must be inflated by a factor of 100/85 to 1130 (approximately 56.5 centres of 20 participants).

Only if there is no possibility of an interaction effect between the drugs of the platform trial and the main and interaction effects in the 2x2 factorial trial, can the trials be considered independent and data from all arms of the adaptive trial can be combined to assess the effects within the 2x2 factorial trial. Assuming non-independence of the two trials, the total number of participants required for the interaction effect within the factorial trial to be detected with 90% power was calculated as 1130 receiving usual care (i.e., not entering the drug trial or randomised to usual care in the drug trial).

Experimental estimates of the prevalence of symptoms that remain 12 weeks after COVID infection range from 3.0% based on tracking specific symptoms, to 11.7% based on self-classification of Long COVID, using data to 1 August 2021 (3). One Long COVID clinic has ~25 new patients per week(16), suggesting there might be around 1300 new patients annually per clinic. Most are expected to give consent to data collection even if not willing to consent to randomisation into a drug study. Long COVID referral rates range from 0-2.5/1000 per PCN. Assuming this rate will not change over the foreseeable future, this will ensure that our 6-10 Long COVID clinics will provide sufficient patients for this study. Moreover, there is a backlog of people waiting for their first Long COVID clinic appointment (from private communication).

Lower than expected recruitment numbers have necessitated a partial decoupling of the cluster trial and the drug trial and the inclusion of drug only centres that do not contribute participants to the cluster trial. Patients previously seen at an integrated care clinic will also be able to join the drug trial, but not the cluster trial. It has been necessary to make the assumption that there will be little interaction effect between the drugs of the platform trial and the main and interaction effects in the 2x2 factorial trial. With this assumption, the total number of participants required for the interaction effect within the factorial (cluster) trial to be detected with 90% power is 1130, regardless of their participation in the drug trial or specific treatment allocation within the drug trial.

14.2. Sample Size Calculation – Nested Drug Trial (and Non-Nested Drug Trial)

The total number of individual drugs tested on the platform is currently unknown.

The total sample size required in an adaptive platform drug trial, with uncertainty in the timing of the introduction of further drugs, cannot be set in advance, but would need to be updated in consultation with the IDMC and funder throughout the trial at set interim points in data collection.

The treatment effect is unknown at this time for the drugs. The plan was to perform interim analysis after 1200 participants in total were recruited and after every 600 participants thereafter. However, the plan for interim analyses was removed through oversight consultation due to changes in the total recruitment target.

With 200 participants per active arm and corresponding concurrent controls the study has 85% power to detect a Cohen's D of 0.3 on FAS (difference between means in two treatment arms divided by the standard deviation of the data) at the 0.05 alpha level. If this number is increased to 300 for the same effect size the power is 95%.

This calculation is not altered by the inclusion of clinics that can only offer the usual care treatment pathway, but offer participants the opportunity to join the drug trial.

14.3. Planned Recruitment Rate

An estimate of the recruitment period for the trial (calculated based on the expected number of eligible and recruited participants available per month/year) with justification that the required sample size will be attainable in practice within 24 months. To plan and cost the trial a nominal maximum of 4520 recruited participants was proposed based on up to 4 (drug and usual care) parallel arms of up to 1130 evaluable participants. The nested drug trial aims to recruit up to 4520 patients from a possible pool of 30,000 patients from 6-10 PCN areas of England. The trial will continue to recruit participants at least until the recruitment target of 1130 participants for each of the initial arms of the adaptive trial has been reached. If the adaptive trial requires the full 4520 participants, it is estimated it will take approximately 10 to 12 months for the recruitment to complete and by the 16th month from the start of the trial the last patient last visit would be completed in first quarter of 2023. The projected recruitment rate is 600 participants per month and approximately 100 participants per month per clinic will need to be consented for data collection into the cluster randomised trial.

A 6-month time extension, the observed recruitment rate into the cluster trial to end of May 2023 of approximately 84 participants per month, and the decision to partially decouple the cluster and drug trials through the inclusion of drug only sites, means that a revision of the planned recruitment rate is required. At least 1130 participants not previously seen by a long Covid clinic, are required in the cluster trial from the cluster trial sites. At least 56 participants per month will need to be consented for data collection into the cluster randomised trial. At least 81 participants per month are required to be recruited to the drug trial from either the cluster trial sites or the drug trial only sites if there are to be at least 200 per drug treatment arm. This is increased to 138 participants per month if there are to be at least 300 per drug treatment arm.

14.4. Randomisation Methods

Randomisation to the cluster trial will be at the PCN level. Block randomisation for the 226 PCNs will be used with blocks of size 4. There will be two factors in the randomisation, and within each clinic: PCN size (categorised into 2 levels) and PCN deprivation (categorised into 2 levels) (defined in Section 8). Within each clinic, the strata of PCNs will be arranged as larger and more deprived; larger and less deprived; smaller and less deprived; and smaller and more deprived, before randomised allocation into the 4 cluster allocations. The randomisation ratio is 1:1:1:1 between the 4 cluster allocations.

For participants from the integrated care sites, randomisation to the drug trial will be at the individual level. Block randomisation will be used with varying block sizes. There will be three factors in the randomisation within each clinic: site, gender (male vs female), treatment pathway and whether or not they have previously attended a clinic. To be able to use a single randomisation process, those who have previously attended a clinic will be nominally assigned to the usual care treatment pathway (No Coverscan™+ Usual Care Self- Management Rehabilitation) before being allocated to a drug treatment. The randomisation ratio will be equal between all of the drug treatment arms (including usual care) open to recruitment at that point in the adaptive trial.

Participants from the cluster sites who have previously attended a clinic,

For clinics only able to offer participation in the drug trial, there will be two factors in the randomisation within each clinic: gender (male vs female) as all patients at these clinics will receive the usual care treatment pathway, and whether or not they have previously attended a clinic. The randomisation ratio will be equal between all of the drug treatment arms (including usual care) open to recruitment at that point in the adaptive trial.

14.5. Statistical Analysis Plan

The trial will be analysed and reported using the "Consolidated Standard of Reporting Trials" ("CONSORT") and the International Conference on Harmonisation E9 guidelines. The main features of the statistical analysis plan are included here.

14.6. Summary of Baseline Data and Flow of Participants

A CONSORT diagram will be produced to report of the flow of participants in the cluster trial. There will be a description of screening log data for eligible participants. There will be a further summary of baseline characteristics of all participants that consent to data collection, by frequency and percentage for categorical variables, and for continuous variables by mean and standard deviation (or median and inter-quartile range for non-normally distributed data).

A CONSORT diagram will be produced to report the flow of participants in all arms of the trial. Summary of baseline characteristics, by study arms, will be by frequency and percentage for categorical variables, and for continuous variables by mean and standard deviation (or median and inter-quartile range for severely skewed data).

14.7. Primary Outcome Analysis

Primary analysis of the trial will be on the basis of the cluster randomisation of the Participant's PCN or the stratified randomisation of the individual participant and will be undertaken by the STIMULATE-ICP team at UCLAN. The primary outcome, FAS at 12 weeks, is expected to be approximately normally distributed (40). A multi-level model analysis will be used to evaluate effects of Coverscan™, digitally enabled community rehabilitation, and their interaction, on FAS at 12 weeks adjusting for baseline FAS, with gender as a fixed effect and clinic and PCN as random effects.

The principal analysis population for these estimates of the effects of Coverscan™, digitally enabled community rehabilitation, and their interaction, has been changed to reflect lower than expected recruitment and will be those participants who consented to data collection for the cluster RCT regardless of their participation in the drug trial or their drug trial treatment allocation. This analysis will provide a generalisable answer to the primary objective in the broadest group of participants in the presence of other treatments including drug treatments allocated in the trial or obtained another way.

A similar multi-level model analysis of the effects of Coverscan™, digitally enabled community rehabilitation, and their interaction, will be applied to all participants who consent to data collection in the cluster trial restricted to those taking no drug, through random allocation to no drug, through ineligibility or through refusal of participation in the drug trial. This analysis, although having lower power than originally planned, will provide an answer to the primary objective in the absence of other treatments including drug treatments and will allow comparison to the results from the principal analysis set.

The principal analysis population for drug vs. usual care comparisons within the drugs trial will be anyone consenting to the drug trial including participants also cluster randomised to any treatment pathway in the cluster trial, participants from cluster trial sites who have previously been seen at a clinic, and participants randomised into the drug trial from clinics that can only offer the control treatment pathway including both new clinic patients and those who have previously been seen at a clinic. A multi-level model for FAS, adjusting for baseline, with gender and pathway (cluster treatment pathway, drug only patient seen at a clinic before, drug only patient new) as fixed effects and clinic as a random effect, will be applied to the drug trial data. The population of all participants of the cluster trial who are randomised into the drugs trial will be used to study the effects of the drugs in combination/interaction with the pathways although it is recognised that the power for this analysis will be lower than originally planned. A multi-level model for FAS, adjusting for baseline, with gender as a fixed effect and clinic and PCN as random effects, will be applied to data from all participants of both the cluster trial and the drug trial to evaluate effects of Coverscan™, digitally enabled community rehabilitation, and their interaction, in combination with the participants' allocated drug treatment. For all drug treatments, particularly any introduced after the start of the trial, comparisons with participants randomised to usual care will include only those participants who could have been randomised to the relevant drug treatment at the time of their randomisation. This will reduce the potential for bias and have the effect of ensuring that only contemporaneous participants allocated to usual care are compared to participants randomised to drug treatments.

Patterns of missingness will be summarised. In modelling, complete case analysis will be the main analysis. For missing outcome data, we will use a threshold approach as a sensitivity analysis. Imputation based on thresholds will be implemented such that missing values will be replaced by values from the lower quantiles of the distribution, e.g., 25th quartile, 10th quartile, and minimum. Further details will be provided in the Statistical Analysis Plan.

14.8. Secondary Outcome Analysis

Secondary outcomes on an interval scale will be analysed similarly to the primary outcome, adjusting for baseline outcome measure, and clinic and PCN as random effects. The principal analysis set for estimates of effects of Coverscan™, digitally enabled community rehabilitation, and their interaction, on these secondary outcomes (EQ-5D-5L, GAD-7, PHQ-9, WSAS, PDQ-5, CFQ, SF-12, MRC Dyspnoea score) being participants who consented to data collection for the cluster RCT regardless of participation in the drug trial or treatment allocation in the drug trial. Where appropriate, alternative methods will be applied to outcomes with skewed distributions. Secondary outcomes on an interval scale will be compared between drug treatment arms using the same methods as for the primary outcome, using a multi-level model for the relevant outcome, adjusting for baseline, with gender and pathway as fixed effects and clinic as a random effect, within the control treatment pathway. The principal analysis population for secondary outcomes in the drug trial will be the same as for the primary outcome.

Patterns of missingness will be summarised. In modelling, complete case analysis will be done as the main analysis. For missing outcome data, we will use a threshold approach to imputation and sensitivity analyses.

14.9. Sensitivity and other planned analysis

If the Living with COVID Recovery™ application is found to be beneficial, further exploratory analyses will explore factors associated with greater benefit. Some participants allocated to this application will not be

able to access it as they lack a suitable mobile phone or tablet. Other participants will have suitable access but may choose to interact with it on a more or less extensive basis. Amount of access will be available as a patient reported variable and as summary variable from within the application.

If Coverscan™ is found to be beneficial, further exploratory analyses will explore factors associated with greater benefit. Only some of those participants allocated to this scan will choose to accept the offer. An analysis of the primary outcome will be carried out restricted to those receiving the scan vs those not allocated to the scan. If Coverscan™ is found to be beneficial, summary data on the number (and identity) of organs impaired identified by Coverscan™ will be presented and used to identify those patients in whom the effects appear to be the greatest.

Subgroup analyses of clinical sub-phenotype, including (but not limited to) cardiorespiratory, neuropsychiatric and "mast-cell activation type" (including rashes, joint pain and gastrointestinal disturbances and serositis symptoms) will be included in the Statistical Analysis Plan. These subgroups are not mutually exclusive as some participants may belong to more than one subgroup depending on their symptoms.

14.10. Interim Analysis

Given the changes in the design to partially decouple the cluster and drug trials and the revision of the analysis populations for both trials, no interim analyses will be completed for the cluster trial. The final analysis at the end of the cluster trial will be at the conventional 0.05 two-sided level. There are practical and operational reasons why a single pathway cannot graduate and become the standard pathway within the recruitment period of this trial.

The interim analyses were expected to occur based on numbers of participants recruited into the drug trial rather than the cluster trial. However, given the lower than expected recruitment within the original recruitment period and the lack of opportunity to introduce new drug arms before the end of recruitment, no interim analysis is expected before the end of recruitment of participants in July 2024.

For a drug to graduate early and become part of the backbone treatment of the trial, its two-sided p-value at the interim time point was to be ≤ 0.005. The final analysis for each drug will be with at least 200 participants in the active arm and 200 concurrent controls, and will use the conventional 2-sided 0.05 level. This analysis has 85% power if Cohen's D is 0.3 and 97% power if Cohen's D is 0.4.

14.11. Exploratory Analysis

14.11.1. Proteome Statistical Analysis

Preliminary proteomics data on a subset of participants, will be used to compare with de-identified imaging metrics to investigate the link between organ impairment found by Coverscan and the proteome. This analysis will be conducted by Perspectum in collaboration with UCL. A multi-organ reference range for all organ imaging abnormalities will be calculated and participants will then be grouped according to the number (and identity) of organs impaired. The proteome of these groups will then be compared.

- ANOVA and t-test between different groups (impaired organ -vs- unimpaired) to compare mean NPX (OLINK normalized protein concentration) values

- Differences in expression of proteins between groups will be linked to biological function by conducting pathway and overrepresentation analysis
- Clustering analysis (K-means) will be used to identify clusters and patterns within the proteome data
- Findings will be summarised and visualised using appropriate plots, e.g. volcano for t-tests, principal component analysis (PCA) and boxplots.
- Regression analysis will be performed and summarised using, e.g. Orthogonal partial least squares (OPLS) and linear models

14.11.2. Investigation into the pathophysiology of Long COVID through reverse translation

In parallel, samples from UCLH participants analysed at the Francis Crick Institute as part of exploratory endpoint 4.5.2 will be used to generate hypotheses for further discovery research in Long COVID and to define therapeutic targets for future clinical trials.

- High throughput proteomics

Plasma proteins will be compared across clusters stratified by fatigue severity (FAS score) using multinomial regression with sparse group lasso penalty. Potential confounders such as age, body-mass index and number of comorbidities will be included as covariates. For nonzero features, statistical significance will be defined as a univariate P value of less than 0.1, after Benjamini-Hochberg Procedure adjustment for multiple testing, with protein expression as the dependent variable. The resulting proteins will then be cross referenced against data generated using the O-LINK platform and published biomarker candidates/protein signatures for validation. If required, a further independent dataset will be identified for external validation of the findings. This analysis will be undertaken by a biostatisticians at the CRICK Laboratory in accordance with an agreed statistical analysis plan. Samples falling within proteomic clusters that pertain to symptom severity/phenotype, particularly fatigue severity will used as the basis for additional exploratory/discovery work on the pathophysiology of Long COVID at the Francis Crick Institute in the future.

- Serology including live-virus neutralisation.

Serum from STIMULATE participants will be tested for the presence of anti-SARS-CoV-2 neutralising antibodies against different variants as a measure of immunity to SARS-CoV-2. These data will be linked to symptoms and time since infection, and used to support the design of future interventions for Long COVID.

15. END OF TRIAL

The study is deemed to have ended following the last data collection point within the trial.

The CI and/or TSC have the right at any time to terminate the trial for clinical or administrative reasons. The funder may withdraw funding in the event of futility, study misconduct, or other unanticipated events. In such instances, the LCTU on behalf of Sponsor will notify the MHRA within 15 days.

The end of the trial will be reported to the REC and Regulatory Authority within the required timeframe if the trial is terminated prematurely. Investigators will inform patients of any premature termination of the trial and ensure that the appropriate follow up is arranged for all involved.

Following the end of the trial a summary report of the trial will be provided to the REC and Regulatory Authority within the required timeframe. LCTU on behalf of the Sponsor will notify MHRA at the end of the clinical trial within 90 days of its completion.

16. RECORD KEEPING AND ARCHIVING

At the end of the trial, all essential documentation and the trial dataset will be prepared for archiving and transfer to the Sponsor organisation by LCTU. Participating sites will be asked to archive for a minimum of 25 years from the declaration of end of trial. Essential documents are those which enable both the conduct of the trial and the quality of the data produced to be evaluated and show whether the site complied with the principles of Good Clinical Practice and all applicable regulatory requirements.

The Sponsor will notify sites when trial documentation can be archived. All archived documents must continue to be available for inspection by appropriate authorities upon request.

17. OVERSIGHT COMMITTEES

There are three main trial management groups which are involved in the set up and management of STIMULATE-ICP.

The roles and responsibilities for each of these committees are outlined in the terms of reference charter for each committee and are provided in separate documents. Membership of each committee is dependent on accepting the terms of reference and declaration of conflict of interest. Copies of the signed copies of the terms of reference, and the declaration of conflict of interest will be stored in the TMF.

17.1. Trial Management Group (TMG)

The TMG will include the Chief Investigator and trial staff. The TMG will be responsible for overseeing the trial. Representatives from public and patient engagement program is a member of the TMG group.
The TMG will review Recruitment figures, SAEs and substantial amendments to the protocol prior to submission to the REC and/or MHRA. All PIs will be kept informed of substantial amendments through their nominated responsible individuals.

The Trial Management Group should meet regularly on a monthly basis, to ensure all practical details of the trial are progressing well and working well and everyone within the trial understands them and will send updates to the PIs.

Members of the TMG must abide by the TMG membership charter which is supplied to the members in a separate document and must also declare any conflict of interest before admission to the group.

17.2. Trial Steering Committee (TSC)

The role of the TSC is to provide overall supervision of the trial. This includes members of the Trial Management Group, plus independent members not otherwise involved in the trial, including the chair of the TSC. The TSC will advise the TMG, particularly with a view to major trial issues, and will review the recommendations of the (Independent) Data Monitoring Committee and, on consideration of this

information, recommend any appropriate amendments/actions for the trial as necessary. The TSC should consider the requirements of the Sponsor and funder(s) as well as the trial and its participants and potential future participants.

17.3. Independent Data Monitoring Committee (IDMC)

The role of the IDMC is to provide independent advice on accumulating comparative data and safety aspects of the trial. The Committee will meet before the trial starts recruiting and then every 12 weeks, until the end of data analysis, to review the trial conduct and data from interim analyses, or as necessary to address any issues. The IDMC is advisory to the TSC and Sponsor and can recommend premature closure of the trial to the TSC.

During the study, interim analyses of all study data were to be supplied in strict confidence to the IDMC. However, the plan for interim analyses was removed through oversight consultation due to changes in the total recruitment target. The IDMC will schedule reviews/meetings according to the emerging data from this and other studies. The IDMC will independently evaluate these analyses and any other information considered relevant. The IDMC will determine if, in their view, the randomised comparisons in the study have provided evidence on mortality that is strong enough (with a range of uncertainty around the results that is narrow enough) to affect treatment strategies. In such a circumstance, the IDMC will inform the TSC who will make the results available to the Sponsor and, if deemed acceptable by the Sponsor and the funder, amend the trial arms accordingly.

17.4. Stopping Rules

The trial may be stopped before completion for the following reasons:

- On the recommendation of the TSC or IDMC (see section 13.3)
- On the recommendation of the Sponsor and CI

All safety data will be reviewed and a decision on continuation will be made by the IDMC with input from the Sponsor.
A single arm of the drug study may be stopped by the IDMC on the grounds of harm or futility at each stopping point.

Additionally, the IDMC may recommend a single arm of the drug study be halted in the event of the death of a single participant that is directly attributable to the study drug.

18. MONITORING, AUDIT, AND INSPECTION

The Sponsor has delegated the responsibility for monitoring the STIMULATE-ICP trial to the LCTU. An appropriate level of monitoring will be assigned to the trial in conjunction with the outcome of the trial Risk Assessment. Risk will be assessed on an ongoing basis and adjustments will be made accordingly.

The degree of monitoring will be proportionate to the objectives, purpose, phase, design, size, complexity, endpoints and risks associated with the trial. A Monitoring Plan will be prepared by the LCTU monitoring

team, in which the frequency and extent and nature of the monitoring will be outlined, and the trial will be monitored in accordance with this agreed plan.

18.1. Direct Access to Source Data

Each participating site should maintain appropriate medical and research records for this trial, in compliance with International Conference on Harmonisation E6 Good Clinical Practice Guidelines Section 4.9 and regulatory and institutional requirements for the protection of confidentiality of participants. The investigator(s)/institution(s) will permit trial-related monitoring, audits, REC review, and regulatory inspection(s), providing direct access to source data/documents. Trial participants are informed of this during the informed consent discussion. Participants will consent to provide access to their medical notes.

Source data will be identified and recorded in the trial Monitoring Plan.

19. ETHICS AND REGULATORY REQUIREMENT REPORTING

The LCTU will ensure that the trial protocol and associated documents, have been approved by the regulatory bodies (HRA and MHRA). Before the start of the trial, approval will be sought from a REC for the trial protocol and other relevant documents e.g. Participant Information Sheet, advertisements and GP information letters. The protocol, all other supporting documents including any agreed amendments, will be documented and submitted for ethical and regulatory approval as required. Amendments will not be implemented prior to receipt of the required approval(s).

Before the sites may be opened to recruit participants, LCTU must receive NHS permission, in writing, from the Trust Research & Development (R&D) to confirm capability and capacity. It is the responsibility of the LCTU to ensure that all subsequent amendments gain the necessary approvals that these are relayed to the sites and confirmation of update into SIF received. This does not affect the individual clinician's responsibility to take immediate action if thought necessary to protect the health and interest of individual participants (see section for reporting urgent safety measures).

Within 90 days after the end of the trial, the CI/Sponsor will ensure that the main REC and the MHRA are notified that the trial has finished. If the trial is terminated prematurely, those reports will be made within 15 days after the end of the trial.

Substantial amendments that require review by REC will not be implemented until the REC and MHRA grants a favourable opinion for the trial and/or NHS R&D department before they can be implemented in practice at participating sites.

All correspondence with the REC /MHRA will be retained in the Trial Master File.

19.1. Peer Review

This protocol has been reviewed by independent experts and the reviews were proportionate:

a) The Proposal underwent peer review as part of the NIHR funding application process.

b) At least two individual experts have reviewed the protocol and the funding application. The definition of independent used here is that the reviewers were external to the Chief Investigators' host institution and not involved in the trial in any way. Reviewers were not anonymous.

c) The protocol has been reviewed by experts in statistical analysis and clinical trials methodology.

19.2. Public and Patient Involvement

The research proposal has been enriched by existing robust patient and public involvement and engagement (PPIE) using multiple channels, including regular updates and webinars (Coverscan™ study), surveys (Long COVID SOS), social media (direct engagement and indirect analysis), and working with ethnic minorities. The proposal for this protocol has been informed by existing engagements with patients in research, clinical practice, policy such as RCGP PPI unit, lived experience or through organisations, such as Long COVID SOS and UK Doctors #Long COVID. Institutions and collaborators have established PPIE systems which will be involved throughout.

Patients have been central in setting research questions, designing, writing and organisation, including focus on care pathways, improved diagnostics, pragmatic trials, new treatments, health inequalities and mental health.

Members of the PPIE will be engaged with the trial conduct and review the trial documentations, assist with amendments and publications of the patient facing documents before submission for regulatory approval. They also assist with review and feedback on the trial literature, adverts and for the purpose of recruitment and enrolment of the participants in the trial. Public and patients will be involved in other stakeholder engagement and the ultimate policy recommendation on publication including the dissemination plans.

19.3. Regulatory Compliance

The trial will not commence until a Clinical Trial Authorisation (CTA) is obtained from the MHRA and favourable REC opinion. The protocol and trial conduct will comply with the Medicines for Human Use (Clinical Trials) Regulations 2004 (SI.2004/1031) and any relevant amendments as well as all applicable national and local laws and regulations.

19.3.1. Protocol Compliance

Prospective, planned deviations or waivers to the protocol are not allowed under the UK regulations on Clinical Trials and must not be used, e.g., it is not acceptable to enrol a participant if they do not meet the eligibility criteria or restrictions specified in the trial protocol.

Accidental protocol deviations can happen at any time. They will be adequately documented on the relevant forms and reported to the LCTU and Sponsor as per Sponsor SOP for the Recording & Reporting of Deviations, Violations, Potential Serious breaches, Serious Breaches and Urgent Safety Measures (SPON/S15).

Deviations from the protocol which are found to frequently recur are not acceptable, will require immediate action and could potentially be classified as a serious breach.

19.3.2. Notification of Serious Breaches to GCP and Protocol

A "serious breach" is a breach which is likely to affect to a significant degree –
(a) the safety or physical or mental integrity of the participants of the trial; or
(b) the scientific value of the trial.

The LCTU and Sponsor will be notified immediately of any case where the above definition applies during the trial conduct phase as per the Sponsor SOP for the Recording & Reporting of Deviations, Violations, Potential Serious breaches, Serious breaches, and Urgent Safety Measures. The Sponsor will notify the licensing authority in writing of any serious breach of:

(a) the conditions and principles of GCP in connection with the trial; or
(b) the protocol relating to the trial, as amended from time to time, within 7 days of becoming aware of that breach.

19.3.3. Safety Reporting

SAE and SUSAR reporting to the Sponsor, IDMC and regulatory authorities will be done according to the details outlined in Section 11.3 and 11.6 of this protocol.

19.3.4. Annual Progress Report

An annual progress report (APR) will be submitted to the REC within 30 days of the anniversary date on which the favourable opinion was given, and annually until the trial is declared ended. APR is produced by the LCTU, but it is the Chief Investigator's responsibility to review and authorise the release of the annual reports as required.

19.3.5. End or Trial Report

At the end of the trial the CI will supply the Sponsor with a clinical trial report and upload the trial results to all applicable trial registries within 12 months of the end of trial declaration date. A copy of this report will also be submitted to the REC and MHRA (as required).

19.4. Data Protection and Participant Confidentiality

All data will be handled in accordance with the Data Protection Act (2018). Participant confidentiality will be maintained, and the trial will be compliant with the requirements of the Act.

Electronic and paper Case Report Forms will be labelled with unique patient identifier allocated upon randomisation/registration. Medical information may be given to the participant's medical team and all appropriate medical personnel responsible for the participant's welfare. UCL and LCTU will preserve the confidentiality of participants taking part in the trial.

UCL as Sponsor is the Data Controller as defined under the Data Protection Act 2018. The University of Central Lancashire is acting as a data processor when handling participants data.

All investigators and trial site staff must comply with the requirements of the Data Protection Act with regards to the collection, storage, processing, and disclosure of personal information and will uphold the Act's core principles.

Personal information collected by the participating site staff will be kept secure and maintained which involves:

- Each participant is given a coded number to replace the participant's identifiable information.
- The information which links the patient identifiable information and the randomisation code will be kept secure and kept in separate locations using encrypted digital files within password protected folders and storage media.
- Access will be limited to the minimum number of individuals necessary for quality control, audit, and analysis.
- Data transmitted to the Sponsor in pseudonymised fashion, with all patient identifiable information removed prior to transfer.

The participant's initials, date of birth and trial identification number will be used for identification, and this will be clearly explained to the patient in the Patient Information Sheet. Patient consent for this will be sought.

Any breach of participants personal data (e.g., name or date of birth being sent to an unauthorised individual) must be reported to the LCTU immediately or within 24 hours of the site become aware of the incidence. LCTU will notify the Sponsor within 24 hours of becoming aware of the incidence for follow-up and reporting (if applicable) as part of data controller responsibilities. The data protection office of both organisations will determine the outcome of the breach and necessary root cause analysis and actions points will be put in place to prevent such recurrence.

The Case Report Forms (CRFs) will not bear the participant's name or other personal identifiable data.

The following personal identifiable information will be collected from patients and sites by LCTU to enable contact with the patient and follow-up of questionnaires and data where required, this will be specified within the participant information sheet and consent form:

- Consent forms (containing patient name and signature)
- NHS number
- Address
- Name
- DOB
- Telephone
- Email

- Contacting patients

The blood samples gifted by the participants for research and storage will be sent to Perspectum (collaborator) without patient identifiable information, for analysis by external laboratories mentioned in section 9.6. The manner by which the CRFs and the blood samples are handled will be clearly described in the Participant Information Sheet and Consent Form.

19.5. Financial and other competing interests for the Chief Investigator & PIs at each site

The trial is funded by a grant from the National Institute for Health Research.

Professor Amitava Banerjee is Chief Investigator for the STIMULATE-ICP clinical trial and has no relevant conflicts of interest in relation to the conduct and analysis of data for this trial. He has received unrelated research grants from AstraZeneca Pharmaceuticals, NIHR, British Medical Association and other funding organisations. He is Trustee of the South Asian Health Foundation.

Dr Rajarshi Banerjee is CEO of Perspectum which developed Coverscan™. Perspectum is providing the Coverscan™ intervention in this trial, central laboratory services and sample analysis and subsequent reporting of study data to Sponsor and Sites. Perspectum will create and maintain a sample biobank for future research for which they will have the right to agree a licence to access samples for their own commercial and research use. Perspectum are receiving funding from the NIHR grant to part cover scanning services, but the provision of central laboratory services and some scanning services will be provided in kind.

Professor Elizabeth Murray leads an NIHR grant which is evaluating "Living with COVID Recovery™" (https://www.ucl.ac.uk/healthcare-engineering/covid-19/covid-19-engineering-research/living-covid-recovery), and is not involved in trial conduct, data collection or analysis.

19.5.1. Payments to participants

The funding for the trial does not include reimbursement for participant time and travel to the Long COVID clinics. The appointment to Coverscan™ and at the Long COVID clinics are usual care, as such no funding is available to support participants in the trial. In areas where support to attend appointments is available e.g. volunteer patient services these will be accessed.

19.6. Insurance and Indemnity

The Sponsor (University College London) is an educational establishment and holds insurance against claims from participants for injury caused by their participation in the clinical trial. Participants may be able to claim compensation if they can prove that UCL has been negligent. However, as this clinical trial is being carried out at NHS sites, the NHS sites continue to have a duty of care to the participant of the clinical trial. University College London does not accept liability for any breach in the NHS duty of care, or any negligence on the part of NHS employees. This applies whether the hospital is an NHS Trust or otherwise.

Participants may also be able to claim compensation for injury caused by participation in this clinical trial without the need to prove negligence on the part of University College London or another party. Participants who sustain injury and wish to make a claim for compensation should do so in writing in the

first instance to the Chief Investigator, who will pass the claim to the Sponsor's Insurers, via the Sponsor's office.

Hospitals and Long COVID clinics selected to participate in this clinical trial shall provide NHS standard indemnity against clinical negligence for harm caused by their employees and a copy of the relevant insurance policy or summary shall be provided to University College London, upon request.

20. PUBLICATION & DISSEMINATION

20.1. Publication Policy

CONSORT Guidelines and checklist will be reviewed prior to generating any publications for the trial to ensure they meet the standards required for submission to high quality peer reviewed journals etc. http://www.consort-statement.org/

Following completion of the study, the Chief Investigator is expected to publish the results of this research in a scientific journal. The International Committee of Medical Journal Editors (ICMJE) member journals have adopted a trials-registration policy as a condition for publication. This policy requires that all clinical trials be registered in a public trials registry. This trial has been registered with ClinicalTrials.gov, which is sponsored by the National Library of Medicine.

Trial investigators have the right and responsibility to separately communicate their findings to the scientific community and to the public. All such publications shall adhere to the publication policy detailed in LCTU SOP TM-05 (Trial Reporting), must not be before the reporting of the main results, and will be submitted, at least 30 days prior to submission, for review by the Sponsor, all appropriate Trial Committees and Funder.

The objectives of the publication policy are:

1. To assure and expedite orderly and timely presentations to the scientific community of all pertinent data resulting from the trial
2. To assure scientifically accurate presentation and papers from investigators
3. To assure that all investigators, particularly those of junior rank, have the opportunity to participate and be recognized in the trial-wide presentations and publications
4. To assure that press releases, interviews, presentations, and publications of trial materials are accurate and do not compromise the scientific integrity of this collaborative trial
5. To establish procedures that allows the Trial Steering Committee and the Independent Data and Safety Monitoring Board to review publications and presentations in a timely manner
6. To maintain a complete up-to-date list of presentations and publications, and to distribute such lists to all investigators.
7. To clarify and ensure proper acknowledgement of National Institutes for Health Research and non-National Institutes that supports the trial including all collaborator institutions.

20.2. Open Access and Data Sharing

This trial is funded by NIHR and as a condition of funding, publications from the outcome of the trial must be made accessible through publications in peer reviewed and open access journals.

The trial will adhere to the NIHR Open Access Policy regarding accessibility and data sharing. Details of the policy is published and updated on the NIHR website; https://www.nihr.ac.uk/documents/nihr-open-access-policy/28999. The policy does not require that the data must be made open.

The findings from STIMULATE-ICP will be published in journals that makes the analysis available using Creative Commons Attribution (CCBY) licences and allow depositing of the final published version in public repositories without restriction on re-use. A copy of the final manuscript will also be deposited with Europe PMC (https://europepmc.org/) upon acceptance for publication which will be made freely available as soon as possible and in any event within 6 months of the Journal publisher's official date of final publication.

Sharing of the trial data must protect the confidentiality and privacy of participants; respect the terms of consent by participants who are involved in the trial; be consistent with relevant legal, ethical and regulatory frameworks; and guard against unreasonable costs.

Release of the anonymized trial data will be subject to data sharing agreement between the Sponsor, CI and the third party requesting the data. The integrity of the data must always be preserved, and the agreement should be aligned with the Sponsor and LCTU SOPs on data sharing.

20.3. Authorship Eligibility

The trial will follow the recommendations for the Conduct, Reporting, Editing, and Publication of Scholarly Work in Medical Journals by the International Committee of Medical Journal Editors (ICMJE) in relation to authorship credit that should be based only on:

1. Substantial contributions to the conception or design of the work; or the acquisition, analysis, or interpretation of data for the work; AND
2. Drafting the work or revising it critically for important intellectual content; AND
3. Final approval of the version to be published; AND
4. Agreement to be accountable for all aspects of the work in ensuring that questions related to the accuracy or integrity of any part of the work are appropriately investigated and resolved.

All those designated as authors should meet all four criteria for authorship and all those who meet the four criteria should be identified as authors. All those who do not meet all four criteria should be acknowledged.

Full details of the recommendation are available on (http://www.icmje.org/).

21. REFERENCES

1. C1248-national-guidance-post-covid-syndrome-assessment-clinics-v2.pdf.
2. Campbell H et al. Integrated care pathways. BMJ. 1998.pdf.
3. Office of National Statistics. <Prevalence of ongoing symptoms following coronavirus (COVID-19) infection in the UK 5 August 2021.pdf>. 2021.
4. Al-Aly Z, Xie Y, Bowe B. High-dimensional characterization of post-acute sequelae of COVID-19. Nature. 2021;594(7862):259-64.
5. Mandal S, Barnett J, Brill SE, Brown JS, Denneny EK, Hare SS, et al. 'Long-COVID': a cross-sectional study of persisting symptoms, biomarker and imaging abnormalities following hospitalisation for COVID-19. Thorax. 2021;76(4):396-8.
6. Maxwell E. Living-with-Covid-Themed-Review-October-2020.pdf. 2020.
7. R H. <Offline COVID-19 is not a pandemic Lancet.pdf>. Lancet. 2020;396(874).
8. Banerjee A, Pasea L, Harris S, Gonzalez-Izquierdo A, Torralbo A, Shallcross L, et al. Estimating excess 1-year mortality associated with the COVID-19 pandemic according to underlying conditions and age: a population-based cohort study. The Lancet. 2020;395(10238):1715-25.
9. Greenhalgh T, Knight M, A'Court C, Buxton M, Husain L. Management of post-acute covid-19 in primary care. BMJ. 2020;370:m3026.
10. NICE. <covid19-rapid-guideline-managing-covid19>. 2021.
11. Jacobson TA, Smith LE, Hirschhorn LR, Huffman MD. Using implementation science to mitigate worsening health inequities in the United States during the COVID-19 pandemic. Int J Equity Health. 2020;19(1):170.
12. Glasgow. <The RE-AIM framework for evaluating interventions what canit tell us about approaches to chronic illness management.pdf>. 2001.
13. McNulty M, Smith JD, Villamar J, Burnett-Zeigler I, Vermeer W, Benbow N, et al. Implementation Research Methodologies for Achieving Scientific Equity and Health Equity. Ethn Dis. 2019;29(Suppl 1):83-92.
14. Moore GF, Audrey S, Barker M, Bond L, Bonell C, Hardeman W, et al. Process evaluation of complex interventions: Medical Research Council guidance. BMJ. 2015;350:h1258.
15. Dennis A, Wamil M, Alberts J, Oben J, Cuthbertson DJ, Wootton D, et al. Multiorgan impairment in low-risk individuals with post-COVID-19 syndrome: a prospective, community-based study. BMJ Open. 2021;11(3):e048391.

16. Heightman M, Prashar J, Hillman TE, Marks M, Livingston R, Ridsdale H, et al. Post-COVID assessment in a specialist clinical service: a 12-month, single-centre analysis of symptoms and healthcare needs in 1325 individuals. medRxiv. 2021:2021.05.25.21257730.
17. Carson G. Research priorities for Long Covid: refined through an international multi-stakeholder forum. BMC Med. 2021;19(1):84.
18. Mandal S, Barnett J, Brill SE, Brown JS, Denneny EK, Hare SS, et al. 'Long-COVID': a cross-sectional study of persisting symptoms, biomarker and imaging abnormalities following hospitalisation for COVID-19. Thorax. 2021;76(4):396-8.
19. Society R. Long Covid what is it and what is needed.pdf.
20. Ayoubkhani D, Khunti K, Nafilyan V, Maddox T, Humberstone B, Diamond I, et al. Post-covid syndrome in individuals admitted to hospital with covid-19: retrospective cohort study. BMJ. 2021;372:n693.
21. Davis HE, Assaf GS, McCorkell L, Wei H, Low RJ, Re'em Y, et al. Characterizing long COVID in an international cohort: 7 months of symptoms and their impact. EClinicalMedicine. 2021:101019.
22. Noor NM, Pett SL, Esmail H, Crook AM, Vale CL, Sydes MR, et al. Adaptive platform trials using multi-arm, multi-stage protocols: getting fast answers in pandemic settings. F1000Res. 2020;9:1109.
23. Parmar MKB, Carpenter J, Sydes MR. More multiarm randomised trials of superiority are needed. The Lancet. 2014;384(9940):283-4.
24. Parmar MK, Barthel FM, Sydes M, Langley R, Kaplan R, Eisenhauer E, et al. Speeding up the evaluation of new agents in cancer. J Natl Cancer Inst. 2008;100(17):1204-14.
25. Parmar MK, Sydes MR, Cafferty FH, Choodari-Oskooei B, Langley RE, Brown L, et al. Testing many treatments within a single protocol over 10 years at MRC Clinical Trials Unit at UCL: Multi-arm, multi-stage platform, umbrella and basket protocols. Clin Trials. 2017;14(5):451-61.
26. Heightman M, Prashar J, Hillman TE, Marks M, Livingston R, Ridsdale H, et al. Post-COVID assessment in a specialist clinical service: a 12-month, single-centre analysis of symptoms and healthcare needs in 1325 individuals. 2021.
27. Mura C, Preissner S, Nahles S, Heiland M, Bourne PE, Preissner R. Real-world evidence for improved outcomes with histamine antagonists and aspirin in 22,560 COVID-19 patients. Signal Transduction and Targeted Therapy. 2021;6(1):267.
28. Glynne P, Tahmasebi N, Gant V, Gupta R. Long-COVID following mild SARS CoV-2 infection: characteristic T cell alterations and response to antihistamines. medRxiv. 2021:2021.06.06.21258272.

29. Malone RW, Tisdall P, Fremont-Smith P, Liu Y, Huang XP, White KM, et al. COVID-19: Famotidine, Histamine, Mast Cells, and Mechanisms. Res Sq. 2020.
30. Malone RW. More Than Just Heartburn: Does Famotidine Effectively Treat Patients with COVID-19? Dig Dis Sci. 2021;66(11):3672-3.
31. Terman JM, Awsumb JM, Cotler J, Jason LA. Confirmatory factor analysis of a myalgic encephalomyelitis and chronic fatigue syndrome stigma scale. J Health Psychol. 2020;25(13-14):2352-61.
32. Broz P, Dixit VM. Inflammasomes: mechanism of assembly, regulation and signalling. Nat Rev Immunol. 2016;16(7):407-20.
33. Dalbeth N, Lauterio TJ, Wolfe HR. Mechanism of action of colchicine in the treatment of gout. Clin Ther. 2014;36(10):1465-79.
34. Adler Y, Charron P, Imazio M, Badano L, Baron-Esquivias G, Bogaert J, et al. 2015 ESC Guidelines for the Diagnosis and Management of Pericardial Diseases. Rev Esp Cardiol (Engl Ed). 2015;68(12):1126.
35. Kotecha T, Knight DS, Razvi Y, Kumar K, Vimalesvaran K, Thornton G, et al. Patterns of myocardial injury in recovered troponin-positive COVID-19 patients assessed by cardiovascular magnetic resonance. Eur Heart J. 2021.
36. Malas MB, Naazie IN, Elsayed N, Mathlouthi A, Marmor R, Clary B. Thromboembolism risk of COVID-19 is high and associated with a higher risk of mortality: A systematic review and meta-analysis. EClinicalMedicine. 2020;29:100639.
37. Marco A, Marco P. Von Willebrand factor and ADAMTS13 activity as clinical severity markers in patients with COVID-19. J Thromb Thrombolysis. 2021.
38. Indraratna PL, Virk S, Gurram D, Day RO. Use of colchicine in pregnancy: a systematic review and meta-analysis. Rheumatology (Oxford). 2018;57(2):382-7.
39. UCL. New rehabilitation app to aid recovery of COVID-19 ‘long-haulers’. : UCL; 2020 [cited 2020. Available from: https://www.ucl.ac.uk/news/2020/aug/new-rehabilitation-app-aid-recovery-covid-19-long-haulers.
40. de Kleijn WP, De Vries J, Wijnen PA, Drent M. Minimal (clinically) important differences for the Fatigue Assessment Scale in sarcoidosis. Respir Med. 2011;105(9):1388-95.
41. Davis HE, Assaf GS, McCorkell L, Wei H, Low RJ, Re’em Y, et al. Characterizing Long COVID in an International Cohort: 7 Months of Symptoms and Their Impact. 2021.
42. <2020_09_HMA_CTFG_Contraception_guidance_Version_1.1_updated.pdf>.
43. Trussell J. Contraceptive failure in the United States. Contraception. 2011;83(5):397-404.

CONFIDENTIAL

APPENDICES

Appendix I

Table 10: List and frequency of important side effects from the trial IMPs and mitigating risk plans
Very common > 1/10, Common > 1/100 and < 1/10, Uncommon > 1/1000 and < 1/100, Rare > 1/10 000 and < 1/1000, Very rare < 1/10 000, Not known (cannot be estimated from the available data)

Sources: SmPC Clarityn (Loratadine) 10mg tablets (Bayer plc)
SmPC Famotidine 40mg tablets (Tillomed Laboratories Ltd)
SmPC Colchicine Tablets BP 500 mcg (Wockhardt UK Ltd)
SmPC Xarelto (Rivaroxaban) 10 mg film-coated tablets (Bayer plc)

System Organ Class	Adverse Reaction and Frequency	Clinical risk	Symptoms to report	Management
**Loratadine 10mg tablets**				
Immune system disorders	Very rare: Hypersensitivity reactions (including angioedema and anaphylaxis)	Medically serious = anaphylaxis & angioedema	Itch, swelling, shortness of breath, rash	Stop drugs, dial 999 in case of anaphylactic shock reaction & follow standard anaphylactic protocols. Refer to ED if evidence of oedema

Nervous system disorders	Very rare: Dizziness, convulsion	Medically important (convulsion)	Seizure, absence attack, associated with memory loss, incontinence, and possible injury	Stop drugs, refer to ED if acute evidence of seizure, refer to neurology if seizures suspected but not witnessed
Cardiac disorders	Very rare: Tachycardia, palpitation	Minor	Dizziness, palpitations	Consider stop drugs, GP review, check ECG & blood pressure
Gastrointestinal disorders	Very rare: Nausea, dry mouth, gastritis	Minor	Nausea, drug mouth, abdominal pain, acid reflux	Symptomatic management, stop drugs if intolerable
Hepatobiliary disorders	Very rare: Abnormal hepatic function	Important	Fatigue, fever, weakness, malaise	Check liver function, if ALT >5 times upper limit of normal stop drugs and refer to SDEC for review
Skin and subcutaneous tissue disorders	Very rare: Rash, alopecia	Minor	Rash, hair loss	Stop drugs, monitor, consider dermatology referral if persistent after stopping drugs
General disorders	Very rare: Fatigue Not known: Weight increased	Minor	Fatigue, weight gain	Symptomatic management, stop drugs if intolerable

**Famotidine 40mg tablets**				
**System Organ Class**	**Adverse Reaction and Frequency**	**Clinical risk**	**Symptoms to report**	**Management**

Blood and lymphatic systems	Very rare: Thrombocytopenia, Leukopenia, Agranulocytosis, pancytopenia, neutropenia	Medically serious	Fatigue, pallor, weakness, postural hypotension, easy bruising, recurrent infections	Stop drugs, needs urgent blood tests (FBC, clotting), urgent/same day review by haematologist if abnormalities detected
Psychiatric Disorders	Very rare: Reversible psychic disturbances including Hallucinations, Disorientation, Confusion, Anxiety disorders, Agitation, Reduced libido, Insomnia	Medically serious	As per side effect list	Exclude alternative causes, consider stopping drug, GP to manage symptoms with support of a psychiatrist if required
Nervous systems	Common: Headache, Dizziness	Minor (headache, dizziness, taste disorder) Medically serious (Epileptic seizures/convulsions)	Headache, dizziness, abnormal taste. Seizure or convulsion, absence attack, associated with memory loss, incontinence, and possible injury	Minor symptoms nil action required, stop drugs if intolerable. If seizure suspected, stop drugs, refer to ED if acute evidence of seizure, refer to neurology if seizures suspected but not witnessed
	Uncommon: Taste disorder			
	Very rare: Paraesthesia, Somnolence, Epileptic seizures, convulsions; grand mal seizures (particularly in patients with impaired renal function);			
Gastrointestinal systems	Common: Constipation, Diarrhoea	Minor	As per side effect list	GP to manage symptomatically with OTC remedies/diet changes. Stop drugs if intolerable

	Uncommon: Nausea, Vomiting, Abdominal discomfort or distension, Flatulence, dry mouth			
Hepato-biliary disorders	Rare: Intrahepatic cholestasis (visible sign: jaundice),	Medically serious	Visible jaundice in skin or eyes, non-specific, commonly detected on blood tests done for alternative reason	Stop drugs, check liver function, clinical review by GP/SDEC/hepatology if ALT/bilirubin >5x upper limit of normal OR visibly jaundiced OR symptomatic OR patient at risk of liver disease e.g. increased alcohol consumption
	Very rare: Hepatitis; cholestatic jaundice, Increase in liver enzyme abnormalities (transaminases, gamma GT, alkaline phosphatase, bilirubin)			
Metabolism and nutrition	Uncommon: Loss of appetite (anorexia)	Minor	Loss of appetite	GP to manage symptomatically with OTC remedies/diet changes. Stop drugs if intolerable
Skin and subcutaneous tissue disorders	Uncommon: Rash, Pruritus, Urticaria	Minor (Pruritus/rash/urticaria). Medically serious (SJS/TEN)	Itch, rashes, bruising. SJS/TEN has a specific rash that resembles a target with a red ring.	Stop drugs (all), for suspected SJS/serious rash requires same day referral to dermatology (via SDEC/ED)
	Very rare: Alopecia, Stevens Johnson syndrome/toxic epidermal necrolysis sometimes fatal			
Immune System disorders	Very rare: Hypersensitivity reactions (angioneurotic oedema, anaphylaxis, bronchospasm)	Medically serious	itch, swelling, shortness of breath, rash	stop drugs, dial 999 in case of anaphylactic shock reaction & follow standard anaphylactic

				protocols. Refer to ED if evidence of oedema
Respiratory, thoracic, and mediastinal disorders	Very rare: Interstitial pneumonia sometimes fatal	Medically serious	cough, chest pain, haemoptysis	Manage as per pneumonia. Stop drugs if suspected, refer to ED/SDEC if patient requires oxygen or parenteral treatment for pneumonia
General disorders and administration site conditions:	Uncommon: fatigue	Minor	As per side effect list	For fatigue: symptomatic management, stop drugs if intolerable and alternative causes excluded.
	Very rare: chest tightness			For chest tightness: exclude alternative causes, consider stopping drug, may need ECG
Reproductive system and breast disorders:	Very rare: impotence, rare cases of gynecomastia, have been reported, however, in controlled clinical trials the incidences were not greater than those seen with placebo.	Minor	impotence, increased breast tissue in males	symptomatic management, stop drugs if intolerable and alternative causes excluded
Musculoskeletal, connective tissue and bone disorders	Very rare: Muscle cramps, Arthralgia	Minor	cramping, pain in muscles or joints	symptomatic management, stop drugs if intolerable

**Colchicine 500mcg tablets**				
**System Organ** Class	**Adverse Reaction and *Frequency***	**Clinical risk**	**Symptoms to report**	**Management**

Gastrointestinal system disorders	*Common:* Abdominal pain, nausea, vomiting and diarrhoea. *Not known:* Gastrointestinal Bleeding	GI bleeding medically serious	Vomiting blood/coffee grounds, dark stools, Fatigue, pallor, postural dizziness, fresh blood PR	Cessation of drug, same-day hospital assessment (SDEC), likely to need endoscopy +/- colonoscopy & prescription of Drugs to stop bleeding (PPI)
Skin and subcutaneous tissue disorders	*Not known:* Alopecia and rash	Minor	Rash	Stop drugs, dermatology referral if persists
Renal and urinary disorders	*Not known:* Renal damage	Medically serious	Non-specific, commonly detected on blood tests done for alternative reason	Stop drugs, check renal function, clinical review by GP/SDEC
Hepatobiliary disorders	*Not known:* Hepatotoxicity	Medically serious	Non-specific, commonly detected on blood tests done for alternative reason	Stop drugs, check liver function, clinical review by GP/SDEC
(Female) Reproductive system disorders	*Not known:* Menstrual cycle irregularities; amenorrhoea, dysmenorrhoea	Minor	Heavy periods	Stop drugs, check FBC, may need iron replacement if anaemic
Musculoskeletal system	*Not known:* Myopathy and rhabdomyolysis	Medically serious	Muscle pain & weakness	Stop drugs, check creatine kinase & renal function, clinical review by GP/SDEC,

				may need IV fluids & careful monitoring of renal function
Nervous System	*Not known:* Peripheral neuritis, neuropathy.	Important	Nerve pain or weakness	Stop drugs, clinical review by GP/SDEC, may need neurology referral
(Male) Reproductive system disorders	*Not known:* oligospermia, azoospermia.	Important	Primary infertility	Stop drugs, clinical review by andrologist
Blood and lymphatic system disorders	*Not known:* bone marrow depression with agranulocytosis, aplastic anaemia, and thrombocytopenia.	Medically serious	Fatigue, pallor, weakness, postural hypotension, easy bruising, recurrent infections	Stop drugs, needs urgent blood tests (FBC, clotting), urgent/same day review by haematologist if abnormalities detected
**Rivaroxaban 10mg tablets**				
**System Organ** Class	**Adverse Reaction and *Frequency***	**Clinical risk**	**Symptoms to report**	**Management**
Blood and lymphatic system disorders	*Common:* Anaemia (incl. respective laboratory parameters) *Uncommon:* Thrombocytosis (incl. platelet count increased), thrombocytopenia	Minor	Fatigue, pallor, weakness, postural hypotension, easy bruising	Stop drugs, check FBC, may need iron replacement if anaemic

Immune system disorders	*Uncommon:* Allergic reaction, dermatitis allergic, angioedema and allergic oedema<br>*Very rare:* Anaphylactic reactions including anaphylactic shock	Medically serious	Itch, swelling, shortness of breath, rash	Stop drugs, dial 999 in case of anaphylactic shock reaction & follow standard anaphylactic protocols. Refer to ED if evidence of oedema
Nervous system disorders	*Common:* Dizziness, headache<br>*Uncommon:* Cerebral and intracranial haemorrhage, syncope	Medically serious (intracranial haemorrhage)	severe headache, weakness of limbs, loss of speech or rapid loss of consciousness	Dial 999 report suspected stroke. Requires urgent assessment in a stroke centre
Eye disorders	*Common:* Eye haemorrhage (incl. conjunctival haemorrhage)	Medically serious	visible blood in the eye, sudden loss of vision, eye pain (unilateral)	stop drugs, refer to same day ophthalmology services
Cardiovascular disorders	*Common:* Hypotension, haematoma<br>*Uncommon:* Tachycardia	Minor	dizziness, palpitations, bruising	stop drugs, GP review, check ECG, FBC & blood pressure
Respiratory, thoracic, and mediastinal disorders	*Common:* Epistaxis, haemoptysis	Minor	visible blood either on coughing or from the nose - difficult or slow to stop with compression	stop drugs, refer to ED if bleeding not stopping for ENT review

Gastrointestinal disorders	*Common:* Gingival bleeding, gastrointestinal tract haemorrhage (incl. rectal haemorrhage), gastrointestinal and abdominal pains, dyspepsia, nausea, constipation, diarrhoea, vomiting<br>*Uncommon:* Dry mouth	GI bleeding medically serous	Vomiting blood/coffee grounds, dark stools, fatigue, pallor, postural dizziness, fresh blood PR	cessation of drug, same-day hospital assessment (SDEC), likely to need endoscopy +/- colonoscopy & prescription of drugs to stop bleeding (PPI)
Hepatobiliary disorders	*Common:* Increase in transaminases<br>*Uncommon:* Hepatic impairment, increased bilirubin, increased blood alkaline phosphatase, increased GGT<br>*Rare:* Jaundice, bilirubin conjugated increased (with or without concomitant increase of ALT), cholestasis, hepatitis (incl. hepatocellular injury)	Medically serious	non-specific, commonly detected on blood tests done for alternative reason	stop drugs, check liver function, clinical review by GP/SDEC/hepatology if ALT/bilirubin >5x upper limit of normal OR visibly jaundiced OR symptomatic OR patient at risk of liver disease e.g. increased alcohol consumption
Skin disorders	*Common:* Pruritus (incl. uncommon cases of generalised pruritus), rash, ecchymosis, cutaneous and subcutaneous haemorrhage<br>*Uncommon:* Urticaria<br>*Very rare:* Stevens-Johnson syndrome/Toxic Epidermal Necrolysis, DRESS syndrome	Minor (Priuritis) Medically serious (SJS/TEN)	Itch, rashes, bruising. SJS/TEN has a specific rash that resembles a target with a red ring.	stop drugs (all), for suspected SJS/serious rash requires same day referral to dermatology (via SDEC/ED)

Musculoskeletal and connective tissue disorders	*Common:* Pain in extremity<br>*Uncommon:* Haemarthrosis<br>*Rare:* Muscle haemorrhage<br>*Not known:* Compartment syndrome secondary to a bleeding	Medically serious	Pain in joint or leg, disproportionate pain to visible changes in limb	Stop drugs, analgesia, GP review. Requires urgent referral to SDEC/ED if haemarthrosis/haemorrhage/comparetment syndrome suspected by GP
Renal and urinary disorders	*Common:* Urogenital tract haemorrhage (incl. haematuria and menorrhagia), renal impairment (incl. blood creatinine increased, blood urea increased)<br>*Not known:* Renal failure/acute renal failure secondary to a bleeding sufficient to cause hypoperfusion	Minor (urogenital tract haemorrhage). Medically serious (renal failure/AKI)	Visible blood in urine, heavy periods, fatigue, weakness, muscle pain, itch.	Stop drugs, check renal function, clinical review by GP/SDEC if evidence of AKI. May need renal ultrasound and review by nephrologist
General disorders and administration site conditions	*Common:* Fever, peripheral oedema, decreased general strength and energy (incl. fatigue and asthenia)<br>*Uncommon:* Feeling unwell (incl. malaise)<br>*Rare:* Localised oedema	Minor	Fatigue, fever, weakness, malaise	Exclude bleeding via medical history and FBC. Exclude other causes of fever and malaise. Stop drug

Investigations	*Uncommon:* Increased LDH, increased lipase, increased amylase	Medically serious	Fatigue, fever, weakness, malaise, abdominal pain	Stop drugs, if symptomatic refer to ED/SDEC. If asymptomatic GP to exclude alternative causes of abnormal enzymes including alcohol and gallstones via history and upper abdominal ultrasound. Monitor enzymes every 48 hours, if increasing refer to ED/SDEC
Injury, poisoning and procedural complications	*Common:* Postprocedural haemorrhage (incl. postoperative anaemia, and wound haemorrhage), contusion, wound secretion<br>*Rare:* Vascular pseudoaneurysm	Medically important	pain, weakness, bleeding, oozing from wound	stop drugs, check FBC, may need iron replacement if anaemic. If pseudoaneurysm suspected requires imaging of the appropriate area and input of a vascular surgeon.

Appendix II

Definition of Acceptable Effective Contraception for this Trial adapted from the Recommendations related to contraception and pregnancy testing in clinical trials (42)

- combined (estrogen and progestogen containing) hormonal contraception associated with inhibition of ovulation [1]:
  - o oral
  - o intravaginal
  - o transdermal
- progestogen-only hormonal contraception associated with inhibition of ovulation [1]:
  - o oral o injectable
  - o implantable [2]
- intrauterine device (IUD) [2]
- intrauterine hormone-releasing system (IUS) [2]
- bilateral tubal occlusion [2]
- vasectomised partner [2,3]
- sexual abstinence [4]

- progestogen-only oral hormonal contraception, where inhibition of ovulation is not the primary mode of action
- male or female condom with spermicide [a]
- cap, diaphragm or sponge with spermicide[a]

[a]A combination of male condom with either cap, diaphragm or sponge with spermicide (double barrier methods) are also considered acceptable birth control methods
[1] Hormonal contraception may be susceptible to interaction with the IMP, which may reduce the efficacy of the contraception method.
[2] Contraception methods that in the context of this guidance are considered to have low user dependency.
[3] Vasectomised partner is a highly effective birth control method provided that partner is the sole sexual partner of the WOCBP trial participant and that the vasectomised partner has received medical assessment of the surgical success.

[4] In the context of this guidance sexual abstinence is considered a highly effective method only if defined as refraining from heterosexual intercourse during the entire period of risk associated with the study treatments. The reliability of sexual abstinence needs to be evaluated in relation to the duration of the clinical trial and the preferred and usual lifestyle of the subject.

Docusign Envelope ID: 694B9EEA-7D5D-4DDE-8CD2-98FA170FF184

University of Central Lancashire
LANCASHIRE
CLINICAL TRIALS UNIT

Trial Title: **A pragmatic, multi-centre, cluster randomised trial of an integrated care pathway with a nested, Phase III, open label, adaptive platform randomised drug trial in individuals with Long COVID**

(STIMULATE-ICP)

Trial Registration: (IRAS 1004698)

Statistical Analysis Plan (SAP)

Protocol: This document has been written based on information contained in the study protocol version (5.1) Date: (18/08/2025)

SAP version: (7.1) Date: 22/03/2026

Table of Contents

1. ADMINISTRATIVE INFORMATION 5
1.1. Title 5
1.2. Trial Registration: IRAS 1004698 5
1.3. Protocol: 5
1.4. SAP version: 6.0 5
1.5. SAP revision history 6
1.6. Names, affiliations and roles of SAP contributors 14
1.7. Signatures 15
2. INTRODUCTION 16
2.1. Background 16
2.2. Rationale for the trial 16
2.3 Trial Summary 19
2.3.1. Aim 19
2.3.2. Objectives 19
3. METHODS 20
3.1. Design 20
3.2. Interventions 22
3.3. Randomisation 22
3.4. Blinding 25
3.5. Sample size 25
3.6. Framework 27
3.7. Outcomes 27
3.7.1. Primary outcome 27
3.7.2. Secondary outcomes 27
3.8. Interim analyses, IDMC and stopping rules 29
3.9. Timing of analyses 29
3.10. Timing of assessments 30
4. STATISTICAL PRINCIPLES 35
4.1. Confidence and significance levels 35
4.2. Adherence and protocol deviations 35
4.2.1. Adherence 35
4.2.2. Adherence to treatments 35
4.2.3. Definition and summary of protocol deviations 35
4.3. Analysis populations 36
5. TRIAL POPULATION 38

5.1. Screening, recruitment, withdrawal/follow-up ... 38
Figure 3. Analysis populations represented using a Venn diagram ... 41
5.2. Eligibility ... 43
5.3. Recruitment and retention ... 45
5.4. Baseline characteristics and demographics ... 45
6. ANALYSIS ... 46
6.1. Analysis of baseline characteristics ... 46
6.2. Generation of derived variables ... 47
6.3. Primary outcome analysis ... 47
6.3.1. Analysis for the primary objective in the cluster trial ... 50
6.3.2. Analysis for the primary objective in the cluster trial restricted to those taking no drug ... 50
6.3.3. Analysis for the secondary objective in the cluster trial taking account of receipt of the ICP interventions ... 50
6.3.4. Analysis for the secondary objective in the drug trial ... 51
6.3.5. Analysis for the secondary objective in the drug and cluster trial together ... 51
6.4. Secondary outcome analysis ... 52
6.5. Sensitivity analysis ... 52
6.6. Pre-specified subgroup analyses ... 53
6.7. Interim analysis ... 56
6.8. Missing data ... 56
6.9. Safety data ... 58
6.9.1. Recording and reporting of adverse events ... 58
6.9.2. Assessing adverse events and their seriousness ... 58
6.9.3. Assessing Serious Adverse Events and Serious Adverse Reactions (nested drug trial participants only) ... 58
6.9.4. Serious Adverse Events which do not require reporting to Sponsor ... 59
6.9.5. SUSAR reporting ... 59
6.10. Statistical software ... 59
7. REFERENCES ... 60

GLOSSARY OF ABBREVIATIONS	
AE	*Adverse Event*
AR	*Adverse Reaction*
BD	*Twice Daily*
CA	*Competent Authority*
CI	*Chief Investigator*
CONSORT	*Consolidated Standards of Reporting Trials*
CRF	*Case Report Form*
CTA	*Clinical Trial Authorisation*
CTIMP	*Clinical Trial of Investigational Medicinal Product*
CTU	*Clinical Trials Unit*
DHSC	*Department of Health & Social Care*
DI	*Designated Individual*
DMEC	*Data Monitoring and Ethics Committee*
DSUR	*Development Safety Update Report*
EC	*European Commission*
EMA	*European Medicines Agency*
EQ-5D-5L	*Euroqol 5 level instrument (5 questions on mobility, self-care, usual activities, pain or discomfort and anxiety or depression)*
EU	*European Union*
EUCTD	*European Clinical Trials Directive*
EudraCT	*European Clinical Trials Database*
EudraVigilance	*European database for Pharmacovigilance*
GAD-7	*Generalised Anxiety Disorder-7 questionnaire*
GAfREC	*Governance Arrangements for NHS (National Health Service) Research Ethics*
GCP	*Good Clinical Practice*
GP	*General Practitioner*
HRA	*Health Research Authority*
IC	*Integrated Care*
ICP	*Integrated Care Pathway*
ICF	*Informed Consent Form*
ICH	*International Council on Harmonisation of technical requirements for registration of pharmaceuticals for human use*
IDMC	*Independent Data Monitoring Committee*
IMP	*Investigational Medicinal Product*
IMPD	*Investigational Medicinal Product Dossier*
IPR	*Individual Patient Randomisation*
IQR	*Inter-Quartile Range*
ISF	*Investigator Site File*
ISRCTN	*International Standard Randomised Controlled Trials*
ITT	*Intention-to-Treat*
MHRA	*Medicines and Healthcare products Regulatory Agency*
MWSAS	*Modified Work and Social Adjustment Scale*
NHS	*National Health Service*
NHS R&D	*National Health Service Research & Development*
NIHR	*National Institute for Health Research*
OD	*Once Daily*
OG	*Operational Group*
PCN	*Primary Care Network*

PCS	*Post COVID Syndrome*
PDQ	*Perceived Deficit Questionnaire*
PHQ-9	*The Primary Care Evaluation of Mental Disorders Patient Health Questionnaire*
PI	*Principal Investigator*
PIS	*Participant Information Sheet*
QA	*Quality Assurance*
QC	*Quality Control*
QP	*Qualified Person*
RCT	*Randomised Control Trial*
REC	*Research Ethics Committee*
RSI	*Reference Safety Information*
SAE	*Serious Adverse Event*
SAP	*Statistical Analysis Plan*
SAR	*Serious Adverse Reaction*
SD	*Standard Deviation*
SDV	*Source Data Verification*
SE	*Standard Error*
SF12	*Short Form Questionnaire 12 questions*
SOP	*Standard Operating Procedure*
SmPC	*Summary of Product Characteristics*
SUSAR	*Suspected Unexpected Serious Adverse Reaction*
TMF	*Trial Master File*
TMG	*Trial Management Group*
TSC	*Trial Steering Committee*
UC	*Usual Care or Standard Care*
UCLan	*University of Central Lancashire*
UCLH	*University College London NHS Foundation Trust*
UK	*United Kingdom*
USM	*Urgent Safety Measure*
WOCBP	*Women of Childbearing Potential*
WP	*Work Package*
WSAS	*Work and Social Adjustment Scale*

1. ADMINISTRATIVE INFORMATION

1.1. Title

Statistical Analysis Plan (SAP) for STIMULATE-ICP: A pragmatic, multi-centre, cluster randomised trial of an integrated care pathway with a nested, Phase III, open label, adaptive platform randomised drug trial in individuals with Long COVID.

1.2. Trial Registration: IRAS 1004698

IRAS Number:	1004698
EudraCT Number:	2021-006598-47
SPONSOR:	University College London (UCL)
SPONSOR Number:	143602
FUNDERS:	National Institute for Health Research<br>Perspectum
FUNDERS Number:	29622 NIHR COV-LT2-0043

1.3. Protocol:

This document has been written based on information contained in the Study Protocol Version 5.1 Date: 18/08/2025.

1.4. SAP version: 7.1

Date: 22/03/2026

Docusign Envelope ID: 694B9EEA-7D5D-4DDE-8CD2-98FA170FF184

1.5. SAP revision history

Protocol version	SAP version	Section number changed	Description and reason for change	Date changed	Condition of change: before/ after review of blinded/ unblinded outcome data	Initials
**2.0**	**1.0**	 **3.5**<br>**3.8**<br>**3.10**<br>**4.2 & 6.3**<br>**6.3**<br>**6.7**	**Reason for change is feedback from IDMC meeting 31/03/2022 and TSC meetings 30/03/2022**<br>**Change of date of last recruitment due to delayed start.**<br>**Stopping rules should be based on safety rather than futility – IDMC.**<br>**Wording on timing of establishing eligibility for the drug trial.**<br>**The phrase "Intention-to-Treat" (ITT) has been removed from the document on the advice of the IDMC as we do not intend to impute missing primary outcomes for the principal analyses.**<br>**Additional text and figure 2 have been added to make the principal analysis sets clearer for the 2x2 factorial trial and the drug trial.**<br>**The choice of p-value for all interim analyses has been reduced to 0.001 from 0.005**	**02/05/2022**	**Before**	**GJP**

			**and justified using Haybittle-Peto.**			
**2.0**	**1.1**	**1.6**<br><br>**6.7**	**Correction of professional affiliation for Prof Tim Peters (IDMC).**<br><br>**Change from p≤0.001 to p<0.001.**	**24/05/2022**	**Before**	**GJP**
**2.0**	**1.2**		**Minor changes and signatures erroneously added to version with tracked changes**	**24/05/2022**	**Before**	**GJP**
	**2.0**		**Removal of comments to have clean copy and addition of new signatures**	**06/11/2022**	**Before**	**GJP**
**3.0**	**3.0**	**3.1 & 3.3**<br><br>**3.10**<br><br>**4.3**<br>**5.2**<br><br>**6.8**	**Update to match protocol v3.0**<br><br>**Addition of page numbers**<br><br>**Addition of drug trial only centres and relevant changes to the randomisation and analysis sections**<br><br>**Date of randomisation clarified as date used for timing of follow-up points for all patients in the drug study. Date on enrolment for all other participants**<br><br>**Analysis populations clarified by number**<br><br>**Additional exclusion criteria for gifting blood and also for the drug trial**<br><br>**If missing data exceeds 10%, a multiple imputation analysis will be completed as well as a complete case analysis and threshold approach**	**02/02/2023**	**Before**	**GJP**

**4.1**	**4.0**		**Update to match protocol v4.1**	**11/04/2024**	**Before**	**GJP**
		**5.2**	**Inclusion of patients (with ongoing long COVID symptoms) seen prior to the study commencement into the drug study.**			
		**5.2**	**SmPC update issued for Famotidine to exclude Posaconazole, Dasatinib, Pazopanib, Erlotinib and Gefitinib for concomitant use. (N.B. Erlotinib and Gefitinib were not previous exclusions for any of the selected IMP`s.)**			
		**5.2**	**Correction eGFR to (eGFR <30 mL/min) in eligibility criteria**			
		**4.3**	**Revision of statistical sections so that the principal analysis population for the cluster trial is changed to "those who consented to data collection for the cluster RCT regardless of their participation in the drug trial or their drug trial treatment allocation"**			
		**4.3**	**Revision of statistical sections so that principal analysis population for drug vs. usual care comparisons within the drugs trial will to "anyone randomised to the drug trial including participants also cluster randomised to any treatment pathway in the cluster trial, participants from cluster trial sites who have previously been seen at a clinic, and participants randomised into the drug trial from clinics that can only offer the control treatment pathway including both new**			

		6.3<br><br>6.3<br><br>6.7<br><br>6.8<br><br>7.0	clinic patients and those who have previously been seen at a clinic".<br><br>General increase of clarity in response to IDMC comments. Sections 6.3.1-6.3.5 added.<br><br>Further analysis for the final secondary objective is outlined in sections 6.3.6 and 6.3.7 and described in detail in appendices 2 and 3.<br><br>The p-value for each of 5 interim analyses was due to be reset from p=0.005 to the more conservative p=0.001 on advice of the IDMC. The interim analysis plan has been rewritten and as the number of interim analyses is reduced from 5 to 1 the p-value for the interim analyses, is instead changed to p=0.005.<br><br>The missing data section has been rewritten including a strategy for imputing a FAS score when there is a single missing item.<br><br>Addition of references section.			
**5.0**	**5.0**	**All**<br><br>**Key contacts**<br><br>**2.3.2, 6.3.6, 6.3.7**	**Update to match protocol v5.0 and response to IDMC.**<br><br>**Independent TSC member Ondine Sherwood, PPI Representative, was erroneously omitted from earlier drafts.**<br><br>**Updates to section 2.3.2 to change characterisation of the pathophysiology and trajectory of Long COVID from**	**06/04/2025**	**Before**	**GJP**

		3.1	**a secondary trial objective to an exploratory objective. Addition of new sections describing the exploratory analysis for the study. This is covered in more detail in appendices 2 and 3.**				
		3.4, 3.8, 4.1, 4.3, 6.8	**Widening of week 24 data collection window to -7 days/+ 6 weeks to allow a greater amount of time for completion of questionnaires by participants.**				
		4.2	**Removal of interim analysis following oversight committee consultation. An interim analysis was planned after the recruitment of 600 participants into the drug trial. This occurred much closer to the end of the recruitment period than anticipated. It was therefore agreed that the interim analysis served no purpose.**				
		6.0<br>6.1	**Text on collection of information about enrolment on Living with COVID Recovery™ app and frequency of use of the was clarified.**				
			**Additional CONSORT references added.**				
		6.3	**Greater detail has been added on the treatment of unexpected patient-reported data in numeric fields. This includes the treatment of ranges and less than or greater than a specific number rather than a number. Greater detail has been added on the treatment of unexpected site-reported**				

		6.3<br>6.3<br>6.3<br>6.3<br>6.5<br>6.6	**data in numeric fields using less than or greater than a specific number.**<br>**Greater detail on intended analyses of the cluster and drug trial data has been added throughout.**<br>**Greater clarity has been given on the meaning of complete or available case analyses**<br>**In models where PCN is included as a random effect, the binary stratification factors used in the randomisation of the PCNs have been added as fixed effects. The random effects will be only random intercepts.**<br>**Participants who join the drug trial directly will not have been cluster randomised by PCN. Therefore, the adjustment for all participants in the drug trial needs to reflect the individual randomisation by sex, site and relevant pathway (cluster treatment pathway and drug only new or previously seen participant).**<br>**To account for systematic effects of participating in both the cluster and drug trial, an indicator term will be added to each model in both the cluster and drug trials.**<br>**Greater detail on the sensitivity analyses has been added. This covers an analysis of those participants receiving the allocated ICP**			

		6.8	**interventions and a further analysis including greater information from the Coverscan or greater engagement with the Living with COVID Recovery™ app.**<br><br>**A table has been added to define three pre-specified subgroups on the presence of symptoms in the baseline participant questionnaire.**<br><br>**The previous statement on missingness of 10% in "all data" requiring multiple imputation was replaced. Greater clarity was added, specifically that missing 12-week FAS of more than 10%, would necessitate a multiple imputation analysis as well as threshold approach. More detail has been given on the form of the multiple imputation.**			
**5.0**	**6.0**		**Removed final comments from IDMC member and responses.**<br><br>**Prepared clean copy for signatures of TCS chair, CI and Trial Statistician.**	**03/05/2025**	**Before**	**GJP**
**5.0**	**7.0**	**Appendices 2 and 3**	**No changes to the main CTU SAP or appendix 1.**<br><br>**Changes made by statisticians at the Crick and Perspectum, at the request of the Sponsor, to appendices 2 and 3, the SAPs for analyses to be carried out by the Crick and Perspectum.**	**07/07/2025**	**After**	**GJP**
**5.1**	**7.1**	**6.4**	**Removed details pertaining to use of NHS data regarding medical history from January**	**22/03/2026**	**After**	**GJP**

			**2020 and up to 1 year following enrolment in the study via NHS digital. This data will no longer be collected as part of the trial.**			

The SAP will be reviewed again by the TSC just prior to the start of the statistical analysis.

1.6. Names, affiliations and roles of SAP contributors

Authors
Dr Gordon Prescott, Director Statistics, Lancashire CTU, University of Central Lancashire (UCLan) (Statistician, unblinded)
Dr Hakim-Moulay Dehbi, Head of Statistics, Comprehensive Clinical Trials Unit, UCL (Statistician unblinded)

Reviewers
Prof Paddy Mallon (Independent, TSC Chair), Professor of Microbial Diseases, School of Medicine University College Dublin and Consultant in Infectious Diseases in St Vincent's University Hospital, Dublin
Dr Marion Mafham (Independent, TSC), Associate Professor and Senior Clinical Fellow, Clinical Trial Service Unit and Epidemiological Studies Unit, Nuffield Department of Population Health, University of Oxford
Prof Matt Sydes, (Statistician, TSC), Professor of Clinical Trials & Methodology, MRC Clinical Trials Unit, University College London
Ondine Sherwood (Independent, TSC), Founder of Long COVID SoS and Public and Patient Involvement (PPI) Representative
Prof Peter Langhorne (Independent, IDMC Chair), Professor of Stroke Care, University of Glasgow
Prof Tim Peters (Independent Statistician, IDMC), Professor of Primary Care Health Services Research, Bristol Medical School and Bristol Dental School, University of Bristol
Dr Chris Sutton (Independent Statistician, IDMC), Associate Professor and Deputy Director (Methodology) and Lead Statistician for Manchester CTU, University of Manchester
Dr Ly-Mee Yu (Independent Statistician, IDMC), Associate Professor and the Deputy Director Academic and Lead Trial Statistician, Primary Care Clinical Trials Unit at the Nuffield Department of Primary Care, University of Oxford

1.7. Signatures

Chief Investigator: Professor Amitava Banerjee

Signature:

Date: 23-03-2026

Trial Statistician (unblinded): Dr Gordon Prescott

Signature:

Date: 23-03-2026

TSC Chair: Professor Patrick Mallon

Signature:

Date: 24-03-2026

2. INTRODUCTION

2.1. Background

Long COVID (1) spans multiple healthcare challenges, particularly how to deliver sustainable, high-quality care for multimorbidity and long-term conditions (LTC). There are inequalities in health, access to healthcare; incomplete pathways across community and hospital care; inadequate research translation to practice; and insufficient resources. Integrated care pathways (ICP) are structured multidisciplinary care plans of essential steps in care of specific conditions (2). An effective and deliverable ICP for Long COVID could reduce direct and indirect pandemic effects and improve LTC care beyond COVID-19 and potentially be cost effective. Long COVID services were initiated whilst research is still defining the disease. Guidelines are developing in absence of evidence. An ICP approach offers coordination across specialties, across investigation, treatment, and rehabilitation, as well as opportunities for real-time iterative improvements in service design and delivery.

ICPs are of increasing relevance in many LTCs. ICPs can improve quality and access to care, patient experience and satisfaction, while reducing complications and non-elective admission rates (3-5). Research and trials of ICPs should incorporate design elements “likely to maximise effectiveness” (5) and engagement of patients, clinicians and health systems to improve healthcare performance (6).

Long COVID is a syndrome, not a single condition. The “syndemic” of COVID-19 (convergence of an infectious disease, under-treated non-communicable diseases, and geographical, and economic determinants of health (7) necessitates ICP strategies, which could inform other LTCs. Predictors of poor acute COVID-19 outcomes are established (8), but effective treatments for Long COVID are not yet defined (9, 10).

The NIHR funded STIMULATE-ICP project consists of three interconnected, cross-linked **Work Packages** which are led and managed by a multidisciplinary team across different organisations from academia, health services and industry.

The work packages are designed to be time-sensitive, iterative, adaptive, and effective, utilising learning health systems (health science, clinical evidence, and care) (11), implementing science, (11-13) and complex interventions (14).

The trial protocol details work package 2 of the STIMULATE-ICP NIHR funded project which is a pragmatic, multi-centre, cluster-randomised, Phase III trial of an integrated care pathway with a nested, adaptive, open label, randomised drug trial in individuals with Long COVID.

2.2. Rationale for the trial

There is variation in access to referral and care, and poor patient experience for Long COVID (1). With stretched resources during the pandemic, the need for rationalisation of investigation and rehabilitation across diseases is paramount. In Long COVID, where up to 70% of patients show evidence of mild impairment in one or more organs, (15) an early supported investigation strategy may direct management, as well as better defining the syndrome, with relevance to other LTCs. Despite the primary care setting, there is no evidence-based treatment or rehabilitation. There have

been few clinical trials to date, particularly in non-hospitalised individuals, or in the context of overall care pathways. As Long COVID clinics are implemented, trials of ICPs can inform diagnosis, care, public health, policy planning, resource allocation and budgeting.

In the UCLH Long COVID clinic (the first and largest in the UK), community-referred patients were more symptomatic, more likely to have psychological impact, and less likely to be fit for work than patient's post-hospitalisation. Any ethnic and socioeconomic disparities in referrals, assessment, and rehabilitation (16) need to be understood at a system level and compared with other LTCs, to provide lessons beyond COVID-19, which has worsened pre-existing inequities (7). Pragmatic implementation of effective and equitable interventions is urgently needed to reduce inequalities (17).

Long COVID is estimated to have a substantial population burden, with 2 million people affected in the UK. Even in non-hospitalised individuals, up to 14% of people with COVID-19 may have persistent symptoms (3), suggesting future major healthcare and economic burden (4, 18). We are challenged "to design research that integrates reported lived experience needs with clinical models of care" (6). Guidelines recommend referral to Long COVID clinics if symptoms persist for 4-12 weeks (10). However, neither the syndrome nor effective care is defined (19). Despite roll-out of 83 centrally funded Long COVID clinics, ICPs are not coordinated and the most effective ICP remains unknown because elements of the pathway and their delivery are lacking in evidence. Patients and public want "recognition, research and rehabilitation" simultaneously (14), as well as timely tests and treatment, which may be more deliverable via an ICP.

Real world data needs to be analysed to understand the trajectory of Long COVID, the nature of "usual care" and the effectiveness of current care pathways. These data have already informed our choice of interventions for diagnosis and rehabilitation for a cluster-randomised trial.

Coverscan™ is a multi-organ MRI assessment to assess the impact of COVID and Long COVID on affected organ function. This is either by scanning the whole organ or (in the case of the lungs) by cross sectional scanning for representational function assessment. Coverscan™ is a UKCA marked medical device which also has exceptional use authorisation by the MHRA and needs to be evaluated at scale, where there is currently significant variation in investigations pre-Long COVID clinic. Self-management has been the mainstay of rehabilitation in usual care, but more comprehensive, digitally enabled programmes need to be quickly evaluated, such as Living with COVID Recovery™. Rather than studying specific components in isolation, given the scale of Long COVID and the urgency of the public health burden, integrated pathways need to be evaluated.

In addition to the components of the ICP, we need a robust evidence-base to inform drug therapies for effective treatment of Long COVID. Some medications are being widely used among patients and health professionals without evidence base.

As a pragmatic trial design based in routine care, it is important to analyse data pre-trial (WP1), data from the trial and demographic and socio-economic data to monitor inequalities (WP3) in the intervention, as well as considering applicability to other LTCs.

Patient surveys, interviews with patients, health professionals and policymakers, and data from UCLH clinic suggest a lack of coordination of care and missed opportunities to improve investigation and rehabilitation. An example of this is repetitive investigations in different modalities for the same organ, and different scans at different times for different organs. One of the Integrated Care components that we will assess is Coverscan™ (15), which is a non-CE marked medical device given MHRA-authorisation for exceptional use in research and clinical care, and has been used in 500 patients. It aligns with national policy for community diagnostics (NHS Diagnostic Hubs (34)). Interviews with clinicians, patients and researchers show support for Coverscan™ to be used as an early investigation strategy, to be evaluated in routine care, and to help stratify treatment.

The second component of Integrated Care to be assessed is "Living with COVID Recovery™" which is an NIHR-funded digital health intervention (NIHR132243), initiated in August 2020 (36), providing videos, information and rehabilitation treatments under guidance of a rehabilitation clinician. The "Living with COVID Recovery™ App is a class I, CE Marked Medical Device. Over 400 patients in 15 of the 83 Long COVID clinics (mean age: 54 years, range 23–78; 50% from ethnic minorities), have used the app to-date. Patients perform seven actions per week such as weight recording, patient reported outcome measures (PROMs), completion of GAD-2, MRC Dyspnoea score or exercise tracking, with half creating and tracking chosen goals. A physiotherapist needs an hour to review each patient, managing 70 individuals per month. Clinicians find it efficient and patient-centred.

To date, little to no treatment data exists for Long COVID; understanding of the pathophysiological mechanisms of disease is poor, and few well-designed clinical trials are currently registered. UCLH hosts the first, and largest of the NHS Long COVID clinics. In a cohort of >1000 patients living with Long COVID at UCLH, the most common reported symptoms are fatigue, shortness of breath and reduced exercise tolerance, similar to reports from other centres (16, 20). Median Fatigue Assessment Scores (FAS) at presentation are >30 in >60% of Long COVID patients (normal range FAS <20) irrespective of time since symptom onset. FAS scores are significantly higher than post-hospitalised patients, similar to other reports (21).

Proposed mechanisms underlying Long COVID symptom clusters include mast cell activation, microcirculatory deficiency caused by microthrombi, and mitochondrial insufficiency. Preliminary observations by our UCLH team suggest Long COVID patients have evidence of persistent inflammation and/or microcirculatory insufficiency, resulting in poor aerobic muscle capacity and profound fatigue. The nested STIMULATE-ICP trial interventions support existing hypotheses and are based on data from UCLH Long COVID clinic patients.

We hypothesise that drug treatments in Long COVID may significantly improve symptoms and physical function, through different mechanisms of action. We have designed an adaptive, platform clinical trial (22-25) within STIMULATE-ICP, to robustly test efficacy of drugs in Long COVID. We will start with Loratadine plus Famotidine, Colchicine and Rivaroxaban and continue to add / remove drugs according to efficacy on fatigue in Long COVID and as approved by the Independent Data Monitoring Committee (IDMC), Trial Steering Committee (TSC) and the Funder (NIHR). It is likely that Long COVID represents a heterogenous group of different pathological responses to primary mild to moderate COVID-19 infection, with unknown predisposition. However, since the underlying cause(s) of Long COVID are unknown, we have chosen to test these drugs across all participants with Long

COVID, irrespective of their individual symptoms or investigation findings, as we cannot currently determine which approach is optimal to treat the hypothesised underlying inflammatory process. This approach will minimise selection bias and allow multiple secondary analyses to test for efficacy in particular sub-groups of patients, based on symptom clusters or imaging findings.

The large sample size will enable us to undertake multiple pre-specified "symptom phenotypes" analyses based on both Coverscan™ findings and symptom clusters to determine if these sub-groups do benefit from specific drugs or not.

Estimating the individual effect size of these drugs on fatigue is complex, as extrapolating preliminary data to the larger patient cohort is difficult. We hypothesise that Long COVID patients who experience symptoms may gain most benefit from the trial drugs for example, in the absence of detailed pathophysiological data for Long COVID, we are open to the possibility of differential effects of these drugs in different sub-groups.

Alternative treatment arms may be included/changed during the trial at pre-specified points on the recommendation of the IDMC and TSC, based on advice and involvement from the COVID-19 UK Government Therapeutics Task Force at the Department of Health & Social Care.

2.3 Trial Summary

2.3.1. Aim

The trial aims to evaluate "Integrated Care" versus "Usual Care" in a cluster randomised trial

"Integrated Care" involves:

a) Multi-organ MRI (Coverscan™) and clinical decision support; and/or

b) Enabling a community-based rehabilitation using a digital Application Platform (Living with COVID Recovery™).

"Usual Care" involves:

a) Usual Investigations; and

b) Self- management website with associated online resources (*Your COVID Recovery™)* for rehabilitation (26).

2.3.2. Objectives

PRIMARY OBJECTIVE:

The overall primary objective is to evaluate the effect of "integrated care" with combinations of multi-organ MRI (Coverscan™) and clinical decision support, and digitally enabled community rehabilitation (Living with COVID Recovery™) versus "usual care" [Usual Investigations; and Your COVID Recovery™ self-management website] on Fatigue Assessment Score (FAS) at 12 Weeks from the enrolment date.

SECONDARY OBJECTIVES:

The secondary objectives include:

1. Clinical efficacy of the individual components of the integrated care pathway, including potential therapies.

2. Effect of the ICP on mean measured health related quality of life, mental health (including depression), work and social adjustment, physical function, organ impairment and healthcare utilisation through patient reported outcomes.

EXPLORATORY OBJECTIVES:
To characterise the pathophysiology and trajectory of long COVID.

3. METHODS

3.1. Design

The STIMULATE-ICP trial is a pragmatic, multi-centre, two by two factorial cluster-randomised trial of an integrated care pathway (ICP) which is adaptive to local resources and needs, reactive to changing pandemic and policy environments and iterative, developed upon ongoing results; with a nested, Phase III, open label, adaptive platform randomised drug trial in individuals with Long COVID to evaluate 12 Weeks treatment of four potential therapies. Using a platform approach, new drugs may be added to the protocol, pending approval from NIHR and Department of Health and Social Care (DHSC)).

The STIMULATE-ICP trial is testing the hypothesis that an Integrated Care Pathway for Long COVID is more effective than Usual Care at improving the most common symptom of fatigue.
Currently, patients with signs and symptoms of Long COVID undergo usual investigations by their primary care physicians before they are referred to Long COVID clinics where they undergo further investigations and clinical decision support.

This trial aims to investigate two components of care to Evaluate “Integrated Care” versus “Usual Care” as shown in Table 1.

“Integrated Care” involves:
i. Multi-organ MRI (Coverscan™) and clinical decision support; and/or
ii. Enabling a community-based rehabilitation using a digital Application Platform (Living with COVID Recovery™).
“Usual Care” involves:
iii. Usual Investigations (includes blood tests, ECG, Chest X ray and Exercise Tolerance Test); and
iv. Self- management website with associated online resources (Your COVID Recovery™) for rehabilitation https:\\www.yourcovidrecovery.nhs.uk (26).

Table 1. 2x2 factorial trial showing integrated care and usual care in relation to the two uplift of care interventions

		Digital application platform intervention	
		Uplift in care	Usual care
Multi-organ MRI Coverscan intervention	Uplift in care	a. Coverscan™ + Living with COVID Recovery™ App.	b. Coverscan™ + Usual Care Self-Management Rehabilitation.
	Usual care	c. No Coverscan™ + Living with COVID Recovery™ App.	d. No Coverscan™+ Usual Care Self- Management Rehabilitation.

Eligible patients will be approached to consent to a nested, adaptive platform randomised drug trial. All patients will be sent an invitation letter to the trial with the trial Participant Information Sheet and Consent Form prior to their Long COVID Clinic appointment. The patients may contact the trial team prior to their Long COVID clinic appointment or at the clinic appointment or up to 7-10 days* after their clinic appointment to participate. Consent will be taken at any of these time points but randomisation to the nested, adaptive platform randomised drug trial will only occur at the clinic appointment or in the 7-10 days after the clinic appointment. No randomisation will occur until eligibility for the nested drug trial has been confirmed by the clinical assessment and patient history by the clinic team. Patients who are ineligible or opt not to participate in the drug trial will be asked to consider consent for data collection or data collection plus blood samples and donation to biobank.

* We will not enrol participants for the drug arm after this time frame, in order to align the taking of IMP as far as possible with the 12-week clinic pathway and the 12-week follow-up. 12-week follow-up is based on date of first clinic appointment and commencement of clinical pathway

Eligible patients who consent to the drug study will be randomised to one of the following 4 arms:

1. Famotidine + Loratadine
2. Colchicine
3. Rivaroxaban
4. No Drug

All patients will be assessed at 12 Weeks and 24 Weeks for Fatigue Assessment Scale.

It was originally intended that other drugs might be added as additional or replacement arms for new patients at predetermined time points during the trial (6 months and 12 months), dependent on interim analysis of trial data with consideration given to updated recommendations from the NIHR advisory board. The changes in Protocol v4.0 mean that further drugs will not be added before the end of the trial. All plans for interim analyses were dropped from Protocol v5.0.

A substantial amendment to the protocol was made to include additional clinics that contribute participants only to the drug trial. In clinic areas that are not able to provide both Coverscan™ and the Living with COVID Recovery™ app, it will not be possible to randomise PCNs into integrated care pathway clusters. These sites will participate in the drug study only, widening access for patients and

increasing participation in the platform drug study. All participants in these sites will receive all Usual Care in addition to the opportunity to participate in the individually randomised drug trial. In these sites we will only collect data from those enrolled in the drug study.

A further substantial amendment allows for patients who have previously been referred to a participating Long COVID Clinic and who are eligible will be offered the opportunity to participate in the individually randomised drug trial. They will also be asked if they are willing to gift blood. Data from these participants will not be used in the analysis of the integrated care pathway trial, only the analysis of the drug trial. These patients will be sent an invitation letter to the trial with the trial Participant Information Sheet. They will be asked to contact the Research Team to arrange a study visit if they wish to participate. Randomisation will be individual patient randomisation.

3.2. Interventions

For the four-arm, 2x2 factorial, cluster randomised controlled pragmatic trial for usual care service uplift:

Test Interventions:

a. Coverscan™ + Living with COVID Recovery™ App.
b. Coverscan™ + Usual Care Self-Management Rehabilitation.
c. No Coverscan™ + Living with COVID Recovery™ App.

Control intervention:

d. No Coverscan™+ Usual Care Self- Management Rehabilitation.

For the nested, adaptive platform randomised drug trial

Test Interventions: Usual care PLUS
Loratadine: 10 mg tablet orally once a day swallowed whole with water every morning PLUS
Famotidine: 40mg tablet orally once a day swallowed with water every morning.
OR
Usual care PLUS Colchicine: 500mcg tablet taken orally twice a day swallowed with water every morning.
OR
Usual care PLUS Rivaroxaban: film coated 10mg tablet taken orally once a day swallowed with water every morning.

Control intervention:
Usual care

3.3. Randomisation

Cluster randomisation for usual care service uplift (2x2 factorial trial)

If using Individual Patient Randomisation (IPR), the provision of information for the scan, time to consider, and consent would need to take place at the General Practitioner (GP) level. Given the current strain on services this is not feasible and could potentially increase waiting times for other

practice patients, affect number of patients approached and recruitment would be unlikely to achieve sample size required within the trial timeline. Similarly, if IPR is used for Living with COVID Recovery™ at the GP level the same barriers to recruitment occur.

IPR would mean patients have a 50/50 chance of receiving a scan and a 50/50 chance of being allocated to Living with COVID Recovery™. Cluster randomising at the PCN level will have the same allocation of access across the total number of patients referred to the participating clinic so that equipoise exists at the clinic level. Clustering will use demographic and socio-economic data available for each PCN to ensure equity of access across deprivation indexes within GP codes. Clustering at the PCN level allows Coverscan™ and/or Living with COVID Recovery™ to be provided as an uplift to previous care in allocated PCNs. It will become integrated care within that PCN and, as such, individual patient consent will not be sought (access to and collection of Coverscan™ and Living with COVID Recovery™ app data for research purposes will be by individual patient consent on entering into the study).

Up to 226 Primary Care Network (PCN) sites will be allocated using blocked stratified randomisation using block size of 4 into one of the four 2x2 factorial allocations labelled as a, b, c, d (see below) in ratio 1:1:1:1:

a. Coverscan™ + Living with COVID Recovery™ App.
b. Coverscan™ + Usual Care Self-Management Rehabilitation.
c. No Coverscan™ + Living with COVID Recovery™ App.
d. No Coverscan™ + Usual Care Self- Management Rehabilitation.

Randomisation lists for the PCN areas will be created by the study statisticians based at UCL and the Lancashire CTU. First a matching exercise will be undertaken of publicly available data linking GP practices and PCNs, numbers of patients registered with GPs (2022) and ranks of deprivation (2019) associated with postcodes of GP practices. Then data will be aggregated by PCN, for each PCN taking the sum of the numbers of registered patients at the relevant GPs and the mean of the ranks of deprivation for each GP postcode weighted by numbers of registered patients at that GP.

To form the strata, PCNs linked to each clinic site will be ordered first by descending size of registered patient population and then defined by a single cut off value close to the median size of all UK PCNs as large PCNs or small PCNs. Then large PCNs will be sorted by descending deprivation and small PCNs by ascending deprivation and defined by a single cut off value close to the median deprivation of all UK PCNs as more or less deprived. Within the four strata of PCNs: large, less deprived; large, more deprived; small, more deprived; and small, less deprived, treatment pathways will be allocated using randomised blocks of fixed size 4. This will reduce bias and ensure that there is balance across the pathways by deprivation and population size within each clinic site.)

Allocation of the PCNs using randomised blocks of size 4 will be undertaken in STATA version 17 using command ralloc to create random allocations for 10 clinic sites and 4 strata within each clinic and 16 PCNs within each strata. The PCNs will be matched to the random allocation using an index based on strata clinic site (1-10), size of PCN population (large, small), and deprivation (less deprived, more deprived) and within the strata by decreasing deprivation (large PCNs) and increasing deprivation (small PCNs). If additional PCNs are added to existing clinic sites after the

Docusign Envelope ID: 694B9EEA-7D5D-4DDE-8CD2-98FA170FF184

first randomisation these will be sorted in the same order, but assigned to the next available allocation. This process for adding PCNs to existing clinic sites after the first randomisation will be repeated whenever additional PCNs are introduced, either individually or several at a time.

All satellite GP practices will treated as being part of the same PCN as the main GP practice and assigned the same allocation as the main practice. GP practices that move location during trial recruitment will retain the same allocation as at the original location. If GP practices are reallocated to different PCNs during trial recruitment, the GP practice will retain the treatment pathway allocation of the original PCN.

Drug trial randomisation

Individual participant randomisation will be undertaken using an online system provided by the company Sealed Envelope™. Coordinated registration and allocation of participant trial numbers will be required to enrol participants. Anyone agreeing to data collection will be registered in the trial database and assigned a participant trial number. Only those participants who have consented to data collection, and additionally to the drug trial, and have had eligibility confirmed by a clinician will be randomised into the drug trial. Participants will be assigned to treatment groups through consecutive allocation, and the use of a Trial Subject Enrolment Log which will be stored on the Sealed Envelope™ system.

The randomisation will be implemented through the online randomisation portal, supplied by Sealed Envelope™ Ltd. Blocked randomisation of size 4 and size 8 (ratio of number of blocks used 1:1) will be used to allocate study participants to one of the 4 drug or control treatment groups, stratified by clinic, birth gender and treatment pathway.

If additional drug treatments are added later in the drug trial later, blocked randomisation of size k and 2k (ratio of number of blocks used 1:1) will be used to allocate trial participants in an equal allocation ratio to one of the k treatment groups under consideration at that point.

Participants recruited through a clinic site taking part in the drug trial only will not undergo the cluster randomisation element or activities. Their pathway will start at the Long COVID clinic appointment, and they will be randomised to one of the four drug arms. The randomisation will be implemented through the online randomisation portal, supplied by Sealed Envelope™ Ltd. Blocked randomisation of size 4 and size 8 (ratio of number of blocks used 1:1) will be used to allocate study participants to one of the 4 drug or control treatment groups, stratified by clinic and gender. Treatment pathway will not be a stratification factor as all participants from these clinics will receive the same usual care treatment pathway. As above, if additional drug treatments are added later in the drug trial later, blocked randomisation of size k and 2k (ratio of number of blocks used 1:1) will be used to allocate trial participants in an equal allocation ratio to one of the k treatment groups under consideration at that point.

Patients who have previously been referred to a participating Long COVID Clinic (prior to the current trial) and who are eligible will be offered the opportunity to participate in the individually randomised drug trial. Participants who have previously been referred will not undergo the cluster

randomisation element or activities. Their pathway will start at the Long COVID clinic appointment, and they will be randomised to one of the four drug arms.

3.4. Blinding

There will be no blinding for the drug arm of this study: this an open label study. As this a pragmatic trial with currently unconfirmed drugs being introduced later, for practical reasons it has not been feasible to pursue encapsulation or placebos for multiple drugs. The majority of the key outcomes are patient-reported outcomes and so there is little potential for outcome assessors to introduce bias by knowing cluster or drug allocation. The large number of interim analyses originally expected in this trial made blinding of the statisticians impractical.

3.5. Sample size

Motivation: The main study takes the form of a 2x2 factorial trial where the unit of randomisation is the PCN. The sample size calculation was based on the power to detect an interaction effect of clinically important size of 3 points (27) on the FAS scale (28) rather than on the size of the main effects themselves. Therefore, the sizes of the main effects are not specified below. For context, the difference in means of FAS between people who say they have or have not recovered from Long COVID was 9 points in an observational study (28).

The sample size was calculated using PASS v21.0.1 (2021) for a 2x2 cluster factorial trial. The study was powered to detect an interaction on the FAS scale between Coverscan™ and Living with COVID Recovery™. The sample size does not depend on the size of main effects of both individual interventions. Based on published data the standard deviation on the FAS scale for patients with Long COVID is estimated at 6 units (see Figure 12 of reference (33) from the Protocol). An interaction effect of 3 points (27) on the FAS can be detected with just over 90% power and (two-sided) significance level of 0.05 with 960 participants in 48 PCN clusters of 20 participants, assuming a conservative intra-cluster correlation coefficient (ICC) of 0.02. If there is dropout of 15% (to be conservative) and assuming missingness is roughly equal across the arms, then the number of participants must be inflated by a factor of 100/85 to 1130 (approximately 56.5 centres of 20 participants).

Only if there is no possibility of an interaction effect between the drugs of the platform trial and the main and interaction effects in the 2x2 factorial trial, can the trials be considered independent and data from all arms of the adaptive trial can be combined to assess the effects within the 2x2 factorial trial. Assuming non-independence of the two trials, the total number of participants required for the interaction effect within the factorial trial to be detected with 90% power is 1130 receiving usual care (i.e. those receiving usual care (no drug) through not entering the drug trial, or those randomised to usual care (no drug) in the drug trial).

Experimental estimates of the prevalence of symptoms that remain 12-weeks after COVID infection range from 3.0% based on tracking specific symptoms, to 11.7% based on self-classification of Long COVID, using data to 1 August 2021 (3). One Long COVID clinic has ~25 new patients per week(16),

suggesting there might be around 1300 new patients annually per clinic. Most are expected to give consent to data collection even if not willing to consent to randomisation into a drug study. Long COVID referral rates range from 0-2.5/1000 per PCN. Assuming this rate will not change over the foreseeable future, this will ensure that our 6-10 Long COVID clinics will provide sufficient patients for this study. Moreover, there is a backlog of people waiting for their first Long COVID clinic appointment (from private communication).

The total number of individual drugs tested on the platform is currently unknown. The total sample size required in an adaptive platform drug trial, with uncertainty in the timing of the introduction of further drugs, cannot be set in advance, but would need to be updated in consultation with the IDMC and funder throughout the trial at set interim points in data collection.

The treatment effect for the drugs is unknown at this time. The original plan was to perform interim analysis after 1200 participants in total were recruited and after every 600 participants thereafter. However, plans for interim analyses have been dropped. With 200 participants per active arm and corresponding concurrent controls the study has 85% power to detect a Cohen's D of 0.3 on FAS (difference between means in two treatment arms divided by the standard deviation of the data) at the 0.05 alpha level. If this number is increased to 300 for the same effect size the power is 95%.

Recruitment and sample size

To plan and cost the trial a nominal maximum of 4520 recruited participants was proposed based on up to 4 (drug and usual care) parallel arms of up to 1130 evaluable participants per arm. The nested drug trial aims to recruit up to 4520 patients from a possible pool of 30 000 patients from 6-10 PCN areas of England. The trial will continue to recruit participants at least until the recruitment target of 1130 participants for each of the initial arms of the adaptive trial has been reached. If the adaptive trial requires the full 4520 participants, it is estimated it will take approximately 10 to 12 months for the recruitment to complete and by the 16th month from the start of the trial, the last patient last visit would be completed, in third quarter of 2023. The projected recruitment rate is 600 participants per month and approximately 100 participants per month per clinic will need to be consented for data collection into the cluster randomised trial.

Revised sample size calculation main study

Lower than expected recruitment numbers have necessitated a partial decoupling of the cluster trial and the drug trial and the inclusion of drug only centres that do not contribute participants to the cluster trial. Patients previously seen at an integrated care clinic will also be able to join the drug trial, but not the cluster trial. It has been necessary to make the assumption that there will be little interaction effect between the drugs of the platform trial and the main and interaction effects in the 2x2 factorial trial. With this assumption, the total number of participants required for the interaction effect within the factorial (cluster) trial to be detected with 90% power is 1130, regardless of their participation in the drug trial or specific treatment allocation within the drug trial.

Revised sample size calculation drug trial

Lower than expected recruitment numbers have necessitated a partial decoupling of the cluster trial and the drug trial. The drug trial can no longer be considered nested within the cluster trial. The sample size calculation for the drug trial is not altered by the inclusion of clinics that can only offer the usual care treatment pathway, but can offer participants the opportunity to join the drug trial. The calculation is also not altered by the inclusion of patients previously seen at an integrated care clinic. With 200 participants per active arm (irrespective of participation in the cluster trial or not) and corresponding concurrent controls the study has 85% power to detect a Cohen's D of 0.3 on FAS at the 0.05 alpha level. If this number is increased to 300 for the same effect size the power is 95%.

3.6. Framework

STIMULATE-ICP is a superiority trial. For each of the outcomes, the null hypotheses are that there are no differences in effects on fatigue between any of the treatment pathways, and that there are no differences in effects on fatigue between any of the drug arms relative to usual care (no drug).

3.7. Outcomes

3.7.1. Primary outcome

Fatigue is the dominant symptom in 60% of patients and Long COVID clinics are being commissioned to provide rehabilitation for 12 weeks or less, therefore, a 12-week outcome is a pragmatic primary outcome measure. Fatigue Assessment Scale (FAS) is a 10-item, validated questionnaire in patients with chronic diseases. Therefore, it is a pragmatic primary end point for the STIMULATE-ICP trial. Five questions reflect physical fatigue and five questions (questions 3 and 6-9) reflect mental fatigue. The total score ranges from 10 to 50.

3.7.2. Secondary outcomes

Most secondary outcomes will be assessed at baseline and 12 and 24 weeks as can be seen in section 3.10.

1. FAS at 24 weeks
2. Health related quality of life (EQ-5D-5L)
3. Mental health (GAD-7)
4. Depression (PHQ-9)
5. Medical Research Council Dyspnoea Score
6. Perceived Deficit Questionnaire (PDQ-5)
7. Work and Social Adjustment Scale (WSAS) [Question 4 from Productivity Cost Questionnaire (iPCQ) for absenteeism and Question 8 from iPCQ for presenteeism added]
8. Short Form Questionnaire (SF-12)
9. Cognitive Failure Questionnaire (CFQ) if a patient scores 3 or more on PDQ5 (patients receive an email to complete this questionnaire online via a secure password and patient ID number)
10. Organ impairment and healthcare utilisation
11. Cost-effectiveness of ICP
12. Process outcomes for different ICP components

13. Blood investigations (e.g., genomic, proteomic, metabolomic/ lipidomic, functional T cell and live virus neutralisation assays and endocrine analyses)
14. Linked electronic health record data to monitor healthcare utilisation and outcomes.

3.8. Interim analyses, IDMC and stopping rules

Interim analyses were originally expected to occur after 1200 participants were recruited and after each additional 600 participants were recruited as mentioned briefly in 3.7 and described more fully in 6.8. Given the changes in the design to partially decouple the cluster and drug trials and the revision of the analysis populations for both trials, no interim analyses will be completed for the cluster trial. The final analysis at the end of the cluster trial will be at the conventional 0.05 two-sided level. Given the lower than expected recruitment, all interim analyses were also dropped from the drug trial. The p-value for the final drug trial analysis remained at the 0.05 two-sided significance level.

The role of the IDMC is to provide independent advice on accumulating comparative efficacy data and safety aspects of the trial. The Committee will meet before the trial starts recruiting and then every 12 weeks, until the end of data analysis, to review the trial conduct and data from interim analyses, or as necessary to address any issues. The IDMC is advisory to the TSC and Sponsor and can recommend premature closure of the trial to the TSC.

During the study, interim analyses were intended to be supplied in strict confidence to the IDMC. The IDMC will schedule reviews/meetings according to the emerging data from this and other studies. The IDMC will independently evaluate these analyses and any other information considered relevant. The IDMC will determine if, in their view, the randomised comparisons in the study have provided evidence that is strong enough (with a range of uncertainty around the results that is narrow enough) to affect the design of the trial moving forwards. In such a circumstance, the IDMC will inform the TSC who will make the results available to the Sponsor and, if deemed acceptable by the Sponsor and the funder, amend the trial arms accordingly.
The trial may be stopped before completion for the following reasons:

- On the recommendation of the TSC or IDMC (see section 13.3 of protocol)
- On the recommendation of the Sponsor and CI

Later changes to the protocol, reflecting the lower rate of recruitment, removed the requirement for interim analyses.

All safety data will be reviewed and a decision on continuation will be made by the IDMC with input from the Sponsor. It was agreed with the IDMC that as there is no standard effective treatment for long COVID, the focus for stopping should not be effectiveness/futility, and instead stopping should be for reasons of ethics, harm and safety. A single arm of the drug study may be stopped by the IDMC on the grounds of ethics, harm and safety at each stopping point.

Additionally, the IDMC may recommend a single arm of the drug study be halted in the event of the death of a single participant that is directly attributable to the study drug.

3.9. Timing of analyses

It is expected that the last patient's primary endpoint data collection will be 12 weeks after the final recruitment. All CRFs for primary endpoint data should be available with 6 weeks of this date. Data cleaning and raising of data queries will be ongoing during the course of the trial as described in the

Data Management Plan. In addition to cleaning and checks by the Data Management Team at LCTU, there will be a monthly data download to the Statistics Team at LCTU to enable further data checking and raising of queries. Final cleaning will commence once this last patient's CRF for 24 weeks is entered into the database.

The final analysis will start during the soft lock when all data for 12 and 24 weeks is entered into the database and all corresponding data cleaning queries are resolved. Hard data lock will occur after resolving further queries in returns for 12 and 24 weeks.

3.10. Timing of assessments

All assessments including outcome measures are scheduled to be collected as shown in Table 2 below. The visiting windows during which the information can be collected are given in the table.

Table 2: Schedule of Assessments

	**STIMULATE-ICP Trial Schedule of Assessments**					
	**Procedures**	**Referral to LCC and /or Coverscan TM***	**First Long COVID Clinic Visit**	**Baseline/ Visit**	**Treatment / Assessments**	**Follow Up / Assessments**
**Visiting window**			**Day 0**	**No later than 7-10 days post clinic***	**12 weeks (-/+7 days) *****	**24 weeks (-7days/+6 weeks) ******
**Visits No**			**0**	**1**	**2**	**3**
	**Informed consent**		**X***			
**Patient's History**	**Demographics**			**X**		
	**Medical history**			**X**		
	**Concomitant Medications**			**X**		
**Physical examination**	**Height, Weight, Temperature**			**X**		
	**Resting Pulse and BP**			**X**		

	STIMULATE-ICP Trial Schedule of Assessments					
	**Procedures**	**Referral to LCC and /or Coverscan TM***	**First Long COVID Clinic Visit**	**Baseline/ Visit**	**Treatment / Assessments**	**Follow Up / Assessments**
**Visiting window**			**Day 0**	**No later than 7-10 days post clinic***	**12 weeks (-/+7 days) *****	**24 weeks (-7days/+6 weeks) ******
**Laboratory Tests[1]**	**FBC**			**X**		
	**LFT**			**X**		
	**Urine pregnancy test[5]**			**X**		
	**COVID-19 Antibody**			**X**		
**Sample Collections**	**Samples of blood for biobank storage at Perspectum, Genomics, proteomics, metabolomic & Lipidemic, functional T cell and live virus Neutralisation antibody, Endocrine analysis and**			**X**	**X[6]**	
**Cardiovascular**	**(ECG, ECHO, Holter Monitor, Stress Electrocardiogram)[1]**			**X**		
**Pulmonary**	**CXR or CMR, CT Pulmonary angiogram[1]**			**X**		

	STIMULATE-ICP Trial Schedule of Assessments					
	Procedures	Referral to LCC and /or Coverscan TM*	First Long COVID Clinic Visit	Baseline/ Visit	Treatment / Assessments	Follow Up / Assessments
Visiting window			Day 0	No later than 7-10 days post clinic*	12 weeks (-/+7 days) ***	24 weeks (-7days/+6 weeks) ****
	6 Minute Walk Test, 1-minute Sit to Stand test,			X	X	X
	PFT (FeNo)[1]			x		
CNS	MRI of brain[1]			X		
	Tilt Table Test			X		
Whole Body	CoverscanTM2* **	X				
Inclusion and Exclusion	Eligibility assessment			X		
	Randomisation iv			X		
IMP	Dispensing of trial IMP [3]			X		
	Accountability				X	
Patient Reported Outcome Assessments	FAS Assessment			X	X	X
	MRC Dyspnoea Score			X	X	X
	General Anxiety Disorder Questionnaire-7 (GAD-7)			X	X	X
	The Primary Care Evaluation of Mental			X	X	X

	STIMULATE-ICP Trial Schedule of Assessments					
	**Procedures**	**Referral to LCC and /or Coverscan TM***	**First Long COVID Clinic Visit**	**Baseline/ Visit**	**Treatment / Assessments**	**Follow Up / Assessments**
**Visiting window**			**Day 0**	**No later than 7-10 days post clinic***	**12 weeks (-/+7 days) *****	**24 weeks (-7days/+6 weeks) *******
	**Disorders Patient Health Questionnaire (PHQ-9)**					
	**EQ-5D-5L (EUROQOL-5 domain- 5 level)**			**X**	**X**	**X**
	**Perceived Deficit Questionnaire) PDQ-5**			**X**	**X**	**X**
	**SF12**			**X**	**X**	**X**
	**Cognitive Failure Questionnaire (CFQ)** [4]			**X**	**X**	**X**
	**Modified Work and Social Adjustment Scale (WSAS)**			**X**	**X**	**X**
**Safety Assessments**	**Adverse Event Reporting**				**X**	**X**[8]
**Concomitant medication**	**Concomitant medication review**				**X**	**X**[7]

*Newly referred patients who have consented to the drug study need to have their baseline appointment no later than 7-10 days post clinic. This is to align the administration on IMP with follow-up data collection and clinic pathway. Patients who do not wish to consent to the drug arm

but do wish to consent to data and research blood arms only (integrated care sites) may be consented and enrolled up to 2 weeks after their first clinic appointment and gift blood samples up to 12 weeks post clinic appointment.
**Coverscan as usual care for allocated clusters
*** The first follow-up assessment is 12 weeks from date of randomisation, or date of consent/enrolment if data collection only
****Follow up assessment 24 weeks from date of randomisation, or date of consent/enrolment if data collection only
1: If requested by the treating physician at Long COVID Clinics as part of their routine care
2: Only if recruited from a site randomised to Coverscan™ cluster
3: If randomised to receive the trial drug
4: if the participant scores $\geq$3 on PDQ5 questionnaire
5: For female participants of childbearing potential randomised to drug arm of the trial
6: Blood samples collected for functional T cell and live virus neutralisation assays from London Participants only at baseline and 12 weeks. All other participating centres will collect blood for external laboratory analysis once at baseline visit. Permission will be sort from all participants to recontact to participate in further testing due to any changes to the study on the basis of emerging evidence/results
7. Concomitant medications will be reviewed at 12- and 24-weeks visit is only for participants to the nested drug trial
8. Adverse events will be collected at the 24 week visit for participants on the nested drug trial only.

Randomisation to the drug arm can only be after clinic attendance. The participant will be consented face to face and randomised following confirmation of eligibility, this will be at the same visit if possible.

4. STATISTICAL PRINCIPLES

4.1. Confidence and significance levels

Uncertainty around point estimates will be presented using two-sided 95% confidence intervals. Significance testing will be two sided. For the primary outcome a 5% significance level will be adopted at the final analysis. Secondary outcomes will be tested at the final analysis time points using a 5% significance level.

4.2. Adherence and protocol deviations

4.2.1. Adherence

Sites will be monitored to confirm adherence to the protocol.

The screening log will be used to check that participants consenting to data collection and recruited to the drug trial reflect those that are eligible. Statistical summaries of baseline characteristics age, gender at birth and ethnicity will be presented.

4.2.2. Adherence to treatments

The number and proportion of patients who did not receive the treatment to which they were allocated will be reported by treatment pathway (for cluster trial participants) and by treatment arm (for drug trial participants). Participants will be asked at the long COVID clinic about whether they have had a Coverscan™. Participants will be asked about enrolment on the digital app. Frequency of their access (within 12 and 24 weeks) will be available from Living with COVID Recovery™ for those who enrolled.

At 12 weeks the participant will be asked for the number of tablets remaining. if there are tablets remaining, the participant will be asked the reason why (if there were any temporary interruptions, if the participant stopped taking the medication permanently or if they have not finished the course yet). If the participant reports temporary interruptions, they will be asked for the reason why (e.g. if tablets were stopped to take other medications like antibiotics). If the participant stopped taking the medication permanently, they will be asked for the reason why (reaction, preference, etc.). At week 24, the participant will be asked how many tablets they have remaining and how they disposed of any remaining tablets.

4.2.3. Definition and summary of protocol deviations

A protocol deviation is defined as an unintended departure from the expected conduct of the trial. A protocol violation log form will be used to capture deviations, violations, potential serious breaches, serious breaches and urgent safety measures. Deviations from protocol by staff, identified during monitoring will be addressed by additional training. No deviations from protocol will result in exclusion of participants from analyses.

4.3. Analysis populations

The main analyses of the primary and secondary outcomes was specified as a complete-case analysis. However, there are different understandings of this terminology and complete case can be taken to mean only participants with all data measures available. With a very large number of recorded variables this is an unrealistic expectation. The intended analysis was to use all available primary outcomes (available case analysis), according to the cluster random allocation of the participant's PCN (regardless of treatment pathways received) or the stratified randomisation of the individual participant (regardless of treatment received). There are 4 key analysis populations under consideration which are described below. These analysis populations will be used for analysis of efficacy and safety data. Figures 1 and 2 show the CONSORT diagram and the same diagram with analysis populations 1-4 depicted using coloured dashed and solid bordered boxes. Figure 3 indicates the analysis populations in a Venn diagram form.

2x2 factorial cluster trial

In the principal analysis population of treatment pathways (Coverscan™, digitally enabled community rehabilitation, and their interaction) for the primary outcome (FAS at 12 weeks), the analysis population was originally intended to be participants attending a long COVID clinic for the first time aged ≥18, who consented to data collection for the cluster RCT and were allocated to receive usual care (after being randomly allocated to usual care (no drug) in the drug trial, or receiving usual care due to ineligibility for the drug trial, or being willing for data collection, but not willing to participate in the drug trial).

The principal analysis population (analysis population 1) for the estimates of the effects of Coverscan™, digitally enabled community rehabilitation, and their interaction, has been changed to reflect lower than expected recruitment and will be those participants who consented to data collection for the cluster RCT regardless of their participation in the drug trial or their drug trial treatment allocation. This analysis will provide a generalisable answer to the primary objective in the broadest group of participants in the presence of other treatments including drug treatments allocated in the trial or obtained another way.

A similar and more pragmatic analysis of the effects of Coverscan™, digitally enabled community rehabilitation, and their interaction, was originally intended to be applied to all participants who consent to data collection in the cluster trial regardless of participation or treatment allocation in the drug trial. This analysis was intended to provide an answer to the primary objective in the presence of other treatments including drug treatments and will allow comparison to the results from the principal analysis set. This analysis was expected to be balanced between the 2x2 factorial treatment pathways. However, within each treatment pathway, there was expected to be more people on allocated to no drug (by randomisation in the drug trial, by contraindication to at least one of the drugs or by refusing the drug trial) than on any one of the drug arms.

The second analysis population (analysis population 2) has been changed to reflect lower than expected recruitment. A similar multi-level model analysis of the effects of Coverscan™, digitally enabled community rehabilitation, and their interaction, will be applied to all participants who consent to data collection in the cluster trial restricted to those taking no drug, through random

allocation to no drug, through ineligibility or through refusal of participation in the drug trial. This analysis, although having much lower power than originally planned, will provide an answer to the primary objective in the absence of other treatments including drug treatments and will allow comparison to the results from the principal analysis set. Those participants not in the drug study may have more comorbidities and contraindications than those in the drug study. There could be people with particularly high fatigue responding in all treatment pathways. If some drugs work, there could be people responding with particularly low fatigue. The ranges of fatigue in every treatment pathway could be larger in this analysis than in the principal analysis of treatment pathways within those allocated to receive no drug.

Drug trial

To ensure that the treatment policy approach comparison of the drugs vs usual care is as unbiased and straightforward as possible, the principal analysis population for the drugs trial was originally intended to be all participants who were randomised into the drug trial (regardless of treatment received), who had previously been cluster randomised to the control treatment pathway, i.e. no Coverscan™ + usual care rehabilitation. It was recognised that this principal analysis would use only one quarter of those randomised into the drug trial. However, the original sample size for the drug trial was calculated to ensue 90% power for comparison of the treatment arms even within this single treatment pathway. However, the lower recruitment rate into the drug trial from the original six sites, necessitated a change in plan and the addition of clinics that could only offer the control treatment pathway (drug only sites) and inclusion of patients previously seen at a Long-COVID clinic at both the original sites and the drug only sites.

The principal analysis population (analysis population 3) for drug vs. usual care comparisons within the drugs trial will now be anyone randomised to the drug trial including participants also cluster randomised to any treatment pathway in the cluster trial, participants from cluster trial sites who have previously been seen at a clinic, and participants randomised into the drug trial from clinics that can only offer the control treatment pathway including both new clinic patients and those who have previously been seen at a clinic.

The second analysis population for the drugs trial to study the effects of the drugs in combination/interaction with the pathways will be the total population of participants who have been randomised into the drugs trial including those from all 4 treatment pathways of the cluster trial (analysis population 4). It is recognised that the power for this analysis will be lower than originally planned.

For both the cluster randomised trial and the drug trial, in an estimands framework, for all primary outcome analyses, the endpoint is FAS at 12 weeks, the population-level summary (adjusting for baseline (post-COVID) FAS score), and any intercurrent events, with the exception of death, will be addressed using a treatment policy approach. Death during follow up is expected to be rare in this patient group and as an intercurrent event will be addressed using a while-alive approach.

If a participant withdraws from the study, any de-identified data collected up to the point that they have been withdrawn from the trial will be kept in the trial for analysis. A participant can request any identifiable research data held by the research team to be destroyed. If withdrawal occurs after data has been anonymised and aggregated, it will not be possible to identify and remove individual data.

The safety population will be a) all patients consenting to data collection, and b) all patients randomised into the drug trial, with patients grouped for analysis according to the treatment they received, as opposed to the treatment they were allocated to receive. For a) this grouping is according to the combination of scan and app received rather than offered on the basis of participant GP postcode. For b) this grouping for analysis is by drug treatment, or no drug treatment, received rather than allocated. Anyone receiving a single dose of a drug treatment would be analysed as receiving this treatment.

5. TRIAL POPULATION

5.1. Screening, recruitment, withdrawal/follow-up

The throughput of patients from those screened, consented for data collection in the cluster trial, recruited and randomised into the drug trial, assessed for trial endpoints, and included in the analysis, will be summarised in a CONSORT flowchart.

The number of patients who have been withdrawn or were unwilling to continue trial follow up will be reported by treatment arm.

Figure 1: STIMULATE-ICP CONSORT flow chart

STIMULATE-ICP CONSORT Flow Chart *

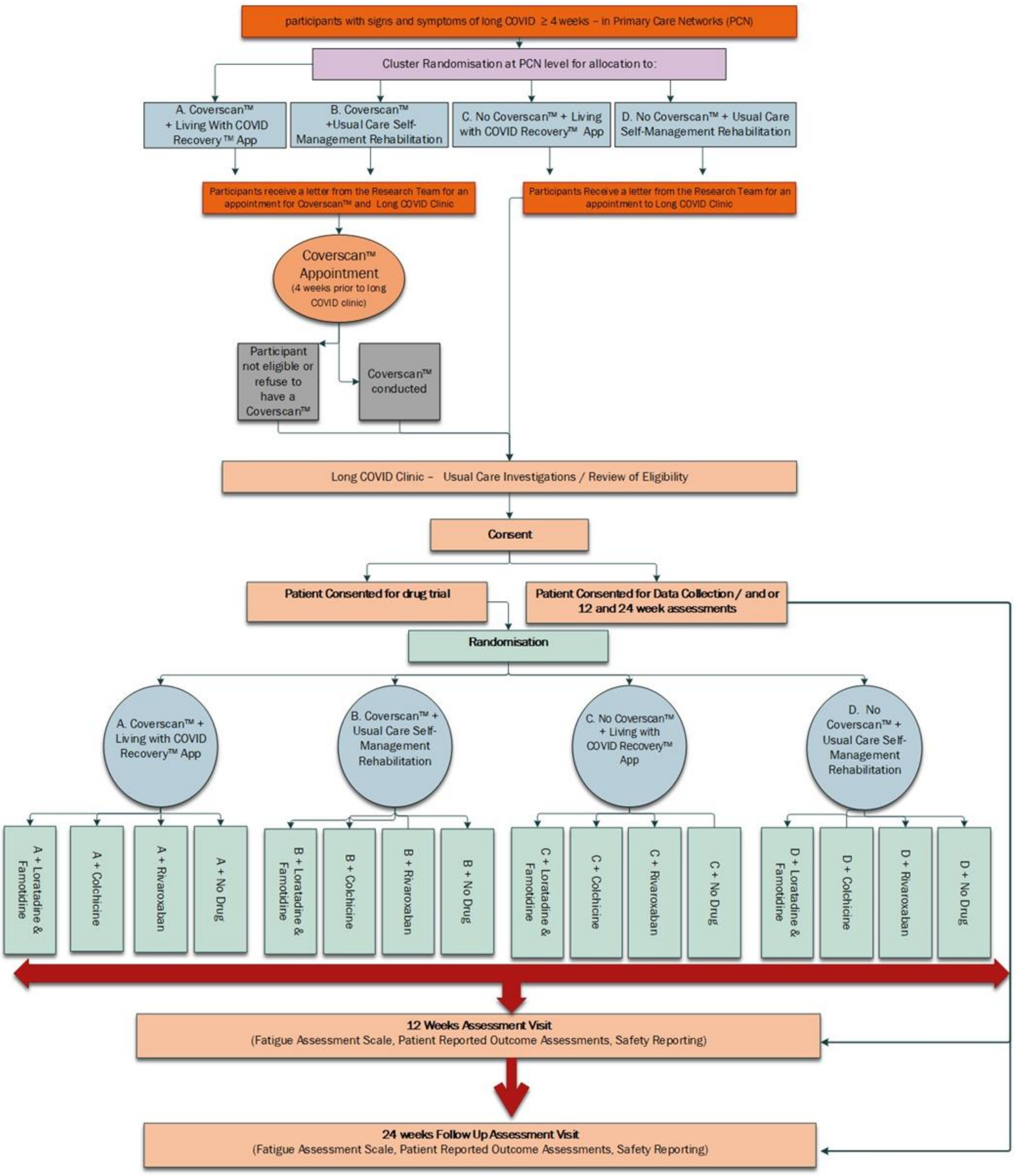

* Patients recruited through a site taking part in the drug study only will not undergo the cluster randomisation element or activities. Their pathway will start at the Long COVID Clinic appointment, and they will be randomised to one of the four drug arms.

Figure 2. Figure showing principal analysis sets 1 and 2 for the cluster trial (green dashed rectangle) and the cluster trial restricted to those allocated to no drug (blue rectangle)

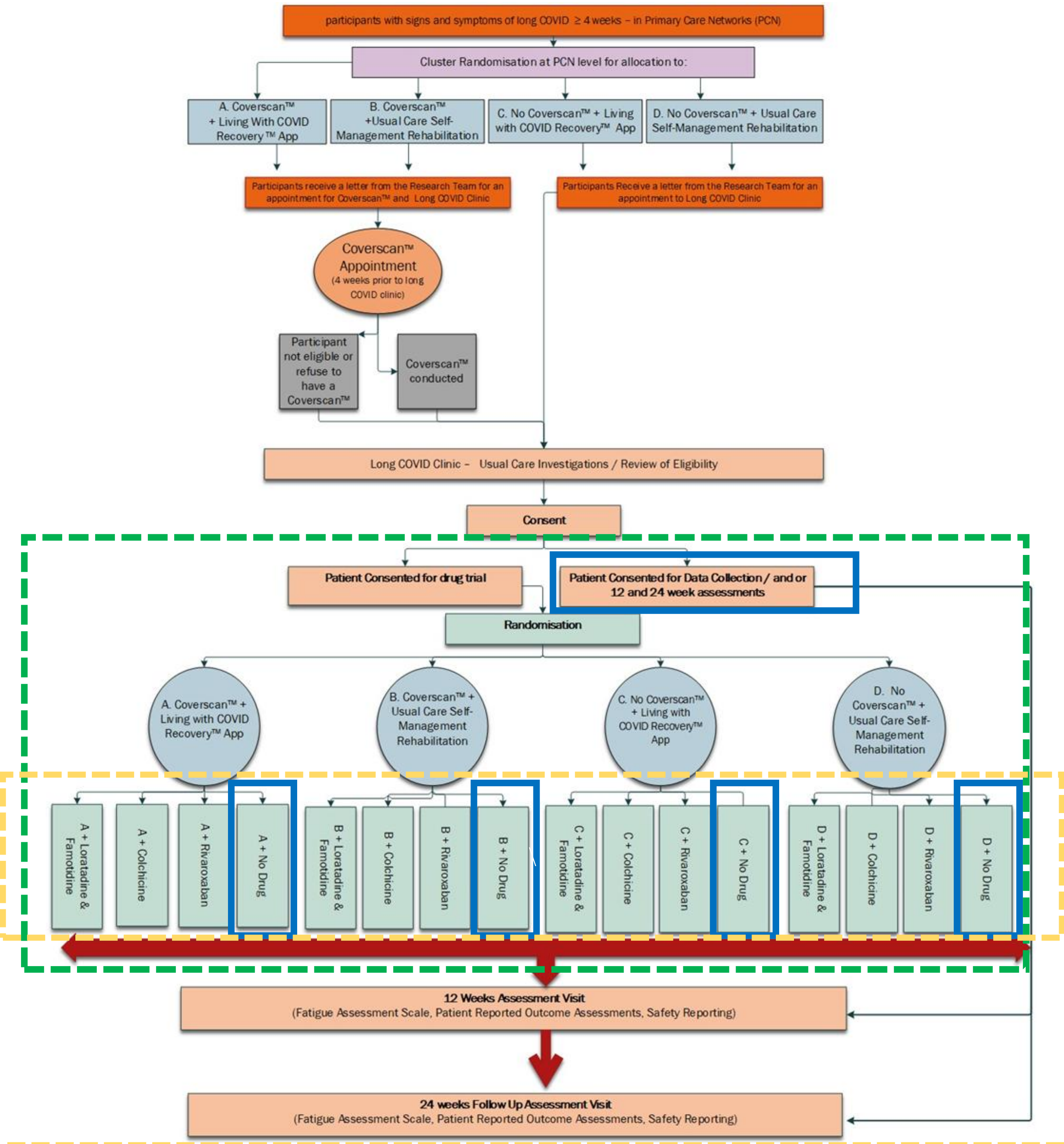

* Patients recruited through a site taking part in the drug study only will not undergo the cluster randomisation element or activities. Their pathway will start at the Long COVID Clinic appointment, and they will be randomised to one of the four drug arms.

Figure 3. Analysis populations represented using a Venn diagram

Analysis Population 1<br>Cluster trial:<br>Pop1 = 1 if Site is 1 to 6<br>&<br>Cluster is not "E – Returning Participant"<br>&<br>the registration form does not indicate "Participant from out of area".	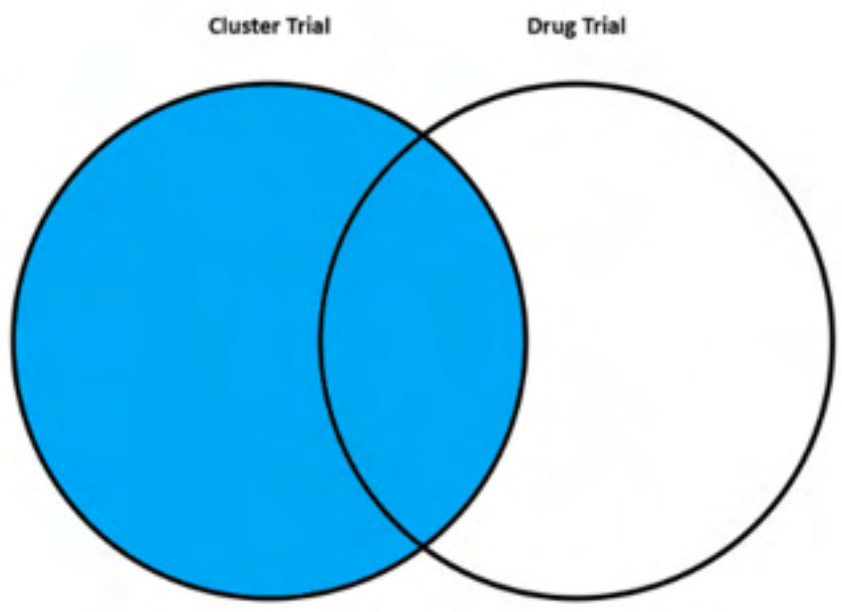
Analysis Population 2<br>Cluster trial, restricted to those not in the drug trial or allocated to usual care within the drug trial:<br><br>Pop2 = Pop1 excluding those with drug trial allocation (Group) of "Famotidine and Loratadine", or "Colchicine", or "Rivaroxaban".	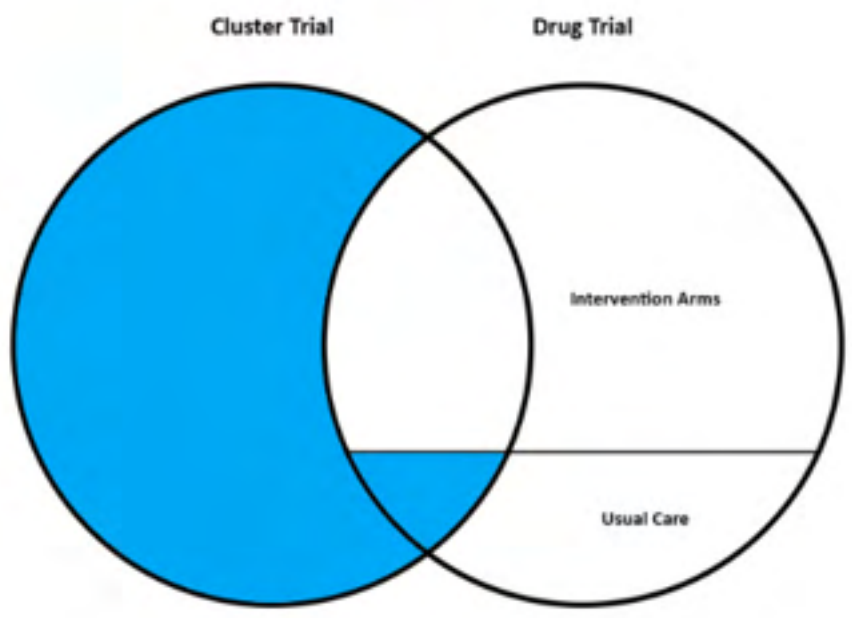
Analysis Population 3<br>Drug trial:<br><br>Pop3=1 if the drug trial allocation from the randomisation form (Group) is present.	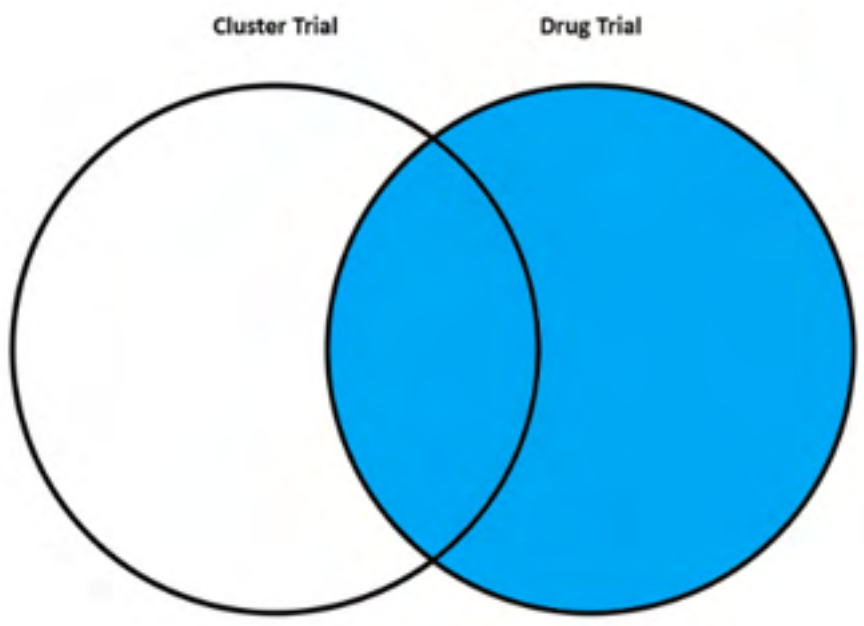
Analysis Population 4<br>Drug trial limited to those also in the cluster trial:<br><br>Pop4 = 1<br>if Pop1 = 1 & Pop3 = 1.	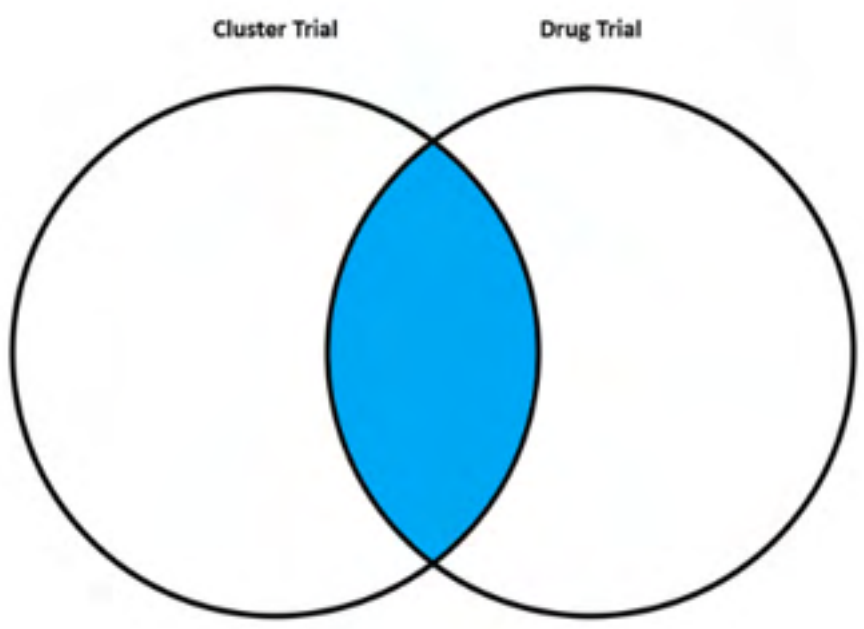

5.2. Eligibility

Eligibility and inclusion/exclusion criteria are also provided in the protocol in section 7.2.
Inclusion criteria:
Participant Inclusion Criteria for ALL Participants

1. Participants capable of giving informed consent.
2. Age 18 years and above
3. Clinical Parameters; persistent signs and symptoms for a period of 4 weeks or longer in duration post-COVID-19 infection (either by test result or symptomology). Presenting at their first referral first visit to a participating Long COVID clinic pathway.
4. Able to read or understand English or have a relative/family member able to read/understand English to facilitate participation (essential for patient reported outcome measures at follow-up time points and virtual contact).
5. Not enrolled in any other interventional study where study intervention/activities may affect outcome measures (patients enrolled in purely observational studies can be included)

Additional Participant Inclusion Criteria for the cluster randomised integrated care pathway study

6. Presenting at their first referral to a participating Long COVID clinic pathway.

Additional Participant Inclusion Criteria for the nested, platform randomised drug trial
<u>(to be eligible participants must meet **all above criteria** and all those below)</u>
Note: Potential participants with drug-specific contraindications for any arm, including interactions of pre-prescribed essential medication will be consented for data collection but will be excluded from the drug study.

7. Presenting at their first referral to a participating Long COVID clinic pathway or previously referred to a participating Long COVID clinic and still suffering from Long COVID symptoms
8. Females of childbearing potential must be willing to use an acceptable, effective method of contraception during the treatment with IMP. Such methods include:

i. combined (oestrogen and progestogen containing) hormonal contraception:
a) oral
b) intravaginal
c) transdermal
ii. progestogen-only hormonal contraception
a) oral
b) injectable
c) implantable
iii. intrauterine device (IUD)
iv. intrauterine hormone-releasing system (IUS)
v. bilateral tubal occlusion
vi. vasectomised partner
vii. male or female condom with or without spermicide
viii. cap, diaphragm or sponge with spermicide
ix. sexual abstinence (true abstinence)

9. Male participants must be willing to use condom during IMP treatment and for a further 90 days after the last dose of trial IMP to protect their female partner becoming pregnant.

10. Patients on pre-existing treatments for the same drug classes MUST undergo a 7-day washout period before being randomised.
(Patients will be assessed, and if safe to exclude that medication for 7 days, they will be asked if they would be willing to undergo a washout period of at least 7 days after consent but before being randomised and exclude that medication until 7 days after last dose of IM.)

Exclusion criteria:
Participant Exclusion Criteria for ALL Participants

1. Previously hospitalised for COVID-19 infection.

Additional Participant Exclusion Criteria for Research Blood Sampling:

2. Participants with any known blood borne viruses (BBV's) including Hepatitis C, Hepatitis B and/or HIV
3. Participants with any condition that may severely prolong clotting times

Additional Participant Exclusion criteria for nested, adaptive randomised drug trial

4. Females who are pregnant, planning pregnancy or breastfeeding
5. Known hypersensitivity to any of the study drugs or their excipients
6. Currently taking any of the following drugs:

Probenecid, Sucrafate, Posaconazole oral-suspension, Dasatinib, erlotinib, gefitinib, pazopanib (for further specific recommendations please refer to product information of individual TKIs product information, Isocarboxazid, Phenylzine, Tranylcypromine of any other CNS depressant (such as diphenhydramine, dextromethorphan, or pseudoephedrine) (Contraindications to famotidine/loratadine)

Amiodarone, Aprepitant, Atanazavir, Atorvostatin, Azithromycin, Bezafibrate, Ciclosporin, Ciprofibrate, Clarithromycin, Cobicistat, Croztibib, Darunavir, Diltiazem, Dronedarone, Eliglustat, Erythromycin, Fenobibrate, Fluconazole, Fluvastatin, Fosamprenavir, Gemfibrozil, Idelalisib, Imatibib, Isavuconazole, Itraconazole, Ketoconazole, Letermovir, Lopinavir, Netupitant, Nilotinib, Posaconazole, Pravastatin, Ranolazine, Ritonavir, Rosuvastatin, Simvastatin, Tipranavir, Velpatasvir, Vemurafenib, Venetoclax, Verapamil, Voriconazole *(Contraindications to colchicine)*
Acalabrutinib, Aceclofenac, Acenocoumarol, Alprostadil, Alteplase, Argatroban, Aspirin, Axitinib, Beniparin, Benzydamine, Bevacizumab, Bismuth, Bivalirudin, Bosutinib, Bromfenac, Cabozantinib, Cangrelor, Caplacizumab, Celecoxib, Cilostazol, Clopidogrel, Cobimetinib, Dabigatran, Dalteparin, Danaparoid, Dasatinib, Dexkeptorofen, Diclofenac, Dipyridamole, Enoxaparin, Epoprostenol, Eptifibatide, Erlotinib, Etodolac, Etoricoxib, Flurbiprofen, Gefitinib, Heparin, Ibrutanib, Ibuprofen, Iloprost, Imatinib, Indomethacin, Inotersen, Ketoprofen, Ketorolac, Levatinib, Mefenamic acid, Meloxicam, Nabumetone, Naproxen, Nicotinic acid, Nintenanib, Parecoxib, Pazopanib, Phenazone, Phenindione, Piroxicam, Ponatinib, Prasugrel, Regorafenib, Ruxolitinib, Sorafenib, Streptokinase, Sulindac, Sunitinib, Tenecteplase, Tenoxicam, Tiaprofenic acid, Ticagrelor, Tinzaparin, Tirofiban,

Tolfenamic acid, Trametinib, Traztuzumab emtansine, Trprostinil, Urokinase, Volanesorsen, Warfarin (Contraindications to Rivaroxaban)

7. No history or presenting symptomology suggestive of Renal failure/insufficiency (eGFR<30 mL/min) on the basis of blood investigations (eGFR) within the last 6 months and clinical assessment
8. Severe liver dysfunction on the basis of blood investigations within the last 6 months (liver function and coagulation) and clinical assessment
9. Patients with blood dyscrasias

5.3. Recruitment and retention

The number of patients who have been withdrawn or were unwilling to continue trial follow up will be reported by treatment arm.

The throughput of patients from those screened, consented for data collection in the cluster trial, recruited and randomised into the drug trial, assessed for trial endpoints, and included in the analysis, will be summarised in a CONSORT flowchart.

Figure 1: STIMULATE-ICP CONSORT Flow Chart

5.4. Baseline characteristics and demographics

A CONSORT diagram will be produced to report of the flow of participants in the cluster trial. There will be a description of screening log data for eligible participants. There will be a further summary of baseline characteristics of all participants that consent to data collection.

Key baseline characteristics and demographics will be summarised for all participants and by cluster trial arms (treatment pathways: Coverscan™ and digitally enabled community rehabilitation, Coverscan™, digitally enabled community rehabilitation, usual care) and by drug trial arms (drug allocations) using descriptive statistics as follows:

- Mean (SD) if continuous (approximately normally distributed or not highly skewed).
- Median and interquartile range for non-normally distributed data or counts.
- Frequency and percentage for categorical variables or ordinal as appropriate.

Characteristics and demographics will include the following:

A. Information collected from baseline examination or case notes:

- Height, Weight, Temperature, Resting Pulse and BP, FBC, LFT
- 6 Minute walk test, 1-minute sit to stand test
- Other tests as listed in section 3.10

B. Information collected from the participant,

- Date of birth (only integer age at trial registration will be shared with the statistics team for statistical analysis, Ethnicity, Birth gender, Self-identified gender, Religion, Highest level of Education, Smoking status (ever and current), Alcohol use,
- Prior to COVID ability to leave the house, and frequency of leaving the house, Reasons to leave the house, Main mode of transport, and the same variables now and, any reasons for a reduction in leaving the house
- Employment, Changes in employment due to COVID (yes/no), and How changed

- Prior to COVID household composition, Social contact beyond the household, Communication beyond the household, Communication methods, and the same variables now
- Screening questions: Illnesses prior to COVID, Menstrual status (if female), Current medications used, Current supplements taken
- Details of first having COVID, Test status at that time, Details of more recent infection with COVID and More recent test status, Other viral infections details, Vaccination status and details including type and dates, Vaccination side effect details
- Medical contacts/resource use at the time of having COVID, Reasons for contact, Method of contact
- Pre-COVID and current (within last 4 weeks) symptoms and severities, and Whether or not resolved
- EQ-5D-5L pre-COVID and after COVID
- Visual analogue health scale pre-COVID and after COVID
- SF-12
- MRC Dyspnoea Score pre-COVID and now
- FAS pre-COVID and now (after having COVID)
- PHQ-9, GAD-7
- Work and Social Adjustment Scale (WSAS) After COVID plus Question 4 from Productivity Cost Questionnaire (iPCQ) for absenteeism, and Question 8 from iPCQ for presenteeism added
- Perceived Deficits Questionnaire (PDQ-5), Cognitive Failure (CF) questionnaire
- Stigma and public perception of Long COVID symptoms
- Engagement (yes/no) and frequency of Engagement with digital media around long COVID and treatment

6. ANALYSIS

The trial will be analysed and reported using the “Consolidated Standard of Reporting Trials” (“CONSORT”) considering CONSORT 2025 (29), and where appropriate, CONSORT extensions for cluster trials (30), the factorial randomised trials (31) and adaptive designs (32) and the International Conference on Harmonisation E9 guidelines. The main features of the statistical analysis plan are included here.

6.1. Analysis of baseline characteristics

The data will be analysed by site and overall. Baseline characteristics will be summarised, with appropriate numerical summaries for data type measure, and reported for each intervention arm.

Patient reported baseline data reported as a range rather than as a numerical value will be taken as the mid-point of the range. Patient reported baseline data unexpectedly reported as less than a number will be taken as that number, e.g. “<1” cigarette per day will be taken as “1” cigarette per day in statistical analyses. Site staff reported baseline clinical data where more than 10% of responses are reported using greater than a number or less than a number, e.g. renal function (eGFR), <15 or ≥90 ml/min/1.73m², will be reported as categorical using the categorisation most frequently adopted in the clinical literature, e.g. <15, 15-29, 30-59, 60-89, ≥90 ml/min/1.73m², stages 1-5 (33).

6.2. Generation of derived variables

Primary outcome FAS

The FAS is a 10-item scale evaluating symptoms of chronic fatigue. The FAS treats fatigue as a uni-dimensional construct and does not separate its measurement into different factors. Each item of the FAS is answered using a five-point, Likert-type scale ranging from 1 ("Never") to 5 ("Always") Additional guidance is given for categories 2. Sometimes (about monthly or less), 3. Regularly (about a few times a month), 4. Often (about weekly), and 5. Always (about every day). Items 4 and 10 are reverse-scored. Total scores can range from 10, indicating the lowest level of fatigue, to 50, denoting the highest. An answer to every question has to be given, even if the person does not have any complaints at the time of completion. Scores on questions 4 and 10 should be recoded (1=5, 2=4, 3=3, 4=2, 5=1). Subsequently, the total FAS score can be calculated by summing the scores on all questions (recoded scores for questions 4 and 10).

"The following ten statements refer to how you usually feel. Per statement you can choose one out of five answer categories, varying from Never to Always.

1. I am bothered by fatigue
2. I get tired very quickly
3. I don't do much during the day
4. I have enough energy for everyday life
5. Physically, I feel exhausted
6. I have problems to start things
7. I have problems to think clearly
8. I feel no desire to do anything
9. Mentally, I feel exhausted
10. When I am doing something, I can concentrate quite well"

The plan for missing data is described in detail in section 6.8. Although the creators of the FAS do not recommend an algorithm for one missing FAS item, it was decided by the trial statistics team (and checked with the TSC/IDMC) that a single missing item will be replaced by the average of the other 9 FAS items (effectively scoring as usual and multiplying by 10/9) to allow a total FAS score to be calculated.

FAS at 24 weeks is calculated as for FAS at 12 weeks. The detailed derivation of the other secondary outcomes included in statistical analysis will be described separately in a data derivation document (appendix 1).

6.3. Primary outcome analysis

The primary analysis of the trial will be on the basis of the cluster randomisation of the Participant's PCN or the stratified randomisation of the individual participant. The primary outcome, FAS at 12 weeks, is expected to be approximately normally distributed (40). A multi-level model analysis will

be used to evaluate effects of Coverscan™, digitally enabled community rehabilitation, and their interaction, on FAS at 12 weeks adjusting for baseline (post-COVID) FAS, with gender as a fixed effect and clinic and PCN as random effects. Where PCN is a random effect, the strata variables binary IMD (deprivation: low ≥14500 vs. high <14500) and binary PCN patient population size (≥45000 vs.<45000) will also be fitted as fixed effects.

The principal analysis population (analysis population 1) for the multi-level model analysis of the effects of Coverscan™, digitally enabled community rehabilitation, and their interaction, has been changed to reflect lower than expected recruitment and will be those participants who consent to data collection in the cluster trial regardless of participation in the drug trial. These participants are shown in the green dashed rectangle in Figure 2. This analysis will provide an answer to the primary objective in the presence of other treatments including drug treatments. This analysis will provide the most straightforward answer to the primary objective in the broadest group of participants (regardless of the allocated drug treatment). The size and significance of the main effects of the ICP interventions will be interpreted considering the statistical significance, or not, of their interaction. The interaction term will be retained as there no statistical advantage to removing a non-significant interaction term in this context. More detail is given in 6.3.1.

The principal analysis population (analysis population 2) for these estimates of the effects of Coverscan™, digitally enabled community rehabilitation, and their interaction, will be those participants who consented to data collection for the cluster RCT and were allocated to receive usual care. These participants are shown in the blue rectangles in Figure 2. These can be participants willing to be in the drug trial and allocated by randomisation to usual care in the drug trial, or being allocated to usual care because they have contraindication to one of the drugs that means they cannot be randomised into the drug trial, or being willing for data collection, but not willing to participate in the drug trial. It is appropriate to consider just those participants receiving no drug in this principal analysis, as it is unknown whether there will be interactions between the drugs and the pathways. This analysis, although having lower power than originally planned, will provide an answer to the primary objective in the absence of other treatments including drug treatments and will allow comparison to the results from the principal analysis set. A multi-level model analysis will be used to evaluate effects of Coverscan™, digitally enabled community rehabilitation, and their interaction, on FAS at 12 weeks, in participants allocated to receive usual care, adjusting for baseline (post-COVID) FAS, with gender as a fixed effect and clinic and PCN as random effects. Where PCN is a random effect, the strata variables binary IMD and binary PCN patient population size will also be fitted as fixed effects. More detail is given in 6.3.2.

The principal analysis population (analysis population 3) for drug vs. usual care comparisons within the drugs trial will be 1) the participants randomised into the drug trial from any cluster treatment pathway, and 2) those participants randomised into the drug trial from clinics that can only offer the control treatment pathway and 3) those participants randomised into the drug trial who had previously been referred to a participating Long COVID Clinic. These participants are shown in Figure 2 as within the gold dashed rectangles. Participants randomly allocated to each drug arm will be compared to those randomly allocated to usual care while both that drug and usual care are options for randomisation (concurrent usual care controls). A multi-level model for FAS, adjusting for

baseline, with gender and treatment pathway as fixed effects and clinic as a random effect, will be applied to the drug trial data. More detail is given in 6.3.4.

Estimates of the differences in effect of the drugs vs. usual care, in the presence of the effects of the treatment pathways, may be obtained by using data from all of the participants in the drug trial who were also in the cluster trial (analysis population 4, the upper gold dashed rectangle). The comparisons will be made more complicated by doing so particularly as the three additional pathways are expected to have greater variation in their implementation than the control treatment pathway. The total population of participants who are randomised into the drugs trial and the cluster trial will be used to study the effects of the drugs in combination/interaction with the pathways. A multi-level model for FAS, adjusting for baseline, with gender as a fixed effect and clinic and PCN as random effects, will be applied to data from all participants of both the cluster trial and the drug trial to evaluate effects of Coverscan™, digitally enabled community rehabilitation, and their interaction, in combination with the participants' allocated drug treatment. It is expected that there is unlikely to be sufficient power to provide definitive answers to the research questions within analysis population 4. More detail is given in 6.3.5.

For all drug treatments, particularly any introduced after the start of the trial, comparisons with participants randomised to usual care will include only those participants who could have been randomised to the relevant drug treatment at the time of their randomisation. This will reduce the potential for bias and have the effect of ensuring that only contemporaneous participants allocated to usual care are compared to participants randomised to drug treatments.

Patterns of missingness will be summarised. In modelling, complete case analysis will be the main analysis. PHD-9 and GAD-7 both have standard algorithms for producing scores if 1-2 items are missing. For the primary outcome, FAS, a similar algorithm will be used to obtain a FAS score, even if one FAS item is missing. After reversing the two items that required this, the missing item should be replaced by the average of the 9 other items (effectively scoring as usual and multiplying by 10/9) to allow a FAS score to be calculated.

For missing outcome data, we will use a threshold approach as a sensitivity analysis. Imputation based on thresholds will be implemented such that missing values will be replaced by values from the lower and upper quantiles of the distribution, e.g. 90th quantile, 75th quartile, 25th quartile, 10th quartile, and minimum. If missing primary outcome data exceeds 10%, a multiple imputation analysis will be completed as described in 6.8.

After running the effectiveness analysis using the multi-level models, assumptions will be checked as follows:
For multiple multi-level linear regression:

- There is a linear relationship between the outcome and corresponding baseline measure (FAS).
- There is homogeneity of variance.
- Residuals are approximately normally distributed when estimating the effect of group allocation on the outcome, adjusting for the stratification variable in the randomisation process and baseline value of the outcome.

- There are no strong relationships between explanatory variables (multi-collinearity).

6.3.1. Analysis for the primary objective in the cluster trial

The overall primary objective is to evaluate the effect of "integrated care" with combinations of multi-organ MRI (Coverscan™) and clinical decision support, and digitally enabled community rehabilitation (Living with COVID Recovery™) versus "usual care" [Usual Investigations; and Your COVID Recovery™ self-management website] on Fatigue Assessment Score (FAS) at 12 Weeks from the enrolment date.

The treatment policy approach research question is about assessing the effect of offering Coverscan™, digitally enabled community rehabilitation, and their interaction, in comparison to usual care, on FAS at 12 weeks. This will be assessed in everyone assigned a pathway in the cluster randomised trial (analysis population 1), who provided a calculable primary outcome at 12 weeks and at baseline, regardless of receipt of the allocated pathway. A multi-level model analysis will be used to evaluate effects of Coverscan™, digitally enabled community rehabilitation, and the interaction of Coverscan™, digitally enabled community rehabilitation, on FAS at 12 weeks adjusting for baseline (post-COVID) FAS, with gender as a fixed effect and clinic and PCN as random effects (random intercepts). To account for any systematic effects of participating in the both the cluster and drug trial, an indicator term will be added to the model.

6.3.2. Analysis for the primary objective in the cluster trial restricted to those taking no drug

This is a repetition of the analysis described in 6.9.1 within analysis population 2 (blue rectangles in Figure 2). This will provide estimates of the effects of Coverscan™, digitally enabled community rehabilitation, and their interaction, within participants receiving no drug by allocation in the drug trial, or by refusing participation into the drug trial or by being ineligible for the drug trial. This is likely to be underpowered, but will provide comparison estimates of the ICP interventions in those participants on no drug or receiving a drug only as part of usual care to those estimates from the analysis of 6.9.1. To account for any systematic effects of participating in both the drug and cluster trial, an indicator term will be added to the model. A multi-level model analysis will be used to evaluate effects of Coverscan™, digitally enabled community rehabilitation, and their interaction, on FAS at 12 weeks adjusting for baseline (post-COVID) FAS, in cluster participants taking no drug, with gender as a fixed effect and clinic and PCN as random effects (random intercepts). Where PCN is a random effect, fixed effects of binary IMD and binary PCN patient population size will also be fitted.

6.3.3. Analysis for the secondary objective in the cluster trial taking account of receipt of the ICP interventions

A further analysis will address the secondary objective, the clinical efficacy of the individual components of the integrated care pathway, including potential therapies. This analysis will be assessed in those assigned a pathway in the cluster randomised trial, who provided a calculable primary outcome at 12 weeks and at baseline, restricted to those who received the Coverscan™, or registered for the digitally enabled community rehabilitation, or both as assigned (restricted analysis population 1). A multi-level model analysis will be used to evaluate effects of Coverscan™, digitally enabled community rehabilitation, and their interaction, on FAS at 12 weeks adjusting for baseline FAS, with gender as a fixed effect and clinic and PCN as random effects (random intercepts). To account for any systematic effects of participating in both the drug and cluster trial, an indicator

term will be added to the model. Where PCN is a random effect, the strata variables binary IMD and binary PCN patient population size will also be fitted as fixed effects.

6.3.4. Analysis for the secondary objective in the drug trial

The principal analysis population (analysis population 3 – gold rectangles in Figure 2) for drug vs. usual care comparisons within the drugs trial will be the participants randomised into the drug trial from any of the 12 centres regardless of participant involvement in the cluster trial, or whether the participant has been seen at a Long COVID clinic before. Participants randomly allocated to each drug arm will be compared to those randomly allocated to usual care (using concurrent usual care controls). The analysis will include all participants randomly allocated to a drug arm. To account for any systematic effects of participating in both the drug and cluster trial, an indicator term will be added to the model. A multi-level model for FAS, adjusting for baseline, with gender and treatment pathway as fixed effects and clinic as a random effect (random intercepts), will be applied to the drug trial data comparing each individual drug arm to the no drug arm.

6.3.5. Analysis for the secondary objective in the drug and cluster trial together

The principal analysis population (analysis population 4 – upper gold rectangle in Figure 2) for drug vs. usual care comparisons within the drugs trial will be the participants randomised into the drug trial and also involved in the cluster trial. Participants randomly allocated to each drug arm will be compared to those randomly allocated to usual care (using concurrent usual care controls). The analysis will include all cluster trial participants randomly allocated to a drug arm. A multi-level model for FAS, adjusting for baseline, with gender as a fixed effect and clinic and PCN as random effects (random intercepts), will be applied to the drug trial data comparing each drug arm to the no drug arm. Where PCN is a random effect, the strata variables binary IMD and binary PCN patient population size will also be fitted as fixed effects.

6.3.6. Proteome Statistical Analysis

Preliminary proteomics data on a subset of participants, will be used to compare with de-identified imaging metrics to investigate the link between organ impairment found by Coverscan and the proteome. In the UCLH participant cohort samples where feasible, proteomics assays will be tested for associations with clinical symptoms and laboratory abnormalities. Assay output data will be stratified by symptom duration and severity to characterise the trajectory of Long COVID.

All data will be used to design further experimental work to delineate the mechanistic basis of Long COVID and design interventions for future clinical trials. We intend to establish a large bioresource of gifted samples and data, to support future work both for the trial investigators and the wider medical community. Preliminary proteomics data on a subset of participants, will be used to compare with de-identified imaging metrics to investigate the link between organ impairment found by Coverscan and the proteome. This analysis will be conducted by Perspectum in collaboration with UCL. The analysis is described in more detail in appendix 2.

6.3.7. Investigation into the pathophysiology of Long COVID through reverse translation

In parallel, samples from UCLH participants analysed at the Francis Crick Institute as part of the exploratory endpoint will be used to generate hypotheses for further discovery research in Long COVID and to define therapeutic targets for future clinical trials. The analysis is described in more detail in appendix 3.

- High throughput proteomics

Plasma proteins will be compared across clusters stratified by fatigue severity (FAS score) using multinomial regression with sparse group lasso penalty. Potential confounders such as age, body-mass index and number of comorbidities will be included as covariates. For nonzero features, statistical significance will be defined as a univariate P value of less than 0.1, after Benjamini-Hochberg Procedure adjustment for multiple testing, with protein expression as the dependent variable. The resulting proteins will then be cross referenced against data generated using the O-LINK platform and published biomarker candidates/protein signatures for validation. If required, a further independent dataset will be identified for external validation of the findings. This analysis will be undertaken by a biostatistician at the CRICK Laboratory in accordance with an agreed statistical analysis plan (appendix 3).

6.4. Secondary outcome analysis

Secondary outcomes on an interval scale for the cluster trial will be analysed similarly to the primary outcome, adjusting for baseline outcome measure, clinic and PCN as a random effect (on analysis population 1). To account for any systematic effects of participating in both the drug and cluster trial, an indicator term will be added to the model. The principal analysis set for estimates of effects of Coverscan™, digitally enabled community rehabilitation, and their interaction, on these secondary outcomes (FAS at 24 weeks, EQ-5D-5L, GAD-7, PHQ-9, WSAS, PDQ-5, CFQ, SF-12, MRC Dyspnoea score) being participants who consented to data collection for the cluster RCT. Where appropriate, alternative methods will be applied to outcomes with skewed distributions.

Secondary outcomes on an interval scale will be compared between drug treatment arms using the same methods as for the primary outcome (analysis population 3), using a multi-level model for the relevant outcome, adjusting for baseline, with gender as a fixed effect and clinic and PCN as random effects. Patterns of missingness will be summarised. In modelling, complete case analysis will be done as the main analysis. For missing outcome data, we will use a threshold approach to imputation and sensitivity analyses.

Assumption checking in linear models will be undertaken as for the primary outcome. For outcomes not on a linear scale, equivalent checks will be made for logistic and ordinal regression models.

6.5. Sensitivity analysis

A sensitivity analysis of the effects of Coverscan™, digitally enabled community rehabilitation, and their interaction, will include only those who consent to data collection for the cluster trial. If the Living with COVID Recovery™ application is found to be beneficial, further exploratory analyses will explore factors associated with greater benefit. Some participants allocated to this application will not be able to access it as they lack a suitable mobile phone or tablet. Other participants will have suitable access but may choose to interact with it on a more or less extensive basis. Amount of access will be available as summary variables from within the application relating to registration with the application, opening of the application, and instances of engagement within 12 and 24 weeks of invitation.

If either of the ICP interventions is found to be significant (in analysis population 1 restricted to those who received their allocated treatment pathway) there will be a further analysis of the same analysis population with be the addition of data quantifying the level of the intervention. In particular, the abnormality/abnormality status of the Coverscan (overall or number of organs abnormal), and/or the degree of engagement with the digitally enabled community rehabilitation (number of engagements in the first 12 weeks). All modelling and adjustment factors will be as in 6.3.1.

The drug trial analysis (analysis population 3) of the primary outcome, FAS at 12 weeks, will be repeated restricting the drug arm participants to those who completed the full 84 days allocation of medication vs. the control (no-drug) arm participants who completed at least 84 days of follow up. This analysis will exclude participants who withdrew/were withdrawn from the drug treatment and/or follow up within 84 days. It will also exclude participants with an interrupted supply of medication. All modelling and adjustment factors will be as in 6.3.3.

6.6. Pre-specified subgroup analyses

All subgroup analyses will be viewed as exploratory. Subgroup analyses are of interest due to the possibility that there will be subgroups for whom specific treatment pathways (cluster interventions) and/or the drug treatments will be more effective. These differing effects will be identified as interactions between the subgroup indicator variable and the treatment effect.

Subgroup analyses of 3 clinical sub-phenotypes, cardiorespiratory, neuropsychiatric and "mast-cell activation type" (including rashes, joint pain and gastrointestinal disturbances and serositis symptoms) will be performed. These subgroups are not mutually exclusive as some participants may belong to more than one subgroup depending on their symptoms. Primary and secondary patient reported outcomes at 12 and 24 weeks will be considered. Only one subgroup indicator (symptoms for this sub-phenotype present or absent) will be considered within a single analysis. It is recognised that with the revised recruitment targets, the pre-specified subgroup analyses are likely to be underpowered and the focus will be on estimates of effect sizes rather than definitive comparisons.

Subgroups are defined by presence of symptoms in Table 3. Other subgroups of interest may become apparent as other research findings on patients with long COVID are published. These subgroups will be defined on a post-hoc basis and investigated as exploratory post-hoc analyses.

Baseline symptoms are collected in the patient baseline questionnaire. The clinical sub-phenotypes will be defined by symptoms being present now (within the last 4 weeks). Analyses of the cluster trial described in 6.3.1 will be repeated to investigate whether the effects of the treatment pathways differ between those participants who are/are not members of each of the subgroups separately. It is unlikely that any interactions with subgroup membership will be found statistically significant.

Analyses of the drug trial described in 6.3.4 will be repeated to investigate whether the effects of the drug treatments differ between those participants who are/are not members of each of the subgroups separately. It is unlikely that any interactions with subgroup membership will be found statistically significant.

Table 3. Definition of pre-specified subgroups using data from the baseline participant questionnaire

Q27 Answer (Mild, Moderate or Severe) to report symptoms "Now (Within the last 4 weeks)"	Cardio-respir-atory	Neuro-psych-iatric	Mast Cell Activation
1) Breathlessness at rest	x		
2) Breathlessness changing position e.g. from lying to sitting or sitting to lying	x		
3) Breathlessness on dressing yourself	x		
4) Breathlessness on walking up a flight of stairs	x		
5) Cough/throat sensitivity	x		x
6) Sore throat	x		x
7) Change of voice	x		x
8) Did you or do you get fatigued easily; Fatigue levels in your usual daily activities	x	x	x
9) Altered smell		x	
10) Altered taste		x	
11) Chest pain at rest	x		
12) Chest pain on exertion	x		
13) Chest pain after exertion	x		
14) Constant chest pain	x		
15) Chest pain on breathing in	x		
16) Chest tenderness/heaviness or feeling of chest pressure	x		
17) Joint pain			x
18) Muscle pain or aches			x
19) Headache			x
20) Abdominal pain			x
21) Problems with concentration		x	
22) Problems with memory		x	
23) Problems with planning for your daily activities		x	
24) Difficulty communicating (finding the right words or understanding others)		x	
25) Fast heartbeat, feeling of missed beats or flutter in certain positions, activity or at rest	x		x
26) Dizziness in certain positions, activity or at rest	x		x
27) Crashing or relapse hours or days after physical, cognitive, or emotional exertion		x	x
28) Duration of post-exertional symptoms Less than 12 hours (<12h) or more than 12 hours (>12h)		x	x
29) Feeling anxious		x	
30) Feeling depressed		x	
31) Having unwanted memories of your illness		x	
32) Having unpleasant dreams about your illness		x	

33) Trying to avoid thoughts or feelings about your illness		x	
34) Sleep problems, such as difficulty falling asleep, staying asleep or oversleeping		x	
Q28 patient reported yes "I had this symptom in the last 4 weeks"			
Fever			x
Night Sweats			x
Hot Flushes			x
Skin rash/discolouration of skin			x
Skin sensation (numbness/tingling/itching/nerve pain)		x	x
Numbness/change in sensation in arms or legs		x	x
Hair loss			
Cold hands or feet	x	x	
Chilblains on fingers or toes			x
Itching eyes			x
New allergy such as medication, food etc?			x
Dry eyes/redness of eyes			x
Visual changes		x	
Sneezing		x	
Tinnitus		x	
Reduced hearing			
Fainting			x
Vertigo			x
Balance problems or falls		x	x
Weakness or movement problems or coordination problems in limbs		x	
Hallucination		x	
Swelling of feet/swelling of hands	x		
Easy bruising/bleeding	x		
Waking up at night gasping for air (also called sleep apnoea)	x		
Difficulty swallowing solids		x	
Difficulty swallowing liquids		x	
Dry mouth/mouth ulcers			x
Acid Reflux/heartburn			
Change in appetite			
Nausea			x
Unintentional weight loss			
Unintentional weight gain			
Constipation, diarrhoea, or bowel incontinence		x	
Bladder frequency, urgency, or incontinence		x	
Change in menstrual cycles or flow			
Reduced interest in sex			

6.7. Interim analysis

Plans for the interim analyses have been dropped given the revision of the numbers expected to be recruited into the cluster and drug trials.

Given the changes in the design to partially decouple the cluster and drug trials and the revision of the analysis populations for both trials, no interim analyses will be completed for the cluster trial or the drug trial. The final analysis at the end of the cluster trial will be at the conventional 0.05 two-sided level. There are practical and operational reasons why a single pathway cannot graduate and become the standard pathway within the recruitment period of this trial.

The interim analyses will occur based on numbers of participants recruited into the drug trial rather than the cluster trial. Given the lower-than-expected recruitment, no interim analysis is expected before the end of recruitment of participants.

The final analysis for each drug will be with at least 200 participants in the active arm and 200 concurrent controls, and will use the conventional 2-sided 0.05 level. This analysis has 85% power if Cohen's D is 0.3 and 97% power if Cohen's D is 0.4.

6.8. Missing data

Patterns of missingness will be summarised. Complete case analysis will be the main analysis strategy.

For FAS recorded at any time point, a single missing item will be replaced to allow a score to be calculated following the standard recommended methods for PHD-9 and GAD-7. After reversing the two FAS items that require this, the missing item should be replaced by the average of the other 9 FAS items (effectively scoring as usual and multiplying by 10/9) to allow a FAS score to be calculated. It is acknowledged that this rule might have the potential to reduce the standard deviation (SD), to an "optimistic" SD, from what it would have been if only those with 10 items were included. However, simulations mode by dropping a single item of baseline FAS data and applying the rule to obtain a FAS score did not change the SD by a factor more extreme than 0.99 to 1.05 (-1% to +5%).

As a sensitivity analysis, we will use a threshold approach for missing primary outcome data (completely missing and fewer than 9 FAS items completed). Imputation based on thresholds will be implemented such that missing values will be replaced by values from the lower and upper quantiles of the distribution, e.g. 90th quantile, 75th quartile, 25th quartile, 10th quantile, and minimum. This is to allow for the possibility that there is informative dropout of those that are the most or least fatigued as measured by FAS.

Reasons for missingness of primary outcome with be reported considering the following categories:

- Incompleteness of the FAS (including incompleteness of particular items);
- Withdrawal;
- Death

Missing data in the outcome FAS at 24 weeks will be addressed as at 12 weeks. Other patient reported secondary outcomes with missing items will be addressed using the recommended methods for missing items for that scale, for example PHQ-9 and GAD-7 recommend the average of other items is used as the imputed value for 1-2 missing items. If no methods are proposed for missing items or the outcome is being treated as missing due to complete absence or because there are too many missing items, then a threshold approach could be adopted.

If missingness of FAS at 12 weeks exceeds 10% then there will be a greater concern about bias and a multiple imputation analysis will be undertaken as an additional sensitivity analysis, in addition to the complete case analysis and the threshold approach proposed above.

For multiple imputation for FAS, missing at random will be assumed. 50 datasets will be imputed for each modelling scenario. Random seeds will be set to enhance reproduceable results. 10,000 iterations will be the default with 1000 iterations burn in. Following an estimation step for the model of the relevant outcome as described in 6.3, estimates will be pooled following Rubin's rules. Following the assumptions of Rubin's rules, the multiple imputation model must be at least as complex as the estimation model. The imputation model will include FAS at baseline, 12 weeks and 24 weeks including randomisation variables site, birth gender, treatment pathway, drug treatment arm and variables known to be influential for missingness such as age and the indicator of participation in both cluster and drug trials. Where a covariance matrix structure is required, it will be specified as unstructured.

For the cluster trial analysis population 1, the multiple imputation model will be as above using FAS at all 3 time points, randomisation variables site, PCN, binary deprivation of the PCN, binary size of the PCN, treatment pathway (app and scan, app, scan, neither), drug treatment arm (no drug for those not in the drug trial) and variables known or anticipated to be associated with missingness: age, gender and the indicator of participation in both cluster and drug trials.

For the cluster trial analysis population 2, in those participants allocated to receive no drug (usual care arm in the drug trial or refused/ineligible for the drug trial), the multiple imputation model will be as above for analysis population 1.

For the drug trial analysis population 3, the multiple imputation model will use FAS at all 3 time points, randomisation variables site, treatment pathway (including whether or not it is a returning participant), drug treatment arm, age, gender and the indicator of participation in both cluster and drug trials.

For the drug trial analysis population 4, in those participants also on the cluster trial, the multiple imputation model will use FAS at all 3 time points, randomisation variables site, PCN, binary deprivation of the PCN, binary size of the PCN, treatment pathway (app and scan, app, scan, neither), drug treatment arm, and variables known or anticipated to be associated with missingness: age and gender.

6.9. Safety data

6.9.1. Recording and reporting of adverse events

Collection, recording and reporting of adverse events to the sponsor will be completed according to the sponsor's SOP for the Recording, Management and Reporting of Adverse Events by Investigators (JRO/INV/S05). Full definitions and criteria for categorising and defining type of adverse events are given in section 11 of the protocol.

All adverse events (AEs) will be recorded in the participant's medical records in the first instance, regardless of the allocation of the participant in the nested drug trial or the cluster trial. Adverse events will be recorded with clinical symptoms and accompanied with a simple, brief description of the event, including dates as appropriate.

In this trial, all AEs potentially related to the treatment will be recorded for both cluster interventions and the drug interventions (see definitions below).

6.9.2. Assessing adverse events and their seriousness

AEs for participants on the data collection arm do not need to be recorded in the eCRF.

For participants enrolled in the nested drug trial all AEs will be recorded in the eCRF following consent. For participants randomised to standard of care drugs only, AEs will be recorded until 12 weeks post enrolment. For participants randomised to receive an IMP, AEs will be recorded until 28 days following last dose of an IMP. Where possible, a diagnosis rather than a list of symptoms should be recorded. If a diagnosis has not been made, then each symptom should be listed individually. All AEs should be captured on the appropriate AE pages in the eCRF.

The assessment of relationship of adverse events to the administration of IMP must be made by the investigator (or delegated medically qualified person). It is based on clinical judgement using all available information at the time of the completion of the CRF.

6.9.3. Assessing Serious Adverse Events and Serious Adverse Reactions (nested drug trial participants only)

All Serious Adverse Reactions (SAEs) occurring from the time of written informed consent until 28 days after the last dose of the IMP, or the end of their assessments at 12 Weeks for participants on standard of care drugs only, must be recorded in the medical records, the eCRF and the trial specific SAE Reporting Form and reported to the Sponsor within 24 hours of the research staff becoming aware of the event. The Investigator or designated individual will complete the Sponsor's trial specific SAE Reporting Form and email it to the Sponsor at SAE@ucl.ac.uk and LCTUSTIMULATE@uclan.ac.uk. The Investigator will respond to any SAE queries raised by the Sponsor as soon as possible.

Any change of condition or other follow-up information should be emailed to the Sponsor, on an SAE Reporting Form (clearly marked as follow-up) as soon as it is available or at least within 24 hours of the information becoming available.
Events will be followed up until the event has resolved or a final outcome has been reached. SAE follow-up should continue after completion of protocol treatment and/or trial follow-up if necessary. Any SAR will need to be reported to the Sponsor, irrespective of how long after IMP administration the reaction has occurred until resolved.

6.9.4. Serious Adverse Events which do not require reporting to Sponsor

SAEs for participants on the data collection only arm of the trial do not need recording in the eCRF or reporting to Sponsor with the exception of the SAEs which are suggestive of suicidal ideation following review of participant completed questionnaire which will be recorded and reported to the Sponsor. For participants enrolled in the nested drug trial, the following events do not require immediate reporting to the sponsor as SAEs, however they will still be recorded in the participant's medical records. Hospitalisation for: • Routine treatment or monitoring of the studied indication not associated with any deterioration in condition. • Any admission to hospital or other institution for general care where there was no deterioration in condition. • Treatment on an emergency, outpatient basis for an event not fulfilling any of the definitions of serious as given above and not resulting in hospital admission.

6.9.5. SUSAR reporting

All SAEs assigned by the PI or delegate as suspected to be related to IMP-treatment (SARs) will be assessed for expectedness against the current approved RSI for the trial by the Sponsor.

In this trial, all AEs potentially related to the treatment will be recorded for both cluster interventions and the drug interventions (see definitions below).

Suspected serious adverse reactions (SSARs) and suspected unexpected serious adverse reactions (SUSARs) will be listed by trial allocation.
For each of the following, counts and percentages will be presented by randomised group. Where possible, the absolute risk differences will also be presented with confidence Intervals

6.10. Statistical software

Statistical analyses will be undertaken using statistical package Stata version 17 or a later version.

7. REFERENCES

1. C1248-national-guidance-post-covid-syndrome-assessment-clinics-v2.pdf.
2. Campbell H et al. Integrated care pathways. BMJ. 1998.pdf.
3. Office of National Statistics. <Prevalence of ongoing symptoms following coronavirus (COVID-19) infection in the UK 5 August 2021.pdf>. 2021.
4. Al-Aly Z, Xie Y, Bowe B. High-dimensional characterization of post-acute sequelae of COVID-19. Nature. 2021;594(7862):259-64.
5. Mandal S, Barnett J, Brill SE, Brown JS, Denneny EK, Hare SS, et al. 'Long-COVID': a cross-sectional study of persisting symptoms, biomarker and imaging abnormalities following hospitalisation for COVID-19. Thorax. 2021;76(4):396-8.
6. Maxwell E. Living-with-Covid-Themed-Review-October-2020.pdf. 2020.
7. R H. <Offline COVID-19 is not a pandemic Lancet.pdf>. Lancet. 2020;396(874).
8. Banerjee A, Pasea L, Harris S, Gonzalez-Izquierdo A, Torralbo A, Shallcross L, et al. Estimating excess 1-year mortality associated with the COVID-19 pandemic according to underlying conditions and age: a population-based cohort study. The Lancet. 2020;395(10238):1715-25.
9. Greenhalgh T, Knight M, A'Court C, Buxton M, Husain L. Management of post-acute covid-19 in primary care. BMJ. 2020;370:m3026.
10. NICE. <covid19-rapid-guideline-managing-covid19>. 2021.
11. Jacobson TA, Smith LE, Hirschhorn LR, Huffman MD. Using implementation science to mitigate worsening health inequities in the United States during the COVID-19 pandemic. Int J Equity Health. 2020;19(1):170.
12. Glasgow. <The RE-AIM framework for evaluating interventions what canit tell us about approaches to chronic illness management.pdf>. 2001.
13. McNulty M, Smith JD, Villamar J, Burnett-Zeigler I, Vermeer W, Benbow N, et al. Implementation Research Methodologies for Achieving Scientific Equity and Health Equity. Ethn Dis. 2019;29(Suppl 1):83-92.
14. Moore GF, Audrey S, Barker M, Bond L, Bonell C, Hardeman W, et al. Process evaluation of complex interventions: Medical Research Council guidance. BMJ. 2015;350:h1258.
15. Dennis A, Wamil M, Alberts J, Oben J, Cuthbertson DJ, Wootton D, et al. Multiorgan impairment in low-risk individuals with post-COVID-19 syndrome: a prospective, community-based study. BMJ Open. 2021;11(3):e048391.
16. Heightman M, Prashar J, Hillman TE, Marks M, Livingston R, Ridsdale H, et al. Post-COVID assessment in a specialist clinical service: a 12-month, single-centre analysis of symptoms and healthcare needs in 1325 individuals. medRxiv. 2021:2021.05.25.21257730.
17. Carson G. Research priorities for Long Covid: refined through an international multi-stakeholder forum. BMC Med. 2021;19(1):84.
18. Mandal S, Barnett J, Brill SE, Brown JS, Denneny EK, Hare SS, et al. 'Long-COVID': a cross-sectional study of persisting symptoms, biomarker and imaging abnormalities following hospitalisation for COVID-19. Thorax. 2021;76(4):396-8.
19. Society R. Long Covid what is it and what is needed.pdf.
20. Ayoubkhani D, Khunti K, Nafilyan V, Maddox T, Humberstone B, Diamond I, et al. Post-covid syndrome in individuals admitted to hospital with covid-19: retrospective cohort study. BMJ. 2021;372:n693.

Docusign Envelope ID: 694B9EEA-7D5D-4DDE-8CD2-98FA170FF184

21. Davis HE, Assaf GS, McCorkell L, Wei H, Low RJ, Re'em Y, et al. Characterizing long COVID in an international cohort: 7 months of symptoms and their impact. EClinicalMedicine. 2021:101019.
22. Noor NM, Pett SL, Esmail H, Crook AM, Vale CL, Sydes MR, et al. Adaptive platform trials using multi-arm, multi-stage protocols: getting fast answers in pandemic settings. F1000Res. 2020;9:1109.
23. Parmar MKB, Carpenter J, Sydes MR. More multiarm randomised trials of superiority are needed. The Lancet. 2014;384(9940):283-4.
24. Parmar MK, Barthel FM, Sydes M, Langley R, Kaplan R, Eisenhauer E, et al. Speeding up the evaluation of new agents in cancer. J Natl Cancer Inst. 2008;100(17):1204-14.
25. Parmar MK, Sydes MR, Cafferty FH, Choodari-Oskooei B, Langley RE, Brown L, et al. Testing many treatments within a single protocol over 10 years at MRC Clinical Trials Unit at UCL: Multi-arm, multi-stage platform, umbrella and basket protocols. Clin Trials. 2017;14(5):451-61.
26. https:\\www.yourcovidrecovery.nhs.uk (accessed 07/04/2025)
27. de Kleijn WP, De Vries J, Wijnen PA, Drent M. Minimal (clinically) important differences for the Fatigue Assessment Scale in sarcoidosis. Respir Med. 2011;105(9):1388-95.
28. Davis HE, Assaf GS, McCorkell L, Wei H, Low RJ, Re'em Y, et al. Characterizing Long COVID in an International Cohort: 7 Months of Symptoms and Their Impact. 2021.
29. Hopewell S, Chan AW, Collins GS, Hróbjartsson A, Moher D, Schulz KF, Tunn R, Aggarwal R, Berkwits M, Berlin JA, Bhandari N, Butcher NJ, Campbell MK, Chidebe RCW, Elbourne D, Farmer A, Fergusson DA, Golub RM, Goodman SN, Hoffmann TC, Ioannidis JPA, Kahan BC, Knowles RL, Lamb SE, Lewis S, Loder E, Offringa M, Ravaud P, Richards DP, Rockhold FW, Schriger DL, Siegried NL, Staniszewska S, Taylor RS, Thabane L, Torgerson D, Vohra S, White IR, Boutron I. CONSORT 2025 statement: updated guideline for reporting randomised trials. BMJ 2025;389:e081123.
30. Campbell MK, Piaggio G, Elbourne DR, Altman DG for the CONSORT Group. Consort 2010 statement: extension to cluster randomised trials. BMJ 2012;345:e5661
31. Kahan BC, Hall SS, Beller EM, Birchenall M, Chan A-W, Elbourne D, Little P, Fletcher J, Golub RM, Goulao B, Hopewell S, Islam N, Zwarenstein M, Juszczak E, Montgomery AA. Reporting of Factorial Randomized Trials Extension of the CONSORT 2010 Statement. JAMA. 2023;330(21):2106–2114.
32. Dimairo M, Pallmann P, Wason J, Todd S, Jaki T, Julious SA, Mander AP, Weir CJ, Koenig F, Walton MK, Nicholl JP, Coates E, Biggs K, Hamasaki T, Proschan MA, Scott JA, Ando Y, Hind D, Altman DG on behalf of the ACE Consensus Group. The Adaptive designs CONSORT Extension (ACE) statement: a checklist with explanation and elaboration guideline for reporting randomised trials that use an adaptive design. BMJ 2020;369:m115.
33. The National Kidney Federation, https://www.kidney.org.uk/chronic-kidney-disease (accessed 07/04/2025)

STIMULATE SAP APPENDIX: Creation and Transformation Variables for Secondary Outcomes V0.10 04-03-2025

1. Fatigue Assessment Scale (FAS) at 24 weeks

Description:

The FAS is a 10-item scale evaluating symptoms of chronic fatigue. The FAS treats fatigue as a uni-dimensional construct and does not separate its measurement into different factors. Each item of the FAS is answered using a five-point, Likert-type scale ranging from 1 (“Never”) to 5 (“Always”) Additional guidance is given for categories 2. Sometimes (about monthly or less), 3. Regularly (about a few times a month), 4. Often (about weekly), and 5. Always (about every day). Items 4 and 10 are reverse scored. Total scores can range from 10, indicating the lowest level of fatigue, to 50, denoting the highest. Scores on questions 4 and 10 should be recoded (1=5, 2=4, 3=3, 4=2, 5=1). Subsequently, the total FAS score can be calculated by summing the scores on all questions (recoded scores for questions 4 and 10).

Questions Comprising Measure Are:

Q1. I am bothered by fatigue
Q2. I get tired very quickly
Q3. I don’t do much during the day
Q4. I have enough energy for everyday life
Q5. Physically, I feel exhausted
Q6. I have problems to start things
Q7. I have problems to think clearly
Q8. I feel no desire to do anything
Q9. Mentally, I feel exhausted
Q10. When I am doing something, I can concentrate quite well”

Algorithm to be used to calculate “FAS Total score”:

Q4b = (-1(Q4) + 6)
Q10b = (-1(Q10) + 6)

Missing Items

“Number Missing” = 0

For `var’ of Q1, Q2, Q3, Q4b, Q5, Q6, Q7, Q8, Q9, Q10b

    If `var’ = missing
        “Number Missing” = “Number Missing” +1
        `var’ = 0

FAS Total score

If “Number Missing” > 1
    “FAS Total score” = Missing

Else

$$\text{“FAS Total score”} = \frac{(Q1 + Q2 + Q3 + Q4b + Q5 + Q6 + Q7 + Q8 + Q9 + Q10b) \times 10}{(10 - \text{“Missing”})}$$

2. Health related quality of life (EQ-5D-5L)

Description:

The EQ-5D-5L is a 6-item measure to assess quality of life comprising 5 items scored 1-5 with 1 indicating No problem and 5 an extreme problem in the areas of “Mobility”, “Self-care”, “Activity”, “Pain”, and “Anxiety”. The 6th item is a Visual Analogue Scale (VAS) assessing the takers perceived current health with a minimum score of 0 indicating “The worst health you can imagine” and a maximum score of 100 indicating “The best health you can imagine”.

The EQ-5D-5L requires no transformation or calculation.

3. Mental health- Generalised Anxiety Disorder Questionnaire (GAD-7)

Description:

The GAD-7 is a 7-item measure that assesses anxiety; each item is has responses “Not at all”, “Several days”, “More than half the days”, and “Nearly every day” with assigned scores of 0, 1 , 2, and 3 respectively. A minimum total score of 0 indicating “No Anxiety” maximum total score of 21 indicating “Severe anxiety” can be obtained.

Questions Comprising Measure Are:

Q1) Feeling nervous, anxious or on edge?
Q2) Not being able to stop or control worrying?
Q3) Worrying too much about different things?
Q4) Trouble relaxing?
Q5) Being so restless that it is hard to sit still?
Q6) Becoming easily annoyed or irritable?
Q7) Feeling afraid as if something awful might happen?

Algorithm to be used to calculate “GAD Total score”:

Missing Items

“Number Missing” = 0

For `var’ of Q1, Q2, Q3, Q4, Q5, Q6, Q7

If `var’ = missing
“Number Missing” = “Number Missing” +1
`var’ = 0
else `var’ = `var’ – 1 [Note in database coded as 1-4 instead of 0-3]

GAD Total score

If “Number Missing” > 2
“GAD Total score” = Missing

Else

$$\text{“GAD Total score”} = \frac{(Q1 + Q2 + Q3 + Q4 + Q5 + Q6 + Q7) \times 7}{(7 - \text{“Missing”})}$$

4. Depression (PHQ-9)

Description:

The PHQ-9 is a 9-item measure that assesses depression; each item has responses “Not at all”, “Several days”, “More than half the days”, and “Nearly every day” with assigned scores of 0, 1, 2, and 3 respectively. A minimum total score of 0 indicating “No to minimal depression” and a maximum total score of 27 indicating “Severe depression” can be obtained.

Questions Comprising Measure Are:

Q1) Little interest or pleasure in doing things?
Q2) Feeling down, depressed, or hopeless?
Q3) Trouble falling or staying asleep, or sleeping too much?
Q4) Feeling tired or having little energy?
Q5) Poor appetite or overeating?
Q6) Feeling bad about yourself - or that you are a failure or have let yourself or your family down?
Q7) Trouble concentrating on things, such as reading the newspaper or watching television?
Q8) Moving or speaking so slowly that other people could have noticed? Or the opposite - being so fidgety or restless that you have been moving around a lot more than usual?
Q9) Thoughts that you would be better off dead, or of hurting yourself in some way?

Algorithm to be used to calculate “PHQ Total score”:

Missing Items

“Number Missing” = 0

For `var’ of Q1, Q2, Q3, Q4, Q5, Q6, Q7, Q8, Q9

    If `var’ = missing
        “Number Missing” = “Number Missing” +1
        `var’ = 0

    else `var’ = `var’ – 1 [Note in database coded as 1-4 instead of 0-3]

PHQ Total score

If “Number Missing” > 2
    “PHQ Total score” = Missing

Else
    “PHQ Total score” = $\frac{(Q1 + Q2 + Q3 + Q4 + Q5 + Q6 + Q7 + Q8 + Q9) \times 9}{(9 - \text{“Missing”})}$

5. Medical Research Council Dyspnoea Score (MRCDS)

Description:

It is a questionnaire in which patient has to understand the activity when he feels dyspnoea and based on that patient has to grade the dyspnoea. The MRC breathlessness scale comprises five statements that describe almost the entire range of respiratory disability from none (Grade 0)

to almost complete incapacity (Grade 4). It can be self-administered by asking subjects to choose the statement that best describes their condition, e.g. 'I only get breathless with strenuous exertion' (Grade 0) or 'I am too breathless to leave the house' (Grade 4). Alternatively, it can be administered by an interviewer with the statements framed as questions, e.g. 'Are you short of breath when hurrying on the level or walking up a slight incline' (Grade 1). The score is the number that best fits to the patient's level of activity. All the questions relate to everyday activities and are generally easily understood by patients. A score can usually be obtained in a few seconds.

Statements Comprising Measure Are:

0- I only get breathless with strenuous exercise
1- I get short of breath when hurrying on level ground or walking up a slight hill
2- On level ground, I walk slower than people of my age because of breathlessness, or I have to stop for breath when walking at my own pace on the level
3- I stop for breath after walking about 100 yards or after a few minutes on level ground
4- I am too breathless to leave the house or I am breathless when dressing/undressing

Algorithm:

MRCDS = MRCDS – 1 [Note in database coded as 1-5 instead of 0-4]

6. Perceived Deficit Questionnaire (PDQ-5)

Description:

The Perceived Deficits Questionnaire – Depression (PDQ-5) is a brief patient-rated scale to assess subjective cognitive dysfunction in people with depression. The PDQ-5 is a 5-item questionnaire that generates a total score. Each question has 5 levels, "Never", "Rarely", "Sometimes", "Often", and "Very often" with assigned scores of 0, 1, 2, 3, and 4 respectively. A total score is obtained with values in the range of 0 -20, higher scores indicate more cognitive dysfunction.

Questions Comprising the Measure Are:

Q1 - Have trouble getting things organised?
Q2 - Have trouble concentrating on things like watching a television programme or reading a book?
Q3 - Forget the date unless you looked it up?
Q4 - Forget what you talked about after a telephone conversation?
Q5 - Feel like your mind went totally blank?

Algorithm to be used to calculate "PDQ Total score":

PDQ_Score = Q1 + Q2 + Q3 + Q4 + Q5 [Note all items must be present to calculate score]

PDQ_Score = PDQ_Score – 5 [Note in database coded as 1-5 instead of 0-4]

7. Work and Social Adjustment Scale (WSAS) [Question 4 from Productivity Cost Questionnaire (iPCQ) for absenteeism and Question 8 from iPCQ for presenteeism added]

WSAS Work and Social Adjustment Scale

Description:

The Work and Social Adjustment Scale (WSAS) assesses how much Long COVID impairs abilities and activities. The minimum score for this test is 0 and maximum score is 40 with higher scores indicating higher impairment. Five questions scored from 0-8 guidance make up this scale and additional guidance is given for scores 0, 2, 4, 6, 8 of “Not at all”, “Slightly”, “Definitely”, “Markedly”, and “Very Severely” respectively. These questions concern ability to work, home management, leisure activities (social and private) and close relationships.

Questions Comprising Measure Are:

Q1- Because of my problem my ability to work is impaired.
Q2 - Because of my problem my home management (cleaning, tidying, shopping, cooking, looking after home or children, paying bills) is impaired
Q3 - Because of my problem my social leisure activities (with other people e.g. parties, bars, clubs, outings, visits, dating, home entertaining) are impaired
Q4 - Because of my problem my private leisure activities (done alone, such as reading, gardening, collecting, sewing, walking alone) are impaired
Q5 - Because of my problem my ability to form and maintain close relationships with others, including those I live with, is impaired

Algorithm to be used to calculate “WSAS Total score”:

WSAS_Score = Q1 + Q2 + Q3 + Q4 + Q5 [Note all items must be present to calculate score]

WSAS_Score = WSAS_Score – 5 [Note in database coded as 1-9 instead of 0-8]

iPCQ [Question 4 from Productivity Cost Questionnaire (iPCQ) for absenteeism and Question 8 from iPCQ for presenteeism added]

Description:

Used to assess the number of working days missed due to problems caused by the illness.

No Score Calculated

8. Short Form Questionnaire (SF-12)

Description:

The SF-12 was constructed using questions drawn from each of the 8 dimensions of the MOS 36 item Short Form Survey (SF-36). It is designed to have similar performance to the SF-36, while taking less time to complete. Two summary scores are reported from the SF-12 – a mental component score (MCS-12) and a physical component score (PCS-12).

Questions Comprising Measure Are:

Q1 - In general, would you say your health is:

Value – Response
1 – “Excellent”

2 – “Very Good”
3 – “Good”
4 – “Fair”
5 – “Poor”

Q2 - Moderate activities such as moving a table, pushing a vacuum cleaner, bowling, or playing golf

Value – Response
1 – “Limited a lot”
2 – “Limited a little”
3 – “Not limited at all”

Q3 - Climbing several flights of stairs

Value - Response
1 – “Limited a lot”
2 – “Limited a little”
3 – “Not limited at all”

Q4 - Accomplished less than you would like

Value – Response
1 – “Yes”
2 – “No”

Q5 - Were limited in the kind of work or other activities

Value – Response
1 – “Yes”
2 – “No”

Q6 - Accomplished less than you would like

Value – Response
1 – “Yes”
2 – “No”

Q7 - Did work or activities less carefully than usual

Value – Response
1 – “Yes”
2 – “No”

Q8 - During the past 4 weeks, how much did pain interfere with your normal work (including work outside the home and housework)?

Value - Response
1 – “Not at all”
2 – “A Little Bit”
3 – “Moderately”
4 – “Quite a bit”
5 – “Extremely”

Q9 - Have you felt calm and peaceful?

Value – Response
1 – “All of the time”
2 – “Most of the time”
3 – “A good bit of the time”
4 – “Some of the time”
5 – “A little of the time”

6 – “None of the time”

Q10 - Did you have a lot of energy?

Value - Response

1 – “All of the time”

2 – “Most of the time”

3 – “A good bit of the time”

4 – “Some of the time”

5 – “A little of the time”

6 – “None of the time”

Q11 - Have you felt downhearted and blue?

Value - Response

1 – “All of the time”

2 – “Most of the time”

3 – “A good bit of the time”

4 – “Some of the time”

5 – “A little of the time”

6 – “None of the time”

Q12 - During the past 4 weeks, how much of the time has your physical health or emotional problems interfered with your social activities (like visiting friend, relatives, etc.)?

Value - Response

1 – “All of the time”

2 – “Most of the time”

3 – “Some of the time”

4 – “A little of the time”

5 – “None of the time”

Algorithm to be used to calculate “PCS-12” & “MCS-12”:

Generation of Temporary variables

if Q2 = 1, PF02_1 = 1, else PF02_1 = 0
if Q2 = 2, PF02_2 = 1, else PF02_2 = 0
if Q3 = 1, PF04_1 = 1, else PF04_1 = 0
if Q3 = 2, PF04_2 = 1, else PF04_2 = 0
if Q4 = 1, RP2_1 = 1, else RP2_1 = 0
if Q5 = 1, RP3_1 = 1, else RP3_1 = 0
if Q8 = 5, BP2_1 = 1, else BP2_1 = 0
if Q8 = 4, BP2_2 = 1, else BP2_2 = 0
if Q8 = 3, BP2_3 = 1, else BP2_3 = 0
if Q8 = 2, BP2_4 = 1, else BP2_4 = 0
if Q1 = 5, GH1_1 = 1, else GH1_1 = 0
if Q1 = 4, GH1_2 = 1, else GH1_2 = 0
if Q1 = 3, GH1_3 = 1, else GH1_3 = 0
if Q1 = 2, GH1_4 = 1, else GH1_4 = 0
if Q10 = 6, VT2_1 = 1, else VT2_1 = 0
if Q10 = 5, VT2_2 = 1, else VT2_2 = 0
if Q10 = 4, VT2_3 = 1, else VT2_3 = 0
if Q10 = 3, VT2_4 = 1, else VT2_4 = 0
if Q10 = 2, VT2_5 = 1, else VT2_5 = 0
if Q12 = 1, SF2_1 = 1, else SF2_1 = 0

if Q12 = 2, SF2_2 = 1, else SF2_2 = 0
if Q12 = 3, SF2_3 = 1, else SF2_3 = 0
if Q12 = 4, SF2_4 = 1, else SF2_4 = 0
if Q6 = 1, RE2_1 = 1, else RE2_1 = 0
if Q7 = 1, RE3_1 = 1, else RE3_1 = 0
if Q9 = 6, MH3_1 = 1, else MH3_1 = 0
if Q9 = 5, MH3_2 = 1, else MH3_2 = 0
if Q9 = 4, MH3_3 = 1, else MH3_3 = 0
if Q9 = 3, MH3_4 = 1, else MH3_4 = 0
if Q9 = 2, MH3_5 = 1, else MH3_5 = 0
if Q11 = 1, MH4_1 = 1, else MH4_1 = 0
if Q11 = 2, MH4_2 = 1, else MH4_2 = 0
if Q11 = 3, MH4_3 = 1, else MH4_3 = 0
if Q11 = 4, MH4_4 = 1, else MH4_4 = 0
if Q11 = 5, MH4_5 = 1, else MH4_5 = 0

Algorithm "PCS-12"

PCS_12 = 56.57706 + (-7.23216 x PF02_1) + (-3.45555 x PF02_2) + (-6.24397 x PF04_1) + (-2.73557 x PF04_2) + (-4.61617 x RP2_1) + (-5.51747 x RP3_1) + (-11.25544 x BP2_1) + (-8.38063 x BP2_2) + (-6.50522 x BP2_3) + (-3.8013 x BP2_4) + (-8.37399 x GH1_1) + (-5.56461 x GH1_2) + (-3.02396 x GH1_3) + (-1.31872 x GH1_4) + (-2.44706 x VT2_1) + (-2.02168 x VT2_2) + (-1.6185 x VT2_3) + (-1.14387 x VT2_4) + (-0.42251 x VT2_5) + (-0.33682 x SF2_1) + (-0.94342 x SF2_2) + (-0.18043 x SF2_3) + (0.11038 x SF2_4) + (3.04365 x RE2_1) + (2.32091 x RE3_1) + (3.46638 x MH3_1) + (2.90426 x MH3_2) + (2.37241 x MH3_3) + (1.36689 x MH3_4) + (0.66514 x MH3_5) + (4.61446 x MH4_1) + (3.41593 x MH4_2) + (2.34247 x MH4_3) + (1.28044 x MH4_4) + (0.41188 x MH4_5)

if (Q1 = Missing, or Q2 = Missing, or Q3 = Missing, or Q4 = Missing, or Q5 = Missing, or Q6 = Missing, or Q7 = Missing, or Q8 = Missing, or Q9 = Missing, or Q10 = Missing, or Q11 = Missing, or Q12 = Missing), PCS_12 = Missing

Algorithm "MCS-12"

MCS_12 = 60.75781 + (3.93115 x PF02_1) + (1.8684 x PF02_2) + (2.68282 x PF04_1) + (1.43103 x PF04_2) + (1.4406 x RP2_1) + (1.66968 x RP3_1) + (1.48619 x BP2_1) + (1.76691 x BP2_2) + (1.49384 x BP2_3) + (0.90384 x BP2_4) + (-1.71175 x GH1_1) + (-0.16891 x GH1_2) + (0.03482 x GH1_3) + (-0.06064 x GH1_4) + (-6.02409 x VT2_1) + (-4.88962 x VT2_2) + (-3.29805 x VT2_3) + (-1.65178 x VT2_4) + (-0.92057 x VT2_5) + (-6.29724 x SF2_1) + (-8.26066 x SF2_2) + (-5.63286 x SF2_3) + (-3.13896 x SF2_4) + (-6.82672 x RE2_1) + (-5.69921 x RE3_1) + (-10.19085 x MH3_1) + (-7.92717 x MH3_2) + (-6.31121 x MH3_3) + (-4.09842 x MH3_4) + (-1.94949 x MH3_5) + (-16.15395 x MH4_1) + (-10.77911 x MH4_2) + (-8.09914 x MH4_3) + (-4.59055 x MH4_4) + (-1.95934 x MH4_5)

if (Q1 = Missing, or Q2 = Missing, or Q3 = Missing, or Q4 = Missing, or Q5 = Missing, or Q6 = Missing, or Q7 = Missing, or Q8 = Missing, or Q9 = Missing, or Q10 = Missing, or Q11 = Missing, or Q12 = Missing), MCS_12 = Missing

9. Cognitive Failure Questionnaire (CFQ) if a patient scores 3 or more on PDQ5

PDQ5

See 6 above

Cognitive Failures Questionnaire (CFQ)

Description:

The cognitive failures questionnaire is a 25-item questionnaire regarding everyday mistakes people make.

Items are scored on a 0-4 scale, with the total scale score calculated as a sum of the items.

There are sub-scores that can be calculated for: forgetfulness, distractibility, and false triggering.

Questions Comprising the CFQ Measures Are:

Q1 - Do you read something and find you haven't been thinking about it and must read it again?
Q2 - Do you find you forget why you went from one part of the house to the other?
Q3 - Do you fail to notice signposts on the road?
Q4 - Do you find you confuse right and left when giving directions?
Q5 - Do you bump into people?
Q6 - Do you find you forget whether you've turned off a light or a fire or locked the door?
Q7 - Do you fail to listen to people's names when you are meeting them?
Q8 - Do you say something and realize afterwards that it might be taken as insulting?
Q9 - Do you fail to hear people speaking to you when you are doing something else?
Q10 - Do you lose your temper and regret it?
Q11 - Do you leave important letters unanswered for days?
Q12 - Do you find you forget which way to turn on a road you know well but rarely use?
Q13 - Do you fail to see what you want in a supermarket (although it's there)?
Q14 - Do you find yourself suddenly wondering whether you've used a word correctly?
Q15 - Do you have trouble making up your mind?
Q16 - Do you find you forget appointments?
Q17 - Do you forget where you put something like a newspaper or a book?
Q18 - Do you find you accidentally throw away the thing you want and keep what you meant to throw away -- as in the example of throwing away the matchbox and putting the used match in your pocket?
Q19 - Do you daydream when you ought to be listening to something?
Q20 - Do you find you forget people's names?
Q21 - Do you start doing one thing at home and get distracted into doing something else (unintentionally)?
Q22 - Do you find you can't quite remember something although it's "on the tip of your tongue"?
Q23 - Do you find you forget what you came to the shops to buy?
Q24 - Do you drop things?
Q25 - Do you find you can't think of anything to say?

CFQ_Total = Q1 + Q2 + Q3 + Q4 + Q5 + Q6 + Q7 + Q8 + Q9 + Q10 + Q11 + Q12 + Q13 + Q14 + Q15 + Q16 + Q17 + Q18 + Q19 + Q20 + Q21 + Q22 + Q23 + Q24 + Q25 - 25
[Note in database coded as 1-5 instead of 0-4, if any component is missing then score is missing]
CFQ_Forgetfulness = Q1 + Q2 + Q5 + Q7 + Q17 + Q20 + Q22 + Q23 - 8
[Note in database coded as 1-5 instead of 0-4, if any component is missing then score is missing]
CFQ_Distractibility = Q8 + Q9 + Q10 + Q11 + Q14 + Q19 + Q21 + Q25 - 8
[Note in database coded as 1-5 instead of 0-4, if any component is missing then score is missing]
CFQ_False_Triggering = Q2 + Q3 + Q5 + Q6 + Q12 + Q18 + Q23 + Q24 - 8
[Note in database coded as 1-5 instead of 0-4, if any component is missing then score is missing]

10. Organ impairment and healthcare utilisation

Beyond scope of this SAP – see the Health Economic Analysis Plan (HEAP)

11. Cost-effectiveness of ICP

Beyond scope of this SAP – see the Health Economic Analysis Plan (HEAP)

12. Process outcomes for different ICP components

Beyond scope of this SAP

13. Blood investigations (e.g., genomic, proteomic, metabolomic/ lipidomic, functional T cell and live virus neutralisation assays and endocrine analyses)

Beyond scope of this SAP

14. Linked electronic health record data to monitor healthcare utilisation and outcomes

Beyond scope of this SAP

PERSPECTUM STATISTICAL ANALYSIS PLAN FOR STIMULATE-ICP TRIAL

Trial Registration Reference: IRAS 1004698

Author(s): Alessandro Fichera, Sarah Larkin

Signatures

Name: Alessandro Fichera

Signature:

Date: 12-06-2025

Name: Sarah Larkin

Signature:

Date: 03-07-2025

Reviewer(s): Professor Amitava Banerjee, Tom Davis

Signatures

Chief Investigator: Professor Amitava Banerjee

Signature:

Date: 10-06-2025

Trial Statistician: Tom Davis

Signature:

Date: 12-06-2025

Scope

This statistical analysis plan relates to specific proteomic analyses which are outlined in the "STIMULATE-ICP. A pragmatic, multi-centre, cluster randomised trial of an integrated care pathway with a nested, Phase III, open label, adaptive platform randomised drug trial in individuals with Long COVID" full protocol, version 5.0 dated 19/11/2024 section 14.11.1 Proteome Statistical Analysis.

Overview:

Perspectum Statistical Analysis Plan- V1.1 06/06/2025 – Investigating the link between organ impairment found by Coverscan and the proteome.

Preliminary proteomics data will be used to compare with de-identified imaging metrics to investigate the link between organ impairment found by Coverscan and the proteomic profile. A multi-organ reference range for all organ imaging abnormalities will be calculated (i) and participants will then be grouped according to the number (and identity) of organs impaired. The proteome of these groups will then be compared.

Required data:

De-identified imaging metrics from Coverscan

Participants in STIMULATE-ICP will be selected for proteomic analysis based on the finding of organ abnormality found in the Coverscan MRI[i]. The ID of the participant being scanned will be used by UCLAN clinical trials unit (LCTU) to create a unique de-identified ID that can be used to select the blood sample for proteomics. The key to link the samples to participant IDs will be held in LCTU and not disclosed to Perspectum.

Proteomic data from OLINK

Plasma samples from non-hospitalized individuals with long COVID will be analyzed using the Olink Explore 3072 panel. Protein abundance data are provided as measurements of NPX (Normalized Protein eXpression), which are relative values that can be compared within assays. A difference of 1 NPX between two measurements for the same protein represents an approximate doubling of the protein concentration (a difference of 2 NPX corresponds to a four-fold relative difference, etc.). A total of 3,000 circulating proteins will be quantified for each sample.

Demographic & patient reported outcome data

De-identified baseline sociodemographic data and symptom data will allow us to control for sociodemographic factors and enable the association of proteome profiles with specific long COVID symptoms that may also be indicative of organ abnormalities.

Samples:

The specific samples for proteomics will be selected from those stored in the STIMULATE-ICP biobank at Perspectum, based on organ abnormalities detected by Coverscan MRI. Proteomic profiles that correspond to particular imaging findings will then be analysed.

Endpoints:

Primary Endpoint

- Objective: To identify proteomic biomarkers associated with single organ impairment detected via CoverScan MRI.
- Definition: Mean NPX (normalised protein expression) levels of pre-specified biomarkers will be compared between participants with a single organ abnormality (heart, lungs, kidney, liver, pancreas, or spleen) and those with no detected abnormalities.

Secondary Endpoint

- Objective: To assess the association between proteomic profiles and multi-organ impairment.
- Definition: Comparison of NPX values for selected biomarkers between participants with two or more organ abnormalities and those with no abnormalities or single organ involvement.

Exploratory Endpoints

- Identification of novel proteomic signatures associated with specific organ combinations (e.g., heart + liver, lung + kidney).
- Correlation between the number of impaired organs and composite proteomic scores.
- Use the sociodemographic and symptom data to investigate whether there are symptom-organ impairment pairs: i.e. single symptoms at baseline (e.g. fatigue, chest pain, shortness of breath) from STIMULATE-ICP questionnaire that are paired with specific organ abnormalities (e.g. fatigue-liver impairment)

Sample size

This is a pilot investigation looking for differences in the proteome linked to organ abnormalities on Coverscan MRI. Protein abundance is highly variable and so the anticipated effect size is unknown. The NPX data from OLINK will be identify those proteins that are significantly up- or down-regulated (at an alpha level of 0.05) and the fold-change in abundance. Data from this pilot will inform sample size selection in any follow-up work. As controls we will use proteomics data from Perspectum's Coverscan study. This is a study of individuals recovering from COVID infection who also received a Coverscan MRI; we will select those individuals without organ abnormalities detected by Coverscan MRI as controls. We aim to analyse ~300 samples in individuals with organ impairment due to cost constraints, and we envisage that this will provide sufficient power to study single and multiple organ impairment across different organs and combinations of organs.

Handling of missing data

Only samples from individuals with available imaging metrics will be considered for proteomics analysis. Samples will be deemed unsuitable and excluded if the corresponding plasma sample is not available in the biobank, or if there is documentation of a significant delay between blood collection and sample processing that could affect data quality.

In the case of missing proteomics data due to technical failure (e.g. assay failure or data corruption), re-analysis will be conducted using the remaining plasma volume, provided sufficient sample is available. If re-analysis is not possible, the affected samples will be excluded from analyses and the extent and impact of missing data will be documented.

Statistical analysis

The following statistical methods will be used to analyse the proteomic profiles

- Analysis of Variance (ANOVA) and logistic regression. These analyses will be adjusted for demographics covariates, including sex, age, and Body Mass Index (BMI). Comparisons will be made between groups with impaired -vs- unimpaired organs as identified by Coverscan MRI, focusing on differences in mean NPX values derived from Olink assays.
- Regression analysis will be performed and summarised using, e.g. Orthogonal partial least squares (OPLS) and linear models.

- Association analysis between proteomic signature and organ abnormality (defined by Coverscan imaging metrics) and metabolic disease.
- Differences in expression of proteins between groups will be linked to biological function by conducting pathway and overrepresentation analysis e.g. using Gene Set Enrichment Analysis (GSEA) or KEGG database to identify overrepresented pathways that may underlie the biology of specific organ impairment.
    - Clustering analysis (K-means) will be used to identify clusters and patterns within the proteome data
    - Findings will be summarised and visualised using appropriate plots, e.g. volcano for association tests, principal component analysis (PCA) and boxplots.

---

[i] Example reference ranges for Coverscan MRI metrics

Organ	Measurement	Normal range
Heart	Ejection fraction (left ventricle)	> 55% - < 77%
Lung	Fractional change (deep breathing)	> 39%
Kidneys	Cortical T1	> 783ms - < 1191ms
Liver	cT1	> 630ms - < 800ms
Pancreas	T1	> 475ms - < 790ms

STIMULATE-ICP Statistical Analysis Plan

This SOP supersedes all previous versions.

Review Interval:	**Biennial**

Prepared by:

Name: David Greenwood

Job Title: **Senior Medical and Biological Data Scientist**

Sign: 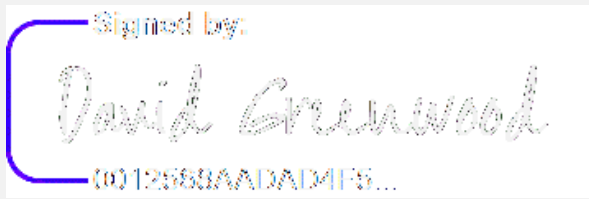

Authorised by:

Name: Emma Wall

Job Title: **Senior Clinical Research Fellow, UCLH-Crick Legacy study**
Consultant Infectious Diseases UCLH

Sign: 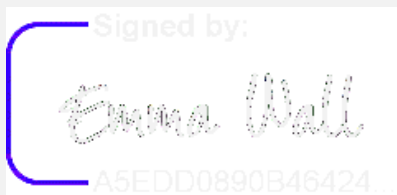

This document and all associated documents have been prepared and authorised electronically. All signatories and dates are traceable.

THIS DOCUMENT MUST BE READ AND UNDERSTOOD BY STAFF USING IT AND DOCUMENTED EVIDENCE PROVIDED THEREOF

DOCUMENT HISTORY & CHANGES

- Changes necessitate reissue of the SOP incrementing the version number
- Changes since the last version was issued are added to the changes log below. Previous changes are incorporated into the re-issued version. The changes log below details all changes made to the SOP.
- Changes to page 1 or headers and footers are not included in the changes log
- Additions to the changes log are not shown as changes.

Changes Applied to:

Version	Section	Date	Brief Description of Change
2	4 Objective 3	15/10/2024	Added SOP numbers and a description of the flow analysis pipeline.
3	2 Objective 1, 3 Objective 2, 4 Objective 3	25/10/2024	Clarified that multiple viral strains will be tested. Updated formatting. Added additional detail about the software package to be utilised.
4	3 Objective 2, 5 Objective 4	16/4/2025	The planned normalisation method did not adequately stabilise the variance, so the pre-processing steps for Objective 2 have been updated. Objective 4 has been added.
5		6/5/2025	Tracked changes cleared.
6		14/5/2024	Each objective split into current work under the STIMULATE-ICP protocol, and future work on the pathogenesis of Long COVID after the trial end as part of UCLH-Crick Legacy study IRAS 286469.

1 Introduction

1.1.1 Exploratory work at the Francis Crick Institute within STIMULATE-ICP is designed to embed discovery work on the underlying pathophysiology of Long COVID into the main trial. The objectives of the analysis are to determine if there are differences in antibody-mediated immunity (Objective 1), protein expression (Objective 2), Moreover, we aim to identify non-hospitalised Long COVID (NH-LC) endotypes based on a clustering analysis of protein expression, laboratory and symptom data (Objective 3).

1.1.2 Data will be analysed in an R coding environment and managed according to the Data Management SOP [CC073].

1.1.3 **Future work**: To extend the discovery work in Long COVID and meet urgent patient need, we will continue our work on samples from STIMULATE-ICP after the end of the Trial, in samples bio-resourced at the Crick. Analyses from objectives 1-3 will be repeated, comparing data from STIMULATE-ICP participants with matched sample and demographic data from healthy recovered adults from the UCLH-Crick Legacy study IRAS 286469 HRA approval number 20/HRA/4717. We will use these data as control samples to fully quantify the applicability and extent of any findings in Long COVID by comparison with the healthy response following infection (Objective 4). As part of future work we will further compare the T and B cellular profile, both between STIMULATE-ICP participants at baseline and after 3 months, stratified by drug allocation and between participants with Long COVID and recovered adults in Legacy.

2 Objective 1

2.1.1 Antibody-mediated immunity will be assed using the Crick's high-throughput neutralisation assay [CC032, CC033, CC034, CC035a, CC036a, CC038a, CC039, CC060a, CC062a, CC071].

2.1.2 In this study, we will conduct assays to test for neutralisation against multiple viral strains.

2.2 Non-Parametric Statistical Tests:

2.2.1 Given the non-normal distribution of the data, non-parametric tests such as the Wilcoxon signed-rank test (for two groups) or the Kruskal-Wallis test (for more than two groups) will be employed to compare the differences in antibody-mediated neutralisation between groups.

2.2.2 The significance threshold (alpha) will be set at 0.05. Results with a p-value less than 0.05 will be considered statistically significant.

2.2.3 For each viral strain, we will perform statistical tests to compare neutralisation between groups. Each viral strain's neutralisation comparison is treated as an independent hypothesis test.

2.3 Log-Fold Change:

2.3.1 The log-fold change will be calculated to quantify the change in antibody-mediated neutralisation between groups. This involves taking the logarithm base 2 of the ratio of the neutralisation levels between groups.

2.3.2 Confidence intervals for the log-fold change will be estimated by bootstrap re-sampling the data with replacement 1000 times.

2.4 Potential risks and mitigation:

2.4.1 POTENTIAL RISK: The majority of observations may fall outside the quantitative range of the neutralisation assay, either below or above the limit of detection.

2.4.2 RISK MITIGATION: In cases where the median result is below or above the limit of detection, results will be dichotomised into detectable & undetectable titres. Chi-squared tests of association will then be applied to test for significant differences in the number of quantifiable titres.

3 Objective 2

3.1.1 Protein expression will be assed using the Crick's high-throughput proteomics assay [CC078].

3.2 Proteomics data pre-processing:

3.2.1 Proteome databases will be accessed from UniProt repository: Homo sapiens, SARS-CoV-2 (FASTA files).

3.2.2 Library free deep learning method DIA-NN will be used to predict a spectral library for a given proteome database and sample. This will generate a protein group matrix quantifying relative protein abundance across each sample (.tsv file).

3.2.3 The protein group matrix will be annotated with participant metadata. Proteins and metabolites will be filtered by presence in ≥70% of samples in either group.

3.2.4 Variance stabilising normalisation (VSN) will be applied to the protein group matrix to stabilize the variance across the range of measured intensities. This ensures that the assumptions of linear regression, particularly the assumption of constant variance of the residuals, are met. Graphical assessment of normalisation will be performed by plotting rank mean expression against the standard deviation for each protein.

3.2.5 In the event that VSN fails to adequately stabilise the variance, Loess normalisation and log2 transformation will instead be applied.

3.2.6 Missing values in the protein group matrix will be imputed using the non-parametric method, missForest, with a maximum of 10 iterations.

3.3 Principal component analysis (PCA)

3.3.1 PCA will be utilised to explore the data, visualise the directions of maximum variation, and identify potential confounders such as batch effects.

3.3.2 If batch effects are detected during PCA, ComBat will be applied to adjust for these effects in the PCA plots. ComBat is a method to adjust for batch effects using an empirical Bayes framework. This adjustment helps to ensure that the observed variations in the PCA plots are due to biological differences rather than technical artifacts.

3.4 Fixed-Effect Linear Regression Models

3.4.1 The fixed-effect linear regression model will be specified to account for the fixed effects of the independent variables (age, sex, batch, study_name) on the dependent variable (concentration).

3.4.2 The parameters of the fixed-effect model will be estimated using the ordinary least squares (OLS) method.

3.4.3 The regression analysis will be conducted separately for each protein, treating each protein as a distinct dependent variable.

3.5 Likelihood Ratio Tests

3.5.1 Likelihood ratio tests will be used to compare nested models and assess the significance of the fixed effects.

3.5.1.1 Model 1 (Reduced Model): concentration~batch+sex+age

3.5.1.2 Model 2 (Full Model): concentration~batch+sex+age+study_name

3.5.2 A significant likelihood ratio test indicates that the full model, which includes the additional variable (study_name), provides a significantly better fit to the data than the reduced model.

3.5.3 The significance threshold (alpha) will be set at 0.05. Results with a p-value less than 0.05 will be considered statistically significant.

3.5.4 To account for multiple comparisons, the Benjamini-Hochberg (BH) procedure will be applied to control the false discovery rate (FDR).

3.5.5 The log-2-fold change will be calculated to quantify the relative change in concentration levels between groups.

3.6 Potential risks and mitigation:

3.6.1 POTENTIAL RISK: the quality of the proteomics data may be heterogenous between samples.

3.6.2 RISK MITIGATION: Quality control reports will be generated for each sample. The quantities of proteins, precursors, and unique proteins will be compared to ensure homogeneity across the sample set. Samples identified as outliers will be excluded from downstream analysis to maintain data integrity.

3.6.3 POTENTIAL RISK: The effect sizes of individual proteins may be insufficient to achieve statistical significance due to the increased risk of type I errors associated with multiple testing.

3.6.4 RISK MITIGATION: If no statistically significant effects are identified, agglomerative hierarchical clustering will be employed to identify functional subgroups of proteins. These subgroups will form a reduced set of variables for inclusion in the model fitting process, enhancing the power to detect significant effects by focusing on biologically relevant clusters.

4 Objective 3

4.1.1 Laboratory observations and symptom reports will be collected by the clinical trials unit during baseline participant study visits. This data will be integrated with the protein expression data described in Sections 3.1.1 and 3.2.

4.2 Pre-processing

4.2.1 Laboratory observations recorded in ≥50% of participants will be retained.

4.2.2 For each laboratory observation, we will use histograms and Q-Q plots to assess normality. If the raw data are normal, they will be analysed as such. Otherwise, a $\log_2$ transformation will be applied; if the $\log_2$-transformed data then appear normal, they will be used for analysis, and if not, rank normalisation will be applied.

4.2.3 Symptom reports comprised of dichotomous and ordinal categorical variables will be one-hot encoded.

4.3 Integration

4.3.1 We will integrate multiple data modalities using Multi-Omics Factor Analysis (MOFA), a framework which generates a low-dimensional latent space which captures global variability across each modality present in the data: continuous and binary indicator variables.

4.4 Clustering

4.4.1 Primary cluster analysis and method selection will be conducted using diceR on the latent space. This approach generates diverse cluster configurations using multiple algorithms (e.g., k-means, hierarchical clustering, dbscan), cluster sizes, and subsets of the input data. The resulting clusters are then combined into a single consensus and internal validation indices are calculated. The number of clusters to be tested corresponds to the number of NH-LC endotypes reported in the literature which ranges between 2 and 5 subgroups.

4.5 Internal validation

4.5.1 We will assess clustering significance via permutation testing, deeming clusters statistically significant if the observed silhouette index exceeds the 95th percentile of the null distribution ($P < 0.05$, one tailed).

4.5.2 We will evaluate if the clusters represent putative NH-LC endotypes by comparison with symptom profiles as defined for the UK PHOSP-COVID study. Fixed-effect logistic regression will be used to test for associations between symptom profiles and cluster allocation.

4.6 Potential risks and mitigation:

4.6.1 POTENTIAL RISK: One potential risk of one-hot encoding ordinal variables is the loss of meaningful ordering, which could obscure the identification of relevant endotypes.

4.6.2 RISK MITIGATION: To mitigate this, a sensitivity analysis will be conducted in which ordinal variables are recoded into binary groups (e.g., "absence" versus "presence" or "absent/mild" versus "moderate/severe") to assess how the different encoding strategies impact the study outcomes.

4.6.3 POTENTIAL RISK: A risk of applying MOFA is that its linear framework may fail to capture non-linear relationships among the input variables, potentially overlooking complex interactions relevant to identifying endotypes.

4.6.4 RISK MITIGATION: As a mitigation strategy, we will explore non-linear data integration techniques, including kernel-based methods (e.g., kernel PCA, kernel CCA) and deep learning approaches (e.g., autoencoders, variational autoencoders), to assess and potentially enhance the robustness of our findings.

Objective 4: Future work under UCLH-Crick Legacy study IRAS 286469

5.1 Cellular profiling

5.1.1 Analysis of cellular populations will be conducted by Flow Cytometry [CC075].

4.6.5 Cytometry datasets will be gated to live single cells using FlowJo and exported as an FCS file per sample. FCS files will be imported into R using flowCore.

4.7 Transformation

4.7.1 Fluorescence values will be transformed using the arcsine transformation with a cofactor of 150.

4.8 High-dimensional flow analysis

4.8.1 Events will be clustered into cell-subsets using the PhenoGraph clustering algorithm with 30 nearest neighbours, utilising all cellular markers as input.

4.8.2 The phenotype of clusters will be characterised by marker enrichment modelling, dimension reduction techniques (UMAP, tSNE) and confirmed by biaxial plots of marker expression.

4.9 Differential state analysis

THIS IS A CONTROLLED DOCUMENT AND MUST NOT BE COPIED OR DISTRIBUTED WITHOUT AUTHORISATION BY FCI

4.9.1 Differential state analysis identifies differential expression of functional or other cell state markers within cell-subsets.

4.9.2 Differential state analysis will be conducted using diffcyt a computational framework for differential discovery analyses in high-dimensional cytometry data which utilises limma an R/Bioconductor software package.

4.9.3 Age, sex, and study name will be included as covariates in the design matrix.

4.9.4 Multiple comparisons will be handled with the Benjamini–Hochberg procedure to control the false discovery rate.

4.10 Potential risks and mitigation:

4.10.1 POTENTIAL RISK: The arcsine transformation with a cofactor of 150 may not produce a single negative population when visualised on a bi-axial plot.

4.10.2 RISK MITIGATION: In such cases, multiple cofactors will be tested for each marker and visualised as histograms. The cofactor that produces the clearest separation between a single negative population and positive populations will be selected.

4.10.3 POTENTIAL RISK: Overclustering or underclustering may occur, where single populations of cells are separated into multiple clusters, or multiple populations are combined into a single cluster.

4.10.4 RISK MITIGATION: Biaxial plots will be used to confirm that phenotypes associated with clusters conform to known cell subsets. In such cases, a combination of distinct dimension reduction methodologies can be used confirm the validity of the cluster configuration.

5.2 Comparisons with Healthy Recovered Adults – Legacy study

All analyses in Objectives 1-4 will be repeated with direct comparisons made against samples from healthy recovered adults from the UCLH-Crick Legacy study. Identical statistical analyses will be made following the above plan. Any deviation from the plan that is required to accelerate discovery will be included in the updated and amended Crick SAP.

27th October 2023

Dear MHRA/HRA/REC

This letter is to report an Urgent Safety Measure for the STIMULATE-ICP Study (IRAS ID:1004698) (EduraCT ID: 2021-006598-47). The Sponsor (University College London) has notified the MHRA safety assessor by telephone 27th October 2023.

There has been an update to the SmPC for Famotidine (attached)

This update includes the addition of further drugs that are contraindicated for concomitant use with Famotidine, requiring an update to the exclusion criteria within the protocol (section 6.8 Drug Interactions and Specific Contraindications) and the participant information sheets.

SmPC update:

Co-administration of posaconazole oral-suspension with famotidine should be avoided if possible, since famotidine may reduce the absorption of posaconazole oral-suspension during concomitant use.

Co-administration of famotidine with the tyrosine kinase inhibitors (TKIs) dasatinib, erlotinib, gefitinib, pazopanib may decrease plasma concentrations of TKIs resulting in lower efficacy, therefore co-administration of famotidine with these TKIs is not recommended. For further specific recommendations please refer to the product information of individual TKI medicinal products.

Our current exclusion criteria include Posaconazole, Dasatinib, and Pazopanib but does not include Erlotinib and Gefitinib.

This Urgent Safety Measure is for the addition of Erlotinib and Gefitinib to the exclusion criteria within the protocol and within the Partici0ant Information Sheets. An audit of all enrolled participants data and medications has shown the are no incidences of the use of either of these drugs within our enrolled participant group. All sites have been notified (25th October 2023) of the update and have confirmed receipt and acknowledgement of update.

We will submit the amended Protocol and Information leaflets within the regulatory period from identification.

Best wishes

Denise

Denise Forshaw

Director of Operations and Clinical Governance

Lancashire Clinical Trials Unit

University of Central Lancashire

Preston PR1 2HE

dforshaw@uclan.ac.uk

STIMULATE-ICP

A pragmatic, multi-centre, cluster randomised trial of an integrated care pathway with a nested, Phase III, open label, adaptive platform randomised drug trial in individuals with Long COVID

Participant Baseline Questionnaire

For each question, please choose the answer which applies to you. If this form is being completed by a friend/carer or member of the research team, please answer these questions on behalf of the **participant**. If you are unsure which answer to choose, please select the one which seems most applicable, rather than leaving that question blank.

You may find some of the questions repetitive but please answer them **ALL** to allow us to create a full picture of Long COVID and how it may affect you day-to-day.

Thank you for taking part. The next step is completing this questionnaire. It will be really helpful if you can complete and return this questionnaire as **soon as possible. If you wish to take part in the drug study this must be within the 7 days following your first clinic appointment and you must return it in person.** This is to ensure that if randomised to receive one of the study drugs you can collect and commence your medication.

The questionnaire may take up to 45-60 minutes to complete, depending on how much time you wish to spend on it. Please do this over a couple of days if it is easier. As you fill it out, don't spend too much time on any one question. There are no right or wrong answers.

If you take this questionnaire home with you to complete, please ensure that you have received a stamped addressed envelope from the research team to post back your completed questionnaire. Otherwise, please return or submit the completed questionnaire to the trial team in clinic.

A member of the trial team may contact you if the answer to any of the questions appear to have been omitted or not complete.

Your answers will be kept **completely confidential**.

Abbreviations used in this questionnaire:

A&E: Accident and Emergency

CFQ: Cognitive Failure Questionnaire

EQ-5D-5L: EuroQol Health related Quality of Life Questionnaire

FAS: Fatigue Assessment Score

GAD-7: General Anxiety Disorder (7 questions)

iPCQ: iMTA Productivity Cost Questionnaire

MRC: Medical Research Council

PCR: Polymerase Chain Reaction (test for diagnosing COVID-19)

PDQ: Perceived Deficit Questionnaire

PHQ: Public Health Questionnaire

SF-12: Short Form Questionnaire (12 questions)

WSAS: Work and Social Adjustment Scale

Please note: this questionnaire is 43 pages (including the cover page). Please ensure you have completed and answered all the questions before sending or submitting the form to the trial team.

INFORMATION ABOUT YOU (DEMOGRAPHICS)

1a. Is this questionnaire being completed by the participant?
If yes, please proceed to question 2.

☐ Yes
☐ No

1b. If no, what is the relationship of the person assisting the participant?

☐ Relative/friend
☐ Research Nurse/Research Practitioner
☐ Other *(please specify)* ______________________________

The following questions are all about the <u>participant</u>

2. What is your date of birth?

D D / M M / Y Y Y Y

3. What is your ethnicity?

<u>White</u>

☐ English, Welsh, Scottish, Northern Irish
☐ Irish
☐ Gypsy or Irish Traveller
☐ Any other White background *(please specify)* _______________

<u>Mixed / Multiple ethnic group</u>

☐ White and Black Caribbean
☐ White and Black African
☐ White and Asian
☐ Any other mixed or multiple ethnic background *(please specify)* _____________

<u>Asian</u>

☐ Indian
☐ Pakistani
☐ Bangladeshi
☐ Chinese
☐ Any other Asian background *(please specify)* _______________

<u>Black</u>

☐ African
☐ Caribbean
☐ Any Other Black, African or Caribbean background *(please specify)* __________

<u>Other Ethnic Group</u>

☐ Arab
☐ Any other ethnic group *(please specify)* _______________

4. What was your sex at birth?
- ☐ Male
- ☐ Female

5. How do you identify your gender?
- ☐ Man
- ☐ Woman
- ☐ Transgender Man
- ☐ Transgender Woman
- ☐ Other *(please specify)* ________________________________
- ☐ Prefer not to say

6. What is your religion?
- ☐ Christianity
- ☐ Islam
- ☐ Hinduism
- ☐ Sikhism
- ☐ Judaism
- ☐ Buddhism
- ☐ Other *(please specify)* ________________________________
- ☐ No religion
- ☐ Prefer not to say

7. What is your highest level of formal education?
- ☐ No formal educational qualifications
- ☐ Secondary education (O-Levels, GCSE, and or equivalent)
- ☐ School leaver (A Levels, Higher National BTEC, and or equivalent)
- ☐ Time served skills (e.g., painter, carpenter, tradesman)
- ☐ Undergraduate university degree
- ☐ Postgraduate degree
- ☐ Other (*please specify)* ________________________________

8a. Have you ever smoked?

If no, please proceed to question 9a.
- ☐ Yes
- ☐ No

8b. If yes, are you a:
- ☐ Current Smoker
- ☐ Former Smoker

8c. If you are a **current or former** smoker, please tick **all** that apply

☐ Cigarettes
☐ Vape / e-Cigarettes
☐ Cigar
☐ Recreational Drugs e.g. Cannabis

8d. If you are a **former** smoker, how many years did you smoke for? ____ years

And, what was your average daily use when smoking? _______________

And, when did you stop smoking? M M / Y Y Y Y

9a. Do you drink alcohol?
If no, please proceed to question 10a.

☐ Yes
☐ No

9b. If you drink alcohol, how many units, on average, per week do you consume?
e.g. A large glass of wine (250ml, ABV 12%) or a pint of beer/cider (ABV 5.2%) contains 3 units.

☐ 0 – 14 units
☐ 15 – 30 units
☐ More than 30 units

10a. **Before** you had COVID-19, did you leave the house for any activities e.g. work, exercise, shopping?
If "Not at all", please proceed to question 11a.

☐ Yes
☐ Not at all

10b. **Before** you had COVID-19, how would you mostly or predominantly get where you wanted to go?
*Please tick **all** relevant boxes.*

☐ Walking
☐ Cycling
☐ Driving own car
☐ Passenger to partner/spouse/relatives
☐ Public transport
☐ Taxi
☐ Other *(please describe)* ______________________

10c. **Before** you had COVID-19, in an average week, how often did you leave the house? *Please tick **one** box.*

☐ 1-2 times a week
☐ 3-4 times a week
☐ 5-7 times a week
☐ More than 7 times a week

10d. **Before** you had COVID-19, what were your reasons for leaving the house? *Please tick **all** relevant boxes.*

☐ Work
☐ Social (visiting friends/family, attending events)
☐ Caring for children and/or dependent adults
☐ Essential day-to-day matters (shopping, bank etc.)
☐ Exercising (e.g. walking)
☐ Other *(please describe)* ____________________________

11a. **At present**, do you leave the house for any activities e.g. work, exercise, shopping? *If "Not at all", please proceed to question 11e.*

☐ Yes
☐ Not at all

11b. **At present**, how do you mostly or predominantly get where you want to go? *Please tick **all** relevant boxes.*

☐ Walking
☐ Cycling
☐ Driving own car
☐ Passenger to partner/spouse/ relatives
☐ Public transport
☐ Taxi
☐ Other *(please describe)* ______________________

11c. **At present**, in an average week, how often do you leave the house? *Please tick **one** box.*

☐ 1-2 times a week
☐ 3-4 times a week
☐ 5-7 times a week
☐ More than 7 times a week

11d. **At present**, what are your reasons for leaving the house?
*Please tick **all** relevant boxes.*

☐ Work
☐ Social (visiting friends/family, attending events)
☐ Caring for children and/or dependent adults
☐ Essential day-to-day matters (shopping, bank etc.)
☐ Exercising (e.g. walking)
☐ Other *(please describe)* ______________________

11e. If you leave the house **less often** than you used to, what has influenced this?
*Please tick **all** relevant boxes.*

☐ Physical impact of Long COVID
☐ Psychological impact of Long COVID
☐ Financial impact of Long COVID
☐ Concerns over catching COVID-19
☐ Change in work pattern
☐ Other *(please specify)* _____________________
☐ Not applicable

12a. What is your current employment status?
*Please tick **all** relevant boxes.*

☐ Full-time – number of hours worked per week: _____
☐ Part-time – number of hours worked per week: _____
☐ 0 hours contract – average number of hours worked per week: _____
☐ Volunteer work – average number of hours worked per week: _____
☐ Full-time, phased return
☐ Part-time, phased return
☐ Employed and on short term sick leave
☐ Employed and on long term sick leave
☐ Unemployed because of long term sickness (receiving disability living allowance)
☐ Unemployed
☐ Retired
☐ Student
☐ Prefer not to say

12b. Has your employment status changed as a result of you having Long COVID?
If no, please proceed to question 12d.

☐ Yes
☐ No

12c. If yes, how has it changed?

*Please tick **all** relevant boxes.*

- ☐ Reduced hours – number of hours worked per week: _____
- ☐ Work from home – number of hours worked per week: _____
- ☐ Phased return – number of phased returns: _____
- ☐ Long term sick leave
- ☐ Changed occupation
- ☐ Change in working pattern
- ☐ Student
- ☐ Unemployed
- ☐ Retired

12d. Has your employment status changed as a result of COVID? *(e.g. the firm you were working for has gone bust).*

If no, please proceed to question 13a.

- ☐ Yes
- ☐ No

12e. If yes, how has it changed?

*Please tick **all** relevant boxes.*

- ☐ Reduced hours – number of hours worked per week: _____
- ☐ Work from home – number of hours worked per week: _____
- ☐ Phased return – number of phased returns: _____
- ☐ Long term sick leave
- ☐ Changed occupation
- ☐ Change in working pattern
- ☐ Student
- ☐ Unemployed
- ☐ Retired

13a. **Before** you had COVID-19, who did you live with?

*Please tick **all** relevant boxes.*

- ☐ Alone
- ☐ With spouse/partner
- ☐ With parents
- ☐ With financially dependent children
- ☐ With financially non-dependent children
- ☐ With friends/housemates
- ☐ Other *(please describe)* ______________________

13b. **Before** you had COVID-19**,** did you see/meet other people that you don't live with?

If "Not at all", please proceed to question 13d.

☐ Yes
☐ Not at all

13c. **Before** you had COVID-19, how often did you see/meet other people that you don't live with? *Please tick **one** box.*

☐ Most days
☐ Less than 3 days a week
☐ Less than once a month

13d. **Before** you had COVID-19**,** or before the pandemic**,** did you contact or communicate with other people that you don't live with?

If "Not at all", please proceed to question 14a.

☐ Yes
☐ Not at all

13e. **Before** you had COVID-19, or before the pandemic, how did you contact or communicate with other people who you don't live with? *Please tick **all** relevant boxes.*

☐ In-person
☐ Phone
☐ Email
☐ Texting
☐ Video-call (Facetime, WhatsApp, social media or similar)

14a. **At present**, who do you live with?

*Please tick **all** relevant boxes.*

☐ Alone
☐ With spouse/partner
☐ With parents
☐ With financially dependent children
☐ With financially non-dependent children
☐ With friends/housemates
☐ Other *(please describe)* ______________________

14b. **At present**, do you see/meet other people who you don't live with?

If "Not at all", please proceed to question 14d.

☐ Yes
☐ Not at all

14c. **At present**, how often do you see/meet other people who you don't live with?
*Please tick **one** box.*

- ☐ Most days
- ☐ Less than 3 days a week
- ☐ Less than once a month

14d. **At present**, do you contact or communicate with other people who you don't live with?
If "Not at all", please proceed to question 15.

- ☐ Yes
- ☐ Not at all

14e. **At present**, how do you contact or communicate with other people who you don't live with? *Please tick **all** relevant boxes.*

- ☐ In-person
- ☐ Phone
- ☐ Email
- ☐ Texting
- ☐ Video-call (Facetime, WhatsApp, social media or similar)

15a. What other illnesses **did** you have **before** contracting COVID-19? *Please tick **all** that apply*.

- ☐ Diabetes
- ☐ High blood pressure
- ☐ Heart disease
- ☐ Previous stroke
- ☐ Previous cancer
- ☐ Acid reflux
- ☐ Irritable bowel syndrome
- ☐ Gastrointestinal disorder
- ☐ Neurological disorder
- ☐ Migraine
- ☐ Asthma
- ☐ Blood clots (deep vein thrombosis or pulmonary embolus)
- ☐ Thyroid disorder
- ☐ Autoimmune illness (e.g. Rheumatoid arthritis)
- ☐ Previous diagnosis of Myalgic Encephalomyelitis / Chronic Fatigue Syndrome
- ☐ Anxiety disorder
- ☐ Depression
- ☐ Other *(please specify)* ______________________________

15b. What other illnesses **do you currently** have (apart from Long COVID)?

☐ Diabetes
☐ High blood pressure
☐ Heart disease
☐ Previous stroke
☐ Previous cancer
☐ Acid reflux
☐ Irritable bowel syndrome
☐ Gastrointestinal disorder
☐ Neurological disorder
☐ Migraine
☐ Asthma
☐ Blood clots (deep vein thrombosis or pulmonary embolus)
☐ Thyroid disorder
☐ Autoimmune illness (e.g. Rheumatoid arthritis)
☐ Previous diagnosis of Myalgic Encephalomyelitis / Chronic Fatigue Syndrome
☐ Anxiety disorder
☐ Depression
☐ Other *(please specify)* ______________________________

16a. If you are female, when was your last menstrual period?

Date of Last menstrual Period: D D / M M / Y Y Y Y

16b. Are your menstrual periods?

☐ Regular
☐ Irregular
☐ Absent

16c. If your menstrual periods are irregular or absent, what is the reason?

☐ This is normal for me
☐ Post-menopausal
☐ On a treatment which affects my cycle *(please specify)* _________________
☐ Changed since having COVID
☐ Other reason / condition which affects my cycle *(please specify)* _________________

16d. If you are female, are you planning on becoming pregnant in the next 16 weeks?

If yes, you will not be eligible to take part in the drug study. Please inform a member of the research team.

☐ Yes
☐ No

17a. What medication/s are you taking at present?

*Please tick **all** that apply*

☐ Anticoagulants (blood thinning medicine, e.g. Rivaroxaban)
☐ Aspirin
☐ Colchicine
☐ Ivabradine
☐ Beta Blockers e.g. Bisoprolol or Propranolol
☐ Hormone replacement therapy (HRT)
☐ Oral contraceptive pill
☐ Mirena coil
☐ Famotidine
☐ Antihistamines e.g. Loratadine/ Fexofenadine/ Cetirizine
☐ Anti-depressants
☐ Other *(please list any other medications you have been taking since you have had Long COVID in the box below. This includes any over the counter and non-prescribed medications)*

17b. Are you taking any vitamins or food supplements?

If no, please proceed to question 18a.

☐ Yes
☐ No

17c. If yes, please specify which vitamins or food supplements you are taking:

*Please tick **all** that apply*

☐ Multivitamin
☐ Iron
☐ Vitamin D
☐ Vitamin B12
☐ Niacin
☐ Melatonin
☐ Other *(please specify)* ________________________

18a. Please note your height if known: ______ metres **OR** ______ Feet ______ Inches

18b. Please note your current weight if known: ______ kg **OR** ______ Stone ______ Pounds

INFORMATION ABOUT YOUR COVID-19

19a. When did you first have COVID-19 symptoms?
If you are unsure of exact date, please estimate.

Start Date: D D / M M / Y Y Y Y

19b. How long were you initially ill for with COVID-19? (How many days was it before you started to feel you were getting better):

Number of days: _____

20a. When you first contracted COVID-19, was it confirmed by a COVID-19 test?
If no, please proceed to question 21a.

☐ No, not tested
☐ Yes, PCR test
☐ Yes, Lateral Flow Test
☐ Yes, blood serology (antibody test)

20b. What was the first date that your positive test was confirmed?
If you are unsure of exact date, please estimate.

D D / M M / Y Y Y Y

21a. Have you had re-infection with COVID-19?
If no, please proceed to question 22a.

☐ Yes
☐ No

21b. If yes, was it confirmed by a test?
If no, please proceed to question 22a.

☐ No, not tested
☐ Yes, PCR test
☐ Yes, Lateral Flow Test
☐ Yes, blood serology (antibody test)

21c. What was the first date that your re-infection was confirmed?
If you are unsure of exact date, please estimate

D D / M M / Y Y Y Y

21d. Have you had re-infection with COVID-19 after the second time?

If no, please proceed to question 22a.

☐ Yes
☐ No

21e. If yes, was it confirmed by a test?

If no, please proceed to question 22a.

☐ No, not tested
☐ Yes, PCR test
☐ Yes, Lateral Flow Test
☐ Yes, blood serology (antibody test)

21f. What was the first date that your re-infection was confirmed?

If you are unsure of exact date, please estimate

D D / M M / Y Y Y Y

22a. Have you had any other viral infections (e.g. Cold, Flu, Shingles) in the **3 months** prior to your initial COVID-19?

☐ Yes
☐ No

22b. If yes, please specify what viral infection(s) you had?

____

____

22c. Have you had any other viral infections (e.g. Cold, Flu, Shingles) **after** your initial COVID-19 infection?

☐ Yes
☐ No

22d. If yes, please specify what viral infection(s) you have had?

____

____

23a. Have you been vaccinated against COVID-19 infection?
If no, please proceed to question 24a.

☐ Yes
☐ No

23b. How many COVID-19 vaccinations did you have **prior** to the episode of **COVID** which led to your **Long COVID**?
If "0", please proceed to question 23d.

☐

23c. If 1 or more, please complete the table below for all the COVID-19 vaccines taken **prior** to the episode of **COVID** which led to your **Long COVID:**

**Doses**	1	2	3	4	5
**Dose Taken prior to COVID** *(please tick all that apply)*	☐	☐	☐	☐	☐
**Date (***If you are unsure of exact date, please estimate)*	DD / MM / YYYY	DD / MM / YYYY	DD / MM / YYYY	DD / MM / YYYY	DD / MM / YYYY
**Vaccine name** *(if other – please specify)*	☐Pfizer<br>☐Moderna<br>☐AstraZeneca<br>☐Other<br>*(please specify)*<br>__________	☐Pfizer<br>☐Moderna<br>☐AstraZeneca<br>☐Other<br>*(please specify)*<br>__________	☐Pfizer<br>☐Moderna<br>☐AstraZeneca<br>☐Other<br>*(please specify)*<br>__________	☐Pfizer<br>☐Moderna<br>☐AstraZeneca<br>☐Other<br>*(please specify)*<br>__________	☐Pfizer<br>☐Moderna<br>☐AstraZeneca<br>☐Other<br>*(please specify)*<br>__________
**Symptoms following vaccination** *(please tick all that apply)*	☐No symptoms<br>☐Fatigue<br>☐Fever<br>☐Muscle aches<br>☐Painful arm<br>☐Headache<br>☐Other<br>*(please specify)*<br>__________	☐No symptoms<br>☐Fatigue<br>☐Fever<br>☐Muscle aches<br>☐Painful arm<br>☐Headache<br>☐Other<br>*(please specify)*<br>__________	☐No symptoms<br>☐Fatigue<br>☐Fever<br>☐Muscle aches<br>☐Painful arm<br>☐Headache<br>☐Other<br>*(please specify)*<br>__________	☐No symptoms<br>☐Fatigue<br>☐Fever<br>☐Muscle aches<br>☐Painful arm<br>☐Headache<br>☐Other<br>*(please specify)*<br>__________	☐No symptoms<br>☐Fatigue<br>☐Fever<br>☐Muscle aches<br>☐Painful arm<br>☐Headache<br>☐Other<br>*(please specify)*<br>__________

23d. How many COVID-19 vaccinations did you have **after** the episode of **COVID** which led to your **Long COVID**?
If "0", please proceed to question 24a.

☐

23e. If 1 or more, please complete the table below for all the COVID-19 vaccines taken **after** the episode of **COVID** which led to your **Long COVID:**

**Doses**	1	2	3	4	5
**Dose Taken**<br>*(please tick all that apply)*	☐	☐	☐	☐	☐
**Date** (*If you are unsure of exact date, please estimate)*	DD / MM / YYYY	DD / MM / YYYY	DD / MM / YYYY	DD / MM / YYYY	DD / MM / YYYY
**Vaccine name**<br>*(if other – please specify)*	☐Pfizer<br>☐Moderna<br>☐AstraZeneca<br>☐Other<br>*(please specify)*<br>____________	☐Pfizer<br>☐Moderna<br>☐AstraZeneca<br>☐Other<br>*(please specify)*<br>____________	☐Pfizer<br>☐Moderna<br>☐AstraZeneca<br>☐Other<br>*(please specify)*<br>____________	☐Pfizer<br>☐Moderna<br>☐AstraZeneca<br>☐Other<br>*(please specify)*<br>____________	☐Pfizer<br>☐Moderna<br>☐AstraZeneca<br>☐Other<br>*(please specify)*<br>____________
**New symptoms following vaccination**<br>*(please tick all that apply)*	☐No symptoms<br>☐Fatigue<br>☐Fever<br>☐Muscle aches<br>☐Painful arm<br>☐Headache<br>☐Other<br>*(please specify)*<br>____________	☐No symptoms<br>☐Fatigue<br>☐Fever<br>☐Muscle aches<br>☐Painful arm<br>☐Headache<br>☐Other<br>*(please specify)*<br>____________	☐No symptoms<br>☐Fatigue<br>☐Fever<br>☐Muscle aches<br>☐Painful arm<br>☐Headache<br>☐Other<br>*(please specify)*<br>____________	☐No symptoms<br>☐Fatigue<br>☐Fever<br>☐Muscle aches<br>☐Painful arm<br>☐Headache<br>☐Other<br>*(please specify)*<br>____________	☐No symptoms<br>☐Fatigue<br>☐Fever<br>☐Muscle aches<br>☐Painful arm<br>☐Headache<br>☐Other<br>*(please specify)*<br>____________
**Long COVID symptoms change post vaccination**<br>*(please tick one)*	☐No change<br>☐Worsened<br>☐Improved	☐No change<br>☐Worsened<br>☐Improved	☐No change<br>☐Worsened<br>☐Improved	☐No change<br>☐Worsened<br>☐Improved	☐No change<br>☐Worsened<br>☐Improved
Duration of **Long COVID symptom** change.<br>*(if improved or worsened above)*	_______ days	_______ days	_______ days	_______ days	_______ days

24a. Have you got any vaccinations scheduled? (e.g. COVID-19/flu/tetanus)
If no, please proceed to question 24c.

☐ Yes
☐ No

24b. If yes, please add details below, and specify the date (if known):

Scheduled Vaccinations	Date Scheduled (DD/MM/YYYY)

24c. Have you had any other vaccinations since October 2019?
If no, please proceed to question 25a.

☐ Yes
☐ No

24d. If yes, please tick **all** that apply and specify the date (if known):

**Vaccine**	**(✓)**	**Date (DD/MM/YYYY)**	**Date Unknown (✓)**
Flu			
Other *(please specify)* 1)			
2)			
3)			

25a. Did you ever **visit** any A&E and/or outpatient hospital departments or COVID Assessment Centre during the first two weeks of your COVID-19?

If no, please proceed to question 25c.

☐ Yes
☐ No

If yes, please state how many times: _________

And

25b. Reasons for visiting: ____

25c. Did you ever **visit** any A&E and/or outpatient hospital departments due to your Long COVID symptoms?

If no, please proceed to question 26a.

☐ Yes
☐ No

If yes, please state how many times: ____________

And

25d. Reasons for visiting: ____

26a. Have you used a GP service since you have had COVID-19?

If no, proceed to question 27.

☐ Yes
☐ No

26b. If yes, please list the last ten reasons for contacting the GP surgery, who did you contact or get contacted by and the method of contact: face to face, online or by telephone?

Visit	Reason for contacting GP Surgery	Who did you see/contact? *(please tick one box for each visit)*		Method of Contact *(please tick one box for each visit)*		
		GP	Practice Nurse	In person	Online	Telephone
1						
2						
3						
4						
5						
6						

**Visit**	**Reason for contacting GP Surgery**	**Who did you see/contact?** *(please tick one box for each visit)*		**Method of Contact** *(please tick one box for each visit)*		
		**GP**	**Practice Nurse**	**In person**	**Online**	**Telephone**
7						
8						
9						
10						

26c. Have you used your GP service more than 10 times since you have had COVID-19?
If no, please proceed to question 27.

☐ Yes
☐ No

26d. If yes, please specify additional visits to your GP service and list the reasons in the table below:

Number of additional visits to your GP Practice in relation to Long COVID symptoms	Reasons for visiting

27. Regarding your symptoms; please answer the questions below to the best of your knowledge.

'Prior to COVID' refers to how you were feeling prior to contracting the illness.
'After COVID-19' *refers to how you were feeling 4 weeks after contracting the illness.*
'Now' refers to how you feel now (within last 4 weeks).

Rate the severity of each problem on a scale of 0-3, please ***circle*** *the relevant number:*
0 = None; no problem
1 = Mild problem; does not affect daily life
2 = Moderate problem; affects daily life to a certain extent
3 = Severe problem; affects all aspects of daily life; life-disturbing

**Symptom**		**Prior to COVID-19**	**After COVID-19** *(At least 4 weeks after)*	**Now** *(Within the last 4 weeks)*
Breathlessness	1) At rest	**0 1 2 3**	**0 1 2 3**	**0 1 2 3**
	2) Changing position e.g. from lying to sitting or sitting to lying	**0 1 2 3**	**0 1 2 3**	**0 1 2 3**
	3) On dressing yourself	**0 1 2 3**	**0 1 2 3**	**0 1 2 3**
	4) On walking up a flight of stairs	**0 1 2 3**	**0 1 2 3**	**0 1 2 3**
Cough/ throat sensitivity/ voicechange	5) Cough/throat sensitivity	**0 1 2 3**	**0 1 2 3**	**0 1 2 3**
	6) Sore throat	**0 1 2 3**	**0 1 2 3**	**0 1 2 3**
	7) Change of voice	**0 1 2 3**	**0 1 2 3**	**0 1 2 3**
Fatigue / (exhaustion not improved by rest)	8) Did you or do you get fatigued easily; fatigue levels in your usual daily activities	**0 1 2 3**	**0 1 2 3**	**0 1 2 3**
Smell/taste	9) Altered smell	**0 1 2 3**	**0 1 2 3**	**0 1 2 3**
	10) Altered taste	**0 1 2 3**	**0 1 2 3**	**0 1 2 3**
Pain/discomfort	11) Chest pain at rest	**0 1 2 3**	**0 1 2 3**	**0 1 2 3**
	12) Chest pain on exertion	**0 1 2 3**	**0 1 2 3**	**0 1 2 3**
	13) Chest pain after exertion	**0 1 2 3**	**0 1 2 3**	**0 1 2 3**
	14) Constant chest pain	**0 1 2 3**	**0 1 2 3**	**0 1 2 3**
	15) Chest pain on breathing in	**0 1 2 3**	**0 1 2 3**	**0 1 2 3**
	16) Chest tenderness/ heaviness or feeling of chest pressure	**0 1 2 3**	**0 1 2 3**	**0 1 2 3**
	17) Joint pain	**0 1 2 3**	**0 1 2 3**	**0 1 2 3**
	18) Muscle pain or aches	**0 1 2 3**	**0 1 2 3**	**0 1 2 3**
	19) Headache	**0 1 2 3**	**0 1 2 3**	**0 1 2 3**
	20) Abdominal pain	**0 1 2 3**	**0 1 2 3**	**0 1 2 3**
Cognition	21) Problems with concentration	**0 1 2 3**	**0 1 2 3**	**0 1 2 3**
	22) Problems with memory	**0 1 2 3**	**0 1 2 3**	**0 1 2 3**

Symptom		Prior to COVID-19	After COVID-19 *(At least 4 weeks after)*	Now *(Within the last 4 weeks)*
Cognition	23) Problems with planning for your daily activities	**0 1 2 3**	**0 1 2 3**	**0 1 2 3**
	24) Difficulty communicating (finding the right words or understanding others)	**0 1 2 3**	**0 1 2 3**	**0 1 2 3**
Palpitations/ dizziness	25) Fast heartbeat, feeling of missed beats or flutter in certain positions, activity or at rest	**0 1 2 3**	**0 1 2 3**	**0 1 2 3**
	26) Dizziness in certain positions, activity or at rest	**0 1 2 3**	**0 1 2 3**	**0 1 2 3**
Post-exertional malaise (worsening of symptoms; flare up of significant symptoms after physical or cognitive activity)	27) Crashing or relapse hours or days after physical, cognitive, or emotional exertion	**0 1 2 3**	**0 1 2 3**	**0 1 2 3**
	28) Duration of post-exertional symptoms **Less than 12 hours (<12h)** or **more than 12 hours (>12h)**	**<12h ☐**<br>**>12h ☐**	**<12h ☐**<br>**>12h ☐**	**<12h ☐**<br>**>12h ☐**
Anxiety/ mood	29) Feeling anxious	**0 1 2 3**	**0 1 2 3**	**0 1 2 3**
	30) Feeling depressed	**0 1 2 3**	**0 1 2 3**	**0 1 2 3**
	31) Having unwanted memories of your illness	**0 1 2 3**	**0 1 2 3**	**0 1 2 3**
	32) Having unpleasant dreams about your illness	**0 1 2 3**	**0 1 2 3**	**0 1 2 3**
	33) Trying to avoid thoughts or feelings about your illness	**0 1 2 3**	**0 1 2 3**	**0 1 2 3**
Sleep	34) Sleep problems, such as difficulty falling asleep, staying asleep or oversleeping	**0 1 2 3**	**0 1 2 3**	**0 1 2 3**

28. Have you had any of the following symptoms before you had COVID-19, after you had COVID-19 or within the last 4 weeks?

**Symptoms**	**I had this symptom before I had COVID-19**		**I had this symptom 4-6 weeks after COVID-19**		**I had this symptom in the last 4 weeks**	
	**Yes**	**No**	**Yes**	**No**	**Yes**	**No**
Fever						
Night sweats						
Hot flushes						
Skin rash/ discolouration of skin						
Skin sensation (numbness/tingling/itching/nerve pain)						
Numbness/change in sensation in arms or legs						
Hair loss						
Cold hands or feet						
Chilblains on fingers or toes (COVID fingers/toes)						
Itching eyes						
New allergies to medication, food etc please list them here and tick in relative boxes:<br>1.<br>2.<br>3.<br>If more than 3 please add them to (other symptoms box at the end of this table)						
Dry eyes/ redness of eyes						
Visual changes						
Sneezing						
Tinnitus						
Reduced hearing						
Fainting						

Symptoms	I had this symptom before I had COVID-19		I had this symptom 4-6 weeks after COVID-19		I had this symptom in the last 4 weeks	
	Yes	No	Yes	No	Yes	No
Vertigo						
Balance problems or falls						
Weakness or movement problems or coordination problems in limbs						
Hallucination						
Swelling of feet/ swelling of hands						
Easy bruising/ bleeding						
Waking up at night gasping for air (also called sleep apnoea)						
Difficulty swallowing solids						
Difficulty swallowing liquids						
Dry mouth/mouth ulcers						
Acid reflux/heartburn						
Change in appetite						
Nausea						
Unintentional weight loss						
Unintentional weight gain						
Constipation, diarrhoea, or bowel incontinence						
Bladder frequency, urgency, or incontinence						
Change in menstrual cycles or flow						
Reduced interest in sex						
Other symptoms: *(if yes, please specify)*:						

EQ-5D-5L

29. Under each heading, please tick the ONE box that best describes your health **before you had COVID-19**.

a**. Mobility**

☐ I had no problems in walking about
☐ I had slight problems in walking about
☐ I had moderate problems in walking about
☐ I had severe problems in walking about
☐ I was unable to walk about

b**. Self-Care**

☐ I had no problems washing or dressing myself
☐ I had slight problems washing or dressing myself
☐ I had moderate problems washing or dressing myself
☐ I had severe problems washing or dressing myself
☐ I was unable to wash or dress myself

c. **Usual Activities** *(e.g. work, study, housework, family or leisure activities)*

☐ I had no problems doing my usual activities
☐ I had slight problems doing my usual activities
☐ I had moderate problems doing my usual activities
☐ I had severe problems doing my usual activities
☐ I was unable to do my usual activities

d**. Pain / Discomfort**

☐ I had no pain or discomfort
☐ I had slight pain or discomfort
☐ I had moderate pain or discomfort
☐ I had severe pain or discomfort
☐ I had extreme pain or discomfort

e**. Anxiety / Depression**

☐ I was not anxious or depressed
☐ I was slightly anxious or depressed
☐ I was moderately anxious or depressed
☐ I was severely anxious or depressed
☐ I was extremely anxious or depressed

f. Please mark an X on the scale to indicate how your health was **before you had COVID-19**

We would like to know how good or bad your health was **before you had COVID-19**

This scale on the right is numbered from 0 – 100
100 means the <u>best</u> health you can imagine
0 means the <u>worst</u> health you can imagine

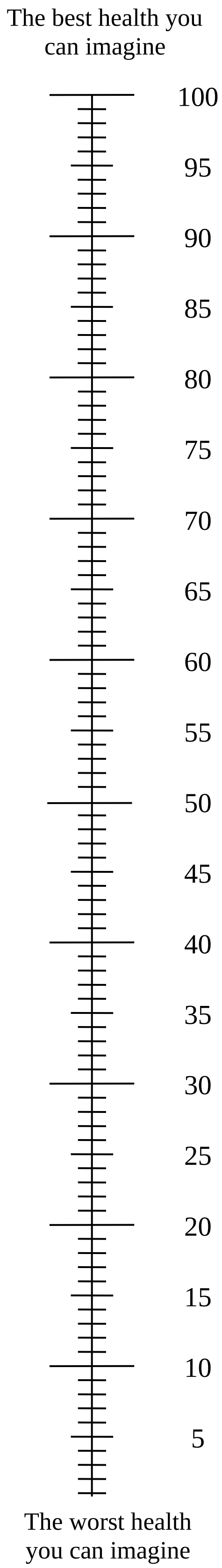

g. Now, write the number you marked on the scale in the box ☐

EQ-5D-5L

30. Under each heading, please tick the ONE box that best describes your health **in the last 4 weeks**.

a**. Mobility**

☐ I have no problems in walking about
☐ I have slight problems in walking about
☐ I have moderate problems in walking about
☐ I have severe problems in walking about
☐ I am unable to walk about

b**. Self-Care**

☐ I have no problems washing or dressing myself
☐ I have slight problems washing or dressing myself
☐ I have moderate problems washing or dressing myself
☐ I have severe problems washing or dressing myself
☐ I am unable to wash or dress myself

c**. Usual Activities** *(e.g. work, study, housework, family or leisure activities)*

☐ I have no problems doing my usual activities
☐ I have slight problems doing my usual activities
☐ I have moderate problems doing my usual activities
☐ I have severe problems doing my usual activities
☐ I am unable to do my usual activities

d**. Pain / Discomfort**

☐ I have no pain or discomfort
☐ I have slight pain or discomfort
☐ I have moderate pain or discomfort
☐ I have severe pain or discomfort
☐ I have extreme pain or discomfort

e**. Anxiety / Depression**

☐ I am not anxious or depressed
☐ I am slightly anxious or depressed
☐ I am moderately anxious or depressed
☐ I am severely anxious or depressed
☐ I am extremely anxious or depressed

f. Please mark an X on the scale to indicate how your health was **in the last 4 weeks.**

We would like to know how good or bad your health was
in the last 4 weeks.

This scale on the right is numbered from 0 – 100
100 means the best health you can imagine
0 means the worst health you can imagine

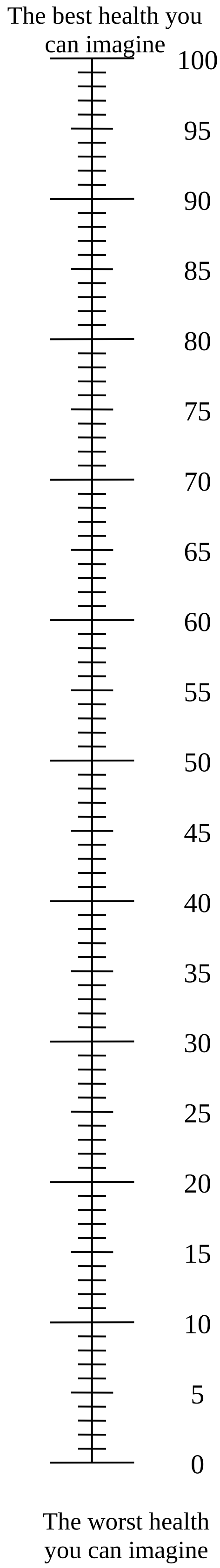

g. Now, write the number you marked on the scale in the box

SF-12

31. The following questions are about how you are feeling now. This survey asks for your views about your health. This information will help keep track of how you feel and how well you are able to do your usual activities. **Answer each question by choosing just one answer**. If you are unsure how to answer a question, please give the best answer you can.

a. In general, would you say your health is:

☐ Excellent
☐ Very good
☐ Good
☐ Fair
☐ Poor

The following questions are about activities you might do during a typical day. Does **your health now limit you** in these activities? If so, how much?

b. **Moderate activities** such as moving a table, pushing a vacuum cleaner, bowling, or playing golf

☐ Yes, limited a lot
☐ Yes, limited a little
☐ No, not limited at all

c. Climbing **several** flights of stairs

☐ Yes, limited a lot
☐ Yes, limited a little
☐ No, not limited at all

During the **past 4 weeks**, have you had any of the following problems with your work or other regular daily activities **as a result of your physical health?**

d. **Accomplished less** than you would like

☐ Yes
☐ No

e. Were limited in the **kind** of work or other activities

☐ Yes
☐ No

During the **past 4 weeks**, have you had any of the following problems with your work or other regular daily activities **as a result of any emotional problems** (such as feeling depressed or anxious)?

f. **Accomplished less** than you would like

☐ Yes
☐ No

g. Did work or activities **less carefully than usual**

☐ Yes
☐ No

h. During the **past 4 weeks**, how much **did pain interfere** with your normal work (including work outside the home and housework)?

☐ Not at all
☐ A little bit
☐ Moderately
☐ Quite a bit
☐ Extremely

These questions are about how you have been feeling during the **past 4 weeks**.
For each question, please give the one answer that comes closest to the way you have been feeling.
How much of the time during the **past 4 weeks**:

i. Have you felt calm and peaceful?

☐ All of the time
☐ Most of the time
☐ A good bit of the time
☐ Some of the time
☐ A little of the time
☐ None of the time

j. Did you have a lot of energy?

☐ All of the time
☐ Most of the time
☐ A good bit of the time
☐ Some of the time
☐ A little of the time
☐ None of the time

k. Have you felt downhearted and blue?

☐ All of the time
☐ Most of the time
☐ A good bit of the time
☐ Some of the time
☐ A little of the time
☐ None of the time

l. During the **past 4 weeks**, how much of the time has your **physical health or emotional problems** interfered with your social activities (like visiting friend, relatives, etc.)?

☐ All of the time
☐ Most of the time
☐ Some of the time
☐ A little of the time
☐ None of the time

MEDICAL RESEARCH COUNCIL DYSPNOEA SCORE

32. The following questions are about being breathless **before** having COVID-19, please select the most appropriate answer by ticking the box that applies to you:

**1**		I only got breathless with strenuous exercise
**2**		I got short of breath when hurrying on the level or walking up a slight hill
**3**		I walked slower than people of the same age on the level because of breathlessness or I had to stop for breath when walking on my own pace on the level
**4**		I stopped for breath after walking about 100 meters or after a few minutes on the level
**5**		I was too breathless to leave the house or I was breathless when dressing or undressing

33a. Are you **currently** suffering from breathlessness? (within the last 4 weeks).
If no, please proceed to question 34.

☐ Yes
☐ No

33b. If yes, please select the most appropriate answer by ticking the box that applies to you:

**1**		I only get breathless with strenuous exercise
**2**		I get short of breath when hurrying on the level or walking up a slight hill
**3**		I walk slower than people of the same age on the level because of breathlessness or I have to stop for breath when walking on my own pace on the level
**4**		I stop for breath after walking about 100 meters or after a few minutes on the level
**5**		I am too breathless to leave the house or I am breathless when dressing or undressing

STIMULATE ICP

Participant ID: [ ][ ] [ ][ ][ ][ ]

FAS

Please answer the following questions as how you usually felt before and after you had COVID-19

34. The following ten statements refer to how you usually feel. Per statement you can choose one out of five answer categories, varying from Never to Always. Please tick the answer to each question that is applicable to you. Please give an answer to each question, even if you do not have any complaints at the moment.

Please ensure you fill out **both sides** of the table, **before** and **after** you had COVID-19.

	**Please answer the following questions as how you usually felt before you had COVID-19**							**Please answer the following questions as how you usually feel now, after having COVID-19**				
		Never	Sometimes	Regularly	Often	Always		Never	Sometimes	Regularly	Often	Always
a	I am bothered by fatigue											
b	I get tired very quickly											
c	I don't do much during the day											
d	I have enough energy for everyday life											
e	Physically, I feel exhausted											
f	I have problems to start things											
g	I have problems to think clearly											
h	I feel no desire to do anything											
i	Mentally, I feel exhausted											
j	When I am doing something, I can concentrate quite well											

References: 1. Michielsen, H. J., De Vries, J., & Van Heck, G. L.(2003). Psychometric qualities of a brief self-ratedfatigue measure the fatigue assessment scale. Journalof Psychosomatic Research, 54, 345–352. 2.De Vries, J., Michielsen, H. J., Van Heck, G. L., & Drent, M. (2004). Measuring fatigue in sarcoidosis:the fatigue assessment scale (FAS). *British Journal of Health Psychology, 9* (3), 279–291.

PHQ-9

35. Over the **last 4 weeks**, how often have you been bothered by any of the following problems?
Please check the box which indicates your answers:

		Not at all	Several Days	More than half the days	Nearly every day
a	Little interest or pleasure in doing things				
b	Feeling down, depressed or hopeless.				
c	Trouble falling/staying asleep, sleeping too much				
d	Feeling tired or having little energy				
e	Poor appetite or overeating				
f	Feeling bad about yourself – or that you are a failure or have let yourself or your family down.				
g	Trouble concentrating on things, such as reading the newspaper or watching television				
h	Moving or speaking so slowly that other people could have noticed. Or the opposite – being so fidgety or restless that you have been moving around a lot more than usual.				
i	Thoughts that would be better off dead or of hurting yourself in some way				

j. If you checked off **any** problems, how **difficult** have these problems made it for you to do your work, take care of things at home, or get along with other people?
*Please tick **one**:*
☐ Not difficult at all
☐ Somewhat difficult
☐ Very difficult
☐ Extremely difficult

Reference:

From the Primary Care Evaluation of Mental Disorders Patient Health Questionnaire (PRIME-MD PHQ). The PHQ was developed by Drs. Robert L. Spitzer, Janet B.W. Williams, Kurt Kroenke and colleagues

GAD-7

36. Over the **last 4 weeks,** how often have you been bothered by the following problems?
Please tick to indicate your answers

		Not at all	Several Days	More than half the days	Nearly every day
a	Feeling nervous, anxious or on edge				
b	Not being able to stop or control worrying				
c	Worrying too much about different things				
d	Trouble relaxing				
e	Being so restless that it is hard to sit still				
f	Becoming easily annoyed or irritable				
g	Feeling afraid as if something awful might happen				

Reference:

The GAD-7 originates from Spitzer RL, Kroenke K, Williams JB, et al; A brief measure for assessing generalized anxiety disorder: the GAD-7. Arch Intern Med. 2006 May 22;166(10):1092-7. GAD-7

WSAS – AFTER COVID-19

37. People's problems (in this case Long COVID symptoms) sometimes affect their ability to do certain day-to-day tasks in their lives. To rate your problems, look at each section and determine on the scale provided how much Long COVID impairs your abilities and activities. This assessment is not intended to be a diagnosis. If you are concerned about your results in any way, please speak with a qualified health professional.

If you are retired or choose not to have a job for personal reasons unrelated to your problem (Long COVID), tick here: ☐ and answer questions b, c, d, and e.

'0' means 'not at all impaired' and '8' means very severely impaired to the point I can't work

0	1	2	3	4	5	6	7	8
Not at all		Slightly		Definitely		Markedly		Very Severely

	**Please write a number (0-8) in the box**
a. Because of my problem my **ability to work** is impaired.	☐
b. Because of my problem my **home management** (cleaning, tidying, shopping, cooking, looking after home or children, paying bills) is impaired	☐
c. Because of my problem my **social leisure activities** (with other people e.g. parties, bars, clubs, outings, visits, dating, home entertaining) are impaired	☐
d. Because of my problem my **private leisure activities** (done alone, such as reading, gardening, collecting, sewing, walking alone) are impaired	☐
e. Because of my problem my ability to form and maintain **close relationships** with others, including those I live with, is impaired	☐

Reference:

Mundt, J. C., I. M. Marks, et al. (2002). "The Work and Social Adjustment Scale: A simple measure of impairment in functioning." Br. J. Psychiatry 180: 461-4.

PCQ

If you are not currently employed, please tick here, ☐ and proceed to question 39.

38a. Have you been absent from your work in the past 4 weeks because you were ill?

☐ Yes, I was absent for ______ days *(only count the working days in the past 4 weeks)*
☐ No

38b. On how many working days have you suffered from physical or psychological problems during your work? *Just count the working days over the past 4 weeks.*

Working days: ________

5-ITEM VERSION (PDQ-5)

39. Everyone at some point experiences problems with memory, attention, or concentration. We are interested in examining how frequently individuals experience these types of problems. The following questions describe several situations in which a person may encounter problems with memory, attention and concentration. For each question, please tick the box to indicate how frequently you have experienced any of these matters during the **past week**. Please answer every question. If you are not sure which answer to select, please tick the one answer that comes closest to describing your experiences.

During the **past week,** how often did you…		**Never**	**Rarely**	**Sometimes**	**Often**	**Almost always**
a	Have trouble getting things organised?					
b	Have trouble concentrating on things like watching a television programme or reading a book?					
c	Forget the date unless you looked it up?					
d	Forget what you talked about after a telephone conversation?					
e	Feel like your mind went totally blank?					

CF QUESTIONNAIRE

40. The following questions are about minor mistakes which everyone makes from time to time, but some of which happen more often than others.

We want to know how often these things have happened to you **in the past 4 weeks**.

Please use the following scale.

0<br>Never	1<br>Very Rarely	2<br>Occasionally	3<br>Quite Often	4<br>Very Often

		Rating
1.	Do you read something and find you haven't been thinking about it and must read it again?	
2.	Do you find you forget why you went from one part of the house to the other?	
3.	Do you fail to notice signposts on the road?	
4.	Do you find you confuse right and left when giving directions?	
5.	Do you bump into people?	
6.	Do you find you forget whether you've turned off a light or a fire or locked the door?	
7.	Do you fail to listen to people's names when you are meeting them?	
8.	Do you say something and realise afterwards that it might be taken as insulting?	
9.	Do you fail to hear people speaking to you when you are doing something else?	
10.	Do you lose your temper and regret it?	
11.	Do you leave important letters unanswered for days?	
12.	Do you find you forget which way to turn on a road you know well but rarely use?	
13.	Do you fail to see what you want in a supermarket (although it's there)?	
14.	Do you find yourself suddenly wondering whether you have used a word correctly?	
15.	Do you have trouble making up your mind?	
16.	Do you find you forget appointments?	
17.	Do you forget where you put something like a newspaper or a book?	

We want to know how often these things have happened to you **in the past 4 weeks**.

Please use the following scale.

0<br>Never	1<br>Very Rarely	2<br>Occasionally	3<br>Quite Often	4<br>Very Often

18.	Do you find you accidentally throw away the thing you want and keep what you meant to throw away – as in the example of throwing away the matchbox and putting the used match in your pocket?	
19.	Do you daydream when you ought to be listening to something?	
20.	Do you find you forget people's names?	
21.	Do you start doing one thing at home and get distracted into doing something else (unintentionally)?	
22.	Do you find you can't quite remember something although it's "on the top of your tongue"?	
23.	Do you find you forget what you came to the shops to buy?	
24.	Do you drop things?	
25.	Do you find you can't think of anything to say?	

Reference:

Broadbent, D.E., Cooper, P.F., FitzGerald, P., & Parkes, K.R. (1982). The Cognitive Failures Questionnaire (CFQ) and its correlates. British Journal of Clinical Psychology, 21, 1-16.

41. The following questions are regarding your experience and public perception of you since you have had Long COVID symptoms. For the following questions, please tick the answer which is most applicable to you currently:

		Never	Rarely	Sometimes	Often	Always
a	Because of my illness, some people seemed uncomfortable with me					
b	I have felt embarrassed about my illness					
c	I felt embarrassed because of my physical limitations					
d	People have acted as if I am dishonest					
e	I have been treated with less respect than other people					
f	I have felt like I am very different from other people					
g	People tend to think Long COVID isn't a real illness					
h	People assume that having Long COVID is a sign of personal weakness					
i	People with Long COVID lose their jobs when their employers find out					
j	I worry that people may judge me when they learn I have Long COVID					
k	I am very careful who I tell that I have Long COVID					
l	I regret having told some people that I have Long COVID					

42a. Thinking about information and treatment for Long COVID, do you access digital information available via Apps and websites?
If no, please proceed to question 43.

☐ Yes
☐ No

42b. If yes, how often do you access the information available on websites or interact with digital Apps?
☐ Daily
☐ Weekly
☐ Once a month

42c. If accessing digital information, which website do you mostly access and or which App do you interact with to gain information and support for managing your Long COVID symptoms?

Please state the name of the App and/or the websites: ______________________

43a. The following space is for any further information or comments that you would like to add:

43b. The date this form was completed: D D / M M / Y Y Y Y

Thank you for your time and participation completing this questionnaire

FOR RESEARCH TEAM USE ONLY

44. Please record the date of the 12 week scheduled visit: D D / M M / Y Y Y Y

45a. Has the participant consented to take part in the drug arm of the trial?
If no, please skip to the end of this section.
☐ Yes
☐ No

45b. If yes, have you checked the participant's current medication use against the contraindicated medication list?
This must be yes to proceed to randomisation.
☐ Yes
☐ No

45c. Was a washout period required prior to randomisation?
If no, please proceed to question 45e.
☐ Yes
☐ No

45d. If yes to 45c, what is the name of the drug(s) that were washed out?

Name of the drug:________________ Period of wash out (in days): ____________

Name of the drug:________________ Period of wash out (in days): ____________

45e. Has the participant been randomised on Sealed Envelope?
☐ Yes
☐ No

45f. Was the participant randomised to IMP?
If no, please proceed to the end of the section.
☐ Yes
☐ No

45f(i). If yes, please tick which drug arm the participant was randomised to:
☐ Loratadine + Famotidine
☐ Colchicine
☐ Rivaroxaban

45f(ii). Date of dispensation: D D / M M / Y Y Y Y

45g. Please record the date of the first dose of IMP: D D / M M / Y Y Y Y
(*This date will be used to calculate the week 12 and week 24 visit dates)*

45h. Was the treatment delayed or interrupted?
☐ Yes
☐ No

45i. If yes, please provide a reason why:

____

If you are a member of the Research Team who has assisted in completing this questionnaire, please add your initials and the date below:

Initials: ☐ Date: D D / M M / Y Y Y Y

Initials of Research Staff receiving the questionnaire, and the date received:

Initials: ☐ Date: D D / M M / Y Y Y Y

Initials of Research Staff who checked this questionnaire for completion, and the date checked:

Initials: ☐ Date: D D / M M / Y Y Y Y

Table (a): CONSORT 2010 checklist of information to include when reporting a cluster randomised trial

Section/Topic	Item No	Standard Checklist item	Extension for cluster designs	Page No *
**Title and abstract**				
	1a	Identification as a randomised trial in the title	Identification as a cluster randomised trial in the title	1
	1b	Structured summary of trial design, methods, results, and conclusions (for specific guidance see CONSORT for abstracts)[1,2]	See table 2	3
**Introduction**				
**Background and objectives**	2a	Scientific background and explanation of rationale	Rationale for using a cluster design	4-5
	2b	Specific objectives or hypotheses	Whether objectives pertain to the cluster level, the individual participant level or both	5
**Methods**				
**Trial design**	3a	Description of trial design (such as parallel, factorial) including allocation ratio	Definition of cluster and description of how the design features apply to the clusters	26
	3b	Important changes to methods after trial commencement (such as eligibility criteria), with reasons		28-29
**Participants**	4a	Eligibility criteria for participants	Eligibility criteria for clusters	27
	4b	Settings and locations where the data were collected		27
**Interventions**	5	The interventions for each group with sufficient details to allow replication, including how and when they were actually administered	Whether interventions pertain to the cluster level, the individual participant level or both	27-28
**Outcomes**	6a	Completely defined pre-specified primary and	Whether outcome measures pertain to the cluster level, the	28

		secondary outcome measures, including how and when they were assessed	individual participant level or both	
	6b	Any changes to trial outcomes after the trial commenced, with reasons		28
**Sample size**	7a	How sample size was determined	Method of calculation, number of clusters(s) (and whether equal or unequal cluster sizes are assumed), cluster size, a coefficient of intracluster correlation (ICC or *k*), and an indication of its uncertainty	29
	7b	When applicable, explanation of any interim analyses and stopping guidelines		n/a
**Randomisation:**				
**Sequence generation**	8a	Method used to generate the random allocation sequence		27
	8b	Type of randomisation; details of any restriction (such as blocking and block size)	Details of stratification or matching if used	27
**Allocation concealment mechanism**	9	Mechanism used to implement the random allocation sequence (such as sequentially numbered containers), describing any steps taken to conceal the sequence until interventions were assigned	Specification that allocation was based on clusters rather than individuals and whether allocation concealment (if any) was at the cluster level, the individual participant level or both	28
**Implementation**	10	Who generated the random allocation sequence, who enrolled participants, and who assigned participants to interventions	Replace by 10a, 10b and 10c	27
	10a		Who generated the random allocation sequence, who enrolled clusters, and who assigned clusters to interventions	27

	10b		Mechanism by which individual participants were included in clusters for the purposes of the trial (such as complete enumeration, random sampling)	27
	10c		From whom consent was sought (representatives of the cluster, or individual cluster members, or both), and whether consent was sought before or after randomisation	27
**Blinding**	11a	If done, who was blinded after assignment to interventions (for example, participants, care providers, those assessing outcomes) and how		27
	11b	If relevant, description of the similarity of interventions		27
**Statistical methods**	12a	Statistical methods used to compare groups for primary and secondary outcomes	How clustering was taken into account	29-30
	12b	Methods for additional analyses, such as subgroup analyses and adjusted analyses		30-31
**Results**				
**Participant flow (a diagram is strongly recommended)**	13a	For each group, the numbers of participants who were randomly assigned, received intended treatment, and were analysed for the primary outcome	For each group, the numbers of clusters that were randomly assigned, received intended treatment, and were analysed for the primary outcome	6, Figure 1
	13b	For each group, losses and exclusions after randomisation, together with reasons	For each group, losses and exclusions for both clusters and individual cluster members	6, Figure 1
**Recruitment**	14a	Dates defining the periods of recruitment and follow-up		6

	14b	Why the trial ended or was stopped		6
**Baseline data**	15	A table showing baseline demographic and clinical characteristics for each group	Baseline characteristics for the individual and cluster levels as applicable for each group	15, Table 1
**Numbers analysed**	16	For each group, number of participants (denominator) included in each analysis and whether the analysis was by original assigned groups	For each group, number of clusters included in each analysis	6
**Outcomes and estimation**	17a	For each primary and secondary outcome, results for each group, and the estimated effect size and its precision (such as 95% confidence interval)	Results at the individual or cluster level as applicable and a coefficient of intracluster correlation (ICC or k) for each primary outcome	7
	17b	For binary outcomes, presentation of both absolute and relative effect sizes is recommended		7-8
**Ancillary analyses**	18	Results of any other analyses performed, including subgroup analyses and adjusted analyses, distinguishing pre-specified from exploratory		8-9
**Harms**	19	All important harms or unintended effects in each group (for specific guidance see CONSORT for harms[3])		8
**Discussion**				
**Limitations**	20	Trial limitations, addressing sources of potential bias, imprecision, and, if relevant, multiplicity of analyses		10
**Generalisability**	21	Generalisability (external validity, applicability) of the trial findings	Generalisability to clusters and/or individual participants (as relevant)	10,12
**Interpretation**	22	Interpretation consistent with results, balancing benefits and harms, and		10-11

		considering other relevant evidence	
**Other information**			
**Registration**	23	Registration number and name of trial registry	3
**Protocol**	24	Where the full trial protocol can be accessed, if available	Supplementary file
**Funding**	25	Sources of funding and other support (such as supply of drugs), role of funders	3

** Note: page numbers optional depending on journal requirements*

Table (b): Extension of CONSORT for abstracts to reports of cluster randomised trials

Item	Standard Checklist item	Extension for cluster trials	
**Title**	Identification of study as randomised	Identification of study as cluster randomised	**1**
**Trial design**	Description of the trial design (e.g. parallel, cluster, non-inferiority)		26
**Methods**			
**Participants**	Eligibility criteria for participants and the settings where the data were collected	Eligibility criteria for clusters	**27**
**Interventions**	Interventions intended for each group		27
**Objective**	Specific objective or hypothesis	Whether objective or hypothesis pertains to the cluster level, the individual participant level or both	**4**
**Outcome**	Clearly defined primary outcome for this report	Whether the primary outcome pertains to the cluster level, the individual participant level or both	**28**
**Randomization**	How participants were allocated to interventions	How clusters were allocated to interventions	**27**
**Blinding (masking)**	Whether or not participants, care givers, and those assessing the outcomes were blinded to group assignment		26,27
**Results**			
**Numbers randomized**	Number of participants randomized to each group	Number of clusters randomized to each group	**6**
**Recruitment**	Trial status[1]		6
**Numbers analysed**	Number of participants analysed in each group	Number of clusters analysed in each group	**6, 15**
**Outcome**	For the primary outcome, a result for each group and the estimated effect size and its precision	Results at the cluster or individual participant level as applicable for each primary outcome	**7**
**Harms**	Important adverse events or side effects		**8**
**Conclusions**	General interpretation of the results		
**Trial registration**	Registration number and name of trial register		**3**
**Funding**	Source of funding		**13**

[1] Relevant to Conference Abstracts

REFERENCES

[1] Hopewell S, Clarke M, Moher D, Wager E, Middleton P, Altman DG, et al. CONSORT for reporting randomised trials in journal and conference abstracts. *Lancet* 2008, 371:281-283

[2] Hopewell S, Clarke M, Moher D, Wager E, Middleton P, Altman DG at al (2008) CONSORT for reporting randomized controlled trials in journal and conference abstracts: explanation and elaboration. *PLoS Med* 5(1): e20

[3] Ioannidis JP, Evans SJ, Gotzsche PC, O'Neill RT, Altman DG, Schulz K, Moher D. Better reporting of harms in randomized trials: an extension of the CONSORT statement. *Ann Intern Med* 2004; 141(10):781-788.
