# Supplementary material for: Integrated care pathway in individuals with Long COVID: STIMULATE-ICP, a cluster-randomized, phase 3 trial
Source: Nat Med. 2026 Jul 28;32(8):3040–7. doi: 10.1038/s41591-026-04552-x (PMC13472957; doi:10.1038/s41591-026-04552-x)
Supplement: Supplementary file 2 [file 41591_2026_4552_MOESM2_ESM.pdf]

nature portfolio

Corresponding author(s): Amitava Banerjee

Last updated by author(s): Jun 25, 2026

Reporting Summary

Nature Portfolio wishes to improve the reproducibility of the work that we publish. This form provides structure for consistency and transparency in reporting. For further information on Nature Portfolio policies, see our Editorial Policies and the Editorial Policy Checklist.

Statistics

For all statistical analyses, confirm that the following items are present in the figure legend, table legend, main text, or Methods section.

n/a	Confirmed	
☐	☒	The exact sample size (*n*) for each experimental group/condition, given as a discrete number and unit of measurement
☐	☒	A statement on whether measurements were taken from distinct samples or whether the same sample was measured repeatedly
☐	☒	The statistical test(s) used AND whether they are one- or two-sided<br>*Only common tests should be described solely by name; describe more complex techniques in the Methods section.*
☐	☒	A description of all covariates tested
☐	☒	A description of any assumptions or corrections, such as tests of normality and adjustment for multiple comparisons
☐	☒	A full description of the statistical parameters including central tendency (e.g. means) or other basic estimates (e.g. regression coefficient) AND variation (e.g. standard deviation) or associated estimates of uncertainty (e.g. confidence intervals)
☐	☒	For null hypothesis testing, the test statistic (e.g. F, t, r) with confidence intervals, effect sizes, degrees of freedom and P value noted<br>*Give P values as exact values whenever suitable.*
☐	☒	For Bayesian analysis, information on the choice of priors and Markov chain Monte Carlo settings
☐	☒	For hierarchical and complex designs, identification of the appropriate level for tests and full reporting of outcomes
☐	☒	Estimates of effect sizes (e.g. Cohen's d, Pearson's r), indicating how they were calculated

Our web collection on statistics for biologists contains articles on many of the points above.

Software and code

Policy information about availability of computer code

Data collection	No bespoke code was developed for these analyses.
Data analysis	Analyses were conducted using statistical package Stata version 17. Any future code will be available at the study website: https://www.stimulate-icp.org/.

For manuscripts utilizing custom algorithms or software that are central to the research but not yet described in published literature, software must be made available to editors and reviewers. We strongly encourage code deposition in a community repository (e.g. GitHub). See the Nature Portfolio guidelines for submitting code & software for further information.

Data

Policy information about availability of data

All manuscripts must include a data availability statement. This statement should provide the following information, where applicable:

- Accession codes, unique identifiers, or web links for publicly available datasets
- A description of any restrictions on data availability
- For clinical datasets or third party data, please ensure that the statement adheres to our policy

Anonymised data (and supporting materials) are held at UCL. Access to the minimum dataset will be available by application to the CI (ami.banerjee@ucl.ac.uk), which will be reviewed and approved by the trial steering committee, aiming for a response within 2 months. These data will be available within 12 months of completion of the study (by December 2026) and will include individual-level participant data.

Research involving human participants, their data, or biological material

Policy information about studies with human participants or human data. See also policy information about sex, gender (identity/presentation), and sexual orientation and race, ethnicity and racism.

Reporting on sex and gender	Sex, gender and race/ethnicity information was collected by self -reported questionnaire. 6 people born male and 14 people born female gave an answer for gender other than man or woman. Since for some gender groups there were very small numbers, they are not provided in detail. Sex-specific data have been reported in the analyses.
Reporting on race, ethnicity, or other socially relevant groupings	Ethnicity was collected by self-reported questionnaire. Race was not collected. As detailed in table 1, of 1152 participants, 918 (85.6%) were of white ethnicity, and 48 (4.5%), 64 (6.0%), 25 (2.3%) and 17 (1.6%) were of Mixed, Asian, Black, and Other ethnicity respectively. There was insufficient statistical power to analyse outcomes by ethnicity.
Population characteristics	Individuals >18 years of age with Long COVID. Other population characteristics were collected by self -reported questionnaire or at interview.
Recruitment	From six specialised NHS Long COVID clinics in England.<br><br>PCNs within the areas served by the individual clinic will be cluster randomised to receive as usual care:<br>a. Coverscan™ + Living with COVID Recovery™ App.<br>b. Coverscan™ + Usual Care Self-Management Rehabilitation.<br>c. No Coverscan™ + Living with COVID Recovery™ App.<br>d. No Coverscan™+ Usual Care Self-Management Rehabilitation.<br>Site-specific research practitioners embedded in the Long COVID Clinics will identify new referrals who have not been previously hospitalised with COVID-19 and, if from within a PCN with Coverscan™ as usual care will arrange a scan appointment circa 4 weeks prior to the first Long COVID clinic appointment (to ensure scan report available at time of clinic assessment). This appointment is usual care within this PCN and will follow the same pathway as other clinical appointments.<br><br>Similarly, patients referred from PCNs allocated to "Living with COVID recovery™" app will be identified and referred to the physiotherapist/s assigned to delivery of this app, who will contact participants directly (as usual care). An appointment with the participant may be required prior to the Long COVID clinic to discuss the app wherever possible or within 7 -10 days of the first Long COVID clinic appointment.<br>In clinic areas that are not able to provide both Coverscan™ and the Living with COVID Recovery™ app, it will not be possible to randomise PCNs into the integrated care pathway clusters. All participants in these clinic areas will receive or will have received the usual care treatment pathway only, participants from these areas will be given the opportunity to participate in the individually randomised drug trial and data collection. They will also be asked to consider gifting blood samples.<br><br>Some patients interested in participating in the trial may have "brain fog" and, therefore, may require more time to consider taking part. We anticipate a gap of around 4-6 weeks between referral by the GP and patient's attendance to the appointment at the Long COVID clinic. Wherever possible, patients will be sent details of the study (the options to participate in all or part of the study) with the first appointment letter. This will include the study details, directions to the study website and contact details of both the research team based at their clinic and LCTU. If they wish to know more about the study prior to their first clinic appointment, then they can contact the team. We will also include details of any participation timeline, i.e., eligibility cannot be determined for the drug study prior to the first appointment, consent for the drug study should be no later than 7-10 days after the first appointment, consent for data only should be no later than 2 weeks after their clinic appointment with the option to gift blood up to 12 weeks post appointment. In instances where the potential participant has not received information, then information will be provided at time of approach or clinic visit. Previously see patients will receive an invitation letter and PIS outlining all sources of information and contact. The patient will be given as much time as they feel they need to make a decision regarding participation and may consent on that day if they wish, or return at a later time point within the required timeframes as per patient choice.<br>We will explain the value of data collection from all participants for the cluster trial and contribution to the blood sample collection in particular, to engage with those participants who may not be eligible for the drug trial but can make a valuable contribution to the study and its outcomes.<br>For patients where English may not be their first language, the information will contain details of how they can take part assisted by an English-speaking relative/friend.<br><br>Up to 226 Primary Care Network (PCN) sites will be allocated using blocked stratified randomisation using block size of 4 into one of the four 2x2 factorial allocations labelled as a, b, c, d (see below) in ratio 1:1:1:1:<br>a. Coverscan™ + Living with COVID Recovery™ App.<br>b. Coverscan™ + Usual Care Self-Management Rehabilitation.<br>c. No Coverscan™ + Living with COVID Recovery™ App.<br>d. No Coverscan™+ Usual Care Self- Management Rehabilitation.<br>Randomisation lists will be created by the study statisticians based at UCL and the Lancashire CTU.<br>To form the strata, PCNs linked to each clinic site will be ordered first by descending size of patient population and then defined by a single cut off value as large PCNs or small PCNs. Then large PCNs will be sorted by descending deprivation and small PCNs by ascending deprivation and defined by a single cut off value as more or less deprived. Within the four strata of PCNs: large, less deprived; large, more deprived; small, more deprived; and small, less deprived, treatment pathways will be allocated using randomised blocks of size 4. This will reduce bias and ensure that there is balance across the pathways by deprivation and population size within each clinic

site.

Ethics oversight: NHS Health Research Authority (IRAS 1004698) Research and Ethics Committee South Central- Berkshire (21/SC/0416) and Medicines Health Regulatory Agency approved the trial.

Note that full information on the approval of the study protocol must also be provided in the manuscript.

Field-specific reporting

Please select the one below that is the best fit for your research. If you are not sure, read the appropriate sections before making your selection.

☒ Life sciences ☐ Behavioural & social sciences ☐ Ecological, evolutionary & environmental sciences

For a reference copy of the document with all sections, see nature.com/documents/nr-reporting-summary-flat.pdf

Life sciences study design

All studies must disclose on these points even when the disclosure is negative.

Sample size

1130 (recruited: 1152).

Sample size was calculated using PASS v21.0.1(2021) for a 2x2 cluster factorial trial, powered to detect an interaction on the FAS scale between Coverscan™ and Living With COVID Recovery™ rather than main effects. Based on published data, a FAS standard deviation for individuals with LC was estimated at 6 units. Difference in mean FAS between people who had or had not recovered from LC was 9 points in a 2021 study. An interaction effect of 3 points in FAS could be detected with >90% power and (2-sided) significance level of 0.05 with 960 participants in 48 PCN clusters of 20 participants, assuming conservative intra-cluster correlation coefficient of 0.02. Assuming a conservative 15% dropout and equal missingness across arms, sample size was inflated by a factor of 100/85 to 1130.

Data exclusions

people without clinician-adjudicated Long COVID

Replication

6 study sites in Long COVID clinics.
The protocol and SAP were published before the study was completed to allow reproducibility of findings. No replicates have been performed for analyses.

Randomization

The rationale for cluster randomisation is published1,10. Randomisation used data available for each primary care network (PCN), the sum of list sizes of linked general practices (GP; larger ≥45000, smaller <45000) and median of the rank of deprivation indexes of linked GP postcodes (less deprived ≥14500, more deprived <14500), ensuring equity of access. PCN-level clustering allowed Coverscan™ and/or Living With COVID Recovery™ as an uplift to usual care in allocated PCNs, becoming an ICP within that PCN without need for individual consent. Research access to and collection of Coverscan™ and Living With COVID Recovery™ data was by individual consent on study entry (by research staff). Cluster randomisation (2x2 factorial design) provided four groups:

- Coverscan™+Usual Care Self-Management Rehabilitation ("multi-organ MRI").
- No Coverscan™+Living With COVID Recovery™ App ("digital rehabilitation").
- Coverscan™+Living With COVID Recovery™ App ("both").
- No Coverscan™+Usual Care Self- Management Rehabilitation ("neither").

Blinding

Like other large, pragmatic COVID-19 era trials, investigators and participants were unblinded. Blinding of analysts was impractical given original plans for interim analyses.

Reporting for specific materials, systems and methods

We require information from authors about some types of materials, experimental systems and methods used in many studies. Here, indicate whether each material, system or method listed is relevant to your study. If you are not sure if a list item applies to your research, read the appropriate section before selecting a response.

Materials & experimental systems

n/a	Involved in the study	
☒	☐	Antibodies
☒	☐	Eukaryotic cell lines
☒	☐	Palaeontology and archaeology
☒	☐	Animals and other organisms
☐	☒	Clinical data
☒	☐	Dual use research of concern
☒	☐	Plants

Methods

n/a	Involved in the study	
☒	☐	ChIP-seq
☒	☐	Flow cytometry
☒	☐	MRI-based neuroimaging

Clinical data

Policy information about clinical studies

All manuscripts should comply with the ICMJE guidelines for publication of clinical research and a completed CONSORT checklist must be included with all submissions.

Clinical trial registration: ISRCTN10665760

Study protocol: https://journals.plos.org/plosone/article?id=10.1371/journal.pone.0272472

Data collection: After informed consent and random treatment allocation, standard case record form (CRF) data was collected, e.g. new diagnoses and prescriptions. Follow-up was at 12 and 24 weeks.

Outcomes: The primary endpoint was fatigue(by mean Fatigue Assessment Scale, FAS) at 12 weeks. Secondary endpoints included FAS at 24 weeks and EQ-5D-5L visual assessment scale(EQ-VAS) at 12 and 24 weeks.

Plants

Seed stocks: n/a

Novel plant genotypes: n/a

Authentication: n/a
