# Supplementary material for: Integrated care pathway in individuals with Long COVID: STIMULATE-ICP, a cluster-randomized, phase 3 trial
Source: Nat Med. 2026 Jul 28;32(8):3040–7. doi: 10.1038/s41591-026-04552-x (PMC13472957; doi:10.1038/s41591-026-04552-x)
Supplement: Supplementary file 3 [file 41591_2026_4552_MOESM3_ESM.pdf]

nature portfolio

Peer Review File

Integrated care pathway in individuals with Long COVID: STIMULATE-ICP, a cluster randomized, phase 3 trial

Corresponding Author: Professor Amitava Banerjee

This file contains all reviewer reports in order by version, followed by all author rebuttals in order by version.

Version 0:

Reviewer comments:

Reviewer #1

(Remarks to the Author)
Comments for 'STIMULATE-ICP: A pragmatic, multi-centre, cluster randomised trial of an integrated care pathway in individuals with Long COVID'

This manuscript reports a cluster-randomized trial evaluating two integrated care pathway interventions for Long COVID (multi-organ MRI and digital rehabilitation) in specialist NHS Long COVID clinics. The primary outcome was fatigue measured by the Fatigue Assessment Scale (FAS) at 12 weeks. There was no benefit of multi-organ MRI at 12 weeks and no significant effect of digital rehabilitation at 12 weeks. At 24 weeks, digital rehabilitation showed a modest benefit on FAS and EQ-VAS. All groups, including usual care, showed substantial improvement over time, with mean FAS improving by 4.5 points from baseline to 12 weeks.
This is an important pragmatic trial addressing how to structure Long COVID care pathways. The cluster-randomized design is appropriate for evaluating service-level interventions, and the multi-site specialist clinic setting enhances generalizability to similar services. The overall improvement in all groups is encouraging, suggesting that specialist Long COVID clinic care may be beneficial. However, several methodological issues limit interpretation, including incomplete reporting of key cluster design parameters, limited detail on attrition and completion rates, and modest between-arm effect sizes whose clinical meaningfulness is uncertain. I have the following comments and suggestions for consideration.

MAJOR COMMENTS
1. This is a cluster-randomized trial with PCNs as the unit of randomisation, yet key cluster design and analysis elements are missing from the manuscript. The intracluster correlation coefficient (ICC) is fundamental for interpreting cluster trials and does not appear to be reported (apologies if I have missed it).

There is also no clear information on: (1) how many PCN clusters were randomized to each of the four arms, (2) cluster sizes (mean, range, and overall distribution), (3) whether there were important imbalances in cluster sizes across arms that might affect power or introduce bias, or (4) how the analysis accounted for clustering beyond a brief reference to "mixed effects models."

At a minimum, the manuscript should report: (a) the ICC for the primary outcome (FAS at 12 weeks), ideally with a 95% confidence interval, (b) the number of PCN clusters per arm, (c) the distribution of cluster sizes with basic descriptive statistics, and (d) a clear description of how clustering at PCN level was modelled in the primary analysis (e.g. PCN as a random effect, fixed effect, or use of cluster-robust standard errors), including how non-convergence was handled. It would also substantially strengthen the paper to indicate (e) whether the observed ICC was broadly consistent with that assumed in the sample size calculation, and (f) to provide simple cluster-level summaries of outcomes.

If feasible, a sensitivity analysis using an alternative approach to modelling the correlation structure. This information is essential for interpreting the validity and precision of the trial's estimates and for informing the design of future cluster-randomized trials in Long COVID populations.

2. This 2×2 factorial trial generates a substantial multiple-testing burden: two main effects and their interaction, assessed alongside numerous secondary outcomes (including FAS and EQ-VAS at 24 weeks), yet no clear strategy for handling multiplicity is described.

The protocol and SAP pre-specify FAS at 12 weeks as the single primary outcome, but the manuscript places considerable emphasis on the digital rehabilitation effect at 24 weeks (FAS difference −1.14, p=0.02; EQ-VAS difference 2.74, p=0.05), which are modest effects with borderline p-values that would likely be attenuated under any reasonable adjustment for multiple comparisons.
I would therefore recommend that the authors (a) more clearly distinguish confirmatory (primary) from exploratory (secondary, including 24-week) analyses, (b) explicitly state that p-values for secondary outcomes are unadjusted for multiplicity and interpret them cautiously, and (c) consider outlining a simple hierarchical or prioritised framework for secondary hypotheses, or at minimum acknowledge that the 24-week findings should be viewed as hypothesis-generating rather than definitive. The abstract and conclusions should be tempered accordingly.

3. The effect of digital rehabilitation at 24 weeks (-1.14 points on FAS) is statistically significant but may not be clinically meaningful. The authors cite an MCID of 4-5 points for FAS based on sarcoidosis literature. The observed effect size is well below this threshold.

This is critically important because: (1) statistical significance does not equal clinical significance, (2) patients and clinicians need to know if a 1-point improvement on a 10-50 scale is perceptible and meaningful, (3) the overall improvement in all groups (~4.5 points) suggests substantial benefit from specialist care alone, and (4) the incremental benefit of digital rehabilitation may not justify additional resources and implementation costs. Furthermore, the MCID from sarcoidosis may not apply to Long COVID fatigue.

The manuscript must: (a) explicitly acknowledge that effect size is below established MCID, (b) discuss uncertainty about MCID applicability to Long COVID population, (c) consider anchor-based MCID analysis using patients' global impression of change, (d) report proportion of participants achieving MCID as a secondary analysis, (e) present results in context of clinical meaningfulness, and (f) revise conclusions to acknowledge that clinical meaningfulness remains uncertain.

4. The multi-organ MRI intervention did not improve outcomes at either time point (e.g. FAS at 12 weeks, p≈0.69, with no signal of benefit at 24 weeks), yet this important negative finding receives little attention in the Discussion. The intervention involves resource-intensive, multi-organ imaging plus clinical decision support, but there was no measurable gain in fatigue or health-related quality of life compared with usual specialist care.

This raises several questions that are highly relevant for practice and policy: (1) what do these results imply about the clinical utility of comprehensive multi-organ imaging in Long COVID, especially when previous work has shown that such scans frequently detect abnormalities; (2) were the imaging findings actually actionable, and did they lead to meaningful changes in management; (3) how did clinicians and patients use or interpret the scan results (e.g. reassurance vs possible anxiety)

At present, the statement that improvements occurred “irrespective of an additional multi-organ MRI-based intervention” tends to downplay this null result. I would encourage the authors to devote more discussion to why MRI-guided care might not have improved outcomes, to comment (if data are available) on whether and how management changed following MRI, and to reflect on whether such imaging is useful in the absence of specific downstream treatments.

5. Baseline characteristics indicate a wide range of time since initial SARS-CoV-2 infection, with a median symptom duration of around one year and a broad interquartile range. However, the distribution of symptom duration is not fully described (e.g. by clinically meaningful categories), and there is little exploration of whether treatment effects differ by duration of illness. This matters because: (1) patients closer to symptom onset may have a higher likelihood of spontaneous improvement, (2) those with very long-standing symptoms may respond differently to interventions than those earlier in their disease course, and (3) mixing patients across very different stages of Long COVID may dilute or mask subgroup-specific treatment effects.

I would consider it important for the manuscript to: (a) report the distribution of time since infection/symptom duration more granularly (for example using pre-specified categories such as <6 months, 6–12 months, >12 months, if available), (b) clarify whether subgroup or sensitivity analyses by symptom duration were pre-specified in the SAP, and (c) if feasible, present exploratory analyses (e.g. interaction tests or stratified estimates) assessing whether the effects of multi-organ MRI and digital rehabilitation vary by duration of symptoms. Also, the discussion should acknowledge the potential for heterogeneity by symptom duration and the implications for generalisability and interpretation of average treatment effects.
6. All groups, including usual care, showed substantial improvement in fatigue (mean FAS reduced from 35.8 at baseline to 31.3 at 12 weeks, a ~4.5-point / 12.6% reduction). This is a large absolute change and appears broadly consistent with trajectories reported in prospective Long COVID cohorts (e.g. Heightman/Prashar et al., Ballouz et al.), where many patients improve over time even without trial interventions.

The discussion currently states that the observed reduction “argues against spontaneous recovery alone” and attributes improvement to specialist clinical care, but does not quantitatively compare the observed trajectory with published natural history data or describe in sufficient detail what “usual care” entailed. As a result, it is difficult for readers to disentangle the contributions of natural recovery, regression to the mean, and specialist-clinic ICP components from the incremental effect of the trialled interventions (multi-organ MRI and digital rehabilitation).

The manuscript should: (a) more fully describe usual care (content, intensity, frequency of contacts, multidisciplinary input), (b) explicitly compare observed improvement to published natural history cohorts without specialist LC clinics, (c) discuss the extent to which the 12-week improvement could reflect natural recovery and regression to the mean versus effects of the

NHS LC ICP, and (d) interpret the additional −1.14-point FAS effect of digital rehabilitation at 24 weeks in the context of the much larger absolute improvement seen in all arms, including usual care, considering whether similar outcomes might be achievable with less resource-intensive care models.

9. Digital rehabilitation showed no significant effect at 12 weeks (primary endpoint; $p \approx 0.25$) but did show statistically significant effects at 24 weeks (secondary endpoints; FAS $p=0.02$, EQ-VAS $p=0.05$). This pattern requires clearer explanation and cautious interpretation.

First, although 24-week outcomes were prespecified as secondary, the manuscript should be explicit about the hierarchy of endpoints and about the fact that the 24-week digital rehabilitation findings are secondary and exploratory. Second, it would be helpful to discuss plausible mechanisms for a delayed benefit (e.g. behavioural change, cumulative rehabilitation exposure) versus the possibility that the 24-week signal reflects chance in the context of multiple time-point and multiple outcome testing. Third, it is unclear whether engagement with the digital platform (e.g. log-ins, module completion, time spent) increased or persisted over time in a way that could explain a delayed effect, and whether there were differential dropout rates by arm at 24 weeks that might bias these estimates.

I would therefore recommend that the authors: (a) clarify in the Methods/Discussion that 24-week analyses were prespecified as secondary and should be interpreted as supportive rather than primary, (b) report available engagement metrics for the digital rehabilitation platform over time and examine whether patterns of engagement are consistent with a delayed benefit, (c) comment on whether differential attrition at 24 weeks could contribute to the observed effect, (d) discuss plausible biological or behavioural mechanisms for a delayed response, and (e) acknowledge that the 24-week digital rehabilitation effect, given its modest size, borderline p-values and the broader multiple-testing context, requires cautious interpretation and ideally independent replication.

MINOR COMMENTS
1. Please consider expanding Table 1 (or supplementary tables) to include individual-level socioeconomic markers, a more detailed LC symptom profile, and epidemiological context (infection period, reinfections), and then briefly discuss how these influence generalisability in the Discussion (complementing your major generalisability comment).

2. Please add intervention fidelity data (uptake, adherence and engagement metrics for the digital rehabilitation platform; proportion scanned among those allocated to MRI, types of MRI abnormalities, and whether these led to changes in management). This will help readers distinguish between ineffective interventions and potentially effective interventions that were not fully implemented.

3. Given the rich set of pre-specified secondary outcomes in the protocol, it would be helpful to present all major PROMs (EQ-5D-5L index, PHQ-9, WSAS, cognitive measures, etc.) by arm and timepoint, at least in a supplementary table, and explicitly note where results were null. This would reassure readers there is no selective outcome reporting and highlight whether the interventions had any broader impact beyond fatigue and EQ-VAS.

4. Provide the exact primary model specification used in the paper (outcome, fixed effects, any random terms, correlation structure), ideally as a short equation or clearly formatted text.

5. Explicitly state that the modelling strategy deviated from the SAP because of convergence problems, and briefly justify why the chosen fixed-effects approach is acceptable in the presence of clustering.

6. Confirm what, if any, diagnostics were done (residuals, influence, heteroscedasticity) and how missing data were handled for each model.

7. In the discussion, please consider:
a. Explicitly acknowledged 24 weeks as a relatively short horizon for a chronic post-viral condition.
b. Clarified which longer-term endpoints (e.g. healthcare utilisation, longer-term PROMs, mortality) are planned and when they will be reported.
c. Commented on whether the observed 24-week trajectories are expected to stabilise, improve or regress based on existing LC natural-history data.

8. In the discussion, for understanding of generalisability of the result, please consider:
Resource requirements for multi-organ MRI (scanner time, specialist reporting, clinical decision support) versus the null effect on patient-reported outcomes.
Infrastructure and equity issues for digital rehabilitation (internet/device access, digital literacy, technical support).
How feasible it would be to scale these ICP components beyond well-resourced specialist clinics, and whether simpler/cheaper diagnostics or rehabilitation approaches might achieve similar outcomes.

9. Please provide a little more detail on the PPIE panel, such as how members were recruited to represent diverse LC experiences; which aspects of design (outcomes, interventions, burden of follow-up) they influenced; whether they had input into interpretation of trial results; and what role they will play in dissemination.
10. Plesae consider the following results/figures to help readers to understanding the findings:
a. a trajectory plot of mean FAS by arm over time with 95% CIs (baseline, 12 and 24 weeks), to visualise the common improvement across arms and any separation at 24 weeks.
b. a forest plot of key subgroup analyses (e.g. symptom duration categories, baseline severity, sex, ethnicity), which will help

readers see consistency of effects.
c. a supplementary table summarising all pre-specified secondary outcomes by arm and timepoint, and a table or figure with intervention uptake/engagement metrics.

Reviewer #2

(Remarks to the Author)
Summary of the key results:
This multicenter trial found that Long COVID patients experienced reduced fatigue over 12 weeks with specialist integrated care, and neither multi-organ MRI nor digital rehabilitation added short-term benefit. However, digital rehabilitation showed modest improvements in fatigue and quality of life by 24 weeks, suggesting potential value for longer-term management.

Originality and significance:
There are multiple studies that have shown that skilled specialty and interdisciplinary care can reduce fatigue and other symptoms in Long COVID and this is also now a part of multiple clinical guidelines and consensus statements such as what was put forth by the AAPMR recently: https://onlinelibrary.wiley.com/doi/full/10.1002/pmrj.13397

The comparison of whether multi-organ MRI scans or digital rehabilitation can hold benefits to specialist care alone is novel, but the findings are not particularly significant to changing standard of care - it is unclear/not clearly stated how a multi-organ MRI scan was expected to enhance overall care coordination, so it is not particularly surprising that it did not improve treatment. In summary, while this is possibly one of the largest studies of its kind, the results are consistent with previously published work and does not yield major new insights into clinical care for Long COVID

Data & methodology:
In general, the methodologies for this study were mostly fine given the nature of the study, but there are still some major issues with the inability to fully capture the complexity of Long COVID cases. This dataset could have been much more informative and yield larger insights if more detailed symptom data were collected on each patient participant. Even from the viewpoint of capturing fatigue data, the authors should aim to better capture some of the elements around the fatigue they're observing (for example, exploring fatigue vs. sleepiness vs. post exertional malaise vs. exercise intolerance vs. autonomic nervous system dysfunction). In our sixth year of studying Long COVID, this level of detail is crucial for adequate phenotyping of Long COVID patients so that much more targeted insights can be gained from studies of this nature.

Appropriate use of statistics and treatment of uncertainties:
The statistical analysis plan is sound.

Conclusions:
The conclusions are appropriate, but modest for a journal of this caliber.

Suggested improvements:
There is a critical need for large studies of this nature to lead with subgroup and responder analysis. The value of non-specific studies of this nature is in the ability to understand the phenotypes of strong responders in the cohort so that we may more specifically target Long COVID cohorts in larger, well-controlled interventional clinical trials.

References:
References were appropriately cited

Clarity and context:
The manuscript was clear and appropriately written

Reviewer #3

(Remarks to the Author)
Long COVID remains a significant clinical challenge with no established treatments. This study, conducted by the STIMULATE-ICP consortium, addresses a critical gap in Long COVID care by conducting a large-scale cluster-randomised trial to determine whether Multi-organ magnetic resonance imaging (MRI; Coverscan™) and/or a digitally enabled rehabilitation platform (Living with COVID Recovery™) impacts clinical outcomes in patients. However, the open-label design with unblinded analysts, combined with reliance on self-reported fatigue measures at 12 and 24 weeks using the Fatigue Assessment Scale, raises concerns about potential bias in the estimation of treatment effects.

Major queries and comments:
1. My major concern about this trial is the open-label design, which includes analysts in combination with self-reported outcome measures, leading to massive potential for bias. They have four groups for this open-label study without any biomarker (which is a general issue with Long COVID studies), and the primary outcome is self-reported FAS. Given that Coverscan screening of 437 participants indicated that 105 (24%) participants had abnormal scan findings (lines 282-284), why did the authors not include participants with abnormal Coverscan at baseline and subdivided them into the four groups? The outcome could have been a change in organ function based on scan reports, which would be much less biased.
2. The authors highlight that digital rehabilitation caused a significant decrease in fatigue and improvement in general health

status at week 24 (lines 268-274). However, if this effect is real, should it also not be evident in the digital rehabilitation+MRI, especially given that the MRI-guided usual care arm did not exhibit any significant changes at week 24? Please explain.
3. Please speculate on the delayed benefit (week 24) of digital rehabilitation. No information on adherence to the Living With COVID Recovery™ app is provided. Is it possible that the delayed benefit reflects selection bias for those who adhered to the rehabilitation regimen?
4. The trial was pragmatic in design—appropriate for effectiveness evaluation—but provides minimal insight into mechanisms driving outcomes, particularly in the large impact from the usual care:
• Why does specialist LC clinic care improve fatigue?
• What specific clinic components drive improvement (are benefits related to diagnosis, multidisciplinary assessment, self-management education, psychological support, or other factors)?

Minor comment:
"Based on published data, a FAS standard deviation for 211 individuals with LC was estimated at 6 units." Lines 211-212. I do not see a reference.

Version 1:

Reviewer comments:

Reviewer #1

(Remarks to the Author)
Thank you for the update. I have no further comment.

Reviewer #3

(Remarks to the Author)
The authors seem to have responded to all critiques.

Only in the tracked version of the manuscript was I able to locate the main figures in deletion mode. In addition, in most responses, the authors state that they have addressed the comments, but do not clearly indicate where the changes were made or provide a brief summary of the revisions in the response letter. It was difficult to evaluate this revised manuscript and the figures.

Open Access This Peer Review File is licensed under a Creative Commons Attribution 4.0 International License, which permits use, sharing, adaptation, distribution and reproduction in any medium or format, as long as you give appropriate credit to the original author(s) and the source, provide a link to the Creative Commons license, and indicate if changes were made.
In cases where reviewers are anonymous, credit should be given to 'Anonymous Referee' and the source.
The images or other third party material in this Peer Review File are included in the article's Creative Commons license, unless indicated otherwise in a credit line to the material. If material is not included in the article's Creative Commons

license and your intended use is not permitted by statutory regulation or exceeds the permitted use, you will need to obtain permission directly from the copyright holder.
To view a copy of this license, visit https://creativecommons.org/licenses/by/4.0/

RESPONSE TO REVIEWERS: NMED-A146419

26th Nov 2025

Reviewer #1 (Remarks to the Author):

Comments for 'STIMULATE-ICP: A pragmatic, multi-centre, cluster randomised trial of an integrated care pathway in individuals with Long COVID'

This manuscript reports a cluster-randomized trial evaluating two integrated care pathway interventions for Long COVID (multi-organ MRI and digital rehabilitation) in specialist NHS Long COVID clinics. The primary outcome was fatigue measured by the Fatigue Assessment Scale (FAS) at 12 weeks. There was no benefit of multi-organ MRI at 12 weeks and no significant effect of digital rehabilitation at 12 weeks. At 24 weeks, digital rehabilitation showed a modest benefit on FAS and EQ-VAS. All groups, including usual care, showed substantial improvement over time, with mean FAS improving by 4.5 points from baseline to 12 weeks.
This is an important pragmatic trial addressing how to structure Long COVID care pathways. The cluster-randomized design is appropriate for evaluating service-level interventions, and the multi-site specialist clinic setting enhances generalizability to similar services. The overall improvement in all groups is encouraging, suggesting that specialist Long COVID clinic care may be beneficial. However, several methodological issues limit interpretation, including incomplete reporting of key cluster design parameters, limited detail on attrition and completion rates, and modest between-arm effect sizes whose clinical meaningfulness is uncertain. I have the following comments and suggestions for consideration.

We thank the reviewer for these helpful comments and have made detailed responses to each of the comments below.

MAJOR COMMENTS
1. This is a cluster-randomized trial with PCNs as the unit of randomisation, yet key cluster design and analysis elements are missing from the manuscript. The intracluster correlation coefficient (ICC) is fundamental for interpreting cluster trials and does not appear to be reported (apologies if I have missed it).

Thanks for this comment. The details are now provided in the revised manuscript. The ICC for was found to be small at close to 0.01 for the random effect of PCN.

There is also no clear information on: (1) how many PCN clusters were randomized to each of the four arms, (2) cluster sizes (mean, range, and overall distribution), (3) whether there were important imbalances in cluster sizes across arms that might affect power or introduce bias, or (4) how the analysis accounted for clustering beyond a brief reference to "mixed effects models."

These data are now available in the revised manuscript.

At a minimum, the manuscript should report: (a) the ICC for the primary outcome (FAS at 12 weeks), ideally with a 95% confidence interval, (b) the number of PCN clusters per arm, (c) the distribution of cluster sizes with basic descriptive statistics, and (d) a clear description of how

clustering at PCN level was modelled in the primary analysis (e.g. PCN as a random effect, fixed effect, or use of cluster-robust standard errors), including how non-convergence was handled. It would also substantially strengthen the paper to indicate (e) whether the observed ICC was broadly consistent with that assumed in the sample size calculation, and (f) to provide simple cluster-level summaries of outcomes.

These data are now available in the revised manuscript. ICC has been provided but because of its small size, the 95% CI for ICC has not been provided. As there were 122 PCN clusters randomised and 114 randomised PCN clusters at least one recruited participant, no cluster level summaries have been provided.

If feasible, a sensitivity analysis using an alternative approach to modelling the correlation structure. This information is essential for interpreting the validity and precision of the trial's estimates and for informing the design of future cluster-randomized trials in Long COVID populations.

Thanks for this comment. We have provided some detail in the revised manuscript. It has not been possible to include a sensitivity analysis of an alternative modelling of the correlation structure. The default correlation structure for mixed models in STATA is independent. In converging models for the primary outcome, the only random effect was the variance of random intercept for PCN so the assumed correlation structure is not critical.

2. This 2×2 factorial trial generates a substantial multiple-testing burden: two main effects and their interaction, assessed alongside numerous secondary outcomes (including FAS and EQ-VAS at 24 weeks), yet no clear strategy for handling multiplicity is described.
The protocol and SAP pre-specify FAS at 12 weeks as the single primary outcome, but the manuscript places considerable emphasis on the digital rehabilitation effect at 24 weeks (FAS difference −1.14, p=0.02; EQ-VAS difference 2.74, p=0.05), which are modest effects with borderline p-values that would likely be attenuated under any reasonable adjustment for multiple comparisons.

I would therefore recommend that the authors (a) more clearly distinguish confirmatory (primary) from exploratory (secondary, including 24-week) analyses, (b) explicitly state that p-values for secondary outcomes are unadjusted for multiplicity and interpret them cautiously, and (c) consider outlining a simple hierarchical or prioritised framework for secondary hypotheses, or at minimum acknowledge that the 24-week findings should be viewed as hypothesis-generating rather than definitive. The abstract and conclusions should be tempered accordingly.

We have addressed these points in detail in the revised manuscript.

3. The effect of digital rehabilitation at 24 weeks (-1.14 points on FAS) is statistically significant but may not be clinically meaningful. The authors cite an MCID of 4-5 points for FAS based on sarcoidosis literature. The observed effect size is well below this threshold.

This is critically important because: (1) statistical significance does not equal clinical significance, (2) patients and clinicians need to know if a 1-point improvement on a 10-50 scale is perceptible and meaningful, (3) the overall improvement in all groups (~4.5 points) suggests substantial benefit from specialist care alone, and (4) the incremental benefit of digital rehabilitation may not justify additional resources and implementation costs. Furthermore, the MCID from sarcoidosis may not apply to Long COVID fatigue.

The manuscript must: (a) explicitly acknowledge that effect size is below established MCID, (b) discuss uncertainty about MCID applicability to Long COVID population, (c) consider anchor-based MCID analysis using patients' global impression of change, (d) report proportion of participants achieving MCID as a secondary analysis, (e) present results in context of clinical meaningfulness, and (f) revise conclusions to acknowledge that clinical meaningfulness remains uncertain.

These points have been addressed in the revision.

We are aware that a difference between treatment groups of 1.14 points on FAS is statistically significant but may not be clinically meaningful. We accept that this different between a treatment group and usual care of <1.5 is small on a 40 FAS point scale.

We recognise that the MCID in the Long-Covid population could be different to the MCID in the Sarcoidosis paper, however it is likely to be similar. Willemien et al investigated the minimal clinically important difference (MCID) in the fatigue assessment scale (FAS) using several methods, including effect size (ES), standard error measurement (SEM) and an anchor-based approach using Physical Quality of Life (AB). The MCID values they derived for a group of sarcoidosis patients were 4.2 (ES), 3.6 (SEM) and 3.5 (AB) respectively and they concluded that the consistency of these results suggests that an MCID of about 4 would be reasonable for this group of sarcoidosis patients when planning trials involving the assessment of FAS scores. While we did not plan explicitly on the value of effect size, the use of change of 3 and SD at baseline of 6 corresponds with the effect size of 0.5 mentioned in the Willemien et al. paper. In our study, we decided to base the sample size power calculation on a difference of 3 for the main effects and interaction of the treatment groups in addition to any differences from baseline at 12 weeks and 24 weeks.

If we restrict to cluster trial participants, the means and standard deviations of the FAS scores were as below

	**Baseline and 12 weeks available**	**Baseline and 12 weeks available**	**Baseline and 12 weeks available**
**N=870**	**Baseline**	**12 weeks**	**Difference**
**Mean**	**35.65**	**31.27**	**-4.39**
**Standard deviation**	**8.16**	**9.33**	**6.80**

The reduction in means of FAS from baseline to 12 weeks was 4.39 with standard deviation of the differences of 6.80.

Although the reduction in FAS at 12 weeks was similar to the MCID for the Willemien study, the main effects and interaction for the treatment groups were small and not significant. At 24 weeks the difference from baseline was 4.29 but the treatment differences, although small in magnitude, were statistically significant. In view of the MCID for FAS, care needs to be taken in considering the clinical significance of any significant treatment effects at 24 weeks. This is explained in our paper.

Using the results of our study, the between patients pooled estimate of the standard deviation of FAS is approximately 8.8 and the correlation between pairs of FAS values at baseline and 12 weeks is 0.705. Using the SEM method to derive a MCID gives MCID = SEM = 4.8. It is likely, given the results in Willemien et al, that alternative methods of deriving MCID using ES or an anchor-based approach, would lead to similar values.

Reference

Willemien P.E. de Kleijn, Jolanda De Vries, Petal A.H.M. Wijnen, Marjolein Drent. Minimal (clinically) important differences for the Fatigue Assessment Scale in sarcoidosis. Respiratory Medicine (2011) 105, 1388e1395.

We have added the percentage of people in the four ICP combinations achieving a reduction of 3 or more points (59% overall) and 4 or more points (51% overall) to ensure that the context of change within each group including usual care is clear.

	Coverscan + App n= 302	Coverscan n= 288	App n= 258	Usual Care n= 304	All n= 1152
FAS change categories 3 week 12 Present n(%)	237(78.5)	205(71.2)	197(76.4)	231(76.0)	870(75.5)
3>= point reduction n(%)	144(60.8)	112(54.6)	118(59.9)	136(58.9)	510(58.6)
4>= point reduction n(%)	125(52.7)	95(46.3)	105(53.3)	122(52.8)	447(51.4)

Logistic regressions for a change in FAS of 3 or more and separately by 4 or more by treatment group have been performed as suggested by the reviewer. The results were very consistent at both 12 and 24 weeks with findings using measured FAS at 12 and 24 weeks. However, the document already presents many analyses, the MCID for FAS is not well established, and concerns have been raised about the interpretation of our findings of small magnitude. Adding in further post-hoc analyses might detract from the planned analyses, and might suggest that we are trying every approach to achieve a significant

result. While we are grateful for the reviewer's suggestion, we would prefer not to present these logistic regression analyses.

We had not intended to establish a different MCID for the Long-COVID population as part of this trial and so had not planned to include an anchor-based MCID analysis. We accept that this is possible using a parallel established measure, perhaps change in SF-12 physical which has a correlation (-0.33) to change in FAS, or another similar measure.

4. The multi-organ MRI intervention did not improve outcomes at either time point (e.g. FAS at 12 weeks, $p \approx 0.69$, with no signal of benefit at 24 weeks), yet this important negative finding receives little attention in the Discussion. The intervention involves resource-intensive, multi-organ imaging plus clinical decision support, but there was no measurable gain in fatigue or health-related quality of life compared with usual specialist care.

Thanks for this remark. We have added discussion of this negative finding in the revised manuscript.

This raises several questions that are highly relevant for practice and policy: (1) what do these results imply about the clinical utility of comprehensive multi-organ imaging in Long COVID, especially when previous work has shown that such scans frequently detect abnormalities; (2) were the imaging findings actually actionable, and did they lead to meaningful changes in management; (3) how did clinicians and patients use or interpret the scan results (e.g. reassurance vs possible anxiety)

At present, the statement that improvements occurred "irrespective of an additional multi-organ MRI-based intervention" tends to downplay this null result. I would encourage the authors to devote more discussion to why MRI-guided care might not have improved outcomes, to comment (if data are available) on whether and how management changed following MRI, and to reflect on whether such imaging is useful in the absence of specific downstream treatments.

This is an insightful comment which we have taken on board. We have added relevant text to the revised manuscript. Data were not available on management before and after MRI, particularly as the Coverscan ideally should have taken place before the first Long-COVID clinic appointment.

5. Baseline characteristics indicate a wide range of time since initial SARS-CoV-2 infection, with a median symptom duration of around one year and a broad interquartile range. However, the distribution of symptom duration is not fully described (e.g. by clinically meaningful categories), and there is little exploration of whether treatment effects differ by duration of illness. This matters because: (1) patients closer to symptom onset may have a higher likelihood of spontaneous improvement, (2) those with very long-standing symptoms may respond differently to interventions than those earlier in their disease course, and (3) mixing patients across very different stages of Long COVID may dilute or mask subgroup-specific treatment effects.

I would consider it important for the manuscript to: (a) report the distribution of time since infection/symptom duration more granularly (for example using pre-specified categories such as <6 months, 6–12 months, >12 months, if available), (b) clarify whether subgroup or sensitivity analyses by symptom duration were pre-specified in the SAP, and (c) if feasible, present exploratory analyses (e.g. interaction tests or stratified estimates) assessing whether the effects of multi-organ MRI and digital rehabilitation vary by duration of symptoms. Also, the discussion should acknowledge the potential for heterogeneity by symptom duration and the implications for generalisability and interpretation of average treatment effects.

We have tried to address all of these areas in the revised manuscript. Symptom duration, when fitted, did not change the main effects of either ICP.

6. All groups, including usual care, showed substantial improvement in fatigue (mean FAS reduced from 35.8 at baseline to 31.3 at 12 weeks, a ~4.5-point / 12.6% reduction). This is a large absolute change and appears broadly consistent with trajectories reported in prospective Long COVID cohorts (e.g. Heightman/Prashar et al., Ballouz et al.), where many patients improve over time even without trial interventions.

The discussion currently states that the observed reduction "argues against spontaneous recovery alone" and attributes improvement to specialist clinical care, but does not quantitatively compare the observed trajectory with published natural history data or describe in sufficient detail what "usual care" entailed. As a result, it is difficult for readers to disentangle the contributions of natural recovery, regression to the mean, and specialist-clinic ICP components from the incremental effect of the trialled interventions (multi-organ MRI and digital rehabilitation).

Thanks for this remark. We have tried to improve the discussion in the revision.

The manuscript should: (a) more fully describe usual care (content, intensity, frequency of contacts, multidisciplinary input), (b) explicitly compare observed improvement to published natural history cohorts without specialist LC clinics, (c) discuss the extent to which the 12-week improvement could reflect natural recovery and regression to the mean versus effects of the NHS LC ICP, and (d) interpret the additional −1.14-point FAS effect of digital rehabilitation at 24 weeks in the context of the much larger absolute improvement seen in all arms, including usual care, considering whether similar outcomes might be achievable with less resource-intensive care models.

We have now made these changes as per the above recommendations.

9. Digital rehabilitation showed no significant effect at 12 weeks (primary endpoint; $p \approx 0.25$) but did show statistically significant effects at 24 weeks (secondary endpoints; FAS $p=0.02$, EQ-VAS $p=0.05$). This pattern requires clearer explanation and cautious interpretation.

First, although 24-week outcomes were prespecified as secondary, the manuscript should be explicit about the hierarchy of endpoints and about the fact that the 24-week digital rehabilitation findings are secondary and exploratory. Second, it would be helpful to discuss

plausible mechanisms for a delayed benefit (e.g. behavioural change, cumulative rehabilitation exposure) versus the possibility that the 24-week signal reflects chance in the context of multiple time-point and multiple outcome testing. Third, it is unclear whether engagement with the digital platform (e.g. log-ins, module completion, time spent) increased or persisted over time in a way that could explain a delayed effect, and whether there were differential dropout rates by arm at 24 weeks that might bias these estimates.

I would therefore recommend that the authors: (a) clarify in the Methods/Discussion that 24-week analyses were prespecified as secondary and should be interpreted as supportive rather than primary, (b) report available engagement metrics for the digital rehabilitation platform over time and examine whether patterns of engagement are consistent with a delayed benefit, (c) comment on whether differential attrition at 24 weeks could contribute to the observed effect, (d) discuss plausible biological or behavioural mechanisms for a delayed response, and (e) acknowledge that the 24-week digital rehabilitation effect, given its modest size, borderline p-values and the broader multiple-testing context, requires cautious interpretation and ideally independent replication.

Thanks for these observations. We have made clarifications accordingly in the revised manuscript.

MINOR COMMENTS

1. Please consider expanding Table 1 (or supplementary tables) to include individual-level socioeconomic markers, a more detailed LC symptom profile, and epidemiological context (infection period, reinfections), and then briefly discuss how these influence generalisability in the Discussion (complementing your major generalisability comment).

We did not have availability of all of these data in our study. We have included further variables, where possible, in Table 1 in the revised manuscript.

2. Please add intervention fidelity data (uptake, adherence and engagement metrics for the digital rehabilitation platform; proportion scanned among those allocated to MRI, types of MRI abnormalities, and whether these led to changes in management). This will help readers distinguish between ineffective interventions and potentially effective interventions that were not fully implemented.

We have added these data, where available.

3. Given the rich set of pre-specified secondary outcomes in the protocol, it would be helpful to present all major PROMs (EQ-5D-5L index, PHQ-9, WSAS, cognitive measures, etc.) by arm and timepoint, at least in a supplementary table, and explicitly note where results were null. This would reassure readers there is no selective outcome reporting and highlight whether the interventions had any broader impact beyond fatigue and EQ-VAS.

These secondary outcomes will be the subject of further manuscript and could not all be included as suggested. WE have made this clear in the manuscript.

4. Provide the exact primary model specification used in the paper (outcome, fixed effects, any random terms, correlation structure), ideally as a short equation or clearly formatted text.

These data were available in the original draft but have been made clearer in the revision.

5. Explicitly state that the modelling strategy deviated from the SAP because of convergence problems, and briefly justify why the chosen fixed-effects approach is acceptable in the presence of clustering.

We have adjusted the manuscript wording accordingly.

6. Confirm what, if any, diagnostics were done (residuals, influence, heteroscedasticity) and how missing data were handled for each model.

We have added comments in the revised manuscript.

7. In the discussion, please consider:
a. Explicitly acknowledged 24 weeks as a relatively short horizon for a chronic post-viral condition.
b. Clarified which longer-term endpoints (e.g. healthcare utilisation, longer-term PROMs, mortality) are planned and when they will be reported.
c. Commented on whether the observed 24-week trajectories are expected to stabilise, improve or regress based on existing LC natural-history data.

All of these are valid points, which we have now addressed.

8. In the discussion, for understanding of generalisability of the result, please consider:
Resource requirements for multi-organ MRI (scanner time, specialist reporting, clinical decision support) versus the null effect on patient-reported outcomes.
Infrastructure and equity issues for digital rehabilitation (internet/device access, digital literacy, technical support).
How feasible it would be to scale these ICP components beyond well-resourced specialist clinics, and whether simpler/cheaper diagnostics or rehabilitation approaches might achieve similar outcomes.

Thanks for these comments. We have now added discussion in relation to these points.

9. Please provide a little more detail on the PPIE panel, such as how members were recruited to represent diverse LC experiences; which aspects of design (outcomes, interventions, burden of follow-up) they influenced; whether they had input into interpretation of trial results; and what role they will play in dissemination.

We have added this detail in the revised manuscript.

10. Plesae consider the following results/figures to help readers to understanding the findings:
a. a trajectory plot of mean FAS by arm over time with 95% CIs (baseline, 12 and 24 weeks), to visualise the common improvement across arms and any separation at 24 weeks.
b. a forest plot of key subgroup analyses (e.g. symptom duration categories, baseline severity, sex, ethnicity), which will help readers see consistency of effects.
c. a supplementary table summarising all pre-specified secondary outcomes by arm and timepoint, and a table or figure with intervention uptake/engagement metrics.

Where possible, we have added further requested detail in the revision.

Reviewer #2 (Remarks to the Author):

Summary of the key results:
This multicenter trial found that Long COVID patients experienced reduced fatigue over 12 weeks with specialist integrated care, and neither multi-organ MRI nor digital rehabilitation added short-term benefit. However, digital rehabilitation showed modest improvements in fatigue and quality of life by 24 weeks, suggesting potential value for longer-term management.

Originality and significance:
There are multiple studies that have shown that skilled specialty and interdisciplinary care can reduce fatigue and other symptoms in Long COVID and this is also now a part of multiple clinical guidelines and consensus statements such as what was put forth by the AAPMR recently: https://onlinelibrary.wiley.com/doi/full/10.1002/pmrj.13397

The comparison of whether multi-organ MRI scans or digital rehabilitation can hold benefits to specialist care alone is novel, but the findings are not particularly significant to changing standard of care - it is unclear/not clearly stated how a multi-organ MRI scan was expected to enhance overall care coordination, so it is not particularly surprising that it did not improve treatment. In summary, while this is possibly one of the largest studies of its kind, the results are consistent with previously published work and does not yield major new insights into clinical care for Long COVID.

Thanks to the reviewer for these insights. We would disagree that there are no clinically relevant implications from these results as negative findings can impact clinical practice.

Data & methodology:
In general, the methodologies for this study were mostly fine given the nature of the study, but there are still some major issues with the inability to fully capture the complexity of Long COVID cases. This dataset could have been much more informative and yield larger insights if more detailed symptom data were collected on each patient participant. Even from the viewpoint of capturing fatigue data, the authors should aim to better capture some of the elements around the fatigue they're observing (for example, exploring fatigue vs. sleepiness vs. post exertional

malaise vs. exercise intolerance vs. autonomic nervous system dysfunction). In our sixth year of studying Long COVID, this level of detail is crucial for adequate phenotyping of Long COVID patients so that much more targeted insights can be gained from studies of this nature.

Our study was funded in August 2021 (the application was submitted in March 2021) and the ethical approvals were all given in late 2021/early 2022. The full post exertional malaise questionnaire could not be included as it would have increased the burden of completing the already substantial baseline participant questionnaire, based on the patient and public involvement group input in our study. Also the protocol was already published. Moreover, we could not change data collection once the study had started and the protocol published. Nonetheless, we have added further detail in the revised manuscript.

Appropriate use of statistics and treatment of uncertainties:
The statistical analysis plan is sound.

Thanks for this supportive remark.

Conclusions:
The conclusions are appropriate, but modest for a journal of this caliber.

Suggested improvements:
There is a critical need for large studies of this nature to lead with subgroup and responder analysis. The value of non-specific studies of this nature is in the ability to understand the phenotypes of strong responders in the cohort so that we may more specifically target Long COVID cohorts in larger, well-controlled interventional clinical trials.

We have attempted some subgroup analysis in the revised manuscript. However, further subgroup analysis by detailed MRI findings and other metrics will be the subject of future analyses.

References:
References were appropriately cited

Clarity and context:
The manuscript was clear and appropriately written

Thanks for these remarks.

Reviewer #3 (Remarks to the Author):

Long COVID remains a significant clinical challenge with no established treatments. This study, conducted by the STIMULATE-ICP consortium, addresses a critical gap in Long COVID care by conducting a large-scale cluster-randomised trial to determine whether Multi-organ magnetic resonance imaging (MRI; Coverscan™) and/or a digitally enabled rehabilitation platform (Living

with COVID Recovery™) impacts clinical outcomes in patients. However, the open-label design with unblinded analysts, combined with reliance on self-reported fatigue measures at 12 and 24 weeks using the Fatigue Assessment Scale, raises concerns about potential bias in the estimation of treatment effects.

Major queries and comments:

1. My major concern about this trial is the open-label design, which includes analysts in combination with self-reported outcome measures, leading to massive potential for bias. They have four groups for this open-label study without any biomarker (which is a general issue with Long COVID studies), and the primary outcome is self-reported FAS. Given that Coverscan screening of 437 participants indicated that 105 (24%) participants had abnormal scan findings (lines 282-284), why did the authors not include participants with abnormal Coverscan at baseline and subdivided them into the four groups? The outcome could have been a change in organ function based on scan reports, which would be much less biased.

Thanks for this comment. The open-label design was pragmatic at that time in the pandemic and this protocol was published and the study approved by the funder, UK government and ethical review committees. Although it does have potential for bias, there are other aspects of our study design which make it more applicable to the real world than other Long COVID studies. The suggested Coverscan analysis was not prespecified analysis in our protocol, and unfortunately, we would not have sufficient statistical power to conduct this analysis. Further analysis of abnormal Coverscan and the nature of those abnormalities is planned in future research. Unfortunately, we cannot change the outcome in the trial.

2. The authors highlight that digital rehabilitation caused a significant decrease in fatigue and improvement in general health status at week 24 (lines 268-274). However, if this effect is real, should it also not be evident in the digital rehabilitation+MRI, especially given that the MRI-guided usual care arm did not exhibit any significant changes at week 24? Please explain.

Thanks for this comment. We have added text in the revised manuscript. We have attempted to make it clearer that the numbers given in the text represent three orthogonal effects: MRI vs. no MRI, digital rehabilitation vs. no digital rehabilitation and the interaction of the two other effects. They do not represent the differences between each of MRI only vs. usual care, digital rehabilitation only vs. usual care and both treatments together vs. usual care. For FAS at 24 weeks, the interaction term is not significant and this means that the small but statistically significant improvement from the offer of digital rehabilitation is similar, whether or not MRI is offered.

3. Please speculate on the delayed benefit (week 24) of digital rehabilitation. No information on adherence to the Living With COVID Recovery™ app is provided. Is it possible that the delayed benefit reflects selection bias for those who adhered to the rehabilitation regimen?

We have added detail regarding the Living With COVID Recovery™ app in the supplementary material of the revised manuscript.

4. The trial was pragmatic in design—appropriate for effectiveness evaluation—but provides minimal insight into mechanisms driving outcomes, particularly in the large impact from the usual care:

• Why does specialist LC clinic care improve fatigue?

Specialist LC clinic care may improve fatigue by several mechanisms, but we can only speculate on actual cause. We have added text to reflect these possibilities in the revised manuscript.

• What specific clinic components drive improvement (are benefits related to diagnosis, multidisciplinary assessment, self-management education, psychological support, or other factors)?

This is critical observation. Unfortunately, we do not have the detailed breakdown of clinic components by site. Our ancillary publication (Prashar et al. In press with BMJ 2026; and in submitted materials for peer review) adds detail about the UCLH clinic.

Minor comment:

"Based on published data, a FAS standard deviation for 211 individuals with LC was estimated at 6 units." Lines 211-212. I do not see a reference.

Thanks for the comment. We have added a reference in the revised manuscript.

RESPONSE TO REVIEWERS

19th Mar 2026

Reviewers' Comments:

Reviewer #1 (Remarks to the Author):

Thank you for the update. I have no further comment.

Thanks for all the very constructive reviews.

Reviewer #3 (Remarks to the Author):

The authors seem to have responded to all critiques.

Only in the tracked version of the manuscript was I able to locate the main figures in deletion mode. In addition, in most responses, the authors state that they have addressed the comments, but do not clearly indicate where the changes were made or provide a brief summary of the revisions in the response letter. It was difficult to evaluate this revised manuscript and the figures.

Figures now clearly provided.
